# The genome of the colonial hydroid *Hydractinia* reveals that their stem cells use a toolkit of evolutionarily shared genes with all animals

**DOI:** 10.1101/gr.278382.123

**Published:** 2024-03

**Authors:** Christine E. Schnitzler, E. Sally Chang, Justin Waletich, Gonzalo Quiroga-Artigas, Wai Yee Wong, Anh-Dao Nguyen, Sofia N. Barreira, Liam B. Doonan, Paul Gonzalez, Sergey Koren, James M. Gahan, Steven M. Sanders, Brian Bradshaw, Timothy Q. DuBuc, Febrimarsa, Danielle de Jong, Eric P. Nawrocki, Alexandra Larson, Samantha Klasfeld, Sebastian G. Gornik, R. Travis Moreland, Tyra G. Wolfsberg, Adam M. Phillippy, James C. Mullikin, Oleg Simakov, Paulyn Cartwright, Matthew Nicotra, Uri Frank, Andreas D. Baxevanis

**Affiliations:** 1 Whitney Laboratory for Marine Bioscience, University of Florida, St. Augustine, Florida 32080, USA;; 2 Department of Biology, University of Florida, Gainesville, Florida 32611, USA;; 3 Division of Intramural Research, National Human Genome Research Institute, National Institutes of Health, Bethesda, Maryland 20892, USA;; 4 National Center for Biotechnology Information, National Library of Medicine, National Institutes of Health, Bethesda, Maryland 20892, USA;; 5 Centre de Recherche en Biologie cellulaire de Montpellier (CRBM), Université de Montpellier, Centre National de la Recherche Scientifique, 34293 Montpellier CEDEX 05, France;; 6 Department for Neurosciences and Developmental Biology, University of Vienna, 1030 Vienna, Austria;; 7 Centre for Chromosome Biology, College of Science and Engineering, University of Galway, Galway H91 W2TY, Ireland;; 8 Department of Biochemistry, University of Oxford, Oxford OX1 3QU, United Kingdom;; 9 Department of Surgery, Thomas E. Starzl Transplantation Institute, University of Pittsburgh, Pittsburgh, Pennsylvania 15261, USA;; 10 Pittsburgh Center for Evolutionary Biology and Medicine, University of Pittsburgh, Pittsburgh, Pennsylvania 15261, USA;; 11 Department of Biology, Swarthmore College, Swarthmore, Pennsylvania 19081, USA;; 12 Pharmaceutical Biology Laboratory, Faculty of Pharmacy, Universitas Muhammadiyah Surakarta, Jawa Tengah 57169, Indonesia;; 13 Center for Organismal Studies, University of Heidelberg, 69117 Heidelberg, Germany;; 14 NIH Intramural Sequencing Center, Rockville, Maryland 20852, USA;; 15 Department of Evolution and Ecology, University of Kansas, Lawrence, Kansas 66045, USA

## Abstract

*Hydractinia* is a colonial marine hydroid that shows remarkable biological properties, including the capacity to regenerate its entire body throughout its lifetime, a process made possible by its adult migratory stem cells, known as i-cells. Here, we provide an in-depth characterization of the genomic structure and gene content of two *Hydractinia* species, *Hydractinia symbiolongicarpus* and *Hydractinia echinata*, placing them in a comparative evolutionary framework with other cnidarian genomes. We also generated and annotated a single-cell transcriptomic atlas for adult male *H. symbiolongicarpus* and identified cell-type markers for all major cell types, including key i-cell markers. Orthology analyses based on the markers revealed that *Hydractinia*’s i-cells are highly enriched in genes that are widely shared amongst animals, a striking finding given that *Hydractinia* has a higher proportion of phylum-specific genes than any of the other 41 animals in our orthology analysis. These results indicate that *Hydractinia*’s stem cells and early progenitor cells may use a toolkit shared with all animals, making it a promising model organism for future exploration of stem cell biology and regenerative medicine. The genomic and transcriptomic resources for *Hydractinia* presented here will enable further studies of their regenerative capacity, colonial morphology, and ability to distinguish self from nonself.

The increasing number of genome sequences that are now available for nonbilaterian animal species has provided a strong foundation for better understanding the molecular innovations that drove the surge of diversity seen in early animal evolution. Of particular interest are the cnidarians, a phylum composed of more than 10,000 species that include the corals, sea anemones, jellyfish, and hydroids (Steele et al. 2011; Gahan et al. 2023). The distinguishing feature that unifies all members of this phylum is that they possess a specialized type of stinging cell called a cnidocyte that is used to both ward off enemies and capture prey. From a genomic standpoint, the cnidarians occupy an informative position on the animal tree as the sister group to the bilaterians, making them a powerful model for studying numerous biological processes common to all animals. From a biomedical standpoint, they have been found to encode more orthologs to genes associated with human disease than do classic invertebrate models, supporting the proposition that they can serve as viable models for studying various classes of human diseases (Maxwell et al. 2014).

One particular cnidarian species that has already proven to be an excellent model for the study of questions regarding stem cells, regeneration, allorecognition, and coloniality is *Hydractinia*, a small colonial marine invertebrate that grows on snail shells inhabited by hermit crabs. The polyp types found within these gonochoristic colonies include feeding polyps (gastrozooids) that feed opportunistically on small plankton and share resources throughout the colony, sexual polyps (gonozooids), and defensive polyps (dactylozooids and tentaculozooids). The colonies lend themselves to experimental study as they are easily cultured on glass microscope slides (Fig. 1A). Marine hydroids, including *Hydractinia*, have fascinated biologists since the late 1800s (Weismann 1883) owing to their population of pluripotent stem cells, called “i-cells” given their localization within the interstitial spaces of its epithelial cells (Varley et al. 2023); these i-cells are responsible for *Hydractinia*’s remarkable regenerative capabilities. In fact, the term “stem cell” (*stamzellen*) was coined by August Weismann in an 1883 chapter on *Hydractinia*’s putative migratory sperm progenitors (Weismann 1883; Wessel 2013). Additional characteristics of these organisms such as allorecognition—a colony's ability to distinguish itself from conspecifics—have also received considerable attention (Nicotra 2019). Their closest well-studied relative is the freshwater *Hydra*, which shares many characteristics with *Hydractinia*, including possessing i-cells, the capacity for whole-body regeneration, and the absence of a medusa adult phase. However, *Hydractinia* differs from *Hydra* in several important respects, including its colonial morphology, polyp polymorphism, and possession of a single self-renewing stem cell lineage (Varley et al. 2023), compared with the three self-renewing lineages found in *Hydra* (interstitial, endodermal, and ectodermal). There are also salient differences in their life cycles, with *Hydractinia* undergoing metamorphosis from the larval to adult form, whereas *Hydra* shows direct development with no larval stage. These differences between the two lineages are unsurprising given that they diverged at least 500 million years ago (MYA) (Steele et al. 2011).

**Figure 1.**
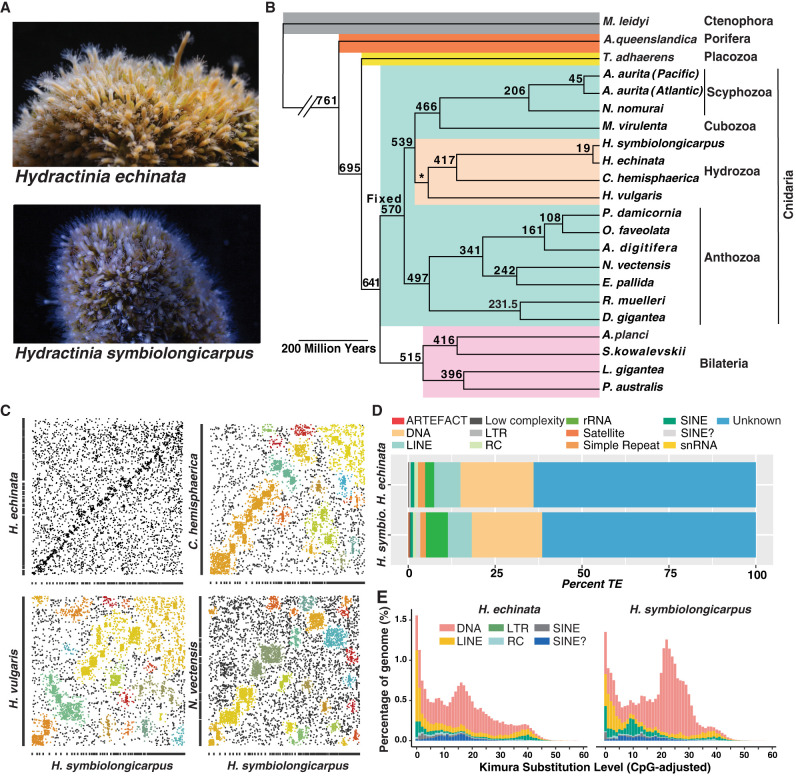
Overview of *Hydractinia*, phylogenetic analysis, synteny analysis, and analysis of repetitive elements. (*A*) *Hydractinia echinata* colony (*top*); *Hydractinia symbiolongicarpus* colony (*bottom*). (*B*) Maximum likelihood phylogeny estimated from a data set of single-copy orthologs as inferred by OrthoFinder2 showing that the two *Hydractinia* species cluster together with *Clytia hemisphaerica* and *Hydra vulgaris* branching next to them within the Hydrozoa. Divergence times were estimated using the r8s program (Sanderson 2003). The age of Cnidaria was fixed at 570 million years ago (MYA) and the age of Hydrozoa constrained to 500 MYA based upon work by Cartwright and Collins (2007). (*C*) Syntenic dot plots comparing *H. symbiolongicarpus* with four cnidarian species: *H. echinata*, *C. hemisphaerica*, *H. vulgaris*, and *Nematostella vectensis*. Colored boxes indicate linkage groups. (*D*) Stacked bar chart showing proportions of different transposable element classes in each *Hydractinia* genome using RepeatMasker de novo analysis. ARTEFACT refers to elements often found in cloning vectors that may contaminate sequencing projects. (*E*) Repeat landscape analysis showing overall a highly similar evolutionary history of invasion of repetitive elements in the two species. In *H. symbiolongicarpus*, there was a species-specific recent expansion (at ∼10% nucleotide substitution) of LTR retrotransposons.

Here, we report highly contiguous genome assemblies for two species—*H. symbiolongicarpus*, found along the east coast of the United States, and *H. echinata*, found in European waters—and compare their genome structure and content with those of other cnidarians and other animals. These whole-genome sequence data have served as the basis for performing several evolutionary analyses, including ortholog clustering based on the predicted proteomes from 49 species that encompass a wide array of animals and unicellular eukaryotes, as well as analyses aimed at deducing lineage-specific evolutionary novelties. Orthology inference analyses allowed for a thorough description of overall gene evolutionary patterns, including lineage specificity and gene family dynamics.

In addition to identifying the homeobox gene complement of *Hydractinia*, we also report the first comprehensive description of the noncoding RNA (ncRNA) landscape of any cnidarian species. Finally, we have used a single-cell transcriptomic approach to create a robust cell-type atlas for *Hydractinia symbiolongicarpus* that has allowed for the identification of several known cell types and cell states, including two clusters with distinct stem cell (“i-cell”) signatures. Our study provides evidence that, despite the level of evolutionary novelty observed within cnidarians (and particularly within the *Hydractinia* genomes themselves), i-cells express a set of evolutionary conserved genes that are found throughout the animal tree, a finding that may have broader implications for our understanding of stem cell and regenerative biology.

## Results

### Sequencing, assembly, and annotation of *Hydractinia* genomes

We estimated the genome sizes for *H. symbiolongicarpus* male wild-type strain 291-10, *Hydractinia echinata* female wild-type strain F4, the closely related hydrozoan *Podocoryna carnea* male wild-type strain PcLH01, and *Hydra vulgaris* strain 105 using propidium iodide staining of isolated nuclei followed by flow cytometric analysis (for details, see Supplemental Material; Hare and Johnston 2011). The resulting genome size estimates were 514 Mb for *H. symbiolongicarpus* and 775 Mb for *H. echinata* (Supplemental Table S1). By way of comparison, our estimate was 517 Mb for *P. carnea* and 1086 Mb for *H. vulgaris*, consistent with previous reports (Chapman et al. 2010). We then isolated high-molecular-weight DNA from adult polyps and sequenced both *Hydractinia* genomes using a combination of Pacific Biosciences (PacBio) SMRT long-read and Illumina short-read sequence data (for details, see Supplemental Material; Supplemental Table S2). These PacBio data were then used to generate primary contig assemblies using the diploid-aware assembler Canu (Supplemental Tables S3, S4; Koren et al. 2017). Canu attempts to assemble and phase contigs representing alternative haplotypes in heterozygous regions into primary and secondary assemblies via a filtering step, but this phasing can be challenging when applied to genomes that show a high level of heterozygosity. Here, we estimated overall heterozygosity to be 1.33% for *H. symbiolongicarpus* and 0.85% for *H. echinata* (Supplemental Fig. S1). In addition, Canu phasing resulted in primary assemblies that had many duplicated loci, with initial BUSCO (Simão et al. 2015) analyses indicating 42% and 29% duplicated genes in the *H. symbiolongicarpus* and *H. echinata* assemblies, respectively. To address this, we used MUMmer 3.23 (Kurtz et al. 2004) to better separate haplotypes (for details, see Supplemental Material). Following this contig filtering procedure, the presence of duplicated loci in the primary assemblies was reduced to 11% for *H. symbiolongicarpus* and 10% for *H. echinata*. These primary contig assemblies were then scaffolded with Illumina Chicago libraries through Dovetail HiRise scaffolding (Putnam et al. 2016) and gap-filled using PBJelly (English et al. 2012). The assemblies were polished using the final consensus-calling algorithm Arrow (Chin et al. 2013) and further polished with Pilon (Walker et al. 2014). The resulting final scaffolded and polished primary assemblies resulted in a 406-Mb assembly for *H. symbiolongicarpus* consisting of 4840 scaffolds with a scaffold N50 of 2236 kb, as well as a 565-Mb assembly for *H. echinata* consisting of 7767 scaffolds with a scaffold N50 of 904 kb (Supplemental Table S3). The discrepancy between the final assembly sizes and the estimated genome sizes is likely mainly owing to unresolved repetitive regions. BUSCO percentages for the final assemblies indicated a high level of completeness for both genomes (89.6% for *H. symbiolongicarpus* and 89.1% for *H. echinata*) (Supplemental Table S3). Karyotype analysis of *H. symbiolongicarpus* previously reported 15 pairs of chromosomes (2n = 30) for this species (Chen et al. 2023), consistent with the chromosome count of several other cnidarians, including *H. vulgaris, Clytia hemisphaerica*, and *Nematostella vectensis* (Zacharias et al. 2004; Putnam et al. 2007; Guo et al. 2018; Munro et al. 2023).

### Gene model prediction and annotation

Using RNA-seq reads and assembled transcripts from adult animals to guide the annotation process, we predicted genes for each genome using AUGUSTUS (Haas et al. 2008), with detailed methods provided in Supplemental Data S1 and S2 and summary statistics in Supplemental Table S3; 22,022 genes were predicted for *H. symbiolongicarpus* and 28,825 for *H. echinata*. Coding regions make up ∼8% of each assembly, whereas noncoding regions account for 92%. On average, *H. symbiolongicarpus* has 7.47 exons and 6.47 introns per gene compared with 6.60 exons and 5.60 introns per gene in *H. echinata* (Supplemental Table S5). The average intergenic region is 6679 bp for *H. symbiolongicarpus* and 7603 bp for *H. echinata* (Supplemental Table S5). 5′ and 3′ UTR predictions were performed with PASA (Haas et al. 2008), indicating that 48% (*H. symbiolongicarpus*) and 42% (*H. echinata*) of the gene models have predicted UTRs. Some *Hydractinia* transcripts undergo *trans*-spliced leader addition processing, which is known to occur in hydrozoan genomes (Stover and Steele 2001; Derelle et al. 2010). The replacement of 5′ UTR sequences by short sequences that are *trans*-spliced from noncoding spliced leader RNAs occurs in a few distantly related animal groups, as well as in several unicellular eukaryotes (Hastings 2005). We detected spliced leader sequences in our mRNA sequencing data, as well as spliced leader genes. Our ability to accurately predict 5′ UTRs for some gene models was likely impacted by this phenomenon.

We evaluated completeness of the predicted gene models via BUSCO v5 (Simão et al. 2015) with the Metazoa data set of 954 proteins. For *H. symbiolongicarpus*, there were 92.5% complete and 10.2% duplicated genes (Supplemental Tables S3, S11, tab SM1), whereas there were 90.7% complete and 12.3% duplicated genes in *H. echinata* (Supplemental Tables S3, S11, tab SM1). The number of duplicated genes may be slightly elevated owing to our gene prediction pipeline strategy (Supplemental Material). We determined the percentage of gene models that had assembled transcript support and performed functional annotation on these gene models, combining our RNA-seq data from adult animals with additional RNA-seq data from *H. symbiolongicarpus* developmental stages or *H. echinata* polyp head regeneration time points (Supplemental Material; Supplemental Code S1; Supplemental Tables S6, S7) for our transcript support analysis. Overall, 78% of *H. symbiolongicarpus* gene models and 63% of *H. echinata* gene models had transcript support with at least 90% gene overlap (Supplemental Figs. S2–S5; Supplemental Table S8). A small percentage of gene models had no overlapping transcript support (14% *H. symbiolongicarpus*, 21.5% *H. echinata*) (Supplemental Figs. S2, S4; Supplemental Table S8). Functional annotation of the gene models was performed using several approaches that included a DIAMOND search (Buchfink et al. 2015) of NCBI's nr database and using PANNZER2 (Supplemental Material; Supplemental Table S9; Törönen et al. 2018). Overall, 88.5% of *H. symbiolongicarpus* gene models and 76.2% of *H. echinata* gene models had some level of annotation: a DIAMOND hit to NCBI nr, a PANNZER2 hit, or both (Supplemental Table S9).

### Mitochondrial genome

Cnidarians are characterized by mitochondrial genomic diversity, varying in overall mtDNA conformation (circular or linear), gene content, gene organization, and the number of mitochondrial chromosomes within each species (Kayal et al. 2012, 2015; Smith et al. 2012). Medusozoan cnidarians possess linear monomeric or multimeric mitochondrial chromosomes, whereas most anthozoan cnidarians possess circular mtDNA (Supplemental Fig. S6; Bridge et al. 1992; Brugler and France 2007; Kayal et al. 2012, 2015). The typical mtDNA observed in cnidarians consists of a set of 17 genes: the small and large ribosomal genes, methionine and tryptophan transfer RNA (tRNA) genes, and 13 energy pathway proteins (Bridge et al. 1992; Beagley et al. 1998). These genes are usually organized in the same transcriptional orientation, with a partial or complete extra copy of the *Cox1* gene in the opposite transcriptional orientation at one end of the chromosome (Kayal and Lavrov 2008). Secondary structures in intergenic regions and at the ends of the mtDNA regions may be involved in the control of replication and transcription (Brugler and France 2007; Stampar et al. 2019) and are also thought to protect the ends of the mitochondrial chromosome given their lack of traditional telomeric repeats, as previously observed in *Hydra oligactis* (Beagley et al. 1998; Brugler and France 2007; Kayal et al. 2012; Smith et al. 2012). Furthermore, introns, duplicated genes, and several additional protein-coding genes have been observed in several nonhydrozoan cnidarian mitogenomes (Beagley et al. 1998; Shao et al. 2006; Chen et al. 2008; Voigt et al. 2008).

The linear mitochondrial genome of *Hydractinia* is located on a single scaffold in both *Hydractinia* species, containing the coding sequences for the large (*16S/RNL*) and small (*12S/RNS*) ribosomal subunits, mitochondrial tRNA genes, all cnidarian mitochondrial proteins (*Cox1-3*, *Cob*, *Nad1-6*, and *Nad4L*), and inverted terminal repeats (ITRs) that form G-rich loops at both ends of the molecule. This strongly suggests that *Hydractinia* contains only one mitochondrial chromosome, similar to what has been observed in other hydrozoan genomes (Supplemental Fig. S7; Supplemental Table S10; Kayal and Lavrov 2008; Kayal et al. 2012; Smith et al. 2012). *Hydractinia*’s mitochondria are mostly devoid of tRNAs, with both species containing just one tRNA-Met sequence and one tRNA-Trp sequence (Supplemental Fig. S8). These sequences form the characteristic tRNA hairpin structure and are in noncoding regions (Supplemental Fig. S8). An alternative mechanism for the replication and expression of linear mitochondrial genomes has been suggested, in which transcription and replication occur in two directions, starting from a large intergenic spacer (Kayal et al. 2015). The origin of replication (Ori) is characterized by stable stem-loop configurations containing T-rich loops and abrupt changes in DNA composition bias (Brugler and France 2007; Stampar et al. 2019). Based on these characteristics, we propose that the Ori in *Hydractinia* is in the intergenic spacer between the large ribosomal subunit (*16S/RNL*) and the *Cox2* gene (Supplemental Figs. S7, S9). The ITRs of both *Hydractinia* species can form G-rich loops that likely protect the ends of these linear mitochondrial chromosomes in the absence of telomeric sequences (Supplemental Fig. S10). In addition, the presence of nonfunctional (and gradually degrading) nuclear copies of mtDNA (NUMTs) have previously been identified in *H. vulgaris* (Song et al. 2013). Sequence similarity searches did not detect NUMTs within either *Hydractinia* genome. This result was confirmed by the lack of sequence variance in Illumina raw reads mapped to their mitochondrial genomes. Other cnidarians with linear mtDNAs, such as the jellyfish *Sanderia malayensis* and *Rhopilema esculentum*, were also shown to not contain NUMTs (Nong et al. 2020).

### Orthology inference, phylogenetic analyses, and divergence time estimates

Orthology inference analysis was performed on a splice-filtered data set (Supplemental Data S3) consisting of proteomes from 49 eukaryotic species encompassing 15 animal phyla and four nonanimal outgroups (Supplemental Material; Supplemental Table S11). Taxon selection was initially based on a data set used by Maxwell et al. (2014) to infer the evolutionary origins of human disease-associated gene families that was then expanded to place the *Hydractinia* genomes in an evolutionary context with other cnidarian genomes. To that end, 16 cnidarian species spread across the main cnidarian lineages were included. This represents the largest sampling of cnidarians in any genome-wide orthology inference study performed to date and provides increased resolution for characterizing evolutionary dynamics among cnidarians, as well as between cnidarians and other animals. An input species tree (Supplemental Fig. S11) based on the current literature was provided to OrthoFinder v2.2.7 (Emms and Kelly 2019). A total of 33,325 orthogroups containing 81.2% of the proteins were recovered in the data set. These orthogroups were then used as the basis for the analyses described below (Supplemental Data S3–S9).

For our phylogenetic analysis, we selected a subset of single-copy ortholog (SCO) sequences from our orthogroup data set (Supplemental Data S10). These SCOs were chosen for their presence in at least 12 of 15 cnidarian species; four bilaterian and three nonbilaterian outgroup species that also contained these SCOs were included in the analysis. The final concatenated, aligned, and trimmed data set included sequences from 216 orthogroups, resulting in an alignment of 50,457 nucleotides (nt) (Supplemental Data S10). The resulting maximum likelihood tree, generated using IQ-Tree2 (Supplemental Data S11, S12), confirmed known relationships within Cnidaria, including placing the two *Hydractinia* species closest to *C. hemisphaerica* (Fig. 1B). This tree was then used to estimate divergence times within the phylum using r8s (Sanderson 2003). Our age estimate for the most recent common ancestor of anthozoans is 496.6 MYA, whereas that of medusozoans is 538.9 MYA. Although the estimated ages for clades within Cnidaria tend to be older than those previously reported (Khalturin et al. 2019), we find the divergence time between the two *Hydractinia* species to be just 19.16 MYA (Fig. 1B). Providing an alternative input species tree with Porifera at the base did not significantly alter overall results of orthology inference or divergence time estimates (Supplemental Fig. S12).

### Synteny

We performed pairwise macrosynteny analyses comparing *H. symbiolongicarpus* and *H. echinata*, as well as a series of comparisons between each *Hydractinia* species and *C. hemisphaerica*, *H. vulgaris*, and *N. vectensis* by clustering scaffolds of these species based on the shared orthogroup numbers (Supplemental Material; Supplemental Code S1). Despite not having chromosomal-level assemblies, we observed local collinearity between the two *Hydractinia* species (Fig. 1C) and general chromosomal-level conservation beyond scaffold boundaries, as evidenced by scaffold clustering within the *Hydractinia* genus and beyond (Fig. 1C). This indicates a high degree of synteny between the two *Hydractinia* species, an observation that is not surprising owing to their close phylogenetic relationship and relatively recent divergence (Fig. 1B). The observation that this conservation is shared with at least three other cnidarian species (Fig. 1C; Supplemental Fig. S13) suggests that *Hydractinia* chromosomes show a similar degree of ancestrality (Simakov et al. 2022). Further chromosomal-level assembly and analysis will be required to validate this hypothesis and identify features unique to *Hydractinia*.

### Characterization of genomic repeats, including transposable elements

According to our RepeatMasker de novo analysis, genomic repeats comprise 55% of the *H. echinata* genome and 50% of the *H. symbiolongicarpus* genome. These figures are slightly lower than the percentage of repetitive DNA found in *H. vulgaris* (57%) but higher than that found in both *C. hemisphaerica* (39%) and *N. vectensis* (25%) (Supplemental Table S12; Putnam et al. 2007; Chapman et al. 2010; Leclère et al. 2019). The overall composition of repeat classes is similar between the two *Hydractinia* species (Fig. 1D; Supplemental Fig. S14; Supplemental Tables S13–S16). The largest proportion of repeats are unclassified in both genomes, accounting for ∼60% of all repetitive elements; these unclassified repeats comprise 35% and 30% of the *H. echinata* and *H. symbiolongicarpus* genomes, respectively.

Beyond the unclassified repeats, DNA transposons comprise the most abundant class of transposable elements, accounting for ∼20% of all repetitive elements and 11% of each genome. This is similar to what has been observed in both *N. vectensis* and *H. vulgaris*, in which DNA transposons are the most abundant class of transposable elements. Some differences between the two species in several DNA transposon superfamilies were noted (Supplemental Fig. S14). Long interspersed nuclear elements (LINEs) accounted for 7% of all repetitive elements and 4% of each genome. Other repetitive element classes have similar compositions in the two genomes, except for long terminal repeat (LTR) retrotransposons. Although LTR retrotransposons only accounted for a small fraction of the genome in both species, there are some significant differences in their family composition and evolution between the species (Supplemental Fig. S14). The LTR retrotransposons accounted for 2.6% of all repetitive elements in *H. echinata* and 3% in *H. symbiolongicarpus*, representing 1.5% and 3% of these genomes, respectively. We performed a repeat landscape analysis (Supplemental Material) that suggests a highly similar evolutionary history of invasion of repetitive elements in the two species (Fig. 1E; Supplemental Code S1) with differences between the species illustrated in Supplemental Figures S15 and S16. One such example is a recent species-specific expansion (at ∼10% nucleotide substitution) of LTR retrotransposons in *H. symbiolongicarpus* (Supplemental Fig. S16). This small expansion was mainly composed of members of the Gypsy family of LTRs. The two genomes also harbor different types of endogenous retroviruses (ERVs). ERV group K genes (ERVKs) are only present in *H. echinata*, whereas ERV group L genes (ERVLs) are only present in *H. symbiolongicarpus*, suggesting two recent independent invasions of ERVs after the speciation event ∼19 MYA (Supplemental Fig. S16).

### Orthogroup lineage specificity and overall patterns of evolutionary novelty

Recent cnidarian genome sequencing projects (Gold et al. 2019; Khalturin et al. 2019; Leclère et al. 2019) have shown the contribution of both taxon-restricted and shared ancestral gene families to cnidarian-specific cell types, such as those found in the medusa. To evaluate the contribution of such gene families to evolutionary novelty in *Hydractinia*, we identified lineage-specific subsets of orthogroups. Out of the 33,325 orthogroups inferred by OrthoFinder, ∼26% are cnidarian specific, 16% are medusozoan specific, 8% are hydrozoan specific, 6% are specific to *Hydractinia* + *Clytia*, and just under 5% are specific to the genus *Hydractinia*. In comparison, only 7% of orthogroups are specific to anthozoans. *H. echinata* possesses 46 species-specific orthogroups, whereas *H. symbiolongicarpus* possesses just 15 such orthogroups. Additionally, based on our sampling of 23 bilaterian species from a variety of phyla, the percentage of bilaterian-specific orthogroups (∼24%) is similar to the 26% found in cnidarians.

To evaluate the contribution of conserved gene families to *Hydractinia*’s evolution and further evaluate the broad suitability of cnidarians as animal models, we calculated the overlaps of orthogroups between major groups of cnidarians and bilaterians (Supplemental Fig. S17; Supplemental Data S13–S15). At the broadest scale, cnidarians and bilaterians possess more shared than unshared orthogroups. This supports previous observations based on the genome sequences of *Hydra* (Chapman et al. 2010) and *Nematostella* (Putnam et al. 2007) that much of the cnidarian toolkit predates the divergence of Cnidaria and Bilateria. Splitting Cnidaria further into the Medusozoa and Anthozoa (Supplemental Fig. S17A), we observe that the number of orthogroups unique to Medusozoa + Bilateria is nearly equal to that for Anthozoa + Bilateria, both of which are greater than the number for Medusozoa + Anthozoa. This is consistent with numerous observations of deep divergence between medusozoan and anthozoan genomes, from fossil estimates to divergence time estimates (Steele et al. 2011; Khalturin et al. 2019).

**Figure 2.**
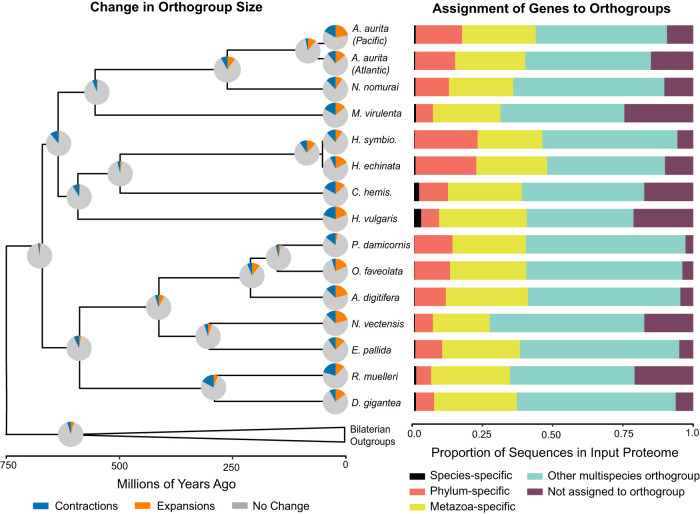
Summary of orthogroup evolution across a subset of sampled taxa. (*Left*) Changes in gene family size estimated using CAFE. Pie charts represent changes along the branch leading to a given node or tip for all 8433 orthogroups inferred to be present in the common ancestor of this tree. Branch lengths are as depicted in Figure 1B. (*Right*) Proportion of input proteome sequences assigned by OrthoFinder to different orthogroup categories. For results for every species included in the OrthoFinder analysis, see Supplemental Figure S16; for the number of input sequences in each proteome, see Supplemental Table S12. The data used to create these figures can be found in Supplemental Table S12. *Aurelia aurita* Pacific genome from Gold et al. (2019); Baltic/Atlantic genome from Khalturin et al. (2019).

To further investigate potential sources of evolutionary novelty, we calculated the percentage of genes within each species that is assigned to orthogroups that are species specific, the percentage of phylum-specific and metazoan-specific genes, the percentage belonging to other multispecies orthogroups, and the percentage of genes not assigned to an orthogroup. These five proportions are visualized in the right panel of Figure 2 for the 15 cnidarian species that were analyzed further using CAFE (see below). Proportions for all metazoan species in our analysis are visualized in Supplemental Figure S18. The two *Hydractinia* species contain the highest percentage of phylum-specific genes of all of the 43 metazoan species we examined (23% and 22%, respectively), thereby indicating that their genomes contain the highest percentage of cnidarian-specific genes of all cnidarians included in this analysis. Coupled with the fact they possess relatively few species-specific orthogroups, this suggests that a significant proportion of their proteomes may have evolved at the genus, family, or subphylum level, which are grouped together under “phylum-specific” in the analysis featured in Figure 2. Additionally, a DIAMOND search indicated that most (90%) unassigned *Hydractinia* genes had no match in the NCBI nr database (Supplemental Table S11). Transcript support for these genes (Supplemental Table S8) indicates that a large proportion of these genes have >90% transcript overlap (51.28% in *H. symbiolongicarpus* and 35.35% in *H. echinata*) and are expressed by the animal. Thus, the two *Hydractinia* genomes appear to contain an abundance of evolutionarily novel genes.

### Estimating the evolutionary dynamics of gene families using CAFE

Focusing just on the Cnidaria + Bilateria subtree (19 species) derived from the 22-species tree inferred using IQ-Tree2 and r8s (described above), we estimated the evolutionary dynamics of the 8433 OrthoFinder-inferred orthogroups that are present in the ancestor of this subtree (Supplemental Material; Supplemental Data S16–S27). Using CAFE, gene family dynamics were estimated for each node and terminal taxon (De Bie et al. 2006; Han et al. 2013) in our subtree and are summarized in Figure 2 (left panel), with additional details available in Supplemental Table S11 (tab X.8).

Across the whole tree (Fig. 2), more changes in gene family size take place on the terminal branches of the tree than in the internal branches of the tree. Terminal branches have significantly more gene expansion or contraction compared with internal branches (mean[terminal] = 2375.7, mean[internal] = 1007, t = 8.5139, df = 33.99, *P*-value = 6.07 × 10^−10^). This pattern is very clear when comparing the internal nodes of the cnidarian phylum with the terminal branches of this group (Fig. 2). Of the 8433 analyzed orthogroups, a total of 592 were found to be evolving rapidly on the subtree (Viterbi *P*-value ≤ 0.05). The distribution of these uniquely fast-evolving gene families per taxon/node can be found in Supplemental Table S11 (tab X.8), and information about their putative identities can be found in the Supplemental Material.

### Comparing evolutionary dynamics of *H. symbiolongicarpus* and *H. echinata* using CAFE

Roughly half of the orthogroups present in the *Hydractinia* genomes and included in the CAFE analysis have undergone some change in size (50% in *H. symbiolongicarpus* and 54% in *H. echinata*) when comparing their observed size to the inferred size of these orthogroups in the Cnidarian + Bilaterian ancestor. Notably, the two *Hydractinia* genomes have very different proportions of gains versus losses over their terminal branches. *H. echinata* has experienced more expansions with a higher number of genes per expansion, resulting in *H. echinata* gaining about twice as many (1.97×) individual gene copies in the past 19 million years. Conversely, *H. symbiolongicarpus* has a higher number of contracted gene families and has lost more genes per contraction, meaning that *H. symbiolongicarpus* has lost nearly 2.5 times more genes in total than *H. echinata* has since their divergence. Additionally, although *H. echinata* and *H. symbiolongicarpus* have lost 248 and 252 gene families, respectively, the identities of the lost families do not overlap at all. This implies that these species have undergone very different evolutionary trajectories since their divergence ∼19 MYA. We performed additional comparisons of evolutionary dynamics in *Hydractinia* versus the other hydrozoan taxa (*H. vulgaris* and *C. hemisphaerica*) and versus the genus *Aurelia* (Supplemental Material). Overall, *H. vulgaris* and *C. hemisphaerica* have more taxon-specific orthogroup size changes than either species of *Hydractinia*. However, when combining data from the two *Hydractinia* species to look at changes at the genus level, the number of changes is roughly similar between these hydrozoans. For the comparison with *Aurelia*, we found that the overall proportions of gains versus losses was much more similar between the two *Aurelia* lineages, in contrast with what we found for the two *Hydractinia* species (Supplemental Material; Supplemental Fig. S19).

### The ncRNA landscape: miRNAs

microRNAs (miRNAs) constitute a unique class of small ncRNAs of ∼22 nt in size that play crucial roles in development, cellular differentiation, and stress response in both plants and animals (Wheeler et al. 2009). Several studies have investigated miRNAs and the miRNA pathway in cnidarians (Moran et al. 2013; Praher et al. 2021). We generated small RNA-seq libraries for five samples of adult *H. echinata* polyps that were then sequenced (Supplemental Material). The resulting reads were trimmed and mapped to the *H. echinata* genome using the miRDeep2 mapping algorithm (Friedländer et al. 2012), yielding 347 predicted miRNAs. Subsequent custom automated filtering and manual screening of this set of miRNAs was performed to identify the highest-quality predicted miRNAs from this set, producing a final list of 38 unique high-quality mature miRNA sequences (Supplemental Figs. S20, S21; Supplemental Table S17). Of these, three are homologous to known cnidarian miRNAs (miR-2022, miR-2025, and miR-2030), with alignments shown in Supplemental Figure S22. Supplemental Figure S23 depicts a proposed evolutionary scenario for miRNAs in cnidarians that includes these new data from *H. echinata*.

### The ncRNA landscape: rRNAs, tRNAs, and snoRNAs

In an attempt to provide the first detailed description of the ncRNA landscape of any cnidarian species, we found that the two *Hydractinia* genomes encode the expected suite of functional ncRNAs commonly present in metazoan genomes. These included ribosomal RNA (rRNA) genes, tRNAs for each amino acid isotype, spliceosomal RNAs for both the major (U1, U2, U4, U5, and U6) and minor spliceosome (U11, U12, U4atac, and U6atac), small nucleolar RNAs (snoRNAs), SRP RNA, RNase P RNA, RNase MRP RNA, and Vault RNA (Supplemental Table S18). This characterization was based on results from Rfam (Kalvari et al. 2018), Infernal (Nawrocki and Eddy 2013), and tRNAscan-SE (Supplemental Material; Chan et al. 2021). An unusual feature of many of these ncRNAs is their apparent organization into roughly evenly spaced tandem arrays of tens or even hundreds of nearly identical or highly similar copies. Each of these copies is separated by spacer regions ranging in length from several hundred to a few thousand nucleotides that are nearly identical or highly similar to one another (Supplemental Tables S19–S21; Supplemental Data S28–S36). In both *Hydractinia* genomes, these arrays include rRNAs, four of the five RNA components of the major spliceosome (U1, U2, U5, and U6), the snoRNA U3, and tRNAs for each of the 20 amino acids (Supplemental Tables S18, S21–S25). Although tandem arrays of some RNA genes—especially clusters of rRNA genes collectively known as rDNA—are common in eukaryotes (Long and Dawid 1980; Cloix et al. 2000), tandem array organization of tRNAs (Bermudez-Santana et al. 2010) is unusual outside of the *Entamoeba* genus of *Amoebozoa* (Tawari et al. 2008), with only one such example having been observed in mammals (Darrow and Chadwick 2014). The ncRNA tandem arrays only make up a small percentage of all regions that appear in tandem repeats in the *Hydractinia* assemblies. Tandem repeat regions detected using TRF (Benson 1999) having seven or more copies with a period length of 50 nt and ≥75% average similarity between repeats cover 18.7% of the *H. echinata* and 15.7% of the *H. symbiolongicarpus* assemblies. These TRF-defined repeats are largely a subset of the unclassified repeats identified by our RepeatMasker analysis detailed above (88.1% of the *H. echinata* and 72.0% of the *H. symbiolongicarpus* nucleotides in the TRF-defined repeat regions also exist in the unclassified repeat regions). The nucleotides covered by the RNA tandem arrays account for only 4.8% and 7.7% of these TRF-defined repetitive regions in *H. echinata* and *H. symbiolongicarpus*, respectively. Although the biological significance of these ncRNA tandem arrays and other tandem repeat regions remains unclear in the absence of functional data, two important observations argue against the presence of these RNA tandem arrays being caused by sequencing or assembly artifacts. First, when comparing these results to other cnidarian species, we were able to identify tandem arrays of 5S rRNA, tRNA, and U5 RNA in the *N. vectensis* genome (Putnam et al. 2007) but did not find RNA tandem arrays in other cnidarian genomes. Second, the draft genome assembly of *H. echinata*, sequenced and assembled using different methods (Török et al. 2016) than the primary *H. echinata* assembly presented here, also includes tandem arrays of 5S rRNA, SRP RNA, and tRNA, and a significant fraction of that assembly is also in TRF-defined tandem repeats (5.1% of the genome).

### The homeobox gene complement of *Hydractinia*

Homeobox genes are a large superfamily of protein-coding genes that encode for a 60-amino-acid helix-turn-helix domain called the homeodomain (Holland 2013). Most homeobox genes are DNA-binding transcription factors (Holland 2013) that play key roles in early embryogenesis (Driever and Nüsslein-Volhard 1988), patterning (Pearson et al. 2005), development of the nervous system and sensory organs (Schulte and Frank 2014), and maintenance of embryonic stem cells (Young 2011). We identified 71 homeodomain-containing genes in the *H. symbiolongicarpus* genome and 82 in the *H. echinata* genome. Phylogenetic (Supplemental Figs. S24, S25; Supplemental Data S37–S42) and secondary domain architecture-based approaches were able to resolve the ANTP, CERS, LIM, POU, PRD, SINE, and TALE homeobox classes, with a small number of genes remaining unclassified (Supplemental Table S26). In both species, the ANTP-class homeodomains were the most abundant. Overall, *H. echinata* has 11 more homeobox genes than does *H. symbiolongicarpus*, with expansions in the CERS, LIM, POU, and PRD classes (Supplemental Table S26). Four unclassified homeobox genes are unique to *H. echinata*. It is possible that some of these expansions in *H. echinata* may be duplicates from different alleles of the same gene that were not properly phased during the separation of haplotypes during the assembly process. All seven unclassified genes in *H. symbiolongicarpus* have a homolog to an unclassified gene in *H. echinata* (Supplemental Table S27). Class-based annotation of homeodomain-containing genes based on phylogenetics, secondary domain information, and associated results from OrthoFinder for both *Hydractinia* species can be found in Supplemental Table S27.

### The HOX-L subclass of homeodomains in *Hydractinia*

Some of the most interesting genes to evolutionary biologists are those belonging to the Hox families of homeobox genes (Procino 2016). Hox genes are members of the ANTP class of homeoboxes, along with the Hox-like (“extended Hox”) genes *Eve*, *Meox/Mox*, *Mnx*, and *Gbx*; the ParaHox cluster of *Gsx*, *Cdx*, and *Pdx/Xlox*; and the NK-like gene subclass (Holland et al. 2007; Holland 2013). The ANTP class is the largest and most diverse class, consisting of more than 50 families; 37 of these families containing more than 100 genes have been identified in humans (Holland et al. 2007). Hox and ParaHox genes are thought to have emerged before animal evolution and were subsequently lost, reduced, or absent in early-emerging taxa (Mendivil Ramos et al. 2012; Steinworth et al. 2023). In many bilaterians, Hox genes are arranged in at least one chromosomal cluster (Duboule 2007). Genomic linkage between Hox genes is present in extant cnidarians, although linked Hox and ParaHox genes were not found in previous cnidarian genome studies (Putnam et al. 2007; Chapman et al. 2010; DuBuc et al. 2012; Gold et al. 2019; Jeon et al. 2019; Khalturin et al. 2019; Leclère et al. 2019).

**Figure 3.**
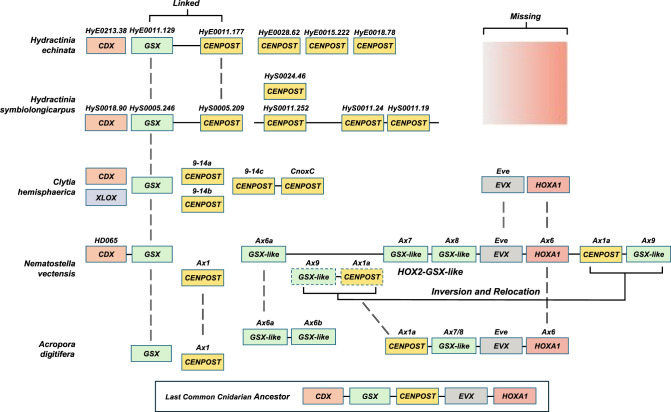
Genomic organization of Hox and ParaHox genes in five cnidarian genomes. Solid lines sharing homeobox genes represent genomic scaffolds. The scaffold and gene ID numbering in *Hydractinia* genomes are shown *above* gene boxes. Broken lines depict homologous cnidarian-specific Hox genes. Alternative gene names are shown *above* gene boxes for *C. hemisphaerica*, *N. vectensis*, and *Acropora digitifera*.

Both *Hydractinia* species possess several genes that belong to the HOX-L subclass (Supplemental Figs. S26, S27; Supplemental Data S43–S48). These include several nonanterior (CenPost) cnidarian Hox genes, the ParaHox genes *Gsx* and *Cdx*, and the Hox-extended group *Mox*. *HoxA1* and *Hox2/Gsx-like* genes are absent in both species even though these genes have been found in other cnidarians, including hydrozoans (Ryan et al. 2006; Chiori et al. 2009). Additional members of the HOX-L repertoire that are present in other cnidarians but are absent in *Hydractinia* are genes encoding for the Hox-extended gene *Eve* and the ParaHox genes *Pdx/Xlox* (Fig. 3; Ryan et al. 2006; Gold et al. 2019; Leclère et al. 2019). A primitive Hox cluster has been observed in anthozoan cnidarians but has not been found in hydrozoans (Chourrout et al. 2006; DuBuc et al. 2012). However, there appears to be some linkage of Hox genes in both *Hydractinia* genomes (Fig. 3). This includes linkage of several cnidarian-specific Hox genes in *H. symbiolongicarpus* and linkage of a cnidarian Hox gene with the ParaHox gene *Gsx* in both *Hydractinia* species. Before this study, the linkage of a Hox and ParaHox gene had not been shown in any other cnidarian genome. A comparison of phylogenetic relatedness and synteny analysis of various cnidarian species suggests that *Hydractinia* species likely lost the HOX-L genes *HoxA1* and *Eve* (Fig. 3). These genes are clustered together in anthozoans (DuBuc et al. 2012; Zimmermann et al. 2023), and *Eve* is found in close proximity to human Hox clusters (D'Esposito et al. 1991; Faiella et al. 1991). In contrast, *Hydra* has retained a *HoxA1* homolog but has also lost *Eve* (Chapman et al. 2010).

To determine the spatial patterning role of some of the homeobox genes relative to other known expression patterns, we performed colorimetric RNA in situ hybridization. Expression patterns for a subset of Hox genes at different stages of the *Hydractinia*’s life cycle were determined (Supplemental Fig. S28). Overall, several genes show a somatic patterning role during early larval formation, whereas other Hox genes are maternally expressed during sexual development. This suggests that Hox genes may have an important role in egg formation.

### The allorecognition complex

Allorecognition is controlled by at least two linked genes, *Allorecognition 1* (*Alr1*) and *Allorecognition 2* (*Alr2*), in *Hydractinia* (Nicotra et al. 2009; Rosa et al. 2010; Nicotra 2019). Both encode single-pass transmembrane proteins with highly polymorphic extracellular domains, with the allorecognition response being controlled by whether colonies share alleles at these loci. In previous work, we examined the partially assembled genome of a strain of *H. symbiolongicarpus* that is homozygous at *Alr1* and *Alr2* and discovered that both genes are part of a family of immunoglobulin superfamily genes that reside in a genomic interval called the allorecognition complex (ARC) (Huene et al. 2022). We identified *Alr1* and *Alr2* on separate scaffolds within the *H. symbiolongicarpus* reference genome, as well as a second *Alr1* allele on a third scaffold. These alleles were likely retained in the final assembly because they were sufficiently divergent from each other not to be recognized as alleles of the same gene. We identified 19 additional genes predicted to encode full-length Alr proteins similar to those previously described (Huene et al. 2022), as well as 44 gene models with some sequence similarity to Alr1 or Alr2 that were not predicted to encode cell surface proteins, suggesting they were pseudogenes. Within the reference genome, most of these Alr1/Alr2-like gene models are located in four clusters (Supplemental Fig. S29). Additional work will be required to phase these contigs into two ARC haplotypes and assign orthology between them and the Alr genes already identified (Huene et al. 2022). The Huene et al. (2022) study found that there are at least 41 *Alr­*-like loci in this region, with more than half of these genes located within one of three Alr clusters. Although the individual Alr proteins encoded by these genes have low overall sequence identity, the domain architecture of these proteins, along with structure-based predictions using AlphaFold, confirms that these Alr proteins are members of the immunoglobulin superfamily (Huene et al. 2022).

### Single-cell transcriptomics of adult animals

**Figure 4.**
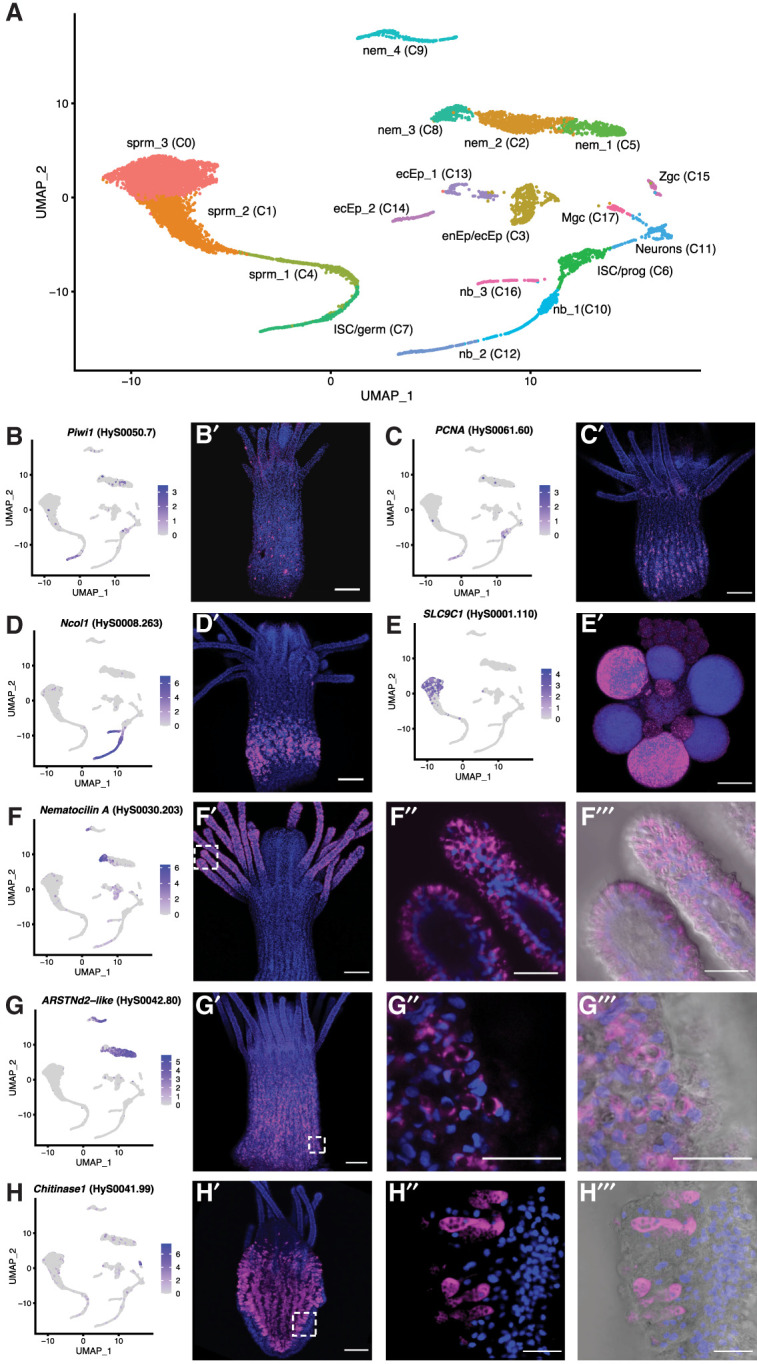
*Hydractinia* single-cell atlas represented as a labeled UMAP and validation of several cell-type markers using fluorescent in situ hybridization (FISH). (*A*) *Hydractinia* single-cell atlas UMAP with 18 clusters (C0–C17). (*B*–*F*) UMAP expression of select marker genes (*left*) and spatial expression pattern of marker gene in polyps via FISH (*right*). Blue staining indicates Hoechst; pink, marker gene. *Piwi1* (*B*) and *PCNA* (*C*) expression in the i-cell band in the middle of the body column of a feeding polyp. (*D*) *Ncol1* expression in nematoblasts in the lower body column of a feeding polyp. (*E*) *SLC9C1* expression in mature sperm cells in gonads of male sexual polyps. (*F*) *Nematocilin A* expression in a subset of nematocytes in the tentacles of a feeding polyp. Close-up view of tentacles in panels *F*′′ and *F*′′′ both show higher magnification images from the same polyp as in panel *F*′, showing expression is specific to cnidocytes. Panel *F*′′′ adds DIC. (*G*) *ARSTNd2-like* expression in a subset of nematocytes in the body column of a feeding polyp. Panels *G*′′ and *G*′′′ both show higher magnification images from the same polyp as in panel *G*′, showing expression is specific to cnidocytes. Panel *G*′′′ adds DIC. (*H*) *Chitinase1* expression in gland cells in the endodermal epithelial cell layer of a feeding polyp. Panel *H*′′ and *H*′′′ both show higher magnification images from the same polyp as in panel *H*′, showing expression is specific to gland cells. Panel *H*′′′ adds DIC. All images shown were projected from confocal stacks. All scale bars = 100 µm. Abbreviations in *A*: (ecEP) ectodermal epithelial cell, (enEP) endodermal epithelial cell, (germ) germ cell, (ISC) interstitial stem cell, (Mgc) mucous gland cell, (nb) nematoblast, (nem) nematocyte, (prog) progenitor, (sprm) sperm, and (Zgc) zymogen gland cell.

A critical part of establishing *Hydractinia* as a useful research organism is having a list of cell type–specific markers for all cell types in the adult animal. Single-cell transcriptome analysis of adult *H. symbiolongicarpus* 291-10 male animals was performed using the 10x Genomics platform (Supplemental Material). Briefly, cell suspensions of dissociated adult feeding and sexual polyps and associated connective mat tissue were prepared, and two samples were resuspended in different final buffers (3×PBS or calcium- and magnesium-free seawater minus EGTA) followed by subsequent 10x single-cell library construction. These two libraries were then sequenced using the Illumina NovaSeq 6000 sequencing system. Statistics from each library can be found in Supplemental Table S28. The two libraries were ultimately combined after analyzing them separately (Supplemental Fig. S30) and determining that they were very similar. Downstream analyses of these sequence data were performed with both the 10x Cell Ranger pipeline version 7.0.1 and Seurat version 4.3.0 (Satija et al. 2015), ultimately yielding heatmaps and UMAP plots for the visualization of cell clusters (Supplemental Material; Supplemental Data S49). The final clustering after filtering of technical artifacts (primarily removing sperm captured with another cell, termed “sperm doublets”) (see Supplemental Material; Supplemental Data S49) with Seurat resulted in 18 clusters from a total of 8888 cells (Fig. 4A). A heatmap was generated to show top variable “marker” genes for each cluster (Supplemental Fig. S31).

Each cluster was then classified as a putative cell type or cell state through the annotation of these marker genes; these included distinct clusters of ectodermal (epidermal) and endodermal (gastrodermal) epithelial cells, mucous and zymogen gland cells, neurons, nematoblasts, nematocytes, germ cells, developing stages of sperm, and two clusters of i-cells (Fig. 4A). These i-cell clusters probably include early progenitor cells as pluripotent i-cells are a rare population (DuBuc et al. 2020; Chrysostomou et al. 2022; Varley et al. 2023); thus, we have labeled them as ISC/prog on our UMAP. UMAP expression patterns for individual genes that were used to identify and annotate the clusters based on previous literature can be found in Supplemental Figure S32, and further details are provided in Supplemental Table S29. We grouped these clusters into seven major cell “types”: sperm and spermatocytes (clusters C0, C1, and C4), nematocytes (C2, C5, C8, and C9), epithelial cells (C3, C13, and C14), i-cells/germ cells (C6 and C7), nematoblasts (C10, C12, and C16), neurons (C11), and gland cells (C15 and C17).

A subset of seven different cell-type marker genes were chosen for fluorescence in situ hybridization (FISH) for validation and for visualization of the spatial expression patterns of various cell types in adult polyps (Fig. 4B–H), including two genes that have been previously published for *Hydractinia* (*Piwi1* for marking i-cells/progenitors and *Ncol1* for marking all stages of maturing nematoblasts) (Bradshaw et al. 2015). The five remaining genes can be considered new cell-type markers for *Hydractinia*. We observed that the proliferating cell nuclear antigen *PCNA*, a known proliferation and broad stem cell marker in other animals (Wagner et al. 2011), marks cells present in the i-cell band; *SLC9C1*, a member of the sodium-hydrogen exchanger (NHE) family required for male fertility and sperm motility (Wang et al. 2003), marks mature sperm in gonads of male sexual polyps; *Nematocilin A*, a known structural component of the cnidocil mechanosensory cilium trigger of mature cnidocytes in *Hydra* (Hwang et al. 2008), marks mature cnidocytes in tentacles; *ARSTNd2-like* (previously undescribed) marks cnidocytes in the polyp body column; and *Chitinase1*, a gland/secretory cell marker in cnidarians (Klug et al. 1984; Sebé-Pedrós et al. 2018), marks endodermal gland cells. These results represent a significant step toward defining the major cell types in *Hydractinia* and the gene expression patterns that define them. A list of all cluster marker genes according to cell type from the Seurat analysis can be found in Supplemental Table S30.

**Figure 5.**
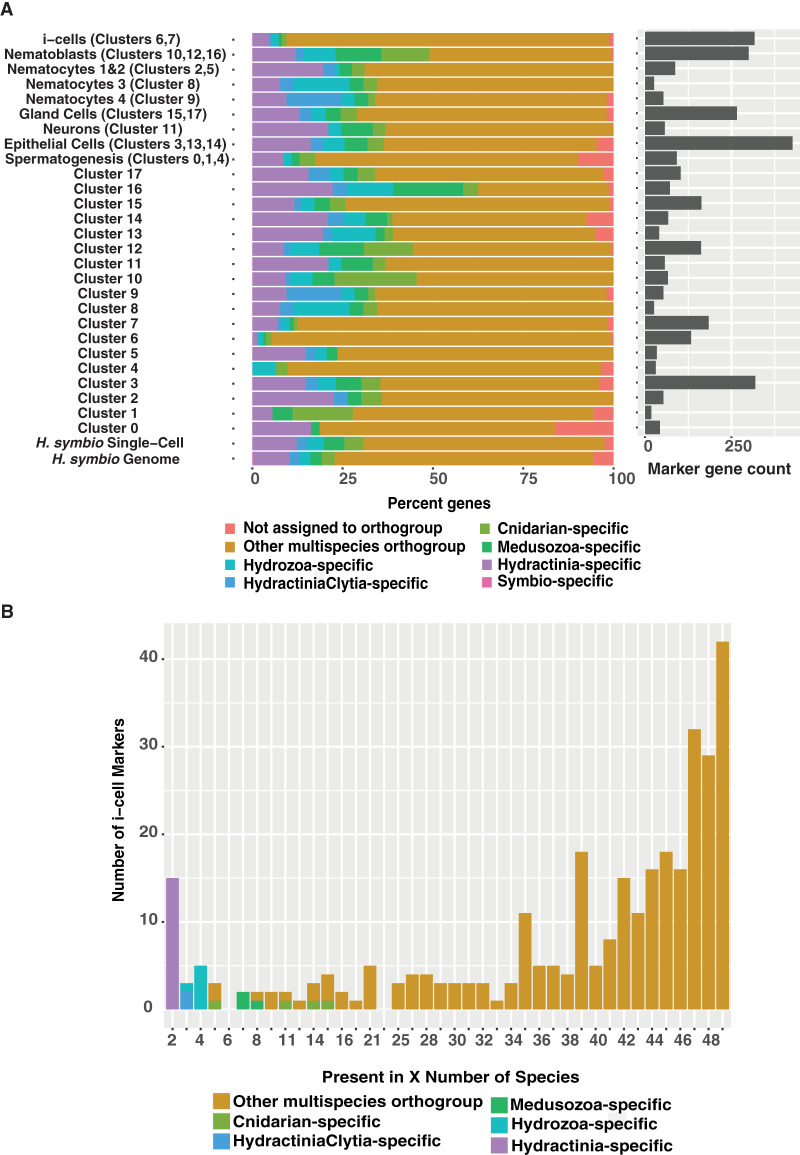
Results from the lineage-specificity analysis using OrthoFinder results and the UMAP cluster marker genes. (*A*) Stacked bar chart showing the percentage of *H. symbiolongicarpus* single-cell atlas cluster markers shared among animal phyla. The *bottom* legend shows eight different categories, dividing the markers into different groups depending on how the orthologs are shared among the species. The “not assigned to orthogroup” category represents markers that could not be placed into an orthogroup. The other categories are markers that have at least one homolog between *H. symbiolongicarpus* and that category, except for the “symbio-specific” category, which represents markers that fell into orthogroups containing only *H. symbiolongicarpus* genes. For example, hypothetical marker gene A from *H. symbiolongicarpus* would be an “other multispecies orthogroup” marker if it was found in *H. symbiolongicarpus* and at least one animal outside of cnidaria, but it would be a “Cnidarian-specific” marker if it was found in *H. symbiolongicarpus* and at least one cnidarian outside the Medusozoa. Stacked bars represent the seven major cell types split into nine groups, followed by all individual clusters and, finally, the total genes expressed in the *Hydractinia* single-cell data set (16,069 genes) and total genes predicted from the *Hydractinia* genome (22,022 genes). The marker gene count bars on the *right* indicate how many markers are present in each major cell type and cluster. (*B*) Histogram dividing the 317 orthogroup-assigned i-cell (clusters C6 and C7) markers by how many are shared by a given number of species. Legend is the same as for panel *A*, but the following categories are excluded from this chart: unassigned genes (two genes) and *H. symbiolongicarpus*-specific genes (none).

We then explored the evolutionary profile of marker genes from the 18 individual clusters and the seven cell types (split further into nine groups) (Fig. 5A) using strict filtering criteria (Supplemental Material). We found that, compared with other cell types (and clusters), i-cells and progenitors (ISC/prog cluster C6, 5.3% lineage-specific; ISC/germ cluster C7, 12.5% lineage-specific; all i-cells and progenitors, 9.5% lineage-specific) and early spermatogonia (cluster C4, 9.7% lineage-specific) are defined primarily by genes that are shared with other animals rather than lineage-specific genes, providing evidence that the toolkit used by these cell types has a shared ancestry with other animals (Fig. 5A). Nematoblasts and nematocytes—cell types that are specific to cnidarians—were marked by a high proportion of phylum-specific or within-phylum genes (nematoblasts 49%, nematocytes 32.5%). Further probing into the i-cell cluster profile (clusters C6 and C7) to analyze how widespread the i-cell/progenitor marker genes were among animals in our data set, we plotted how many species in our orthology-inference analysis shared each i-cell marker gene and found that the vast majority of the genes that mark i-cells are present in 40 or more species (Fig. 5B). Overall, our finding that the 317 i-cell marker genes were widely shared among all animals stands in contrast to the fact that the *H. symbiolongicarpus* genome has a higher proportion of phylum-specific and within-Cnidaria-specific genes (23%) than any of the other 41 animals in our orthology-inference analysis. The *Hydractinia* single-cell data set has an even higher proportion of phylum-specific and within-Cnidaria-specific genes (30.8%).

## Discussion

The extensive analyses performed in the course of this study have served to place the *Hydractinia* genome into a wider evolutionary context. In addition to providing an in-depth characterization of its nuclear genome, we determined that *Hydractinia* contains a single mitochondrial chromosome. This is similar to what has been observed in other cnidarian species but differs from what has been observed in *Hydra*, which contains two mitochondrial chromosomes. Another significant difference observed between the genomes of these two species is that, although they are present in *Hydra*, we find no evidence for the presence of NUMTs in *Hydractinia*. A possible scenario giving rise to this difference may lie in the mechanism that severed the *Hydra* mitochondrial genome in two (for the break region, see Supplemental Fig. S7), enabling the introduction of mitochondrial sequences into the nuclear genome.

Our orthology analyses, which were based on both the predicted proteomes of the two *Hydractinia* species as well as the proteomes from 41 additional animal species and six related eukaryotes, provided a strong foundation for the subsequent analyses described here. The phylogenic analyses, which were based on conserved single-copy genes from species for which high-quality genomes are available, agreed with previous placements of *Hydractinia* within the hydrozoan cnidarians, positioning *Hydractinia* together with the hydrozoans *C. hemisphaerica* and *H. vulgaris*. Although there are many additional hydrozoan taxa that have been placed between *Hydractinia* and *Clytia* based on various criteria, these species were not included in the present study owing to a lack of available whole-genome sequence data. A sister taxon to *Hydractinia* is *Podocoryna*, whose genome is currently being sequenced; availability of these new genomic data will ultimately allow for more informative comparisons between these closely related groups. Comparing the two *Hydractinia* species to one another, divergence time analyses yielded an estimate that the two species diverged ∼19 MYA. This estimate is much shorter than the estimated divergence times between lineages of the moon jelly *Aurelia aurita* (45.35 MYA in our study; 51–193 MYA reported by Khalturin et al. 2019) and is more comparable to the divergence time between lineages of *H. vulgaris* (10–16 MYA) (Wong et al. 2019).

Gene synteny analyses between the two *Hydractinia* species indicate a high degree of synteny, which also extended to at least three other cnidarian species. We anticipate that macrosynteny analysis will only improve in the future with the increased availability of chromosomal-level cnidarian genome assemblies (Kon-Nanjo et al. 2023; Zimmermann et al. 2023). The repeat content analyses presented here indicate that at least 50% of each *Hydractinia* genome is composed of repeats. Further, the overall repeat landscape was similar in the two species, with DNA transposons comprising the most abundant class of transposable elements, a finding similar to what has been observed in other cnidarian genomes (Putnam et al. 2007; Chapman et al. 2010).

Our orthology analyses indicate that 26% of the inferred orthogroups were cnidarian specific compared with the 24% of bilaterian-specific orthogroups from all sampled bilaterian species. This observation strongly suggests that the evolutionary novelty of orthogroups found across *all* of the Bilateria is equal to that found just within Cnidaria itself. Additional analyses focused on gene lineage specificity indicated that the two *Hydractinia* genomes possess the highest number of cnidarian-specific genes (22%–23%) compared with the other 15 cnidarian genomes that were included in the analysis. In addition, the vast majority of *Hydractinia* genes that did not ultimately cluster into any orthogroup also had no matches in GenBank, indicating that *Hydractinia* genomes contain a significant proportion of evolutionarily novel genes, positioning these genomes well for subsequent studies of both novel and conserved genes. Although these findings are obviously focused on evolution from a sequence-based perspective, future studies based on protein structure predictions and subsequent structure-based comparisons similar to those previously described for the freshwater sponge *Spongilla* (Ruperti et al. 2023) could further inform the degree of gene novelty within the *Hydractinia* genomes described here.

An evolutionary feature characterized in the course of this work involves the ncRNA landscape of *Hydractinia*, the first such analysis in any cnidarian species. We were able to identify all of the functional ncRNAs that are also present in other animal genomes; these ncRNAs are organized into a large number of nearly identical or highly similar tandem arrays. Further, we could identify tandem arrays of 5S rRNA, tRNA, and U5 RNA in the *Nematostella* genome but not in any other published cnidarian genome, opening up an avenue for further study as more highly contiguous genome sequence data become available.

Given the importance and high degree of evolutionary conservation of homeodomain proteins, we have deduced the presence and absence of homeodomain-containing genes in the two *Hydractinia* species, using a phylogenetic approach to resolve the ANTP, CERS, LIM, POU, PRD, SINE, and TALE homeobox classes. Our analyses have provided evidence for the linkage of several cnidarian-specific Hox genes in *H. symbiolongicarpus* and linkage of a cnidarian Hox gene with the ParaHox gene *Gsx* in both *Hydractinia* species. This has not been observed in any other cnidarian genome to date, providing evidence for the first time that bilaterian-like ParaHox genes may have once been located near the central/posterior region of the Hox cluster (Fig. 3). Further, this suggests that the last common ancestor of the cnidarians presumably had a linked Hox/ParaHox cluster flanked by NK-class and other homeobox genes (Fig. 3; D'Esposito et al. 1991). This finding could highlight that the breaking apart of the Hox and ParaHox cluster that occurred in the bilaterian ancestor may have been instrumental for their evolution.

Our characterization and analyses of *Hydractinia*’s ARC reinforce previous findings that Alr genes (and pseudogenes) are organized into a few discrete clusters covering a single large genomic region (Huene et al. 2022). The recent availability of chromosome-length genome sequence for *Hydractinia* (Kon-Nanjo et al. 2023), coupled with the highly annotated data presented in this paper and methodological advances on the protein structure prediction front, is forming the foundation for future studies focused on the conservation of synteny within this gene complex across cnidarian species, studies that will, in turn, advance our understanding of the evolution of the immune system in bilaterians.

Through the use of single-cell transcriptomic approaches, we have created a robust cell-type atlas with well-annotated clusters, which, in turn, has allowed us to identify specific genes that define individual cell types in adult animals. We identified two clusters with i-cell signatures that we designated “i-cells/prog,” which are cells heading toward a somatic fate, and “i-cells/germ,” which are heading toward a germline fate. We observed some continuity between somatic i-cells and nematoblasts, as well as between somatic i-cells and neurons. We also found continuity between germline i-cells and cells involved in spermatogenesis in *Hydractinia*. This continuity was also observed in single-cell data from other hydrozoans such as *Hydra* (Siebert et al. 2019), in which cells are continuously replaced, and, to a lesser extent, in *Clytia* (Chari et al. 2021). We were not able to capture other cell state transitions (e.g., somatic i-cells to gland cells, or somatic i-cells to epithelial cells), as those clusters were isolated in the atlas. This likely reflects both the technical limitations of our sampling (8888 cells in our atlas) and the biology of *Hydractinia*; turnover of these cell types is likely relatively low in adults compared with *Hydra*, which has constant cell turnover in adult animals (Siebert et al. 2019). Further exploration of our cell-type marker lists revealed that i-cells and progenitors are defined by genes that are highly conserved among animals, in contrast to most other cell types that contain a significant proportion of cnidarian-specific genes. This finding strongly suggests that there is a shared ancestry with other animals in the form of a conserved toolkit for regeneration. Although it remains to be seen whether other animals do indeed share the same or partially overlapping toolkits of genes specifically within their stem cells (an important question that is beginning to be addressed using new methodologies currently under development) (Wang et al. 2021), the results of the current study hold promise for future exploration from an evolutionary standpoint and, through a longer lens, potentially from a biomedical standpoint as well.

## Methods

### Genome sequencing and assembly

Genomic DNA was prepared from adult polyps from a single strain for each species (291-10 males for *H. symbiolongicarpus* and F4 females from *H. echinata*). PacBio long-read and Illumina short-read data were generated. Canu was used as the contig assembler. Scaffolding was performed by Dovetail HiRise scaffolding with Illumina Chicago libraries.

### Gene model prediction and annotation

Gene models were generated with a pipeline that involved both PASA and AUGUSTUS. Strand-specific RNA-seq data from each species were used as input at different points of the pipeline as reads and as assembled transcripts. Functional annotation was performed with a DIAMOND search of NCBI's nr database and PANNZER2.

### Orthology inference, phylogenetic analyses, and divergence time estimates

Orthology-inference analysis was performed on a splice-filtered proteome data set of 49 species from 15 metazoan phyla and four nonmetazoan outgroups. Orthology assignment was performed using OrthoFinder version 2.2.7 (Emms and Kelly 2019). Divergence times between *H. echinata* and *H. symbiolongicarpus* and between other cnidarian lineages were estimated by inferring a time-calibrated maximum-likelihood phylogeny using only SCOs. The topology of our maximum-likelihood phylogenetic tree was inferred using IQ-Tree2, and divergence date estimates were calculated for major nodes on the tree using a Langley–Fitch approach together with the TN algorithm, using r8s version 1.8.1 (Fig. 1B).

### Orthogroup lineage specificity and overall patterns

Output from OrthoFinder was processed using custom R scripts (Supplemental Data S13–S15; R Core Team 2021) to analyze patterns of the presence and absence of orthogroups across taxa and characterize the taxon specificity of each orthogroup. Taxon specificity and other related information for each *H. symbiolongicarpus* and *H. echinata* gene model can be found in Supplemental Table S11 (tabs X.10, X.11).

### Estimating the evolutionary dynamics of gene families using CAFE

We used the software package CAFE v.4.2.1 (https://hahnlab.github.io/CAFE/) to estimate ancestral gene family sizes and changes in gene family size among 15 cnidarian species, as well as to infer which gene families are evolving significantly faster in specific cnidarian lineages. As input, we provided our time-calibrated tree and the gene counts per species for a subset of the orthogroups inferred by OrthoFinder.

### Single-cell transcriptomics of adult animals and OrthoMarker analyses

Tissue from adult male *H. symbiolongicarpus* clone 291-10 was dissociated in 1% pronase E in calcium- and magnesium-free artificial seawater (CMFASW) with EGTA for 90 min total. The cell suspension was filtered through a 70-µm Flowmi cell filter, and pelleted at 300 rcf for 5 min at 4C, and the pellet was gently resuspended in either CMFASW without EGTA or 3×PBS. This cell suspension was filtered through a 40-µm Flowmi cell filter and placed on ice. The 10x single-cell 3′ version 3 RNA-seq library construction was performed at the University of Florida's Interdisciplinary Center for Biotechnology Research. Libraries were sequenced at the NIH Intramural Sequencing Center using the Illumina NovaSeq 6000_SP sequencing system. The 10x Cell Ranger pipeline version 7.0.1 was used to preprocess the sequencing data for downstream analysis. Seurat version 4.3.0 was used to generate clusters, find marker genes for each cluster, and further analyze the data. A marker gene list for each cluster was created using Seurat and the settings used by Siebert et al. (2019). The OrthoFinder results (Supplemental Data S3–S9) were used to apply several levels of taxon specificity to the marker gene list using R and the “dyplr” package. The R package “ggplot” was used to create the bar plot and histogram shown as Figure 5, A and B, respectively. Markers were validated with FISH (Supplemental Material), and primers for those genes are found in Supplemental Table S31.

### Data sets

All sequencing read data related to this project can be accessed from the NCBI BioProject database (https://www.ncbi.nlm.nih.gov/bioproject/) under accession numbers PRJNA807936 (*H. symbiolongicarpus*) and PRJNA812777 (*H. echinata*).

## Data access

The whole-genome shotgun project data generated in this study have been submitted to DDBJ/ENA/GenBank under accession numbers JARYZW000000000 (*H. symbiolongicarpus*) and JASGCC000000000 (*H. echinata*). The version described in this paper is version JARYZW010000000 (*H. symbiolongicarpus*) and JASGCC010000000 (*H. echinata*). The PacBio sequencing reads generated in this study have been submitted to the NCBI Sequence Read Archive (https://www.ncbi.nlm.nih.gov/sra) under accession numbers SRX14365301, SRX14365302, SRX14365308, SRX14365309, and SRX14365310 (*H. echinata*) and at SRX14210182, SRX14210183, and SRX14210193 (*H. symbiolongicarpus*). All Dovetail Chicago library sequencing data and mapping data for scaffolding assemblies, as well as additional data available for download, can be found at https://research.nhgri.nih.gov/hydractinia/download/index.cgi?dl=sd. All custom scripts are available in Supplemental Code S1. The *Hydractinia* Genome Project portal (https://research.nhgri.nih.gov/hydractinia) provides a rich source of data for both species, including a BLAST interface, genome browser, DNA and protein sequence downloads, and functional annotation of gene models, as well as a single-cell browser and RNA-seq expression data.

## Supplementary Material

Supplement 1

Supplement 2

Supplement 3

Supplement 4

Supplement 5

Supplement 6

Supplement 7

Supplement 8

Supplement 9

Supplement 10

Supplement 11
