# Supplementary material for: The genome of the colonial hydroid Hydractinia reveals that their stem cells use a toolkit of evolutionarily shared genes with all animals
Source: Genome Res. 2024 Mar;34(3):498–513. doi: 10.1101/gr.278382.123 (PMC11067881; doi:10.1101/gr.278382.123)
Supplement: Supplement 9 [file Supplemental_Code_S1.zip › ./Supplemental_Code_S5.html]

```
from Bio import SeqIO
```

```
def parse_fasta(fasta_file):
    id_length = {}
    for seq_record in SeqIO.parse(fasta_file, "fasta"):
        new_id = seq_record.id.split("|")[1]
        length = len(seq_record)
        id_length[new_id] = length
    return(id_length)
```

```
def select_iso(splice_file, id_length):
    file = str(splice_file)
    prefix = file.split("/")[1]
    prefix = prefix.split("_")
    prefix = (prefix[0][0]).upper() + prefix[1][0:3] + "|"
    with open(splice_file) as isoforms:
        longest_isoforms = []
        for line in isoforms:
            line = line.rstrip().split(';')
            lengths = { gene: id_length.get(gene, 0) for gene in line}
            longest_isoforms.append(prefix + (max(lengths, key=lengths.get)))
    return(longest_isoforms)
```

```
# Read list of file names
file_dict = {}
with open('filenames.txt') as filenames:
    for line in filenames:
        fasta = line.split("\t")[0]
        splice = line.split("\t")[1].rstrip()
        file_dict[fasta] = splice
```

```
# Create new FASTA files
for fasta, splice in file_dict.items():
    id_length = parse_fasta(fasta)
    longest_iso = select_iso(splice, id_length)
    prot_dict = SeqIO.to_dict(SeqIO.parse(fasta, "fasta"))
    new_prot_dict = { gene_id: prot_dict[gene_id] for gene_id in longest_iso }
    out_name = fasta.split("/")[1]
    out_name = out_name.split(".")[0] + "_long_iso.fa"
    print("writing ", out_name)
    with open(out_name, 'w') as outfile:
        SeqIO.write(new_prot_dict.values(), outfile, 'fasta')
```
