# Supplementary material for: The genome of the colonial hydroid Hydractinia reveals that their stem cells use a toolkit of evolutionarily shared genes with all animals
Source: Genome Res. 2024 Mar;34(3):498–513. doi: 10.1101/gr.278382.123 (PMC11067881; doi:10.1101/gr.278382.123)
Supplement: Supplement 10 [file Supplemental_Data.zip › Supplemental_Data/README.docx]

**Supplemental Data S1**. Text file detailing how different transcriptomes were generated for *Hydractinia echinata* and *Hydractinia symbiolognicarpus*. Details include how assemblies were generated using Trinity, TopHat, and HISAT2/StringTie and includes parameters used to generate each assembly.

**Supplemental Data S2**. Text file detailing the pipeline for gene model prediction for both *Hydractinia* species. The file covers the steps used and the parameters used at each step. This pipeline involves PASA and Augustus.

**Supplemental Data S3.** Orthogroups_Dec_20.tsv is a tab separated text file that is an OrthoFinder results file. Each row contains the genes belonging to a single orthogroup. The genes from each orthogroup are organized into columns, one per species.

**Supplemental Data S4**. Orthogroups_SpeciesOverlaps.tsv is a tab separated text file that is an OrthoFinder results file that contains the number of orthogroups shared between each species-pair as a square matrix.

**Supplemental Data S5.** Orthogroups_UnassignedGenes_Dec20.tsv is a tab separated text file that is an OrthoFinder results file that is identical in format to Orthogroups.tsv but contains all of the genes that were not assigned to any orthogroup.

**Supplemental Data S6**. Orthogroups.GeneCount_dec_20.tsv is a tab separated text file that is an OrthoFinder results file that is identical in format to Orthogroups.csv but contains counts of the number of genes for each species in each orthogroup.

**Supplemental Data S7**. Orthogroups.txt is an OrthoFinder results file containing the orthogroups described in the Orthogroups_Dec_20.tsv file (Supplemental Data S3) but using the OrthoMCL output format.

**Supplemental Data S8**. Statistics_Overall.tsv is a tab separated text file that is an OrthoFinder results file that contains general statistics about orthogroup sizes and proportion of genes assigned to orthogroups.

**Supplemental Data S9**. Statistics_PerSpecies.tsv is a tab separated text file that is an OrthoFinder results file that contains the same information as the Statistics_Overall.tsv file (Supplemental Data S8) but for each individual species.

**Supplemental Data S10**. concat_trim.fa is a text file in fasta format that represents the final input matrix provided to IQ-TREE2. It is comprised of a subset of single copy ortholog (SCO) sequences from our orthogroup data set. These SCOs were chosen for their presence in at least 12 of 15 cnidarian species; four bilaterian and three non-bilaterian outgroup species that also contained these SCOs were included in the analysis. The final concatenated, aligned, and trimmed data set included sequences from 216 orthogroups, resulting in an alignment of 50,457 nucleotides.

**Supplemental Data S11**. concat_trim.fa.iqtree is a text file that is the main IQ-TREE2 output file. It includes a text representation of the final maximum likelihood tree.

**Supplemental Data S12**. r8s_simple_aque_root_with_chronogram.out is a text file that is the output from r8s.

**Supplemental Data S13**. orthogroup_queries.html is an html-formatted R markdown file that provides details of R code and information related to calculating the numbers of overlaps of orthogroups between major groups of cnidarians and bilaterians.

**Supplemental Data S14**. phylum_specific_and_unassigned.html is an html-formatted R markdown file that provides details of R code and information related to calculating the numbers of phylum specific and unassigned orthogroups.

**Supplemental Data S15**. unassigned_annotation.html is an html-formatted R markdown file that provides details of R code and information related to annotating unassigned genes from the OrthoFinder results.

**Supplemental Data S16**. NoTadh_r8s_gene_counts_sampled_FULL_input.txt is a text file that represents the full matrix of gene family sizes per species estimated by OrthoFinder for gene families present in the selected species for the CAFÉ analysis.

**Supplemental Data S17**. NoTadh_r8s_gene_counts_sampled_small_input.txt is a text file that represents the reduced matrix of gene family sizes per species estimated by OrthoFinder for gene families present in the selected species with fewer than 100 sequences per species. Before running CAFE to estimate ancestral gene family sizes and gene family gains/losses over the selected subtree, one first needs to estimate a value for lambda (λ), the symmetrical gene birth-death rate for the entire tree expressed in gains or losses per gene per million years. To estimate λ, it is recommended that only orthogroups with low variance in gene family size amongst taxa be used; this can be achieved by selecting those with fewer than 100 sequences per species.

**Supplemental Data S18**. NoTadh.constrained.FULL.cafe_test.sh: Script submitted to run CAFE to do actual estimates of evolutionary dynamics, using as input the full set of possible orthogroups inferred to be in the common ancestor of the included species (Supplemental Data S28) and the value for λ calculated using Supplemental Data S19.

**Supplemental Data S19**. NoTadh.constrained.LAMBDA.cafe_test.sh: Script submitted to run CAFE in order to infer the λ parameter (symmetrical gene birth-death rate) for our data, including an input tree and directing CAFE to analyze only those groups those orthogroups that meet requirements (see Supplemental Data S29). To estimate λ, it is recommended that only orthogroups with low variance in gene family size amongst taxa be used; this can be achieved by selecting those with less than 100 sequences per species.

**Supplemental Data S20**. NoTadh.constrained.CAFE.report.cafe: Report generated from raw CAFE output for the run using Supplemental Data S18, using accessory scripts included with the CAFE installation. Explanation of the data found in this file can be found in CAFE documentation: [https://hahnlab.github.io/CAFE/src_docs/html/Report.html](https://nam10.safelinks.protection.outlook.com/?url=https%3A%2F%2Fhahnlab.github.io%2FCAFE%2Fsrc_docs%2Fhtml%2FReport.html&data=05%7C02%7Cchristine.schnitzler%40whitney.ufl.edu%7C681069e07da445a617b308dc2d8c0bbc%7C0d4da0f84a314d76ace60a62331e1b84%7C0%7C0%7C638435326739034442%7CUnknown%7CTWFpbGZsb3d8eyJWIjoiMC4wLjAwMDAiLCJQIjoiV2luMzIiLCJBTiI6Ik1haWwiLCJXVCI6Mn0%3D%7C0%7C%7C%7C&sdata=BX4ZmjM8xQHmPgny40SyMQQCSbqXMfphqZoYQi4n%2Fj4%3D&reserved=0)

**Supplemental Data S21**. NoTadh.constrained.FULL.log: Progress log produced during running of CAFE as specified in Supplemental Data S18.

**Supplemental Data S22**. NoTadh.constrained.LAMBDA.log: Progress log produced during running of CAFE as specified in Suppelmental Data S19 to estimate λ parameter.

**Supplemental Data S23**. NoTadh.constrained.LAMBDA.report.cafe:  Report generated from raw CAFE output for the run using Supplemental Data S19, using accessory scripts included with the CAFE installation. Explanation of the data found in this file can be found in CAFE documentation: [https://hahnlab.github.io/CAFE/src_docs/html/Report.html](https://nam10.safelinks.protection.outlook.com/?url=https%3A%2F%2Fhahnlab.github.io%2FCAFE%2Fsrc_docs%2Fhtml%2FReport.html&data=05%7C02%7Cchristine.schnitzler%40whitney.ufl.edu%7C681069e07da445a617b308dc2d8c0bbc%7C0d4da0f84a314d76ace60a62331e1b84%7C0%7C0%7C638435326739050073%7CUnknown%7CTWFpbGZsb3d8eyJWIjoiMC4wLjAwMDAiLCJQIjoiV2luMzIiLCJBTiI6Ik1haWwiLCJXVCI6Mn0%3D%7C0%7C%7C%7C&sdata=sT8af4G9Yr4a7U0IB%2FPGcqMvjozJflBVWEy5JmS06co%3D&reserved=0)

**Supplemental Data S24**. NoTadh.constrained.summary_anc.txt: Output produced by running accessory scripts on output from script S18.  Each row contains counts of members of each gene family present inferred to be present at the common ancestor (i.e. at each node). Node numbers are as designated in the tree on top of Supplemental Data S25.

**Supplemental Data S25**. NoTadh.constrained.summary_fams.txt: Output produced by running accessory scripts on output from Supplemental Data S18. This shows how many rapidly evolving families, and their identities, were found overall on the tree, and for each species (terminal branch) and on each internal branch on the input tree. Nodes and species are named in accordance with the version of the input tree rendered at the top of this file.

**Supplemental Data S26**. NoTadh.constrained.summary_node.txt: Output produced by running accessory scripts on output from Supplemental Data S18. This gives a per-node (or per-terminal-taxon) counts of expansions, contractions and significantly rapidly evolving families. Node numbers are as designated in the tree on top of Supplemental Data S25.

**Supplemental Data S27**. NoTadh.constrained.summary_pub.txt: “Publication friendly” per-species summary of the results in Supplemental Data S20 across all branches of the tree. Numbers in parentheses indicate counts of significantly rapidly evolving families in relevant categories.

**Supplemental Data S28**. README-gff.md is a file that provides detailed explanation of all *Hydractinia* RNA annotation GFF files (Supplemental Data S29-S36).

**Supplemental Data S29**. hech.rfam.detailed.gff is a gff file for Rfam predictions (all RNAs except tRNA) of *H. echinata*, with all metadata included.

**Supplemental Data S30**. hech.rfam.minimal.gff is a gff file Rfam predictions (all RNAs except tRNA) of *H. echinata*, without metadata.

**Supplemental Data S31**. hech.trna.detailed.gff is a gff file for tRNA predictions of *H. echinata*, with all metadata included.

**Supplemental Data S32**. hech.trna.minimal.gff is a gff file for for tRNA predictions of *H. echinata*, without metadata.

**Supplemental Data S33**. hsym.rfam.detailed.gff is a gff file for Rfam predictions (all RNAs except tRNA) of *H. symbiolongicarpus*, with all metadata included.

**Supplemental Data S34**. hsym.rfam.minimal.gff is a gff file for Rfam predictions (all RNAs except tRNA) of *H. symbiolongicarpus*, without metadata.

**Supplemental Data S35**. hsym.trna.detailed.gff is a gff file for tRNA predictions of *H. symbiolongicarpus*, with all metadata included.

**Supplemental Data S36**. hsym.trna.minimal.gff is a gff file for tRNA predictions of *H. symbiolongicarpus,* without metadata.

**Supplemental Data S37**. Hech_SuperTree.phy is a phylip-formatted text file containing the final 60 amino acid homeodomain alignment for *H. echinata* that was used for subsequent phylogenetic analyses.

**Supplemental Data S38**. Hysm_SuperTree.phy is a phylip-formatted text file containing the final 60 amino acid homeodomain alignment for *H. symbiolongicarpus* that was used for subsequent phylogenetic analyses.

**Supplemental Data S39**. RAxML_bipartitions.hech_Hbox is a RAXML generated maximum likelihood tree file for the homeobox genes from *H. echinata*. Bootstrap values are included.

**Supplemental Data S40**. RAxML_bipartitions.hsym_Hbox is a RAXML generated maximum likelihood tree file for the homeobox genes from *H. symbiolongicarpus*. Bootstrap values are included.

**Supplemental Data S41**. Hech_SuperTree_renamed.nex.con.tre is the final consensus Bayseian tree file for the homeobox genes from *H. echinata* generated by MRBAYES.

**Supplemental Data S42**. Hsym_SuperTree_renamed.nex.con.tre is the final consensus Bayseian tree file for the homeobox genes from *H. symbiolongicarpus* generated by MRBAYES.

**Supplemental Data S43**. ANTP_aln_Hech.phy is a phylip-formatted text file containing the final homeodomain alignment for the ANTP class of homeobox proteins for *H. echinata* that was used for subsequent phylogenetic analyses.

**Supplemental Data S44**. ANTP_aln_Hsym.phy is a phylip-formatted text file containing the final homeodomain alignment for the ANTP class of homeobox proteins for *H. symbiolongicarpus* that was used for subsequent phylogenetic analyses.

**Supplemental Data S45**. RAxML_bipartitions.hsym_ANTP is a RAXML generated maximum likelihood tree file for the ANTP class of homeobox proteins from *H. symbiolongicarpus*. Bootstrap values are included.

**Supplemental Data S46**. RAxML_bipartitions.hech_ANTP is a RAXML generated maximum likelihood tree file for the ANTP class of homeobox proteins from *H. echinata*. Bootstrap values are included.

**Supplemental Data S47**. ANTP_aln_hech.nex.con.tre is the final consensus Bayseian tree file for the ANTP class of homeobox proteins from *H. echinata* generated by MRBAYES.

**Supplemental Data S48**. ANTP_aln_Hsym.next.con.tre is the final consensus Bayseian tree file for the ANTP class of homeobox proteins from *H. symbiolongicarpus* generated by MRBAYES.

**Supplemental Data S49**. Supplemental_analysis_FINAL.docx is a word document that includes step-by-step descriptions of the computational analyses used to create the final filtered *H. symbiolongicarpus*single cell atlas.
