# Supplementary material for: The genome of the colonial hydroid Hydractinia reveals that their stem cells use a toolkit of evolutionarily shared genes with all animals
Source: Genome Res. 2024 Mar;34(3):498–513. doi: 10.1101/gr.278382.123 (PMC11067881; doi:10.1101/gr.278382.123)
Supplement: Supplement 10 [file Supplemental_Data.zip › Supplemental_Data/Supplemental_Data_S13.html]

Supplemental\_Data\_S13.knit


<!DOCTYPE html>

Orthogroup analysis


Code 

- Show All Code
- Hide All Code
- Download Rmd

# Orthogroup analysis


```
library(tidyverse)
```


```
Registered S3 method overwritten by 'dplyr':
  method           from
  print.rowwise_df     
Registered S3 methods overwritten by 'dbplyr':
  method         from
  print.tbl_lazy     
  print.tbl_sql      
[30m── [1mAttaching packages[22m ───────────────────────────────────────────────────────────────────────────────────────────────────────────────────────────────────── tidyverse 1.3.0 ──[39m
[30m[32m✓[30m [34mggplot2[30m 3.2.1     [32m✓[30m [34mpurrr  [30m 0.3.3
[32m✓[30m [34mtibble [30m 2.1.3     [32m✓[30m [34mdplyr  [30m 0.8.3
[32m✓[30m [34mtidyr  [30m 1.0.0     [32m✓[30m [34mstringr[30m 1.4.0
[32m✓[30m [34mreadr  [30m 1.3.1     [32m✓[30m [34mforcats[30m 0.4.0[39m
[30m── [1mConflicts[22m ──────────────────────────────────────────────────────────────────────────────────────────────────────────────────────────────────────── tidyverse_conflicts() ──
[31mx[30m [34mdplyr[30m::[32mfilter()[30m masks [34mstats[30m::filter()
[31mx[30m [34mdplyr[30m::[32mlag()[30m    masks [34mstats[30m::lag()[39m
```


```
library(viridis)
```


```
Loading required package: viridisLite
```


---

### Read orthogroup table


```
ortho_counts <- read.table("Orthogroups.GeneCount_dec_20.csv")
ortho_counts
```


```
ortho_binary <- ortho_counts[,1:49] #Remove the Totals column
ortho_binary[ortho_binary >= 1] <- 1 #Convert to presence/absence
#ortho_binary[33325,] # The end of the file is single gene orthogroups (unassigned, singletons)
ortho_binary_names <- ortho_binary %>% mutate(ortho_id = row.names(ortho_counts), gene_counts = rowSums(ortho_counts))
```


---

### Read taxonomy table


```
taxonomy <- read_csv("taxonomy_run3.csv")
```


```
Parsed with column specification:
cols(
  species = [31mcol_character()[39m,
  clade = [31mcol_character()[39m
)
```


```
taxonomy
```


---

### Functions

---

#### Present in at least one of X, absent in all of Y

##### default species for Y: all species not in X

##### species\_comp outputs text: number of orthogroups and total number of genes in orthogroups

##### species\_comp\_num outputs integers


```
# species_names is a vector of species names
species_comp <- function(species_in, species_out, number_select_species = length(species_in)){
  if (missing(species_out)){
    select_species <- ortho_binary %>% select(species_in)
    other_species <- ortho_binary %>% select(-species_in)
    specific_orthogroups <- ortho_binary_names[rowSums(select_species) == number_select_species & rowSums(other_species) == 0,] %>% select(ortho_id)
    gene_number <- sum(ortho_counts[specific_orthogroups$ortho_id,species_in])
    out_species_names <- "all other species"
  }
  else{
    select_species <- ortho_binary %>% select(species_in)
    other_species <- ortho_binary %>% select(species_out)
    specific_orthogroups <- ortho_binary_names[rowSums(select_species) == number_select_species & rowSums(other_species) == 0,] %>% select(ortho_id)
    out_species_names <- paste(colnames(other_species), collapse = ",")
    gene_number <- sum(rowSums(ortho_counts[specific_orthogroups$ortho_id,species_in]))
  }
  ortho_number <- nrow(specific_orthogroups)
  in_species_names <- paste(colnames(select_species), collapse = ",")
  output <- paste("present in: ", in_species_names, "\n", "absent in: ", out_species_names, "\n", "number of orthogroups: ", ortho_number, "\n", "number of genes: ",gene_number,  sep = "")
  cat(output)
  }
```


---


```
species_comp_num <- function(species_in, species_out, number_select_species = length(species_in)){
  if (missing(species_out)){
    select_species <- ortho_binary %>% select(species_in)
    other_species <- ortho_binary %>% select(-species_in)
    specific_orthogroups <- ortho_binary_names[rowSums(select_species) == number_select_species & rowSums(other_species) == 0,] %>% select(ortho_id)
    gene_number <- sum(ortho_counts[specific_orthogroups$ortho_id,species_in])
  }
  else{
    select_species <- ortho_binary %>% select(species_in)
    other_species <- ortho_binary %>% select(species_out)
    specific_orthogroups <- ortho_binary_names[rowSums(select_species) == number_select_species & rowSums(other_species) == 0,] %>% select(ortho_id)
    gene_number <- sum(rowSums(ortho_counts[specific_orthogroups$ortho_id,species_in]))
  }
  ortho_number <- nrow(specific_orthogroups)
  output <- c(ortho_number, gene_number)
  output
  }
```


---

#### Present in at least one of X and in at least one of y, lost in all of Z

##### default species for Z: all species not in X or Y


```
one_X_one_Y_none_Z <- function(x, y, z){
  ancestral_species <- ortho_binary %>% select(x)
  shared_species <- ortho_binary %>% select(y)
  if (missing(z)){
    shared_orthogroups <- ortho_binary[rowSums(ancestral_species) >= 1 & rowSums(shared_species) >=1,]
    shared_num <- nrow(shared_orthogroups)
    anc_names <- paste(x, collapse = ",")
    shared_names <- paste(colnames(shared_species), collapse = ",")
    output <- paste(c("Present in at least one of: ", anc_names, "\nand in at least one of: ", shared_names, ": ", shared_num), collapse = "")
    cat(output)
  }
  else{
    for (species in z){
      if (species %in% colnames(y)){
        shared_species <- shared_species %>% (-species)
      }
    } 
  lost_species <- ortho_binary %>% select(z)
  lost_orthogroups <- ortho_binary[rowSums(ancestral_species) >= 1 & rowSums(shared_species) >=1 & rowSums(lost_species) == 0,]
  lost_num <- nrow(lost_orthogroups)
  anc_names <- paste(x, collapse = ",")
  shared_names <- paste(colnames(shared_species), collapse = ",")
  lost_names <- paste(z, collapse = ",")
  output <- paste(c("Present in at least one of: ", anc_names, "\nand in at least one of: ", shared_names, "\nand lost in: ", lost_names, ": ", lost_num), collapse = "")
  cat(output)
  }
}
```


---

#### Present in at least one of X, none of Y


```
one_X_none_Y <- function(x, y){
  select_species <- ortho_binary %>% select(x)
  other_species <- ortho_binary %>% select(y)
  specific_orthogroups <- ortho_binary[rowSums(select_species)  >=1 & rowSums(other_species) == 0,]
  num <- nrow(specific_orthogroups)
  at_least_one_names <- paste(x, collapse = ",")
  none_names <- paste(y, collapse = ",")
  output <- paste(c("Present in at least one of: ", at_least_one_names, "\nand in none of: ", none_names, ": ", num), collapse = "")
  cat(output)
}
```


---

#### Present in at least one of X, and at least one of Y, and lost in at least one of Z

##### Used to calculate losses since ancestor


```
one_X_one_Y_lost_one_Z <- function(ancestors, shared, lost){
  ancestral_species <- ortho_binary %>% select(ancestors)
  shared_species <- ortho_binary %>% select(shared)
  lost_number <- length(lost)
  for (species in lost){
    if (species %in% colnames(shared)){
      shared_species <- shared_species %>% (-species)
    }
  }
  lost_species <- ortho_binary %>% select(lost)
  lost_orthogroups <- ortho_binary[rowSums(ancestral_species) >= 1 & rowSums(shared_species) >=1 & rowSums(lost_species) < lost_number,]
  lost_num <- nrow(lost_orthogroups)
  anc_names <- paste(ancestors, collapse = ",")
  shared_names <- paste(colnames(shared_species), collapse = ",")
  lost_names <- paste(lost, collapse = ",")
  output <- paste(c("Present in at least one of: ", anc_names, "\nand in at least one of: ", shared_names, "\nand lost in at least one of: ", lost_names, ": ", lost_num), collapse = "")
  cat(output)
}
```


---

#### Count orthogroups in at least x species in taxon


```
count_orthogroups <- function(species){
  num_species <- length(species)
  og_counts <- integer(0)
  gene_counts <- integer(0)
  for (i in 1:num_species){
    comp <- species_comp_num(species, number_select_species = i)
    num <- comp[1]
    genes <- comp[2]
    og_counts[i] <- num
    gene_counts[i] <- genes
    print(paste("Number of species: ", i, " ; Number of orthogroups: ", num, " ; Number of genes: ", genes))
  }
  rev_og_count <- rev(og_counts)
  rev_gene_counts <- rev(gene_counts)
  for (i in 1:num_species){
    j <-  num_species - (i - 1)
    og_cumulative_sum <- sum(rev_og_count[1:i])
    gene_cumulative_sum <- sum(rev_gene_counts[1:i])
    print(paste("Present in at least ", j, " species: ", og_cumulative_sum, " orthogroups; ",  gene_cumulative_sum, " genes" ))
  }
}
```


---

#### Counts orthogroups present in a taxon, regardless of other taxa


```
shared_orthogroups <- function(species_in){
  select_species <- ortho_binary %>% select(species_in)
  other_species <- ortho_binary %>% select(-species_in)
  #number_select_species <- length(species_in)
  specific_orthogroups <- ortho_binary[rowSums(select_species) == length(species_in),]
  ortho_number <- nrow(specific_orthogroups)
  in_species_names <- paste(colnames(select_species), collapse = ",")
  output <- paste("present in: ", in_species_names, "\n", "number of orthogroups: ", ortho_number, "\n", sep = "")
  cat(output)
}
```


---

#### Subset and group species from taxon


```
select_clade <- function(clade_name){
  clade <- taxonomy %>% filter(clade == clade_name)
  select_species <- clade$species
  select_species
}
```


```
fuse_taxa <- function(clade_names){
  select_species <- taxonomy %>% filter(clade %in% clade_names)
  species_names <- select_species$species
  species_names
}
```


---


---

### Query orthofinder output

---

#### Orthogroups specific to Hydractinia

##### genus Hydractinia only


```
hydractinia <- c("hydractinia_echinata", "hydractinia_symbiolongicarpus")
species_comp(hydractinia)
```


```
present in: hydractinia_echinata,hydractinia_symbiolongicarpus
absent in: all other species
number of orthogroups: 1564
number of genes: 4819
```


---

##### Hydractinia echinata only


```
species_comp("hydractinia_echinata")
```


```
present in: hydractinia_echinata
absent in: all other species
number of orthogroups: 46
number of genes: 149
```


---

##### Hydractinia symbiolongicarpus only


```
species_comp("hydractinia_symbiolongicarpus")
```


```
present in: hydractinia_symbiolongicarpus
absent in: all other species
number of orthogroups: 15
number of genes: 37
```


---

##### Orthogroups specific to one cnidarian


```
cnidaria <- fuse_taxa(c("Actinaria", "Hexacorallia", "Octocorallia", "Scyphozoa", "Cubozoa", "Hydrozoa",  "Myxozoa"))
cnidaria
```


```
 [1] "exaiptasia_pallida"            "nematostella_vectensis"        "orbicella_faveolata"           "acropora_digitifera"           "pocillopora_damicornis"       
 [6] "renilla_muelleri"              "dendronephthya_gigantea"       "aurelia_aurita_atlantic"       "aurelia_aurita_pacific"        "nemopilema_nomurai"           
[11] "hydractinia_echinata"          "hydractinia_symbiolongicarpus" "hydra_magnipapillata"          "clytia_hemisphaerica"          "morbakka_virulenta"           
[16] "kudoa_iwatai"
```


```
for (species in cnidaria){
  species_comp(species)
  cat("\n\n")
}
```


```
present in: exaiptasia_pallida
absent in: all other species
number of orthogroups: 21
number of genes: 106

present in: nematostella_vectensis
absent in: all other species
number of orthogroups: 19
number of genes: 69

present in: orbicella_faveolata
absent in: all other species
number of orthogroups: 9
number of genes: 23

present in: acropora_digitifera
absent in: all other species
number of orthogroups: 20
number of genes: 52

present in: pocillopora_damicornis
absent in: all other species
number of orthogroups: 5
number of genes: 12

present in: renilla_muelleri
absent in: all other species
number of orthogroups: 54
number of genes: 181

present in: dendronephthya_gigantea
absent in: all other species
number of orthogroups: 43
number of genes: 153

present in: aurelia_aurita_atlantic
absent in: all other species
number of orthogroups: 32
number of genes: 109

present in: aurelia_aurita_pacific
absent in: all other species
number of orthogroups: 67
number of genes: 164

present in: nemopilema_nomurai
absent in: all other species
number of orthogroups: 28
number of genes: 98

present in: hydractinia_echinata
absent in: all other species
number of orthogroups: 46
number of genes: 149

present in: hydractinia_symbiolongicarpus
absent in: all other species
number of orthogroups: 15
number of genes: 37

present in: hydra_magnipapillata
absent in: all other species
number of orthogroups: 154
number of genes: 864

present in: clytia_hemisphaerica
absent in: all other species
number of orthogroups: 81
number of genes: 460

present in: morbakka_virulenta
absent in: all other species
number of orthogroups: 52
number of genes: 172

present in: kudoa_iwatai
absent in: all other species
number of orthogroups: 8
number of genes: 49
```


---

#### Hydrozoa queries

---

##### Orthogroups present in all hydrozoans but no other taxa


```
hydrozoa <- select_clade("Hydrozoa")
species_comp(hydrozoa)
```


```
present in: hydractinia_echinata,hydractinia_symbiolongicarpus,hydra_magnipapillata,clytia_hemisphaerica
absent in: all other species
number of orthogroups: 209
number of genes: 1608
```


---

##### Orthogroups present in 3 hydrozoans but no other taxa


```
for (comb in combn(hydrozoa, 3, simplify = F)){
  species_comp(comb)
  cat("\n\n")
}
```


```
present in: hydractinia_echinata,hydractinia_symbiolongicarpus,hydra_magnipapillata
absent in: all other species
number of orthogroups: 129
number of genes: 794

present in: hydractinia_echinata,hydractinia_symbiolongicarpus,clytia_hemisphaerica
absent in: all other species
number of orthogroups: 280
number of genes: 1765

present in: hydractinia_echinata,hydra_magnipapillata,clytia_hemisphaerica
absent in: all other species
number of orthogroups: 11
number of genes: 105

present in: hydractinia_symbiolongicarpus,hydra_magnipapillata,clytia_hemisphaerica
absent in: all other species
number of orthogroups: 12
number of genes: 43
```


---

##### Orthogroups present in 2 hydrozoans but no other taxa


```
for (comb in combn(hydrozoa, 2, simplify = F)){
  species_comp(comb)
  cat("\n\n")
}
```


```
present in: hydractinia_echinata,hydractinia_symbiolongicarpus
absent in: all other species
number of orthogroups: 1564
number of genes: 4819

present in: hydractinia_echinata,hydra_magnipapillata
absent in: all other species
number of orthogroups: 26
number of genes: 114

present in: hydractinia_echinata,clytia_hemisphaerica
absent in: all other species
number of orthogroups: 39
number of genes: 174

present in: hydractinia_symbiolongicarpus,hydra_magnipapillata
absent in: all other species
number of orthogroups: 17
number of genes: 53

present in: hydractinia_symbiolongicarpus,clytia_hemisphaerica
absent in: all other species
number of orthogroups: 35
number of genes: 104

present in: hydra_magnipapillata,clytia_hemisphaerica
absent in: all other species
number of orthogroups: 44
number of genes: 186
```


---

##### Orthogroups present only in Nematostella and Hydra


```
species_comp(c("nematostella_vectensis", "hydra_magnipapillata"))
```


```
present in: nematostella_vectensis,hydra_magnipapillata
absent in: all other species
number of orthogroups: 2
number of genes: 6
```


---


---

#### Anthozoa queries


```
anthozoa <- fuse_taxa(c("Octocorallia", "Hexacorallia", "Actinaria"))
anthozoa
```


```
[1] "exaiptasia_pallida"      "nematostella_vectensis"  "orbicella_faveolata"     "acropora_digitifera"     "pocillopora_damicornis"  "renilla_muelleri"       
[7] "dendronephthya_gigantea"
```


---

##### Orthogroups shared by all anthozoans


```
shared_orthogroups(anthozoa)
```


```
present in: exaiptasia_pallida,nematostella_vectensis,orbicella_faveolata,acropora_digitifera,pocillopora_damicornis,renilla_muelleri,dendronephthya_gigantea
number of orthogroups: 4900
```


---

##### Orthogroups shared by all anthozoans and absent in all other taxa


```
species_comp(anthozoa)
```


```
present in: exaiptasia_pallida,nematostella_vectensis,orbicella_faveolata,acropora_digitifera,pocillopora_damicornis,renilla_muelleri,dendronephthya_gigantea
absent in: all other species
number of orthogroups: 38
number of genes: 469
```


---

##### Orthogroups present in at least one anthozoan and absent in all other taxa


```
count_orthogroups(anthozoa)
```


```
[1] "Number of species:  1  ; Number of orthogroups:  171  ; Number of genes:  596"
[1] "Number of species:  2  ; Number of orthogroups:  1066  ; Number of genes:  2938"
[1] "Number of species:  3  ; Number of orthogroups:  556  ; Number of genes:  2485"
[1] "Number of species:  4  ; Number of orthogroups:  310  ; Number of genes:  1892"
[1] "Number of species:  5  ; Number of orthogroups:  238  ; Number of genes:  1829"
[1] "Number of species:  6  ; Number of orthogroups:  79  ; Number of genes:  972"
[1] "Number of species:  7  ; Number of orthogroups:  38  ; Number of genes:  469"
[1] "Present in at least  7  species:  38  orthogroups;  469  genes"
[1] "Present in at least  6  species:  117  orthogroups;  1441  genes"
[1] "Present in at least  5  species:  355  orthogroups;  3270  genes"
[1] "Present in at least  4  species:  665  orthogroups;  5162  genes"
[1] "Present in at least  3  species:  1221  orthogroups;  7647  genes"
[1] "Present in at least  2  species:  2287  orthogroups;  10585  genes"
[1] "Present in at least  1  species:  2458  orthogroups;  11181  genes"
```


---


---

#### Medusozoa queries

---


```
medusozoa <- fuse_taxa(c("Hydrozoa", "Scyphozoa", "Cubozoa"))
medusozoa
```


```
[1] "aurelia_aurita_atlantic"       "aurelia_aurita_pacific"        "nemopilema_nomurai"            "hydractinia_echinata"          "hydractinia_symbiolongicarpus"
[6] "hydra_magnipapillata"          "clytia_hemisphaerica"          "morbakka_virulenta"
```


---

##### Orthogroups shared by all medusozoans


```
shared_orthogroups(medusozoa)
```


```
present in: aurelia_aurita_atlantic,aurelia_aurita_pacific,nemopilema_nomurai,hydractinia_echinata,hydractinia_symbiolongicarpus,hydra_magnipapillata,clytia_hemisphaerica,morbakka_virulenta
number of orthogroups: 4180
```


---

##### Orthogroups shared by all medusozoans and absent in all other taxa


```
species_comp(medusozoa)
```


```
present in: aurelia_aurita_atlantic,aurelia_aurita_pacific,nemopilema_nomurai,hydractinia_echinata,hydractinia_symbiolongicarpus,hydra_magnipapillata,clytia_hemisphaerica,morbakka_virulenta
absent in: all other species
number of orthogroups: 84
number of genes: 935
```


---

##### Orthogroups present in at least one medusozoan and absent in all other taxa


```
count_orthogroups(medusozoa)
```


```
[1] "Number of species:  1  ; Number of orthogroups:  475  ; Number of genes:  2053"
[1] "Number of species:  2  ; Number of orthogroups:  3025  ; Number of genes:  9175"
[1] "Number of species:  3  ; Number of orthogroups:  1142  ; Number of genes:  5995"
[1] "Number of species:  4  ; Number of orthogroups:  446  ; Number of genes:  3157"
[1] "Number of species:  5  ; Number of orthogroups:  81  ; Number of genes:  774"
[1] "Number of species:  6  ; Number of orthogroups:  59  ; Number of genes:  662"
[1] "Number of species:  7  ; Number of orthogroups:  78  ; Number of genes:  929"
[1] "Number of species:  8  ; Number of orthogroups:  84  ; Number of genes:  935"
[1] "Present in at least  8  species:  84  orthogroups;  935  genes"
[1] "Present in at least  7  species:  162  orthogroups;  1864  genes"
[1] "Present in at least  6  species:  221  orthogroups;  2526  genes"
[1] "Present in at least  5  species:  302  orthogroups;  3300  genes"
[1] "Present in at least  4  species:  748  orthogroups;  6457  genes"
[1] "Present in at least  3  species:  1890  orthogroups;  12452  genes"
[1] "Present in at least  2  species:  4915  orthogroups;  21627  genes"
[1] "Present in at least  1  species:  5390  orthogroups;  23680  genes"
```


---


---

#### Cnidaria queries


```
cnidaria <- fuse_taxa(c("Actinaria", "Hexacorallia", "Octocorallia", "Scyphozoa", "Cubozoa", "Hydrozoa",  "Myxozoa"))
cnidaria
```


```
 [1] "exaiptasia_pallida"            "nematostella_vectensis"        "orbicella_faveolata"           "acropora_digitifera"           "pocillopora_damicornis"       
 [6] "renilla_muelleri"              "dendronephthya_gigantea"       "aurelia_aurita_atlantic"       "aurelia_aurita_pacific"        "nemopilema_nomurai"           
[11] "hydractinia_echinata"          "hydractinia_symbiolongicarpus" "hydra_magnipapillata"          "clytia_hemisphaerica"          "morbakka_virulenta"           
[16] "kudoa_iwatai"
```


---

##### Orthogroups present in one or more cnidarian and absent in all other species


```
count_orthogroups(cnidaria)
```


```
[1] "Number of species:  1  ; Number of orthogroups:  654  ; Number of genes:  2698"
[1] "Number of species:  2  ; Number of orthogroups:  4255  ; Number of genes:  12756"
[1] "Number of species:  3  ; Number of orthogroups:  1848  ; Number of genes:  9355"
[1] "Number of species:  4  ; Number of orthogroups:  864  ; Number of genes:  5981"
[1] "Number of species:  5  ; Number of orthogroups:  391  ; Number of genes:  3558"
[1] "Number of species:  6  ; Number of orthogroups:  202  ; Number of genes:  2470"
[1] "Number of species:  7  ; Number of orthogroups:  161  ; Number of genes:  2119"
[1] "Number of species:  8  ; Number of orthogroups:  123  ; Number of genes:  1694"
[1] "Number of species:  9  ; Number of orthogroups:  52  ; Number of genes:  894"
[1] "Number of species:  10  ; Number of orthogroups:  35  ; Number of genes:  606"
[1] "Number of species:  11  ; Number of orthogroups:  35  ; Number of genes:  728"
[1] "Number of species:  12  ; Number of orthogroups:  24  ; Number of genes:  648"
[1] "Number of species:  13  ; Number of orthogroups:  31  ; Number of genes:  830"
[1] "Number of species:  14  ; Number of orthogroups:  35  ; Number of genes:  1051"
[1] "Number of species:  15  ; Number of orthogroups:  34  ; Number of genes:  1097"
[1] "Number of species:  16  ; Number of orthogroups:  2  ; Number of genes:  137"
[1] "Present in at least  16  species:  2  orthogroups;  137  genes"
[1] "Present in at least  15  species:  36  orthogroups;  1234  genes"
[1] "Present in at least  14  species:  71  orthogroups;  2285  genes"
[1] "Present in at least  13  species:  102  orthogroups;  3115  genes"
[1] "Present in at least  12  species:  126  orthogroups;  3763  genes"
[1] "Present in at least  11  species:  161  orthogroups;  4491  genes"
[1] "Present in at least  10  species:  196  orthogroups;  5097  genes"
[1] "Present in at least  9  species:  248  orthogroups;  5991  genes"
[1] "Present in at least  8  species:  371  orthogroups;  7685  genes"
[1] "Present in at least  7  species:  532  orthogroups;  9804  genes"
[1] "Present in at least  6  species:  734  orthogroups;  12274  genes"
[1] "Present in at least  5  species:  1125  orthogroups;  15832  genes"
[1] "Present in at least  4  species:  1989  orthogroups;  21813  genes"
[1] "Present in at least  3  species:  3837  orthogroups;  31168  genes"
[1] "Present in at least  2  species:  8092  orthogroups;  43924  genes"
[1] "Present in at least  1  species:  8746  orthogroups;  46622  genes"
```


---


---

##### Orthogroups present in one non-metazoan and absent in all cnidarians


```
non_metazoans <- select_clade("Non-metazoans")
non_metazoans
```


```
[1] "capsaspora_owczarzaki"    "creolimax_fragrantissima" "monosiga_brevicolis"      "saccharomyces_cerevisiae" "salpingoeca_rosetta"      "sphaeroforma_arctica"
```


```
one_X_none_Y(non_metazoans, cnidaria)
```


```
Present in at least one of: capsaspora_owczarzaki,creolimax_fragrantissima,monosiga_brevicolis,saccharomyces_cerevisiae,salpingoeca_rosetta,sphaeroforma_arctica
and in none of: exaiptasia_pallida,nematostella_vectensis,orbicella_faveolata,acropora_digitifera,pocillopora_damicornis,renilla_muelleri,dendronephthya_gigantea,aurelia_aurita_atlantic,aurelia_aurita_pacific,nemopilema_nomurai,hydractinia_echinata,hydractinia_symbiolongicarpus,hydra_magnipapillata,clytia_hemisphaerica,morbakka_virulenta,kudoa_iwatai: 3310
```


---

##### Orthogroups present in at least one non-metazoan and at least one cnidarian but absent in Hydractinia echinata


```
one_X_one_Y_none_Z(non_metazoans, cnidaria, "hydractinia_echinata")
```


```
Present in at least one of: capsaspora_owczarzaki,creolimax_fragrantissima,monosiga_brevicolis,saccharomyces_cerevisiae,salpingoeca_rosetta,sphaeroforma_arctica
and in at least one of: exaiptasia_pallida,nematostella_vectensis,orbicella_faveolata,acropora_digitifera,pocillopora_damicornis,renilla_muelleri,dendronephthya_gigantea,aurelia_aurita_atlantic,aurelia_aurita_pacific,nemopilema_nomurai,hydractinia_echinata,hydractinia_symbiolongicarpus,hydra_magnipapillata,clytia_hemisphaerica,morbakka_virulenta,kudoa_iwatai
and lost in: hydractinia_echinata: 1209
```


---

##### Orthogroups present in at least one non-metazoan and at least one cnidarian but absent in Hydractinia symbiolongicarpus


```
one_X_one_Y_none_Z(non_metazoans, cnidaria, "hydractinia_symbiolongicarpus")
```


```
Present in at least one of: capsaspora_owczarzaki,creolimax_fragrantissima,monosiga_brevicolis,saccharomyces_cerevisiae,salpingoeca_rosetta,sphaeroforma_arctica
and in at least one of: exaiptasia_pallida,nematostella_vectensis,orbicella_faveolata,acropora_digitifera,pocillopora_damicornis,renilla_muelleri,dendronephthya_gigantea,aurelia_aurita_atlantic,aurelia_aurita_pacific,nemopilema_nomurai,hydractinia_echinata,hydractinia_symbiolongicarpus,hydra_magnipapillata,clytia_hemisphaerica,morbakka_virulenta,kudoa_iwatai
and lost in: hydractinia_symbiolongicarpus: 1212
```


---

##### Orthogroups present in at least one non-metazoan and at least one cnidarian but absent in Hydra


```
one_X_one_Y_none_Z(non_metazoans, cnidaria, "hydra_magnipapillata")
```


```
Present in at least one of: capsaspora_owczarzaki,creolimax_fragrantissima,monosiga_brevicolis,saccharomyces_cerevisiae,salpingoeca_rosetta,sphaeroforma_arctica
and in at least one of: exaiptasia_pallida,nematostella_vectensis,orbicella_faveolata,acropora_digitifera,pocillopora_damicornis,renilla_muelleri,dendronephthya_gigantea,aurelia_aurita_atlantic,aurelia_aurita_pacific,nemopilema_nomurai,hydractinia_echinata,hydractinia_symbiolongicarpus,hydra_magnipapillata,clytia_hemisphaerica,morbakka_virulenta,kudoa_iwatai
and lost in: hydra_magnipapillata: 1386
```


---

##### Orthogroups present in at least one non-metazoan and at least one cnidarian but absent in both Hydractinia


```
one_X_one_Y_none_Z(non_metazoans, cnidaria, hydractinia)
```


```
Present in at least one of: capsaspora_owczarzaki,creolimax_fragrantissima,monosiga_brevicolis,saccharomyces_cerevisiae,salpingoeca_rosetta,sphaeroforma_arctica
and in at least one of: exaiptasia_pallida,nematostella_vectensis,orbicella_faveolata,acropora_digitifera,pocillopora_damicornis,renilla_muelleri,dendronephthya_gigantea,aurelia_aurita_atlantic,aurelia_aurita_pacific,nemopilema_nomurai,hydractinia_echinata,hydractinia_symbiolongicarpus,hydra_magnipapillata,clytia_hemisphaerica,morbakka_virulenta,kudoa_iwatai
and lost in: hydractinia_echinata,hydractinia_symbiolongicarpus: 1024
```


---

##### Orthogroups present in at least one non-metazoan and at least one cnidarian but absent in Clytia


```
one_X_one_Y_none_Z(non_metazoans, cnidaria, "clytia_hemisphaerica")
```


```
Present in at least one of: capsaspora_owczarzaki,creolimax_fragrantissima,monosiga_brevicolis,saccharomyces_cerevisiae,salpingoeca_rosetta,sphaeroforma_arctica
and in at least one of: exaiptasia_pallida,nematostella_vectensis,orbicella_faveolata,acropora_digitifera,pocillopora_damicornis,renilla_muelleri,dendronephthya_gigantea,aurelia_aurita_atlantic,aurelia_aurita_pacific,nemopilema_nomurai,hydractinia_echinata,hydractinia_symbiolongicarpus,hydra_magnipapillata,clytia_hemisphaerica,morbakka_virulenta,kudoa_iwatai
and lost in: clytia_hemisphaerica: 1463
```


---

##### Orthogroups present in at least one non-metazoan and Hydra but absent in both Hydractinia


```
one_X_one_Y_none_Z(non_metazoans, "hydra_magnipapillata", hydractinia)
```


```
Present in at least one of: capsaspora_owczarzaki,creolimax_fragrantissima,monosiga_brevicolis,saccharomyces_cerevisiae,salpingoeca_rosetta,sphaeroforma_arctica
and in at least one of: hydra_magnipapillata
and lost in: hydractinia_echinata,hydractinia_symbiolongicarpus: 177
```


---

##### Orthogroups present in at least one non-metazoan and at least one Hydractinia but absent in Hydra


```
one_X_one_Y_none_Z(non_metazoans, hydractinia, "hydra_magnipapillata")
```


```
Present in at least one of: capsaspora_owczarzaki,creolimax_fragrantissima,monosiga_brevicolis,saccharomyces_cerevisiae,salpingoeca_rosetta,sphaeroforma_arctica
and in at least one of: hydractinia_echinata,hydractinia_symbiolongicarpus
and lost in: hydra_magnipapillata: 539
```


---

##### Orthogroups present in at least one non-metazoan but absent in all cnidarians


```
one_X_none_Y(non_metazoans, cnidaria)
```


```
Present in at least one of: capsaspora_owczarzaki,creolimax_fragrantissima,monosiga_brevicolis,saccharomyces_cerevisiae,salpingoeca_rosetta,sphaeroforma_arctica
and in none of: exaiptasia_pallida,nematostella_vectensis,orbicella_faveolata,acropora_digitifera,pocillopora_damicornis,renilla_muelleri,dendronephthya_gigantea,aurelia_aurita_atlantic,aurelia_aurita_pacific,nemopilema_nomurai,hydractinia_echinata,hydractinia_symbiolongicarpus,hydra_magnipapillata,clytia_hemisphaerica,morbakka_virulenta,kudoa_iwatai: 3310
```


---

##### Orthogroups present in at least one non-metazoan and one bilaterian but absent in all cnidarians


```
non_bilaterians <- c(cnidaria, "Porifera", "Ctenophora", "Placozoa", "Non-metazoans")
non_bilaterians
```


```
 [1] "exaiptasia_pallida"            "nematostella_vectensis"        "orbicella_faveolata"           "acropora_digitifera"          
 [5] "pocillopora_damicornis"        "renilla_muelleri"              "dendronephthya_gigantea"       "aurelia_aurita_atlantic"      
 [9] "aurelia_aurita_pacific"        "nemopilema_nomurai"            "hydractinia_echinata"          "hydractinia_symbiolongicarpus"
[13] "hydra_magnipapillata"          "clytia_hemisphaerica"          "morbakka_virulenta"            "kudoa_iwatai"                 
[17] "Porifera"                      "Ctenophora"                    "Placozoa"                      "Non-metazoans"
```


```
bilateria <- taxonomy %>% filter(!clade %in% non_bilaterians)
bilateria <- bilateria$species
bilateria
```


```
 [1] "danio_rerio"                   "xenopus_tropicalis"            "gallus_gallus"                 "homo_sapiens"                 
 [5] "ciona_intestinalis"            "branchiostoma_floridae"        "saccoglossus_kowalevskii"      "acanthaster_planci"           
 [9] "pristionchus_pacificus"        "caenorhabditis_elegans"        "drosophila_melanogaster"       "tribolium_castaneum"          
[13] "daphnia_pulex"                 "ixodes_scapularis"             "lottia_gigantea"               "octopus_bimaculoides"         
[17] "phoronis_australis"            "notospermus_geniculatus"       "schistosoma_mansoni"           "schmidtea_mediterranea"       
[21] "capitella_teleta"              "helobdella_robusta"            "exaiptasia_pallida"            "nematostella_vectensis"       
[25] "orbicella_faveolata"           "acropora_digitifera"           "pocillopora_damicornis"        "renilla_muelleri"             
[29] "dendronephthya_gigantea"       "aurelia_aurita_atlantic"       "aurelia_aurita_pacific"        "nemopilema_nomurai"           
[33] "hydractinia_echinata"          "hydractinia_symbiolongicarpus" "hydra_magnipapillata"          "clytia_hemisphaerica"         
[37] "morbakka_virulenta"            "hofstenia_miamia"              "kudoa_iwatai"
```


```
one_X_one_Y_none_Z(non_metazoans, bilateria, cnidaria)
```


```
Present in at least one of: capsaspora_owczarzaki,creolimax_fragrantissima,monosiga_brevicolis,saccharomyces_cerevisiae,salpingoeca_rosetta,sphaeroforma_arctica
and in at least one of: danio_rerio,xenopus_tropicalis,gallus_gallus,homo_sapiens,ciona_intestinalis,branchiostoma_floridae,saccoglossus_kowalevskii,acanthaster_planci,pristionchus_pacificus,caenorhabditis_elegans,drosophila_melanogaster,tribolium_castaneum,daphnia_pulex,ixodes_scapularis,lottia_gigantea,octopus_bimaculoides,phoronis_australis,notospermus_geniculatus,schistosoma_mansoni,schmidtea_mediterranea,capitella_teleta,helobdella_robusta,exaiptasia_pallida,nematostella_vectensis,orbicella_faveolata,acropora_digitifera,pocillopora_damicornis,renilla_muelleri,dendronephthya_gigantea,aurelia_aurita_atlantic,aurelia_aurita_pacific,nemopilema_nomurai,hydractinia_echinata,hydractinia_symbiolongicarpus,hydra_magnipapillata,clytia_hemisphaerica,morbakka_virulenta,hofstenia_miamia,kudoa_iwatai
and lost in: exaiptasia_pallida,nematostella_vectensis,orbicella_faveolata,acropora_digitifera,pocillopora_damicornis,renilla_muelleri,dendronephthya_gigantea,aurelia_aurita_atlantic,aurelia_aurita_pacific,nemopilema_nomurai,hydractinia_echinata,hydractinia_symbiolongicarpus,hydra_magnipapillata,clytia_hemisphaerica,morbakka_virulenta,kudoa_iwatai: 447
```


---

##### Output these orthogroups


```
x <- ortho_binary %>% select(non_metazoans)
y <- ortho_binary %>% select(bilateria)
z <- ortho_binary %>% select(cnidaria)
lost_orthogroups <- ortho_binary[rowSums(x) >= 1 & rowSums(y) >=1 & rowSums(z) == 0,]
lost_orthogroups
```


---

##### Orthogroups present in non-metazoans and at least one bilaterian but absent in at least one Hydractinia


```
one_X_one_Y_lost_one_Z(non_metazoans, bilateria, hydractinia)
```


```
Present in at least one of: capsaspora_owczarzaki,creolimax_fragrantissima,monosiga_brevicolis,saccharomyces_cerevisiae,salpingoeca_rosetta,sphaeroforma_arctica
and in at least one of: danio_rerio,xenopus_tropicalis,gallus_gallus,homo_sapiens,ciona_intestinalis,branchiostoma_floridae,saccoglossus_kowalevskii,acanthaster_planci,pristionchus_pacificus,caenorhabditis_elegans,drosophila_melanogaster,tribolium_castaneum,daphnia_pulex,ixodes_scapularis,lottia_gigantea,octopus_bimaculoides,phoronis_australis,notospermus_geniculatus,schistosoma_mansoni,schmidtea_mediterranea,capitella_teleta,helobdella_robusta,exaiptasia_pallida,nematostella_vectensis,orbicella_faveolata,acropora_digitifera,pocillopora_damicornis,renilla_muelleri,dendronephthya_gigantea,aurelia_aurita_atlantic,aurelia_aurita_pacific,nemopilema_nomurai,hydractinia_echinata,hydractinia_symbiolongicarpus,hydra_magnipapillata,clytia_hemisphaerica,morbakka_virulenta,hofstenia_miamia,kudoa_iwatai
and lost in at least one of: hydractinia_echinata,hydractinia_symbiolongicarpus: 1844
```


---

##### Orthogroups present in non-metazoans and at least one bilaterian but absent in both Hydractinia


```
one_X_one_Y_none_Z(non_metazoans, bilateria, hydractinia)
```


```
Present in at least one of: capsaspora_owczarzaki,creolimax_fragrantissima,monosiga_brevicolis,saccharomyces_cerevisiae,salpingoeca_rosetta,sphaeroforma_arctica
and in at least one of: danio_rerio,xenopus_tropicalis,gallus_gallus,homo_sapiens,ciona_intestinalis,branchiostoma_floridae,saccoglossus_kowalevskii,acanthaster_planci,pristionchus_pacificus,caenorhabditis_elegans,drosophila_melanogaster,tribolium_castaneum,daphnia_pulex,ixodes_scapularis,lottia_gigantea,octopus_bimaculoides,phoronis_australis,notospermus_geniculatus,schistosoma_mansoni,schmidtea_mediterranea,capitella_teleta,helobdella_robusta,exaiptasia_pallida,nematostella_vectensis,orbicella_faveolata,acropora_digitifera,pocillopora_damicornis,renilla_muelleri,dendronephthya_gigantea,aurelia_aurita_atlantic,aurelia_aurita_pacific,nemopilema_nomurai,hydractinia_echinata,hydractinia_symbiolongicarpus,hydra_magnipapillata,clytia_hemisphaerica,morbakka_virulenta,hofstenia_miamia,kudoa_iwatai
and lost in: hydractinia_echinata,hydractinia_symbiolongicarpus: 1471
```


---

##### Orthogroups present in at least one non-metazoan and one bilaterian but absent in one hydrozoan


```
for (species in hydrozoa){
  one_X_one_Y_none_Z(non_metazoans, bilateria, species)
  cat("\n\n")
}
```


```
Present in at least one of: capsaspora_owczarzaki,creolimax_fragrantissima,monosiga_brevicolis,saccharomyces_cerevisiae,salpingoeca_rosetta,sphaeroforma_arctica
and in at least one of: danio_rerio,xenopus_tropicalis,gallus_gallus,homo_sapiens,ciona_intestinalis,branchiostoma_floridae,saccoglossus_kowalevskii,acanthaster_planci,pristionchus_pacificus,caenorhabditis_elegans,drosophila_melanogaster,tribolium_castaneum,daphnia_pulex,ixodes_scapularis,lottia_gigantea,octopus_bimaculoides,phoronis_australis,notospermus_geniculatus,schistosoma_mansoni,schmidtea_mediterranea,capitella_teleta,helobdella_robusta,exaiptasia_pallida,nematostella_vectensis,orbicella_faveolata,acropora_digitifera,pocillopora_damicornis,renilla_muelleri,dendronephthya_gigantea,aurelia_aurita_atlantic,aurelia_aurita_pacific,nemopilema_nomurai,hydractinia_echinata,hydractinia_symbiolongicarpus,hydra_magnipapillata,clytia_hemisphaerica,morbakka_virulenta,hofstenia_miamia,kudoa_iwatai
and lost in: hydractinia_echinata: 1656

Present in at least one of: capsaspora_owczarzaki,creolimax_fragrantissima,monosiga_brevicolis,saccharomyces_cerevisiae,salpingoeca_rosetta,sphaeroforma_arctica
and in at least one of: danio_rerio,xenopus_tropicalis,gallus_gallus,homo_sapiens,ciona_intestinalis,branchiostoma_floridae,saccoglossus_kowalevskii,acanthaster_planci,pristionchus_pacificus,caenorhabditis_elegans,drosophila_melanogaster,tribolium_castaneum,daphnia_pulex,ixodes_scapularis,lottia_gigantea,octopus_bimaculoides,phoronis_australis,notospermus_geniculatus,schistosoma_mansoni,schmidtea_mediterranea,capitella_teleta,helobdella_robusta,exaiptasia_pallida,nematostella_vectensis,orbicella_faveolata,acropora_digitifera,pocillopora_damicornis,renilla_muelleri,dendronephthya_gigantea,aurelia_aurita_atlantic,aurelia_aurita_pacific,nemopilema_nomurai,hydractinia_echinata,hydractinia_symbiolongicarpus,hydra_magnipapillata,clytia_hemisphaerica,morbakka_virulenta,hofstenia_miamia,kudoa_iwatai
and lost in: hydractinia_symbiolongicarpus: 1659

Present in at least one of: capsaspora_owczarzaki,creolimax_fragrantissima,monosiga_brevicolis,saccharomyces_cerevisiae,salpingoeca_rosetta,sphaeroforma_arctica
and in at least one of: danio_rerio,xenopus_tropicalis,gallus_gallus,homo_sapiens,ciona_intestinalis,branchiostoma_floridae,saccoglossus_kowalevskii,acanthaster_planci,pristionchus_pacificus,caenorhabditis_elegans,drosophila_melanogaster,tribolium_castaneum,daphnia_pulex,ixodes_scapularis,lottia_gigantea,octopus_bimaculoides,phoronis_australis,notospermus_geniculatus,schistosoma_mansoni,schmidtea_mediterranea,capitella_teleta,helobdella_robusta,exaiptasia_pallida,nematostella_vectensis,orbicella_faveolata,acropora_digitifera,pocillopora_damicornis,renilla_muelleri,dendronephthya_gigantea,aurelia_aurita_atlantic,aurelia_aurita_pacific,nemopilema_nomurai,hydractinia_echinata,hydractinia_symbiolongicarpus,hydra_magnipapillata,clytia_hemisphaerica,morbakka_virulenta,hofstenia_miamia,kudoa_iwatai
and lost in: hydra_magnipapillata: 1833

Present in at least one of: capsaspora_owczarzaki,creolimax_fragrantissima,monosiga_brevicolis,saccharomyces_cerevisiae,salpingoeca_rosetta,sphaeroforma_arctica
and in at least one of: danio_rerio,xenopus_tropicalis,gallus_gallus,homo_sapiens,ciona_intestinalis,branchiostoma_floridae,saccoglossus_kowalevskii,acanthaster_planci,pristionchus_pacificus,caenorhabditis_elegans,drosophila_melanogaster,tribolium_castaneum,daphnia_pulex,ixodes_scapularis,lottia_gigantea,octopus_bimaculoides,phoronis_australis,notospermus_geniculatus,schistosoma_mansoni,schmidtea_mediterranea,capitella_teleta,helobdella_robusta,exaiptasia_pallida,nematostella_vectensis,orbicella_faveolata,acropora_digitifera,pocillopora_damicornis,renilla_muelleri,dendronephthya_gigantea,aurelia_aurita_atlantic,aurelia_aurita_pacific,nemopilema_nomurai,hydractinia_echinata,hydractinia_symbiolongicarpus,hydra_magnipapillata,clytia_hemisphaerica,morbakka_virulenta,hofstenia_miamia,kudoa_iwatai
and lost in: clytia_hemisphaerica: 1910
```


---

##### Orthogroups present in at least one non-metazoan and one bilaterian but absent in all hydrozoans


```
one_X_one_Y_none_Z(non_metazoans, bilateria, hydrozoa)
```


```
Present in at least one of: capsaspora_owczarzaki,creolimax_fragrantissima,monosiga_brevicolis,saccharomyces_cerevisiae,salpingoeca_rosetta,sphaeroforma_arctica
and in at least one of: danio_rerio,xenopus_tropicalis,gallus_gallus,homo_sapiens,ciona_intestinalis,branchiostoma_floridae,saccoglossus_kowalevskii,acanthaster_planci,pristionchus_pacificus,caenorhabditis_elegans,drosophila_melanogaster,tribolium_castaneum,daphnia_pulex,ixodes_scapularis,lottia_gigantea,octopus_bimaculoides,phoronis_australis,notospermus_geniculatus,schistosoma_mansoni,schmidtea_mediterranea,capitella_teleta,helobdella_robusta,exaiptasia_pallida,nematostella_vectensis,orbicella_faveolata,acropora_digitifera,pocillopora_damicornis,renilla_muelleri,dendronephthya_gigantea,aurelia_aurita_atlantic,aurelia_aurita_pacific,nemopilema_nomurai,hydractinia_echinata,hydractinia_symbiolongicarpus,hydra_magnipapillata,clytia_hemisphaerica,morbakka_virulenta,hofstenia_miamia,kudoa_iwatai
and lost in: hydractinia_echinata,hydractinia_symbiolongicarpus,hydra_magnipapillata,clytia_hemisphaerica: 1204
```


---

##### Orthogroups present in at least one non-metazoan and one bilaterian but absent in all medusozoans


```
medusozoa <- fuse_taxa(c("Hydrozoa", "Scyphozoa", "Cubozoa"))
medusozoa
```


```
[1] "aurelia_aurita_atlantic"       "aurelia_aurita_pacific"        "nemopilema_nomurai"            "hydractinia_echinata"         
[5] "hydractinia_symbiolongicarpus" "hydra_magnipapillata"          "clytia_hemisphaerica"          "morbakka_virulenta"
```


```
one_X_one_Y_none_Z(non_metazoans, bilateria, medusozoa)
```


```
Present in at least one of: capsaspora_owczarzaki,creolimax_fragrantissima,monosiga_brevicolis,saccharomyces_cerevisiae,salpingoeca_rosetta,sphaeroforma_arctica
and in at least one of: danio_rerio,xenopus_tropicalis,gallus_gallus,homo_sapiens,ciona_intestinalis,branchiostoma_floridae,saccoglossus_kowalevskii,acanthaster_planci,pristionchus_pacificus,caenorhabditis_elegans,drosophila_melanogaster,tribolium_castaneum,daphnia_pulex,ixodes_scapularis,lottia_gigantea,octopus_bimaculoides,phoronis_australis,notospermus_geniculatus,schistosoma_mansoni,schmidtea_mediterranea,capitella_teleta,helobdella_robusta,exaiptasia_pallida,nematostella_vectensis,orbicella_faveolata,acropora_digitifera,pocillopora_damicornis,renilla_muelleri,dendronephthya_gigantea,aurelia_aurita_atlantic,aurelia_aurita_pacific,nemopilema_nomurai,hydractinia_echinata,hydractinia_symbiolongicarpus,hydra_magnipapillata,clytia_hemisphaerica,morbakka_virulenta,hofstenia_miamia,kudoa_iwatai
and lost in: aurelia_aurita_atlantic,aurelia_aurita_pacific,nemopilema_nomurai,hydractinia_echinata,hydractinia_symbiolongicarpus,hydra_magnipapillata,clytia_hemisphaerica,morbakka_virulenta: 869
```


---

##### Orthogroups present in at least one non-metazoan and one bilaterian but absent in Nematostella


```
one_X_one_Y_none_Z(non_metazoans, bilateria, "nematostella_vectensis")
```


```
Present in at least one of: capsaspora_owczarzaki,creolimax_fragrantissima,monosiga_brevicolis,saccharomyces_cerevisiae,salpingoeca_rosetta,sphaeroforma_arctica
and in at least one of: danio_rerio,xenopus_tropicalis,gallus_gallus,homo_sapiens,ciona_intestinalis,branchiostoma_floridae,saccoglossus_kowalevskii,acanthaster_planci,pristionchus_pacificus,caenorhabditis_elegans,drosophila_melanogaster,tribolium_castaneum,daphnia_pulex,ixodes_scapularis,lottia_gigantea,octopus_bimaculoides,phoronis_australis,notospermus_geniculatus,schistosoma_mansoni,schmidtea_mediterranea,capitella_teleta,helobdella_robusta,exaiptasia_pallida,nematostella_vectensis,orbicella_faveolata,acropora_digitifera,pocillopora_damicornis,renilla_muelleri,dendronephthya_gigantea,aurelia_aurita_atlantic,aurelia_aurita_pacific,nemopilema_nomurai,hydractinia_echinata,hydractinia_symbiolongicarpus,hydra_magnipapillata,clytia_hemisphaerica,morbakka_virulenta,hofstenia_miamia,kudoa_iwatai
and lost in: nematostella_vectensis: 1330
```


---

##### Orthologs present in at last one bilaterian and one non-metazoan


```
one_X_one_Y_none_Z(bilateria, non_metazoans)
```


```
Present in at least one of: danio_rerio,xenopus_tropicalis,gallus_gallus,homo_sapiens,ciona_intestinalis,branchiostoma_floridae,saccoglossus_kowalevskii,acanthaster_planci,pristionchus_pacificus,caenorhabditis_elegans,drosophila_melanogaster,tribolium_castaneum,daphnia_pulex,ixodes_scapularis,lottia_gigantea,octopus_bimaculoides,phoronis_australis,notospermus_geniculatus,schistosoma_mansoni,schmidtea_mediterranea,capitella_teleta,helobdella_robusta,exaiptasia_pallida,nematostella_vectensis,orbicella_faveolata,acropora_digitifera,pocillopora_damicornis,renilla_muelleri,dendronephthya_gigantea,aurelia_aurita_atlantic,aurelia_aurita_pacific,nemopilema_nomurai,hydractinia_echinata,hydractinia_symbiolongicarpus,hydra_magnipapillata,clytia_hemisphaerica,morbakka_virulenta,hofstenia_miamia,kudoa_iwatai
and in at least one of: capsaspora_owczarzaki,creolimax_fragrantissima,monosiga_brevicolis,saccharomyces_cerevisiae,salpingoeca_rosetta,sphaeroforma_arctica: 6787
```


---

##### Orthologs present in at last one bilaterian and one Hydractinia


```
one_X_one_Y_none_Z(bilateria, hydractinia)
```


```
Present in at least one of: danio_rerio,xenopus_tropicalis,gallus_gallus,homo_sapiens,ciona_intestinalis,branchiostoma_floridae,saccoglossus_kowalevskii,acanthaster_planci,pristionchus_pacificus,caenorhabditis_elegans,drosophila_melanogaster,tribolium_castaneum,daphnia_pulex,ixodes_scapularis,lottia_gigantea,octopus_bimaculoides,phoronis_australis,notospermus_geniculatus,schistosoma_mansoni,schmidtea_mediterranea,capitella_teleta,helobdella_robusta,exaiptasia_pallida,nematostella_vectensis,orbicella_faveolata,acropora_digitifera,pocillopora_damicornis,renilla_muelleri,dendronephthya_gigantea,aurelia_aurita_atlantic,aurelia_aurita_pacific,nemopilema_nomurai,hydractinia_echinata,hydractinia_symbiolongicarpus,hydra_magnipapillata,clytia_hemisphaerica,morbakka_virulenta,hofstenia_miamia,kudoa_iwatai
and in at least one of: hydractinia_echinata,hydractinia_symbiolongicarpus: 10385
```


---

##### Orthologs present in at least one bilaterian and Mnemiopsis or Amphimedon


```
cteno_sponge <-c("mnemiopsis_leidyi", "amphimedon_queenslandica")
one_X_one_Y_none_Z(bilateria, cteno_sponge)
```


```
Present in at least one of: danio_rerio,xenopus_tropicalis,gallus_gallus,homo_sapiens,ciona_intestinalis,branchiostoma_floridae,saccoglossus_kowalevskii,acanthaster_planci,pristionchus_pacificus,caenorhabditis_elegans,drosophila_melanogaster,tribolium_castaneum,daphnia_pulex,ixodes_scapularis,lottia_gigantea,octopus_bimaculoides,phoronis_australis,notospermus_geniculatus,schistosoma_mansoni,schmidtea_mediterranea,capitella_teleta,helobdella_robusta,exaiptasia_pallida,nematostella_vectensis,orbicella_faveolata,acropora_digitifera,pocillopora_damicornis,renilla_muelleri,dendronephthya_gigantea,aurelia_aurita_atlantic,aurelia_aurita_pacific,nemopilema_nomurai,hydractinia_echinata,hydractinia_symbiolongicarpus,hydra_magnipapillata,clytia_hemisphaerica,morbakka_virulenta,hofstenia_miamia,kudoa_iwatai
and in at least one of: mnemiopsis_leidyi,amphimedon_queenslandica: 7685
```


---

##### Orthologs present in Hydractinia and human


```
one_X_one_Y_none_Z(hydractinia, "homo_sapiens")
```


```
Present in at least one of: hydractinia_echinata,hydractinia_symbiolongicarpus
and in at least one of: homo_sapiens: 5703
```


---

#### Ctenophora queries

---

##### Orthogroups present in ctenophores and bilaterians but absent in cnidarians


```
one_X_one_Y_none_Z("mnemiopsis_leidyi", bilateria, cnidaria)
```


```
Present in at least one of: mnemiopsis_leidyi
and in at least one of: danio_rerio,xenopus_tropicalis,gallus_gallus,homo_sapiens,ciona_intestinalis,branchiostoma_floridae,saccoglossus_kowalevskii,acanthaster_planci,pristionchus_pacificus,caenorhabditis_elegans,drosophila_melanogaster,tribolium_castaneum,daphnia_pulex,ixodes_scapularis,lottia_gigantea,octopus_bimaculoides,phoronis_australis,notospermus_geniculatus,schistosoma_mansoni,schmidtea_mediterranea,capitella_teleta,helobdella_robusta,exaiptasia_pallida,nematostella_vectensis,orbicella_faveolata,acropora_digitifera,pocillopora_damicornis,renilla_muelleri,dendronephthya_gigantea,aurelia_aurita_atlantic,aurelia_aurita_pacific,nemopilema_nomurai,hydractinia_echinata,hydractinia_symbiolongicarpus,hydra_magnipapillata,clytia_hemisphaerica,morbakka_virulenta,hofstenia_miamia,kudoa_iwatai
and lost in: exaiptasia_pallida,nematostella_vectensis,orbicella_faveolata,acropora_digitifera,pocillopora_damicornis,renilla_muelleri,dendronephthya_gigantea,aurelia_aurita_atlantic,aurelia_aurita_pacific,nemopilema_nomurai,hydractinia_echinata,hydractinia_symbiolongicarpus,hydra_magnipapillata,clytia_hemisphaerica,morbakka_virulenta,kudoa_iwatai: 176
```


---

##### Orthogroups present in ctenophores and bilaterians but not in medusozoans


```
one_X_one_Y_none_Z("mnemiopsis_leidyi", bilateria, medusozoa)
```


```
Present in at least one of: mnemiopsis_leidyi
and in at least one of: danio_rerio,xenopus_tropicalis,gallus_gallus,homo_sapiens,ciona_intestinalis,branchiostoma_floridae,saccoglossus_kowalevskii,acanthaster_planci,pristionchus_pacificus,caenorhabditis_elegans,drosophila_melanogaster,tribolium_castaneum,daphnia_pulex,ixodes_scapularis,lottia_gigantea,octopus_bimaculoides,phoronis_australis,notospermus_geniculatus,schistosoma_mansoni,schmidtea_mediterranea,capitella_teleta,helobdella_robusta,exaiptasia_pallida,nematostella_vectensis,orbicella_faveolata,acropora_digitifera,pocillopora_damicornis,renilla_muelleri,dendronephthya_gigantea,aurelia_aurita_atlantic,aurelia_aurita_pacific,nemopilema_nomurai,hydractinia_echinata,hydractinia_symbiolongicarpus,hydra_magnipapillata,clytia_hemisphaerica,morbakka_virulenta,hofstenia_miamia,kudoa_iwatai
and lost in: aurelia_aurita_atlantic,aurelia_aurita_pacific,nemopilema_nomurai,hydractinia_echinata,hydractinia_symbiolongicarpus,hydra_magnipapillata,clytia_hemisphaerica,morbakka_virulenta: 345
```


---

##### Orthogroups present in ctenophores and at least one bilaterian but absent in hydrozoans


```
one_X_one_Y_none_Z("mnemiopsis_leidyi", bilateria, hydrozoa)
```


```
Present in at least one of: mnemiopsis_leidyi
and in at least one of: danio_rerio,xenopus_tropicalis,gallus_gallus,homo_sapiens,ciona_intestinalis,branchiostoma_floridae,saccoglossus_kowalevskii,acanthaster_planci,pristionchus_pacificus,caenorhabditis_elegans,drosophila_melanogaster,tribolium_castaneum,daphnia_pulex,ixodes_scapularis,lottia_gigantea,octopus_bimaculoides,phoronis_australis,notospermus_geniculatus,schistosoma_mansoni,schmidtea_mediterranea,capitella_teleta,helobdella_robusta,exaiptasia_pallida,nematostella_vectensis,orbicella_faveolata,acropora_digitifera,pocillopora_damicornis,renilla_muelleri,dendronephthya_gigantea,aurelia_aurita_atlantic,aurelia_aurita_pacific,nemopilema_nomurai,hydractinia_echinata,hydractinia_symbiolongicarpus,hydra_magnipapillata,clytia_hemisphaerica,morbakka_virulenta,hofstenia_miamia,kudoa_iwatai
and lost in: hydractinia_echinata,hydractinia_symbiolongicarpus,hydra_magnipapillata,clytia_hemisphaerica: 515
```


---

##### Orthogroups present in ctenophores and bilaterians but absent in Hydractinia


```
one_X_one_Y_none_Z("mnemiopsis_leidyi", bilateria, hydractinia)
```


```
Present in at least one of: mnemiopsis_leidyi
and in at least one of: danio_rerio,xenopus_tropicalis,gallus_gallus,homo_sapiens,ciona_intestinalis,branchiostoma_floridae,saccoglossus_kowalevskii,acanthaster_planci,pristionchus_pacificus,caenorhabditis_elegans,drosophila_melanogaster,tribolium_castaneum,daphnia_pulex,ixodes_scapularis,lottia_gigantea,octopus_bimaculoides,phoronis_australis,notospermus_geniculatus,schistosoma_mansoni,schmidtea_mediterranea,capitella_teleta,helobdella_robusta,exaiptasia_pallida,nematostella_vectensis,orbicella_faveolata,acropora_digitifera,pocillopora_damicornis,renilla_muelleri,dendronephthya_gigantea,aurelia_aurita_atlantic,aurelia_aurita_pacific,nemopilema_nomurai,hydractinia_echinata,hydractinia_symbiolongicarpus,hydra_magnipapillata,clytia_hemisphaerica,morbakka_virulenta,hofstenia_miamia,kudoa_iwatai
and lost in: hydractinia_echinata,hydractinia_symbiolongicarpus: 746
```


---

##### Orthogroups present in ctenophores and bilaterians but absent in Hydractinia echinata


```
one_X_one_Y_none_Z("mnemiopsis_leidyi", bilateria, "hydractinia_echinata")
```


```
Present in at least one of: mnemiopsis_leidyi
and in at least one of: danio_rerio,xenopus_tropicalis,gallus_gallus,homo_sapiens,ciona_intestinalis,branchiostoma_floridae,saccoglossus_kowalevskii,acanthaster_planci,pristionchus_pacificus,caenorhabditis_elegans,drosophila_melanogaster,tribolium_castaneum,daphnia_pulex,ixodes_scapularis,lottia_gigantea,octopus_bimaculoides,phoronis_australis,notospermus_geniculatus,schistosoma_mansoni,schmidtea_mediterranea,capitella_teleta,helobdella_robusta,exaiptasia_pallida,nematostella_vectensis,orbicella_faveolata,acropora_digitifera,pocillopora_damicornis,renilla_muelleri,dendronephthya_gigantea,aurelia_aurita_atlantic,aurelia_aurita_pacific,nemopilema_nomurai,hydractinia_echinata,hydractinia_symbiolongicarpus,hydra_magnipapillata,clytia_hemisphaerica,morbakka_virulenta,hofstenia_miamia,kudoa_iwatai
and lost in: hydractinia_echinata: 897
```


---

##### Orthogroups present in ctenophores and bilaterians but absent in Hydractinia symbiolongicarpus


```
one_X_one_Y_none_Z("mnemiopsis_leidyi", bilateria, "hydractinia_symbiolongicarpus")
```


```
Present in at least one of: mnemiopsis_leidyi
and in at least one of: danio_rerio,xenopus_tropicalis,gallus_gallus,homo_sapiens,ciona_intestinalis,branchiostoma_floridae,saccoglossus_kowalevskii,acanthaster_planci,pristionchus_pacificus,caenorhabditis_elegans,drosophila_melanogaster,tribolium_castaneum,daphnia_pulex,ixodes_scapularis,lottia_gigantea,octopus_bimaculoides,phoronis_australis,notospermus_geniculatus,schistosoma_mansoni,schmidtea_mediterranea,capitella_teleta,helobdella_robusta,exaiptasia_pallida,nematostella_vectensis,orbicella_faveolata,acropora_digitifera,pocillopora_damicornis,renilla_muelleri,dendronephthya_gigantea,aurelia_aurita_atlantic,aurelia_aurita_pacific,nemopilema_nomurai,hydractinia_echinata,hydractinia_symbiolongicarpus,hydra_magnipapillata,clytia_hemisphaerica,morbakka_virulenta,hofstenia_miamia,kudoa_iwatai
and lost in: hydractinia_symbiolongicarpus: 878
```

##### Orthogroups present in ctenophores and bilaterians but absent in Clytia


```
one_X_one_Y_none_Z("mnemiopsis_leidyi", bilateria, "clytia_hemisphaerica")
```


```
Present in at least one of: mnemiopsis_leidyi
and in at least one of: danio_rerio,xenopus_tropicalis,gallus_gallus,homo_sapiens,ciona_intestinalis,branchiostoma_floridae,saccoglossus_kowalevskii,acanthaster_planci,pristionchus_pacificus,caenorhabditis_elegans,drosophila_melanogaster,tribolium_castaneum,daphnia_pulex,ixodes_scapularis,lottia_gigantea,octopus_bimaculoides,phoronis_australis,notospermus_geniculatus,schistosoma_mansoni,schmidtea_mediterranea,capitella_teleta,helobdella_robusta,exaiptasia_pallida,nematostella_vectensis,orbicella_faveolata,acropora_digitifera,pocillopora_damicornis,renilla_muelleri,dendronephthya_gigantea,aurelia_aurita_atlantic,aurelia_aurita_pacific,nemopilema_nomurai,hydractinia_echinata,hydractinia_symbiolongicarpus,hydra_magnipapillata,clytia_hemisphaerica,morbakka_virulenta,hofstenia_miamia,kudoa_iwatai
and lost in: clytia_hemisphaerica: 990
```


---

##### Orthogroups present in ctenophores and bilaterians but absent in Hydra


```
one_X_one_Y_none_Z("mnemiopsis_leidyi", bilateria, "hydra_magnipapillata")
```


```
Present in at least one of: mnemiopsis_leidyi
and in at least one of: danio_rerio,xenopus_tropicalis,gallus_gallus,homo_sapiens,ciona_intestinalis,branchiostoma_floridae,saccoglossus_kowalevskii,acanthaster_planci,pristionchus_pacificus,caenorhabditis_elegans,drosophila_melanogaster,tribolium_castaneum,daphnia_pulex,ixodes_scapularis,lottia_gigantea,octopus_bimaculoides,phoronis_australis,notospermus_geniculatus,schistosoma_mansoni,schmidtea_mediterranea,capitella_teleta,helobdella_robusta,exaiptasia_pallida,nematostella_vectensis,orbicella_faveolata,acropora_digitifera,pocillopora_damicornis,renilla_muelleri,dendronephthya_gigantea,aurelia_aurita_atlantic,aurelia_aurita_pacific,nemopilema_nomurai,hydractinia_echinata,hydractinia_symbiolongicarpus,hydra_magnipapillata,clytia_hemisphaerica,morbakka_virulenta,hofstenia_miamia,kudoa_iwatai
and lost in: hydra_magnipapillata: 991
```


---

#### Porifera queries

---

##### Orthogroups present in Amphimedon and at least one bilaterian but absent in cnidarians


```
one_X_one_Y_none_Z("amphimedon_queenslandica", bilateria, cnidaria)
```


```
Present in at least one of: amphimedon_queenslandica
and in at least one of: danio_rerio,xenopus_tropicalis,gallus_gallus,homo_sapiens,ciona_intestinalis,branchiostoma_floridae,saccoglossus_kowalevskii,acanthaster_planci,pristionchus_pacificus,caenorhabditis_elegans,drosophila_melanogaster,tribolium_castaneum,daphnia_pulex,ixodes_scapularis,lottia_gigantea,octopus_bimaculoides,phoronis_australis,notospermus_geniculatus,schistosoma_mansoni,schmidtea_mediterranea,capitella_teleta,helobdella_robusta,exaiptasia_pallida,nematostella_vectensis,orbicella_faveolata,acropora_digitifera,pocillopora_damicornis,renilla_muelleri,dendronephthya_gigantea,aurelia_aurita_atlantic,aurelia_aurita_pacific,nemopilema_nomurai,hydractinia_echinata,hydractinia_symbiolongicarpus,hydra_magnipapillata,clytia_hemisphaerica,morbakka_virulenta,hofstenia_miamia,kudoa_iwatai
and lost in: exaiptasia_pallida,nematostella_vectensis,orbicella_faveolata,acropora_digitifera,pocillopora_damicornis,renilla_muelleri,dendronephthya_gigantea,aurelia_aurita_atlantic,aurelia_aurita_pacific,nemopilema_nomurai,hydractinia_echinata,hydractinia_symbiolongicarpus,hydra_magnipapillata,clytia_hemisphaerica,morbakka_virulenta,kudoa_iwatai: 376
```


---

##### Orthogroups present in Amphimedon and at least one bilaterian but absent in medusozoans


```
one_X_one_Y_none_Z("amphimedon_queenslandica", bilateria, medusozoa)
```


```
Present in at least one of: amphimedon_queenslandica
and in at least one of: danio_rerio,xenopus_tropicalis,gallus_gallus,homo_sapiens,ciona_intestinalis,branchiostoma_floridae,saccoglossus_kowalevskii,acanthaster_planci,pristionchus_pacificus,caenorhabditis_elegans,drosophila_melanogaster,tribolium_castaneum,daphnia_pulex,ixodes_scapularis,lottia_gigantea,octopus_bimaculoides,phoronis_australis,notospermus_geniculatus,schistosoma_mansoni,schmidtea_mediterranea,capitella_teleta,helobdella_robusta,exaiptasia_pallida,nematostella_vectensis,orbicella_faveolata,acropora_digitifera,pocillopora_damicornis,renilla_muelleri,dendronephthya_gigantea,aurelia_aurita_atlantic,aurelia_aurita_pacific,nemopilema_nomurai,hydractinia_echinata,hydractinia_symbiolongicarpus,hydra_magnipapillata,clytia_hemisphaerica,morbakka_virulenta,hofstenia_miamia,kudoa_iwatai
and lost in: aurelia_aurita_atlantic,aurelia_aurita_pacific,nemopilema_nomurai,hydractinia_echinata,hydractinia_symbiolongicarpus,hydra_magnipapillata,clytia_hemisphaerica,morbakka_virulenta: 672
```


---

##### Orthogroups present in Amphimedon and at least one bilaterian but absent in both Hydractinia


```
one_X_one_Y_none_Z("amphimedon_queenslandica", bilateria, hydractinia)
```


```
Present in at least one of: amphimedon_queenslandica
and in at least one of: danio_rerio,xenopus_tropicalis,gallus_gallus,homo_sapiens,ciona_intestinalis,branchiostoma_floridae,saccoglossus_kowalevskii,acanthaster_planci,pristionchus_pacificus,caenorhabditis_elegans,drosophila_melanogaster,tribolium_castaneum,daphnia_pulex,ixodes_scapularis,lottia_gigantea,octopus_bimaculoides,phoronis_australis,notospermus_geniculatus,schistosoma_mansoni,schmidtea_mediterranea,capitella_teleta,helobdella_robusta,exaiptasia_pallida,nematostella_vectensis,orbicella_faveolata,acropora_digitifera,pocillopora_damicornis,renilla_muelleri,dendronephthya_gigantea,aurelia_aurita_atlantic,aurelia_aurita_pacific,nemopilema_nomurai,hydractinia_echinata,hydractinia_symbiolongicarpus,hydra_magnipapillata,clytia_hemisphaerica,morbakka_virulenta,hofstenia_miamia,kudoa_iwatai
and lost in: hydractinia_echinata,hydractinia_symbiolongicarpus: 1334
```


---

##### Orthogroups present in Amphimedon and at least one bilaterian but absent in one hydrozoan


```
for (taxon in c("hydractinia_echinata", "hydractinia_symbiolongicarpus", "clytia_hemisphaerica", "hydra_magnipapillata")){
  one_X_one_Y_none_Z("amphimedon_queenslandica", bilateria, taxon)
  cat("\n\n")
}
```


```
Present in at least one of: amphimedon_queenslandica
and in at least one of: danio_rerio,xenopus_tropicalis,gallus_gallus,homo_sapiens,ciona_intestinalis,branchiostoma_floridae,saccoglossus_kowalevskii,acanthaster_planci,pristionchus_pacificus,caenorhabditis_elegans,drosophila_melanogaster,tribolium_castaneum,daphnia_pulex,ixodes_scapularis,lottia_gigantea,octopus_bimaculoides,phoronis_australis,notospermus_geniculatus,schistosoma_mansoni,schmidtea_mediterranea,capitella_teleta,helobdella_robusta,exaiptasia_pallida,nematostella_vectensis,orbicella_faveolata,acropora_digitifera,pocillopora_damicornis,renilla_muelleri,dendronephthya_gigantea,aurelia_aurita_atlantic,aurelia_aurita_pacific,nemopilema_nomurai,hydractinia_echinata,hydractinia_symbiolongicarpus,hydra_magnipapillata,clytia_hemisphaerica,morbakka_virulenta,hofstenia_miamia,kudoa_iwatai
and lost in: hydractinia_echinata: 1526

Present in at least one of: amphimedon_queenslandica
and in at least one of: danio_rerio,xenopus_tropicalis,gallus_gallus,homo_sapiens,ciona_intestinalis,branchiostoma_floridae,saccoglossus_kowalevskii,acanthaster_planci,pristionchus_pacificus,caenorhabditis_elegans,drosophila_melanogaster,tribolium_castaneum,daphnia_pulex,ixodes_scapularis,lottia_gigantea,octopus_bimaculoides,phoronis_australis,notospermus_geniculatus,schistosoma_mansoni,schmidtea_mediterranea,capitella_teleta,helobdella_robusta,exaiptasia_pallida,nematostella_vectensis,orbicella_faveolata,acropora_digitifera,pocillopora_damicornis,renilla_muelleri,dendronephthya_gigantea,aurelia_aurita_atlantic,aurelia_aurita_pacific,nemopilema_nomurai,hydractinia_echinata,hydractinia_symbiolongicarpus,hydra_magnipapillata,clytia_hemisphaerica,morbakka_virulenta,hofstenia_miamia,kudoa_iwatai
and lost in: hydractinia_symbiolongicarpus: 1529

Present in at least one of: amphimedon_queenslandica
and in at least one of: danio_rerio,xenopus_tropicalis,gallus_gallus,homo_sapiens,ciona_intestinalis,branchiostoma_floridae,saccoglossus_kowalevskii,acanthaster_planci,pristionchus_pacificus,caenorhabditis_elegans,drosophila_melanogaster,tribolium_castaneum,daphnia_pulex,ixodes_scapularis,lottia_gigantea,octopus_bimaculoides,phoronis_australis,notospermus_geniculatus,schistosoma_mansoni,schmidtea_mediterranea,capitella_teleta,helobdella_robusta,exaiptasia_pallida,nematostella_vectensis,orbicella_faveolata,acropora_digitifera,pocillopora_damicornis,renilla_muelleri,dendronephthya_gigantea,aurelia_aurita_atlantic,aurelia_aurita_pacific,nemopilema_nomurai,hydractinia_echinata,hydractinia_symbiolongicarpus,hydra_magnipapillata,clytia_hemisphaerica,morbakka_virulenta,hofstenia_miamia,kudoa_iwatai
and lost in: clytia_hemisphaerica: 1731

Present in at least one of: amphimedon_queenslandica
and in at least one of: danio_rerio,xenopus_tropicalis,gallus_gallus,homo_sapiens,ciona_intestinalis,branchiostoma_floridae,saccoglossus_kowalevskii,acanthaster_planci,pristionchus_pacificus,caenorhabditis_elegans,drosophila_melanogaster,tribolium_castaneum,daphnia_pulex,ixodes_scapularis,lottia_gigantea,octopus_bimaculoides,phoronis_australis,notospermus_geniculatus,schistosoma_mansoni,schmidtea_mediterranea,capitella_teleta,helobdella_robusta,exaiptasia_pallida,nematostella_vectensis,orbicella_faveolata,acropora_digitifera,pocillopora_damicornis,renilla_muelleri,dendronephthya_gigantea,aurelia_aurita_atlantic,aurelia_aurita_pacific,nemopilema_nomurai,hydractinia_echinata,hydractinia_symbiolongicarpus,hydra_magnipapillata,clytia_hemisphaerica,morbakka_virulenta,hofstenia_miamia,kudoa_iwatai
and lost in: hydra_magnipapillata: 1662
```


---

#### Placozoa queries


```
placozoa <- select_clade("Placozoa")
placozoa
```


```
[1] "hoilungia_hongkongensis" "trichoplax_adhaerens"
```


---

##### Orthogroups present in at least one placozoan and one bilaterian but absent in all cnidarians


```
one_X_one_Y_none_Z(placozoa, bilateria, cnidaria)
```


```
Present in at least one of: hoilungia_hongkongensis,trichoplax_adhaerens
and in at least one of: danio_rerio,xenopus_tropicalis,gallus_gallus,homo_sapiens,ciona_intestinalis,branchiostoma_floridae,saccoglossus_kowalevskii,acanthaster_planci,pristionchus_pacificus,caenorhabditis_elegans,drosophila_melanogaster,tribolium_castaneum,daphnia_pulex,ixodes_scapularis,lottia_gigantea,octopus_bimaculoides,phoronis_australis,notospermus_geniculatus,schistosoma_mansoni,schmidtea_mediterranea,capitella_teleta,helobdella_robusta,exaiptasia_pallida,nematostella_vectensis,orbicella_faveolata,acropora_digitifera,pocillopora_damicornis,renilla_muelleri,dendronephthya_gigantea,aurelia_aurita_atlantic,aurelia_aurita_pacific,nemopilema_nomurai,hydractinia_echinata,hydractinia_symbiolongicarpus,hydra_magnipapillata,clytia_hemisphaerica,morbakka_virulenta,hofstenia_miamia,kudoa_iwatai
and lost in: exaiptasia_pallida,nematostella_vectensis,orbicella_faveolata,acropora_digitifera,pocillopora_damicornis,renilla_muelleri,dendronephthya_gigantea,aurelia_aurita_atlantic,aurelia_aurita_pacific,nemopilema_nomurai,hydractinia_echinata,hydractinia_symbiolongicarpus,hydra_magnipapillata,clytia_hemisphaerica,morbakka_virulenta,kudoa_iwatai: 171
```


---

##### Orthogroups present in at least one placozoan and one bilaterian but absent in all medusozoans


```
one_X_one_Y_none_Z(placozoa, bilateria, medusozoa)
```


```
Present in at least one of: hoilungia_hongkongensis,trichoplax_adhaerens
and in at least one of: danio_rerio,xenopus_tropicalis,gallus_gallus,homo_sapiens,ciona_intestinalis,branchiostoma_floridae,saccoglossus_kowalevskii,acanthaster_planci,pristionchus_pacificus,caenorhabditis_elegans,drosophila_melanogaster,tribolium_castaneum,daphnia_pulex,ixodes_scapularis,lottia_gigantea,octopus_bimaculoides,phoronis_australis,notospermus_geniculatus,schistosoma_mansoni,schmidtea_mediterranea,capitella_teleta,helobdella_robusta,exaiptasia_pallida,nematostella_vectensis,orbicella_faveolata,acropora_digitifera,pocillopora_damicornis,renilla_muelleri,dendronephthya_gigantea,aurelia_aurita_atlantic,aurelia_aurita_pacific,nemopilema_nomurai,hydractinia_echinata,hydractinia_symbiolongicarpus,hydra_magnipapillata,clytia_hemisphaerica,morbakka_virulenta,hofstenia_miamia,kudoa_iwatai
and lost in: aurelia_aurita_atlantic,aurelia_aurita_pacific,nemopilema_nomurai,hydractinia_echinata,hydractinia_symbiolongicarpus,hydra_magnipapillata,clytia_hemisphaerica,morbakka_virulenta: 436
```


---

##### Orthogroups present in at least one placozoan and one bilaterian but absent in all medusozoans


```
one_X_one_Y_none_Z(placozoa, bilateria, hydrozoa)
```


```
Present in at least one of: hoilungia_hongkongensis,trichoplax_adhaerens
and in at least one of: danio_rerio,xenopus_tropicalis,gallus_gallus,homo_sapiens,ciona_intestinalis,branchiostoma_floridae,saccoglossus_kowalevskii,acanthaster_planci,pristionchus_pacificus,caenorhabditis_elegans,drosophila_melanogaster,tribolium_castaneum,daphnia_pulex,ixodes_scapularis,lottia_gigantea,octopus_bimaculoides,phoronis_australis,notospermus_geniculatus,schistosoma_mansoni,schmidtea_mediterranea,capitella_teleta,helobdella_robusta,exaiptasia_pallida,nematostella_vectensis,orbicella_faveolata,acropora_digitifera,pocillopora_damicornis,renilla_muelleri,dendronephthya_gigantea,aurelia_aurita_atlantic,aurelia_aurita_pacific,nemopilema_nomurai,hydractinia_echinata,hydractinia_symbiolongicarpus,hydra_magnipapillata,clytia_hemisphaerica,morbakka_virulenta,hofstenia_miamia,kudoa_iwatai
and lost in: hydractinia_echinata,hydractinia_symbiolongicarpus,hydra_magnipapillata,clytia_hemisphaerica: 694
```


---

##### Orthogroups present in at least one placozoan and one bilaterian but absent in both Hydractinia


```
one_X_one_Y_none_Z(placozoa, bilateria, hydractinia)
```


```
Present in at least one of: hoilungia_hongkongensis,trichoplax_adhaerens
and in at least one of: danio_rerio,xenopus_tropicalis,gallus_gallus,homo_sapiens,ciona_intestinalis,branchiostoma_floridae,saccoglossus_kowalevskii,acanthaster_planci,pristionchus_pacificus,caenorhabditis_elegans,drosophila_melanogaster,tribolium_castaneum,daphnia_pulex,ixodes_scapularis,lottia_gigantea,octopus_bimaculoides,phoronis_australis,notospermus_geniculatus,schistosoma_mansoni,schmidtea_mediterranea,capitella_teleta,helobdella_robusta,exaiptasia_pallida,nematostella_vectensis,orbicella_faveolata,acropora_digitifera,pocillopora_damicornis,renilla_muelleri,dendronephthya_gigantea,aurelia_aurita_atlantic,aurelia_aurita_pacific,nemopilema_nomurai,hydractinia_echinata,hydractinia_symbiolongicarpus,hydra_magnipapillata,clytia_hemisphaerica,morbakka_virulenta,hofstenia_miamia,kudoa_iwatai
and lost in: hydractinia_echinata,hydractinia_symbiolongicarpus: 903
```


---

##### Orthogroups present in at least one placozoan and one bilaterian but absent in one hydrozoan


```
for (taxon in c("hydractinia_echinata", "hydractinia_symbiolongicarpus", "clytia_hemisphaerica", "hydra_magnipapillata")){
  one_X_one_Y_none_Z(placozoa, bilateria, taxon)
  cat("\n\n")
}
```


```
Present in at least one of: hoilungia_hongkongensis,trichoplax_adhaerens
and in at least one of: danio_rerio,xenopus_tropicalis,gallus_gallus,homo_sapiens,ciona_intestinalis,branchiostoma_floridae,saccoglossus_kowalevskii,acanthaster_planci,pristionchus_pacificus,caenorhabditis_elegans,drosophila_melanogaster,tribolium_castaneum,daphnia_pulex,ixodes_scapularis,lottia_gigantea,octopus_bimaculoides,phoronis_australis,notospermus_geniculatus,schistosoma_mansoni,schmidtea_mediterranea,capitella_teleta,helobdella_robusta,exaiptasia_pallida,nematostella_vectensis,orbicella_faveolata,acropora_digitifera,pocillopora_damicornis,renilla_muelleri,dendronephthya_gigantea,aurelia_aurita_atlantic,aurelia_aurita_pacific,nemopilema_nomurai,hydractinia_echinata,hydractinia_symbiolongicarpus,hydra_magnipapillata,clytia_hemisphaerica,morbakka_virulenta,hofstenia_miamia,kudoa_iwatai
and lost in: hydractinia_echinata: 1091

Present in at least one of: hoilungia_hongkongensis,trichoplax_adhaerens
and in at least one of: danio_rerio,xenopus_tropicalis,gallus_gallus,homo_sapiens,ciona_intestinalis,branchiostoma_floridae,saccoglossus_kowalevskii,acanthaster_planci,pristionchus_pacificus,caenorhabditis_elegans,drosophila_melanogaster,tribolium_castaneum,daphnia_pulex,ixodes_scapularis,lottia_gigantea,octopus_bimaculoides,phoronis_australis,notospermus_geniculatus,schistosoma_mansoni,schmidtea_mediterranea,capitella_teleta,helobdella_robusta,exaiptasia_pallida,nematostella_vectensis,orbicella_faveolata,acropora_digitifera,pocillopora_damicornis,renilla_muelleri,dendronephthya_gigantea,aurelia_aurita_atlantic,aurelia_aurita_pacific,nemopilema_nomurai,hydractinia_echinata,hydractinia_symbiolongicarpus,hydra_magnipapillata,clytia_hemisphaerica,morbakka_virulenta,hofstenia_miamia,kudoa_iwatai
and lost in: hydractinia_symbiolongicarpus: 1090

Present in at least one of: hoilungia_hongkongensis,trichoplax_adhaerens
and in at least one of: danio_rerio,xenopus_tropicalis,gallus_gallus,homo_sapiens,ciona_intestinalis,branchiostoma_floridae,saccoglossus_kowalevskii,acanthaster_planci,pristionchus_pacificus,caenorhabditis_elegans,drosophila_melanogaster,tribolium_castaneum,daphnia_pulex,ixodes_scapularis,lottia_gigantea,octopus_bimaculoides,phoronis_australis,notospermus_geniculatus,schistosoma_mansoni,schmidtea_mediterranea,capitella_teleta,helobdella_robusta,exaiptasia_pallida,nematostella_vectensis,orbicella_faveolata,acropora_digitifera,pocillopora_damicornis,renilla_muelleri,dendronephthya_gigantea,aurelia_aurita_atlantic,aurelia_aurita_pacific,nemopilema_nomurai,hydractinia_echinata,hydractinia_symbiolongicarpus,hydra_magnipapillata,clytia_hemisphaerica,morbakka_virulenta,hofstenia_miamia,kudoa_iwatai
and lost in: clytia_hemisphaerica: 1341

Present in at least one of: hoilungia_hongkongensis,trichoplax_adhaerens
and in at least one of: danio_rerio,xenopus_tropicalis,gallus_gallus,homo_sapiens,ciona_intestinalis,branchiostoma_floridae,saccoglossus_kowalevskii,acanthaster_planci,pristionchus_pacificus,caenorhabditis_elegans,drosophila_melanogaster,tribolium_castaneum,daphnia_pulex,ixodes_scapularis,lottia_gigantea,octopus_bimaculoides,phoronis_australis,notospermus_geniculatus,schistosoma_mansoni,schmidtea_mediterranea,capitella_teleta,helobdella_robusta,exaiptasia_pallida,nematostella_vectensis,orbicella_faveolata,acropora_digitifera,pocillopora_damicornis,renilla_muelleri,dendronephthya_gigantea,aurelia_aurita_atlantic,aurelia_aurita_pacific,nemopilema_nomurai,hydractinia_echinata,hydractinia_symbiolongicarpus,hydra_magnipapillata,clytia_hemisphaerica,morbakka_virulenta,hofstenia_miamia,kudoa_iwatai
and lost in: hydra_magnipapillata: 1269
```


---

#### Potential losses since split with Ctenoophora + sponge + Placozoa (CSP)


```
csp <- fuse_taxa(c("Ctenophora", "Porifera", "Placozoa"))
csp
```


```
[1] "hoilungia_hongkongensis"  "trichoplax_adhaerens"     "amphimedon_queenslandica" "mnemiopsis_leidyi"
```


---

##### Orthogroups present in at least one of CSP and one bilaterian but absent in all cnidarians


```
one_X_one_Y_none_Z(csp, bilateria, cnidaria)
```


```
Present in at least one of: hoilungia_hongkongensis,trichoplax_adhaerens,amphimedon_queenslandica,mnemiopsis_leidyi
and in at least one of: danio_rerio,xenopus_tropicalis,gallus_gallus,homo_sapiens,ciona_intestinalis,branchiostoma_floridae,saccoglossus_kowalevskii,acanthaster_planci,pristionchus_pacificus,caenorhabditis_elegans,drosophila_melanogaster,tribolium_castaneum,daphnia_pulex,ixodes_scapularis,lottia_gigantea,octopus_bimaculoides,phoronis_australis,notospermus_geniculatus,schistosoma_mansoni,schmidtea_mediterranea,capitella_teleta,helobdella_robusta,exaiptasia_pallida,nematostella_vectensis,orbicella_faveolata,acropora_digitifera,pocillopora_damicornis,renilla_muelleri,dendronephthya_gigantea,aurelia_aurita_atlantic,aurelia_aurita_pacific,nemopilema_nomurai,hydractinia_echinata,hydractinia_symbiolongicarpus,hydra_magnipapillata,clytia_hemisphaerica,morbakka_virulenta,hofstenia_miamia,kudoa_iwatai
and lost in: exaiptasia_pallida,nematostella_vectensis,orbicella_faveolata,acropora_digitifera,pocillopora_damicornis,renilla_muelleri,dendronephthya_gigantea,aurelia_aurita_atlantic,aurelia_aurita_pacific,nemopilema_nomurai,hydractinia_echinata,hydractinia_symbiolongicarpus,hydra_magnipapillata,clytia_hemisphaerica,morbakka_virulenta,kudoa_iwatai: 639
```


---

##### Orthogroups present in at least one of CSP and one bilaterian but absent in all medusozoans


```
one_X_one_Y_none_Z(csp, bilateria, medusozoa)
```


```
Present in at least one of: hoilungia_hongkongensis,trichoplax_adhaerens,amphimedon_queenslandica,mnemiopsis_leidyi
and in at least one of: danio_rerio,xenopus_tropicalis,gallus_gallus,homo_sapiens,ciona_intestinalis,branchiostoma_floridae,saccoglossus_kowalevskii,acanthaster_planci,pristionchus_pacificus,caenorhabditis_elegans,drosophila_melanogaster,tribolium_castaneum,daphnia_pulex,ixodes_scapularis,lottia_gigantea,octopus_bimaculoides,phoronis_australis,notospermus_geniculatus,schistosoma_mansoni,schmidtea_mediterranea,capitella_teleta,helobdella_robusta,exaiptasia_pallida,nematostella_vectensis,orbicella_faveolata,acropora_digitifera,pocillopora_damicornis,renilla_muelleri,dendronephthya_gigantea,aurelia_aurita_atlantic,aurelia_aurita_pacific,nemopilema_nomurai,hydractinia_echinata,hydractinia_symbiolongicarpus,hydra_magnipapillata,clytia_hemisphaerica,morbakka_virulenta,hofstenia_miamia,kudoa_iwatai
and lost in: aurelia_aurita_atlantic,aurelia_aurita_pacific,nemopilema_nomurai,hydractinia_echinata,hydractinia_symbiolongicarpus,hydra_magnipapillata,clytia_hemisphaerica,morbakka_virulenta: 1214
```


---

##### Orthogroups present in at least one of CSP and one bilaterian but absent in all hydrozoans


```
one_X_one_Y_none_Z(csp, bilateria, hydrozoa)
```


```
Present in at least one of: hoilungia_hongkongensis,trichoplax_adhaerens,amphimedon_queenslandica,mnemiopsis_leidyi
and in at least one of: danio_rerio,xenopus_tropicalis,gallus_gallus,homo_sapiens,ciona_intestinalis,branchiostoma_floridae,saccoglossus_kowalevskii,acanthaster_planci,pristionchus_pacificus,caenorhabditis_elegans,drosophila_melanogaster,tribolium_castaneum,daphnia_pulex,ixodes_scapularis,lottia_gigantea,octopus_bimaculoides,phoronis_australis,notospermus_geniculatus,schistosoma_mansoni,schmidtea_mediterranea,capitella_teleta,helobdella_robusta,exaiptasia_pallida,nematostella_vectensis,orbicella_faveolata,acropora_digitifera,pocillopora_damicornis,renilla_muelleri,dendronephthya_gigantea,aurelia_aurita_atlantic,aurelia_aurita_pacific,nemopilema_nomurai,hydractinia_echinata,hydractinia_symbiolongicarpus,hydra_magnipapillata,clytia_hemisphaerica,morbakka_virulenta,hofstenia_miamia,kudoa_iwatai
and lost in: hydractinia_echinata,hydractinia_symbiolongicarpus,hydra_magnipapillata,clytia_hemisphaerica: 1749
```


---

##### Orthogroups present in at least one of CSP and one bilaterian but absent in both hydractinia


```
one_X_one_Y_none_Z(csp, bilateria, hydractinia)
```


```
Present in at least one of: hoilungia_hongkongensis,trichoplax_adhaerens,amphimedon_queenslandica,mnemiopsis_leidyi
and in at least one of: danio_rerio,xenopus_tropicalis,gallus_gallus,homo_sapiens,ciona_intestinalis,branchiostoma_floridae,saccoglossus_kowalevskii,acanthaster_planci,pristionchus_pacificus,caenorhabditis_elegans,drosophila_melanogaster,tribolium_castaneum,daphnia_pulex,ixodes_scapularis,lottia_gigantea,octopus_bimaculoides,phoronis_australis,notospermus_geniculatus,schistosoma_mansoni,schmidtea_mediterranea,capitella_teleta,helobdella_robusta,exaiptasia_pallida,nematostella_vectensis,orbicella_faveolata,acropora_digitifera,pocillopora_damicornis,renilla_muelleri,dendronephthya_gigantea,aurelia_aurita_atlantic,aurelia_aurita_pacific,nemopilema_nomurai,hydractinia_echinata,hydractinia_symbiolongicarpus,hydra_magnipapillata,clytia_hemisphaerica,morbakka_virulenta,hofstenia_miamia,kudoa_iwatai
and lost in: hydractinia_echinata,hydractinia_symbiolongicarpus: 2269
```

##### Orthogroups present in at least one of CSP and one bilaterian but absent in one hydrozoan


```
for (taxon in c("hydractinia_echinata", "hydractinia_symbiolongicarpus", "clytia_hemisphaerica", "hydra_magnipapillata")){
  one_X_one_Y_none_Z(csp, bilateria, taxon)
  cat("\n\n")
}
```


```
Present in at least one of: hoilungia_hongkongensis,trichoplax_adhaerens,amphimedon_queenslandica,mnemiopsis_leidyi
and in at least one of: danio_rerio,xenopus_tropicalis,gallus_gallus,homo_sapiens,ciona_intestinalis,branchiostoma_floridae,saccoglossus_kowalevskii,acanthaster_planci,pristionchus_pacificus,caenorhabditis_elegans,drosophila_melanogaster,tribolium_castaneum,daphnia_pulex,ixodes_scapularis,lottia_gigantea,octopus_bimaculoides,phoronis_australis,notospermus_geniculatus,schistosoma_mansoni,schmidtea_mediterranea,capitella_teleta,helobdella_robusta,exaiptasia_pallida,nematostella_vectensis,orbicella_faveolata,acropora_digitifera,pocillopora_damicornis,renilla_muelleri,dendronephthya_gigantea,aurelia_aurita_atlantic,aurelia_aurita_pacific,nemopilema_nomurai,hydractinia_echinata,hydractinia_symbiolongicarpus,hydra_magnipapillata,clytia_hemisphaerica,morbakka_virulenta,hofstenia_miamia,kudoa_iwatai
and lost in: hydractinia_echinata: 2516

Present in at least one of: hoilungia_hongkongensis,trichoplax_adhaerens,amphimedon_queenslandica,mnemiopsis_leidyi
and in at least one of: danio_rerio,xenopus_tropicalis,gallus_gallus,homo_sapiens,ciona_intestinalis,branchiostoma_floridae,saccoglossus_kowalevskii,acanthaster_planci,pristionchus_pacificus,caenorhabditis_elegans,drosophila_melanogaster,tribolium_castaneum,daphnia_pulex,ixodes_scapularis,lottia_gigantea,octopus_bimaculoides,phoronis_australis,notospermus_geniculatus,schistosoma_mansoni,schmidtea_mediterranea,capitella_teleta,helobdella_robusta,exaiptasia_pallida,nematostella_vectensis,orbicella_faveolata,acropora_digitifera,pocillopora_damicornis,renilla_muelleri,dendronephthya_gigantea,aurelia_aurita_atlantic,aurelia_aurita_pacific,nemopilema_nomurai,hydractinia_echinata,hydractinia_symbiolongicarpus,hydra_magnipapillata,clytia_hemisphaerica,morbakka_virulenta,hofstenia_miamia,kudoa_iwatai
and lost in: hydractinia_symbiolongicarpus: 2534

Present in at least one of: hoilungia_hongkongensis,trichoplax_adhaerens,amphimedon_queenslandica,mnemiopsis_leidyi
and in at least one of: danio_rerio,xenopus_tropicalis,gallus_gallus,homo_sapiens,ciona_intestinalis,branchiostoma_floridae,saccoglossus_kowalevskii,acanthaster_planci,pristionchus_pacificus,caenorhabditis_elegans,drosophila_melanogaster,tribolium_castaneum,daphnia_pulex,ixodes_scapularis,lottia_gigantea,octopus_bimaculoides,phoronis_australis,notospermus_geniculatus,schistosoma_mansoni,schmidtea_mediterranea,capitella_teleta,helobdella_robusta,exaiptasia_pallida,nematostella_vectensis,orbicella_faveolata,acropora_digitifera,pocillopora_damicornis,renilla_muelleri,dendronephthya_gigantea,aurelia_aurita_atlantic,aurelia_aurita_pacific,nemopilema_nomurai,hydractinia_echinata,hydractinia_symbiolongicarpus,hydra_magnipapillata,clytia_hemisphaerica,morbakka_virulenta,hofstenia_miamia,kudoa_iwatai
and lost in: clytia_hemisphaerica: 2721

Present in at least one of: hoilungia_hongkongensis,trichoplax_adhaerens,amphimedon_queenslandica,mnemiopsis_leidyi
and in at least one of: danio_rerio,xenopus_tropicalis,gallus_gallus,homo_sapiens,ciona_intestinalis,branchiostoma_floridae,saccoglossus_kowalevskii,acanthaster_planci,pristionchus_pacificus,caenorhabditis_elegans,drosophila_melanogaster,tribolium_castaneum,daphnia_pulex,ixodes_scapularis,lottia_gigantea,octopus_bimaculoides,phoronis_australis,notospermus_geniculatus,schistosoma_mansoni,schmidtea_mediterranea,capitella_teleta,helobdella_robusta,exaiptasia_pallida,nematostella_vectensis,orbicella_faveolata,acropora_digitifera,pocillopora_damicornis,renilla_muelleri,dendronephthya_gigantea,aurelia_aurita_atlantic,aurelia_aurita_pacific,nemopilema_nomurai,hydractinia_echinata,hydractinia_symbiolongicarpus,hydra_magnipapillata,clytia_hemisphaerica,morbakka_virulenta,hofstenia_miamia,kudoa_iwatai
and lost in: hydra_magnipapillata: 2733
```


---

##### Orthogroups present in at least one of CSP but absent in all cnidarians


```
one_X_none_Y(csp, cnidaria)
```


```
Present in at least one of: hoilungia_hongkongensis,trichoplax_adhaerens,amphimedon_queenslandica,mnemiopsis_leidyi
and in none of: exaiptasia_pallida,nematostella_vectensis,orbicella_faveolata,acropora_digitifera,pocillopora_damicornis,renilla_muelleri,dendronephthya_gigantea,aurelia_aurita_atlantic,aurelia_aurita_pacific,nemopilema_nomurai,hydractinia_echinata,hydractinia_symbiolongicarpus,hydra_magnipapillata,clytia_hemisphaerica,morbakka_virulenta,kudoa_iwatai: 2103
```


---

##### Orthogroups present in at least one of CSP but absent in all medusozoans


```
one_X_none_Y(csp, medusozoa)
```


```
Present in at least one of: hoilungia_hongkongensis,trichoplax_adhaerens,amphimedon_queenslandica,mnemiopsis_leidyi
and in none of: aurelia_aurita_atlantic,aurelia_aurita_pacific,nemopilema_nomurai,hydractinia_echinata,hydractinia_symbiolongicarpus,hydra_magnipapillata,clytia_hemisphaerica,morbakka_virulenta: 2678
```


---

##### Orthogroups present in at least one of CSP but absent in all hydrozoans


```
one_X_none_Y(csp, hydrozoa)
```


```
Present in at least one of: hoilungia_hongkongensis,trichoplax_adhaerens,amphimedon_queenslandica,mnemiopsis_leidyi
and in none of: hydractinia_echinata,hydractinia_symbiolongicarpus,hydra_magnipapillata,clytia_hemisphaerica: 3213
```


---

##### Orthogroups present in at least one of CSP but absent in both Hydractinia


```
one_X_none_Y(csp, hydractinia)
```


```
Present in at least one of: hoilungia_hongkongensis,trichoplax_adhaerens,amphimedon_queenslandica,mnemiopsis_leidyi
and in none of: hydractinia_echinata,hydractinia_symbiolongicarpus: 3733
```

##### Orthogroups present in at least one of CSP but absent in one hydrozoan


```
for (taxon in c("hydractinia_echinata", "hydractinia_symbiolongicarpus", "hydra_magnipapillata", "clytia_hemisphaerica")){
  one_X_none_Y(csp, taxon)
  cat("\n\n")
}
```


```
Present in at least one of: hoilungia_hongkongensis,trichoplax_adhaerens,amphimedon_queenslandica,mnemiopsis_leidyi
and in none of: hydractinia_echinata: 3980

Present in at least one of: hoilungia_hongkongensis,trichoplax_adhaerens,amphimedon_queenslandica,mnemiopsis_leidyi
and in none of: hydractinia_symbiolongicarpus: 3998

Present in at least one of: hoilungia_hongkongensis,trichoplax_adhaerens,amphimedon_queenslandica,mnemiopsis_leidyi
and in none of: hydra_magnipapillata: 4197

Present in at least one of: hoilungia_hongkongensis,trichoplax_adhaerens,amphimedon_queenslandica,mnemiopsis_leidyi
and in none of: clytia_hemisphaerica: 4185
```


---

#### Potential losses since split between (non-metazoans + Ctenophora + Porifera + Placozoa) and (Cnidaria+Bilateria)


```
ancestor <- fuse_taxa(c("Non-metazoans","Ctenophora", "Porifera", "Placozoa"))
ancestor
```


```
 [1] "hoilungia_hongkongensis"  "trichoplax_adhaerens"     "amphimedon_queenslandica" "mnemiopsis_leidyi"        "capsaspora_owczarzaki"   
 [6] "creolimax_fragrantissima" "monosiga_brevicolis"      "saccharomyces_cerevisiae" "salpingoeca_rosetta"      "sphaeroforma_arctica"
```


---

##### Orthogroups present in at least one early-branching taxon and absent in all cnidarians


```
one_X_none_Y(ancestor, cnidaria)
```


```
Present in at least one of: hoilungia_hongkongensis,trichoplax_adhaerens,amphimedon_queenslandica,mnemiopsis_leidyi,capsaspora_owczarzaki,creolimax_fragrantissima,monosiga_brevicolis,saccharomyces_cerevisiae,salpingoeca_rosetta,sphaeroforma_arctica
and in none of: exaiptasia_pallida,nematostella_vectensis,orbicella_faveolata,acropora_digitifera,pocillopora_damicornis,renilla_muelleri,dendronephthya_gigantea,aurelia_aurita_atlantic,aurelia_aurita_pacific,nemopilema_nomurai,hydractinia_echinata,hydractinia_symbiolongicarpus,hydra_magnipapillata,clytia_hemisphaerica,morbakka_virulenta,kudoa_iwatai: 5135
```


---

##### Orthogroups present in at least one early-branching taxon and at least one cnidarian but absent in H. echinata


```
one_X_one_Y_none_Z(ancestor, cnidaria, "hydractinia_echinata")
```


```
Present in at least one of: hoilungia_hongkongensis,trichoplax_adhaerens,amphimedon_queenslandica,mnemiopsis_leidyi,capsaspora_owczarzaki,creolimax_fragrantissima,monosiga_brevicolis,saccharomyces_cerevisiae,salpingoeca_rosetta,sphaeroforma_arctica
and in at least one of: exaiptasia_pallida,nematostella_vectensis,orbicella_faveolata,acropora_digitifera,pocillopora_damicornis,renilla_muelleri,dendronephthya_gigantea,aurelia_aurita_atlantic,aurelia_aurita_pacific,nemopilema_nomurai,hydractinia_echinata,hydractinia_symbiolongicarpus,hydra_magnipapillata,clytia_hemisphaerica,morbakka_virulenta,kudoa_iwatai
and lost in: hydractinia_echinata: 2325
```


---

##### Orthogroups present in at least one early-branching taxon and at least one cnidarian but absent in H. symbioongicarpus


```
one_X_one_Y_none_Z(ancestor, cnidaria, "hydractinia_symbiolongicarpus")
```


```
Present in at least one of: hoilungia_hongkongensis,trichoplax_adhaerens,amphimedon_queenslandica,mnemiopsis_leidyi,capsaspora_owczarzaki,creolimax_fragrantissima,monosiga_brevicolis,saccharomyces_cerevisiae,salpingoeca_rosetta,sphaeroforma_arctica
and in at least one of: exaiptasia_pallida,nematostella_vectensis,orbicella_faveolata,acropora_digitifera,pocillopora_damicornis,renilla_muelleri,dendronephthya_gigantea,aurelia_aurita_atlantic,aurelia_aurita_pacific,nemopilema_nomurai,hydractinia_echinata,hydractinia_symbiolongicarpus,hydra_magnipapillata,clytia_hemisphaerica,morbakka_virulenta,kudoa_iwatai
and lost in: hydractinia_symbiolongicarpus: 2362
```


---

##### Orthogroups present in at least one early-branching taxon and at least one cnidarian but absent in Hydra


```
one_X_one_Y_none_Z(ancestor, cnidaria, "hydra_magnipapillata")
```


```
Present in at least one of: hoilungia_hongkongensis,trichoplax_adhaerens,amphimedon_queenslandica,mnemiopsis_leidyi,capsaspora_owczarzaki,creolimax_fragrantissima,monosiga_brevicolis,saccharomyces_cerevisiae,salpingoeca_rosetta,sphaeroforma_arctica
and in at least one of: exaiptasia_pallida,nematostella_vectensis,orbicella_faveolata,acropora_digitifera,pocillopora_damicornis,renilla_muelleri,dendronephthya_gigantea,aurelia_aurita_atlantic,aurelia_aurita_pacific,nemopilema_nomurai,hydractinia_echinata,hydractinia_symbiolongicarpus,hydra_magnipapillata,clytia_hemisphaerica,morbakka_virulenta,kudoa_iwatai
and lost in: hydra_magnipapillata: 2579
```


---

##### Orthogroups present in at least one early-branching taxon and at least one cnidarian but absent in both Hydractinia


```
one_X_one_Y_none_Z(ancestor, cnidaria, hydractinia)
```


```
Present in at least one of: hoilungia_hongkongensis,trichoplax_adhaerens,amphimedon_queenslandica,mnemiopsis_leidyi,capsaspora_owczarzaki,creolimax_fragrantissima,monosiga_brevicolis,saccharomyces_cerevisiae,salpingoeca_rosetta,sphaeroforma_arctica
and in at least one of: exaiptasia_pallida,nematostella_vectensis,orbicella_faveolata,acropora_digitifera,pocillopora_damicornis,renilla_muelleri,dendronephthya_gigantea,aurelia_aurita_atlantic,aurelia_aurita_pacific,nemopilema_nomurai,hydractinia_echinata,hydractinia_symbiolongicarpus,hydra_magnipapillata,clytia_hemisphaerica,morbakka_virulenta,kudoa_iwatai
and lost in: hydractinia_echinata,hydractinia_symbiolongicarpus: 2067
```


---

##### Orthogroups present in at least one early-branching taxon and at least one cnidarian but absent in Clytia


```
one_X_one_Y_none_Z(ancestor, cnidaria, "clytia_hemisphaerica")
```


```
Present in at least one of: hoilungia_hongkongensis,trichoplax_adhaerens,amphimedon_queenslandica,mnemiopsis_leidyi,capsaspora_owczarzaki,creolimax_fragrantissima,monosiga_brevicolis,saccharomyces_cerevisiae,salpingoeca_rosetta,sphaeroforma_arctica
and in at least one of: exaiptasia_pallida,nematostella_vectensis,orbicella_faveolata,acropora_digitifera,pocillopora_damicornis,renilla_muelleri,dendronephthya_gigantea,aurelia_aurita_atlantic,aurelia_aurita_pacific,nemopilema_nomurai,hydractinia_echinata,hydractinia_symbiolongicarpus,hydra_magnipapillata,clytia_hemisphaerica,morbakka_virulenta,kudoa_iwatai
and lost in: clytia_hemisphaerica: 2552
```


---

##### Orthogroups present in at least one early-branching taxon and Hydra but absent in both Hydractinia


```
one_X_one_Y_none_Z(ancestor, "hydra_magnipapillata", hydractinia)
```


```
Present in at least one of: hoilungia_hongkongensis,trichoplax_adhaerens,amphimedon_queenslandica,mnemiopsis_leidyi,capsaspora_owczarzaki,creolimax_fragrantissima,monosiga_brevicolis,saccharomyces_cerevisiae,salpingoeca_rosetta,sphaeroforma_arctica
and in at least one of: hydra_magnipapillata
and lost in: hydractinia_echinata,hydractinia_symbiolongicarpus: 384
```

##### Orthogroups present in at least one early-branching taxon and one Hydractinia but absent in Hydra


```
one_X_one_Y_none_Z(ancestor, hydractinia, "hydra_magnipapillata")
```


```
Present in at least one of: hoilungia_hongkongensis,trichoplax_adhaerens,amphimedon_queenslandica,mnemiopsis_leidyi,capsaspora_owczarzaki,creolimax_fragrantissima,monosiga_brevicolis,saccharomyces_cerevisiae,salpingoeca_rosetta,sphaeroforma_arctica
and in at least one of: hydractinia_echinata,hydractinia_symbiolongicarpus
and lost in: hydra_magnipapillata: 896
```


---

##### Orthogroups present in at least one early-branching taxon and one bilaterian but absent in all cnidarians


```
one_X_one_Y_none_Z(ancestor, bilateria, cnidaria)
```


```
Present in at least one of: hoilungia_hongkongensis,trichoplax_adhaerens,amphimedon_queenslandica,mnemiopsis_leidyi,capsaspora_owczarzaki,creolimax_fragrantissima,monosiga_brevicolis,saccharomyces_cerevisiae,salpingoeca_rosetta,sphaeroforma_arctica
and in at least one of: danio_rerio,xenopus_tropicalis,gallus_gallus,homo_sapiens,ciona_intestinalis,branchiostoma_floridae,saccoglossus_kowalevskii,acanthaster_planci,pristionchus_pacificus,caenorhabditis_elegans,drosophila_melanogaster,tribolium_castaneum,daphnia_pulex,ixodes_scapularis,lottia_gigantea,octopus_bimaculoides,phoronis_australis,notospermus_geniculatus,schistosoma_mansoni,schmidtea_mediterranea,capitella_teleta,helobdella_robusta,exaiptasia_pallida,nematostella_vectensis,orbicella_faveolata,acropora_digitifera,pocillopora_damicornis,renilla_muelleri,dendronephthya_gigantea,aurelia_aurita_atlantic,aurelia_aurita_pacific,nemopilema_nomurai,hydractinia_echinata,hydractinia_symbiolongicarpus,hydra_magnipapillata,clytia_hemisphaerica,morbakka_virulenta,hofstenia_miamia,kudoa_iwatai
and lost in: exaiptasia_pallida,nematostella_vectensis,orbicella_faveolata,acropora_digitifera,pocillopora_damicornis,renilla_muelleri,dendronephthya_gigantea,aurelia_aurita_atlantic,aurelia_aurita_pacific,nemopilema_nomurai,hydractinia_echinata,hydractinia_symbiolongicarpus,hydra_magnipapillata,clytia_hemisphaerica,morbakka_virulenta,kudoa_iwatai: 923
```


---

##### Orthogroups present in at least one early-branching taxon and one bilaterian but absent in both Hydractinia


```
one_X_one_Y_lost_one_Z(ancestor, bilateria, hydractinia)
```


```
Present in at least one of: hoilungia_hongkongensis,trichoplax_adhaerens,amphimedon_queenslandica,mnemiopsis_leidyi,capsaspora_owczarzaki,creolimax_fragrantissima,monosiga_brevicolis,saccharomyces_cerevisiae,salpingoeca_rosetta,sphaeroforma_arctica
and in at least one of: danio_rerio,xenopus_tropicalis,gallus_gallus,homo_sapiens,ciona_intestinalis,branchiostoma_floridae,saccoglossus_kowalevskii,acanthaster_planci,pristionchus_pacificus,caenorhabditis_elegans,drosophila_melanogaster,tribolium_castaneum,daphnia_pulex,ixodes_scapularis,lottia_gigantea,octopus_bimaculoides,phoronis_australis,notospermus_geniculatus,schistosoma_mansoni,schmidtea_mediterranea,capitella_teleta,helobdella_robusta,exaiptasia_pallida,nematostella_vectensis,orbicella_faveolata,acropora_digitifera,pocillopora_damicornis,renilla_muelleri,dendronephthya_gigantea,aurelia_aurita_atlantic,aurelia_aurita_pacific,nemopilema_nomurai,hydractinia_echinata,hydractinia_symbiolongicarpus,hydra_magnipapillata,clytia_hemisphaerica,morbakka_virulenta,hofstenia_miamia,kudoa_iwatai
and lost in at least one of: hydractinia_echinata,hydractinia_symbiolongicarpus: 3543
```


---

##### Orthogroups present in at least one early-branching taxon and one bilaterian but absent in one hydrozoan


```
for (species in hydrozoa){
  one_X_one_Y_none_Z(ancestor, bilateria, species)
  cat("\n\n")
}
```


```
Present in at least one of: hoilungia_hongkongensis,trichoplax_adhaerens,amphimedon_queenslandica,mnemiopsis_leidyi,capsaspora_owczarzaki,creolimax_fragrantissima,monosiga_brevicolis,saccharomyces_cerevisiae,salpingoeca_rosetta,sphaeroforma_arctica
and in at least one of: danio_rerio,xenopus_tropicalis,gallus_gallus,homo_sapiens,ciona_intestinalis,branchiostoma_floridae,saccoglossus_kowalevskii,acanthaster_planci,pristionchus_pacificus,caenorhabditis_elegans,drosophila_melanogaster,tribolium_castaneum,daphnia_pulex,ixodes_scapularis,lottia_gigantea,octopus_bimaculoides,phoronis_australis,notospermus_geniculatus,schistosoma_mansoni,schmidtea_mediterranea,capitella_teleta,helobdella_robusta,exaiptasia_pallida,nematostella_vectensis,orbicella_faveolata,acropora_digitifera,pocillopora_damicornis,renilla_muelleri,dendronephthya_gigantea,aurelia_aurita_atlantic,aurelia_aurita_pacific,nemopilema_nomurai,hydractinia_echinata,hydractinia_symbiolongicarpus,hydra_magnipapillata,clytia_hemisphaerica,morbakka_virulenta,hofstenia_miamia,kudoa_iwatai
and lost in: hydractinia_echinata: 3248

Present in at least one of: hoilungia_hongkongensis,trichoplax_adhaerens,amphimedon_queenslandica,mnemiopsis_leidyi,capsaspora_owczarzaki,creolimax_fragrantissima,monosiga_brevicolis,saccharomyces_cerevisiae,salpingoeca_rosetta,sphaeroforma_arctica
and in at least one of: danio_rerio,xenopus_tropicalis,gallus_gallus,homo_sapiens,ciona_intestinalis,branchiostoma_floridae,saccoglossus_kowalevskii,acanthaster_planci,pristionchus_pacificus,caenorhabditis_elegans,drosophila_melanogaster,tribolium_castaneum,daphnia_pulex,ixodes_scapularis,lottia_gigantea,octopus_bimaculoides,phoronis_australis,notospermus_geniculatus,schistosoma_mansoni,schmidtea_mediterranea,capitella_teleta,helobdella_robusta,exaiptasia_pallida,nematostella_vectensis,orbicella_faveolata,acropora_digitifera,pocillopora_damicornis,renilla_muelleri,dendronephthya_gigantea,aurelia_aurita_atlantic,aurelia_aurita_pacific,nemopilema_nomurai,hydractinia_echinata,hydractinia_symbiolongicarpus,hydra_magnipapillata,clytia_hemisphaerica,morbakka_virulenta,hofstenia_miamia,kudoa_iwatai
and lost in: hydractinia_symbiolongicarpus: 3285

Present in at least one of: hoilungia_hongkongensis,trichoplax_adhaerens,amphimedon_queenslandica,mnemiopsis_leidyi,capsaspora_owczarzaki,creolimax_fragrantissima,monosiga_brevicolis,saccharomyces_cerevisiae,salpingoeca_rosetta,sphaeroforma_arctica
and in at least one of: danio_rerio,xenopus_tropicalis,gallus_gallus,homo_sapiens,ciona_intestinalis,branchiostoma_floridae,saccoglossus_kowalevskii,acanthaster_planci,pristionchus_pacificus,caenorhabditis_elegans,drosophila_melanogaster,tribolium_castaneum,daphnia_pulex,ixodes_scapularis,lottia_gigantea,octopus_bimaculoides,phoronis_australis,notospermus_geniculatus,schistosoma_mansoni,schmidtea_mediterranea,capitella_teleta,helobdella_robusta,exaiptasia_pallida,nematostella_vectensis,orbicella_faveolata,acropora_digitifera,pocillopora_damicornis,renilla_muelleri,dendronephthya_gigantea,aurelia_aurita_atlantic,aurelia_aurita_pacific,nemopilema_nomurai,hydractinia_echinata,hydractinia_symbiolongicarpus,hydra_magnipapillata,clytia_hemisphaerica,morbakka_virulenta,hofstenia_miamia,kudoa_iwatai
and lost in: hydra_magnipapillata: 3502

Present in at least one of: hoilungia_hongkongensis,trichoplax_adhaerens,amphimedon_queenslandica,mnemiopsis_leidyi,capsaspora_owczarzaki,creolimax_fragrantissima,monosiga_brevicolis,saccharomyces_cerevisiae,salpingoeca_rosetta,sphaeroforma_arctica
and in at least one of: danio_rerio,xenopus_tropicalis,gallus_gallus,homo_sapiens,ciona_intestinalis,branchiostoma_floridae,saccoglossus_kowalevskii,acanthaster_planci,pristionchus_pacificus,caenorhabditis_elegans,drosophila_melanogaster,tribolium_castaneum,daphnia_pulex,ixodes_scapularis,lottia_gigantea,octopus_bimaculoides,phoronis_australis,notospermus_geniculatus,schistosoma_mansoni,schmidtea_mediterranea,capitella_teleta,helobdella_robusta,exaiptasia_pallida,nematostella_vectensis,orbicella_faveolata,acropora_digitifera,pocillopora_damicornis,renilla_muelleri,dendronephthya_gigantea,aurelia_aurita_atlantic,aurelia_aurita_pacific,nemopilema_nomurai,hydractinia_echinata,hydractinia_symbiolongicarpus,hydra_magnipapillata,clytia_hemisphaerica,morbakka_virulenta,hofstenia_miamia,kudoa_iwatai
and lost in: clytia_hemisphaerica: 3475
```


---

##### Orthogroups present in at least one early-branching taxon and one bilaterian but absent in all hydrozoans


```
one_X_one_Y_none_Z(ancestor, bilateria, hydrozoa)
```


```
Present in at least one of: hoilungia_hongkongensis,trichoplax_adhaerens,amphimedon_queenslandica,mnemiopsis_leidyi,capsaspora_owczarzaki,creolimax_fragrantissima,monosiga_brevicolis,saccharomyces_cerevisiae,salpingoeca_rosetta,sphaeroforma_arctica
and in at least one of: danio_rerio,xenopus_tropicalis,gallus_gallus,homo_sapiens,ciona_intestinalis,branchiostoma_floridae,saccoglossus_kowalevskii,acanthaster_planci,pristionchus_pacificus,caenorhabditis_elegans,drosophila_melanogaster,tribolium_castaneum,daphnia_pulex,ixodes_scapularis,lottia_gigantea,octopus_bimaculoides,phoronis_australis,notospermus_geniculatus,schistosoma_mansoni,schmidtea_mediterranea,capitella_teleta,helobdella_robusta,exaiptasia_pallida,nematostella_vectensis,orbicella_faveolata,acropora_digitifera,pocillopora_damicornis,renilla_muelleri,dendronephthya_gigantea,aurelia_aurita_atlantic,aurelia_aurita_pacific,nemopilema_nomurai,hydractinia_echinata,hydractinia_symbiolongicarpus,hydra_magnipapillata,clytia_hemisphaerica,morbakka_virulenta,hofstenia_miamia,kudoa_iwatai
and lost in: hydractinia_echinata,hydractinia_symbiolongicarpus,hydra_magnipapillata,clytia_hemisphaerica: 2398
```


---

##### Orthogroups present in at least one early-branching taxon and one bilaterian but absent in all medusozoans


```
one_X_one_Y_none_Z(ancestor, bilateria, medusozoa)
```


```
Present in at least one of: hoilungia_hongkongensis,trichoplax_adhaerens,amphimedon_queenslandica,mnemiopsis_leidyi,capsaspora_owczarzaki,creolimax_fragrantissima,monosiga_brevicolis,saccharomyces_cerevisiae,salpingoeca_rosetta,sphaeroforma_arctica
and in at least one of: danio_rerio,xenopus_tropicalis,gallus_gallus,homo_sapiens,ciona_intestinalis,branchiostoma_floridae,saccoglossus_kowalevskii,acanthaster_planci,pristionchus_pacificus,caenorhabditis_elegans,drosophila_melanogaster,tribolium_castaneum,daphnia_pulex,ixodes_scapularis,lottia_gigantea,octopus_bimaculoides,phoronis_australis,notospermus_geniculatus,schistosoma_mansoni,schmidtea_mediterranea,capitella_teleta,helobdella_robusta,exaiptasia_pallida,nematostella_vectensis,orbicella_faveolata,acropora_digitifera,pocillopora_damicornis,renilla_muelleri,dendronephthya_gigantea,aurelia_aurita_atlantic,aurelia_aurita_pacific,nemopilema_nomurai,hydractinia_echinata,hydractinia_symbiolongicarpus,hydra_magnipapillata,clytia_hemisphaerica,morbakka_virulenta,hofstenia_miamia,kudoa_iwatai
and lost in: aurelia_aurita_atlantic,aurelia_aurita_pacific,nemopilema_nomurai,hydractinia_echinata,hydractinia_symbiolongicarpus,hydra_magnipapillata,clytia_hemisphaerica,morbakka_virulenta: 1727
```


---

##### Orthogroups present in at least one early-branching taxon and one bilaterian but absent in Nematostella


```
one_X_one_Y_none_Z(ancestor, bilateria, "nematostella_vectensis")
```


```
Present in at least one of: hoilungia_hongkongensis,trichoplax_adhaerens,amphimedon_queenslandica,mnemiopsis_leidyi,capsaspora_owczarzaki,creolimax_fragrantissima,monosiga_brevicolis,saccharomyces_cerevisiae,salpingoeca_rosetta,sphaeroforma_arctica
and in at least one of: danio_rerio,xenopus_tropicalis,gallus_gallus,homo_sapiens,ciona_intestinalis,branchiostoma_floridae,saccoglossus_kowalevskii,acanthaster_planci,pristionchus_pacificus,caenorhabditis_elegans,drosophila_melanogaster,tribolium_castaneum,daphnia_pulex,ixodes_scapularis,lottia_gigantea,octopus_bimaculoides,phoronis_australis,notospermus_geniculatus,schistosoma_mansoni,schmidtea_mediterranea,capitella_teleta,helobdella_robusta,exaiptasia_pallida,nematostella_vectensis,orbicella_faveolata,acropora_digitifera,pocillopora_damicornis,renilla_muelleri,dendronephthya_gigantea,aurelia_aurita_atlantic,aurelia_aurita_pacific,nemopilema_nomurai,hydractinia_echinata,hydractinia_symbiolongicarpus,hydra_magnipapillata,clytia_hemisphaerica,morbakka_virulenta,hofstenia_miamia,kudoa_iwatai
and lost in: nematostella_vectensis: 2781
```


---

##### Orthogroups present in at least one early-branching taxon and one bilaterian


```
one_X_one_Y_none_Z(bilateria, ancestor)
```


```
Present in at least one of: danio_rerio,xenopus_tropicalis,gallus_gallus,homo_sapiens,ciona_intestinalis,branchiostoma_floridae,saccoglossus_kowalevskii,acanthaster_planci,pristionchus_pacificus,caenorhabditis_elegans,drosophila_melanogaster,tribolium_castaneum,daphnia_pulex,ixodes_scapularis,lottia_gigantea,octopus_bimaculoides,phoronis_australis,notospermus_geniculatus,schistosoma_mansoni,schmidtea_mediterranea,capitella_teleta,helobdella_robusta,exaiptasia_pallida,nematostella_vectensis,orbicella_faveolata,acropora_digitifera,pocillopora_damicornis,renilla_muelleri,dendronephthya_gigantea,aurelia_aurita_atlantic,aurelia_aurita_pacific,nemopilema_nomurai,hydractinia_echinata,hydractinia_symbiolongicarpus,hydra_magnipapillata,clytia_hemisphaerica,morbakka_virulenta,hofstenia_miamia,kudoa_iwatai
and in at least one of: hoilungia_hongkongensis,trichoplax_adhaerens,amphimedon_queenslandica,mnemiopsis_leidyi,capsaspora_owczarzaki,creolimax_fragrantissima,monosiga_brevicolis,saccharomyces_cerevisiae,salpingoeca_rosetta,sphaeroforma_arctica: 9459
```


---

#### Gene counts in cnidarian-specific orthogroups


```
count_orthogroups(cnidaria)
```


```
[1] "Number of species:  1  ; Number of orthogroups:  654  ; Number of genes:  2698"
[1] "Number of species:  2  ; Number of orthogroups:  4255  ; Number of genes:  12756"
[1] "Number of species:  3  ; Number of orthogroups:  1848  ; Number of genes:  9355"
[1] "Number of species:  4  ; Number of orthogroups:  864  ; Number of genes:  5981"
[1] "Number of species:  5  ; Number of orthogroups:  391  ; Number of genes:  3558"
[1] "Number of species:  6  ; Number of orthogroups:  202  ; Number of genes:  2470"
[1] "Number of species:  7  ; Number of orthogroups:  161  ; Number of genes:  2119"
[1] "Number of species:  8  ; Number of orthogroups:  123  ; Number of genes:  1694"
[1] "Number of species:  9  ; Number of orthogroups:  52  ; Number of genes:  894"
[1] "Number of species:  10  ; Number of orthogroups:  35  ; Number of genes:  606"
[1] "Number of species:  11  ; Number of orthogroups:  35  ; Number of genes:  728"
[1] "Number of species:  12  ; Number of orthogroups:  24  ; Number of genes:  648"
[1] "Number of species:  13  ; Number of orthogroups:  31  ; Number of genes:  830"
[1] "Number of species:  14  ; Number of orthogroups:  35  ; Number of genes:  1051"
[1] "Number of species:  15  ; Number of orthogroups:  34  ; Number of genes:  1097"
[1] "Number of species:  16  ; Number of orthogroups:  2  ; Number of genes:  137"
[1] "Present in at least  16  species:  2  orthogroups;  137  genes"
[1] "Present in at least  15  species:  36  orthogroups;  1234  genes"
[1] "Present in at least  14  species:  71  orthogroups;  2285  genes"
[1] "Present in at least  13  species:  102  orthogroups;  3115  genes"
[1] "Present in at least  12  species:  126  orthogroups;  3763  genes"
[1] "Present in at least  11  species:  161  orthogroups;  4491  genes"
[1] "Present in at least  10  species:  196  orthogroups;  5097  genes"
[1] "Present in at least  9  species:  248  orthogroups;  5991  genes"
[1] "Present in at least  8  species:  371  orthogroups;  7685  genes"
[1] "Present in at least  7  species:  532  orthogroups;  9804  genes"
[1] "Present in at least  6  species:  734  orthogroups;  12274  genes"
[1] "Present in at least  5  species:  1125  orthogroups;  15832  genes"
[1] "Present in at least  4  species:  1989  orthogroups;  21813  genes"
[1] "Present in at least  3  species:  3837  orthogroups;  31168  genes"
[1] "Present in at least  2  species:  8092  orthogroups;  43924  genes"
[1] "Present in at least  1  species:  8746  orthogroups;  46622  genes"
```

LS0tCnRpdGxlOiAiT3J0aG9ncm91cCBhbmFseXNpcyIKb3V0cHV0OiBodG1sX25vdGVib29rCi0tLQoKCmBgYHtyfQpsaWJyYXJ5KHRpZHl2ZXJzZSkKbGlicmFyeSh2aXJpZGlzKQpgYGAKCioqKgoKIyMjIFJlYWQgb3J0aG9ncm91cCB0YWJsZQoKYGBge3J9Cm9ydGhvX2NvdW50cyA8LSByZWFkLnRhYmxlKCJPcnRob2dyb3Vwcy5HZW5lQ291bnRfZGVjXzIwLmNzdiIpCm9ydGhvX2NvdW50cwpvcnRob19iaW5hcnkgPC0gb3J0aG9fY291bnRzWywxOjQ5XSAjUmVtb3ZlIHRoZSBUb3RhbHMgY29sdW1uCm9ydGhvX2JpbmFyeVtvcnRob19iaW5hcnkgPj0gMV0gPC0gMSAjQ29udmVydCB0byBwcmVzZW5jZS9hYnNlbmNlCm9ydGhvX2JpbmFyeV9uYW1lcyA8LSBvcnRob19iaW5hcnkgJT4lIG11dGF0ZShvcnRob19pZCA9IHJvdy5uYW1lcyhvcnRob19jb3VudHMpLCBnZW5lX2NvdW50cyA9IHJvd1N1bXMob3J0aG9fY291bnRzKSkKYGBgCgoqKioKCiMjIyBSZWFkIHRheG9ub215IHRhYmxlCgpgYGB7cn0KdGF4b25vbXkgPC0gcmVhZF9jc3YoInRheG9ub215X3J1bjMuY3N2IikKdGF4b25vbXkKYGBgCgoqKioKCiMjIyBGdW5jdGlvbnMKCioqKgoKIyMjIyBQcmVzZW50IGluIGF0IGxlYXN0IG9uZSBvZiBYLCBhYnNlbnQgaW4gYWxsIG9mIFkKIyMjIyMgZGVmYXVsdCBzcGVjaWVzIGZvciBZOiBhbGwgc3BlY2llcyBub3QgaW4gWAojIyMjIyBzcGVjaWVzX2NvbXAgb3V0cHV0cyB0ZXh0OiBudW1iZXIgb2Ygb3J0aG9ncm91cHMgYW5kIHRvdGFsIG51bWJlciBvZiBnZW5lcyBpbiBvcnRob2dyb3VwcwojIyMjIyBzcGVjaWVzX2NvbXBfbnVtIG91dHB1dHMgaW50ZWdlcnMKCmBgYHtyfQojIHNwZWNpZXNfbmFtZXMgaXMgYSB2ZWN0b3Igb2Ygc3BlY2llcyBuYW1lcwpzcGVjaWVzX2NvbXAgPC0gZnVuY3Rpb24oc3BlY2llc19pbiwgc3BlY2llc19vdXQsIG51bWJlcl9zZWxlY3Rfc3BlY2llcyA9IGxlbmd0aChzcGVjaWVzX2luKSl7CiAgaWYgKG1pc3Npbmcoc3BlY2llc19vdXQpKXsKICAgIHNlbGVjdF9zcGVjaWVzIDwtIG9ydGhvX2JpbmFyeSAlPiUgc2VsZWN0KHNwZWNpZXNfaW4pCiAgICBvdGhlcl9zcGVjaWVzIDwtIG9ydGhvX2JpbmFyeSAlPiUgc2VsZWN0KC1zcGVjaWVzX2luKQogICAgc3BlY2lmaWNfb3J0aG9ncm91cHMgPC0gb3J0aG9fYmluYXJ5X25hbWVzW3Jvd1N1bXMoc2VsZWN0X3NwZWNpZXMpID09IG51bWJlcl9zZWxlY3Rfc3BlY2llcyAmIHJvd1N1bXMob3RoZXJfc3BlY2llcykgPT0gMCxdICU+JSBzZWxlY3Qob3J0aG9faWQpCiAgICBnZW5lX251bWJlciA8LSBzdW0ob3J0aG9fY291bnRzW3NwZWNpZmljX29ydGhvZ3JvdXBzJG9ydGhvX2lkLHNwZWNpZXNfaW5dKQogICAgb3V0X3NwZWNpZXNfbmFtZXMgPC0gImFsbCBvdGhlciBzcGVjaWVzIgogIH0KICBlbHNlewogICAgc2VsZWN0X3NwZWNpZXMgPC0gb3J0aG9fYmluYXJ5ICU+JSBzZWxlY3Qoc3BlY2llc19pbikKICAgIG90aGVyX3NwZWNpZXMgPC0gb3J0aG9fYmluYXJ5ICU+JSBzZWxlY3Qoc3BlY2llc19vdXQpCiAgICBzcGVjaWZpY19vcnRob2dyb3VwcyA8LSBvcnRob19iaW5hcnlfbmFtZXNbcm93U3VtcyhzZWxlY3Rfc3BlY2llcykgPT0gbnVtYmVyX3NlbGVjdF9zcGVjaWVzICYgcm93U3VtcyhvdGhlcl9zcGVjaWVzKSA9PSAwLF0gJT4lIHNlbGVjdChvcnRob19pZCkKICAgIG91dF9zcGVjaWVzX25hbWVzIDwtIHBhc3RlKGNvbG5hbWVzKG90aGVyX3NwZWNpZXMpLCBjb2xsYXBzZSA9ICIsIikKICAgIGdlbmVfbnVtYmVyIDwtIHN1bShyb3dTdW1zKG9ydGhvX2NvdW50c1tzcGVjaWZpY19vcnRob2dyb3VwcyRvcnRob19pZCxzcGVjaWVzX2luXSkpCgogIH0KICBvcnRob19udW1iZXIgPC0gbnJvdyhzcGVjaWZpY19vcnRob2dyb3VwcykKICBpbl9zcGVjaWVzX25hbWVzIDwtIHBhc3RlKGNvbG5hbWVzKHNlbGVjdF9zcGVjaWVzKSwgY29sbGFwc2UgPSAiLCIpCiAgb3V0cHV0IDwtIHBhc3RlKCJwcmVzZW50IGluOiAiLCBpbl9zcGVjaWVzX25hbWVzLCAiXG4iLCAiYWJzZW50IGluOiAiLCBvdXRfc3BlY2llc19uYW1lcywgIlxuIiwgIm51bWJlciBvZiBvcnRob2dyb3VwczogIiwgb3J0aG9fbnVtYmVyLCAiXG4iLCAibnVtYmVyIG9mIGdlbmVzOiAiLGdlbmVfbnVtYmVyLCAgc2VwID0gIiIpCiAgY2F0KG91dHB1dCkKICB9CmBgYAoKKioqCgpgYGB7cn0Kc3BlY2llc19jb21wX251bSA8LSBmdW5jdGlvbihzcGVjaWVzX2luLCBzcGVjaWVzX291dCwgbnVtYmVyX3NlbGVjdF9zcGVjaWVzID0gbGVuZ3RoKHNwZWNpZXNfaW4pKXsKICBpZiAobWlzc2luZyhzcGVjaWVzX291dCkpewogICAgc2VsZWN0X3NwZWNpZXMgPC0gb3J0aG9fYmluYXJ5ICU+JSBzZWxlY3Qoc3BlY2llc19pbikKICAgIG90aGVyX3NwZWNpZXMgPC0gb3J0aG9fYmluYXJ5ICU+JSBzZWxlY3QoLXNwZWNpZXNfaW4pCiAgICBzcGVjaWZpY19vcnRob2dyb3VwcyA8LSBvcnRob19iaW5hcnlfbmFtZXNbcm93U3VtcyhzZWxlY3Rfc3BlY2llcykgPT0gbnVtYmVyX3NlbGVjdF9zcGVjaWVzICYgcm93U3VtcyhvdGhlcl9zcGVjaWVzKSA9PSAwLF0gJT4lIHNlbGVjdChvcnRob19pZCkKICAgIGdlbmVfbnVtYmVyIDwtIHN1bShvcnRob19jb3VudHNbc3BlY2lmaWNfb3J0aG9ncm91cHMkb3J0aG9faWQsc3BlY2llc19pbl0pCiAgfQogIGVsc2V7CiAgICBzZWxlY3Rfc3BlY2llcyA8LSBvcnRob19iaW5hcnkgJT4lIHNlbGVjdChzcGVjaWVzX2luKQogICAgb3RoZXJfc3BlY2llcyA8LSBvcnRob19iaW5hcnkgJT4lIHNlbGVjdChzcGVjaWVzX291dCkKICAgIHNwZWNpZmljX29ydGhvZ3JvdXBzIDwtIG9ydGhvX2JpbmFyeV9uYW1lc1tyb3dTdW1zKHNlbGVjdF9zcGVjaWVzKSA9PSBudW1iZXJfc2VsZWN0X3NwZWNpZXMgJiByb3dTdW1zKG90aGVyX3NwZWNpZXMpID09IDAsXSAlPiUgc2VsZWN0KG9ydGhvX2lkKQogICAgZ2VuZV9udW1iZXIgPC0gc3VtKHJvd1N1bXMob3J0aG9fY291bnRzW3NwZWNpZmljX29ydGhvZ3JvdXBzJG9ydGhvX2lkLHNwZWNpZXNfaW5dKSkKCiAgfQogIG9ydGhvX251bWJlciA8LSBucm93KHNwZWNpZmljX29ydGhvZ3JvdXBzKQogIG91dHB1dCA8LSBjKG9ydGhvX251bWJlciwgZ2VuZV9udW1iZXIpCiAgb3V0cHV0CiAgfQpgYGAKKioqCiMjIyMgUHJlc2VudCBpbiBhdCBsZWFzdCBvbmUgb2YgWCBhbmQgaW4gYXQgbGVhc3Qgb25lIG9mIHksIGxvc3QgaW4gYWxsIG9mIFogCiMjIyMjIGRlZmF1bHQgc3BlY2llcyBmb3IgWjogYWxsIHNwZWNpZXMgbm90IGluIFggb3IgWQoKYGBge3J9Cm9uZV9YX29uZV9ZX25vbmVfWiA8LSBmdW5jdGlvbih4LCB5LCB6KXsKICBhbmNlc3RyYWxfc3BlY2llcyA8LSBvcnRob19iaW5hcnkgJT4lIHNlbGVjdCh4KQogIHNoYXJlZF9zcGVjaWVzIDwtIG9ydGhvX2JpbmFyeSAlPiUgc2VsZWN0KHkpCiAgaWYgKG1pc3NpbmcoeikpewogICAgc2hhcmVkX29ydGhvZ3JvdXBzIDwtIG9ydGhvX2JpbmFyeVtyb3dTdW1zKGFuY2VzdHJhbF9zcGVjaWVzKSA+PSAxICYgcm93U3VtcyhzaGFyZWRfc3BlY2llcykgPj0xLF0KICAgIHNoYXJlZF9udW0gPC0gbnJvdyhzaGFyZWRfb3J0aG9ncm91cHMpCiAgICBhbmNfbmFtZXMgPC0gcGFzdGUoeCwgY29sbGFwc2UgPSAiLCIpCiAgICBzaGFyZWRfbmFtZXMgPC0gcGFzdGUoY29sbmFtZXMoc2hhcmVkX3NwZWNpZXMpLCBjb2xsYXBzZSA9ICIsIikKICAgIG91dHB1dCA8LSBwYXN0ZShjKCJQcmVzZW50IGluIGF0IGxlYXN0IG9uZSBvZjogIiwgYW5jX25hbWVzLCAiXG5hbmQgaW4gYXQgbGVhc3Qgb25lIG9mOiAiLCBzaGFyZWRfbmFtZXMsICI6ICIsIHNoYXJlZF9udW0pLCBjb2xsYXBzZSA9ICIiKQogICAgY2F0KG91dHB1dCkKICB9CiAgZWxzZXsKICAgIGZvciAoc3BlY2llcyBpbiB6KXsKICAgICAgaWYgKHNwZWNpZXMgJWluJSBjb2xuYW1lcyh5KSl7CiAgICAgICAgc2hhcmVkX3NwZWNpZXMgPC0gc2hhcmVkX3NwZWNpZXMgJT4lICgtc3BlY2llcykKICAgICAgfQogICAgfSAKICBsb3N0X3NwZWNpZXMgPC0gb3J0aG9fYmluYXJ5ICU+JSBzZWxlY3QoeikKICBsb3N0X29ydGhvZ3JvdXBzIDwtIG9ydGhvX2JpbmFyeVtyb3dTdW1zKGFuY2VzdHJhbF9zcGVjaWVzKSA+PSAxICYgcm93U3VtcyhzaGFyZWRfc3BlY2llcykgPj0xICYgcm93U3Vtcyhsb3N0X3NwZWNpZXMpID09IDAsXQogIGxvc3RfbnVtIDwtIG5yb3cobG9zdF9vcnRob2dyb3VwcykKICBhbmNfbmFtZXMgPC0gcGFzdGUoeCwgY29sbGFwc2UgPSAiLCIpCiAgc2hhcmVkX25hbWVzIDwtIHBhc3RlKGNvbG5hbWVzKHNoYXJlZF9zcGVjaWVzKSwgY29sbGFwc2UgPSAiLCIpCiAgbG9zdF9uYW1lcyA8LSBwYXN0ZSh6LCBjb2xsYXBzZSA9ICIsIikKICBvdXRwdXQgPC0gcGFzdGUoYygiUHJlc2VudCBpbiBhdCBsZWFzdCBvbmUgb2Y6ICIsIGFuY19uYW1lcywgIlxuYW5kIGluIGF0IGxlYXN0IG9uZSBvZjogIiwgc2hhcmVkX25hbWVzLCAiXG5hbmQgbG9zdCBpbjogIiwgbG9zdF9uYW1lcywgIjogIiwgbG9zdF9udW0pLCBjb2xsYXBzZSA9ICIiKQogIGNhdChvdXRwdXQpCiAgfQp9CmBgYAoKKioqCiMjIyMgUHJlc2VudCBpbiBhdCBsZWFzdCBvbmUgb2YgWCwgbm9uZSBvZiBZCgpgYGB7cn0Kb25lX1hfbm9uZV9ZIDwtIGZ1bmN0aW9uKHgsIHkpewogIHNlbGVjdF9zcGVjaWVzIDwtIG9ydGhvX2JpbmFyeSAlPiUgc2VsZWN0KHgpCiAgb3RoZXJfc3BlY2llcyA8LSBvcnRob19iaW5hcnkgJT4lIHNlbGVjdCh5KQogIHNwZWNpZmljX29ydGhvZ3JvdXBzIDwtIG9ydGhvX2JpbmFyeVtyb3dTdW1zKHNlbGVjdF9zcGVjaWVzKSAgPj0xICYgcm93U3VtcyhvdGhlcl9zcGVjaWVzKSA9PSAwLF0KICBudW0gPC0gbnJvdyhzcGVjaWZpY19vcnRob2dyb3VwcykKICBhdF9sZWFzdF9vbmVfbmFtZXMgPC0gcGFzdGUoeCwgY29sbGFwc2UgPSAiLCIpCiAgbm9uZV9uYW1lcyA8LSBwYXN0ZSh5LCBjb2xsYXBzZSA9ICIsIikKICBvdXRwdXQgPC0gcGFzdGUoYygiUHJlc2VudCBpbiBhdCBsZWFzdCBvbmUgb2Y6ICIsIGF0X2xlYXN0X29uZV9uYW1lcywgIlxuYW5kIGluIG5vbmUgb2Y6ICIsIG5vbmVfbmFtZXMsICI6ICIsIG51bSksIGNvbGxhcHNlID0gIiIpCiAgY2F0KG91dHB1dCkKfQpgYGAKCioqKgoKIyMjIyBQcmVzZW50IGluIGF0IGxlYXN0IG9uZSBvZiBYLCBhbmQgYXQgbGVhc3Qgb25lIG9mIFksIGFuZCBsb3N0IGluIGF0IGxlYXN0IG9uZSBvZiBaCiMjIyMjIFVzZWQgdG8gY2FsY3VsYXRlIGxvc3NlcyBzaW5jZSBhbmNlc3RvcgoKYGBge3J9Cm9uZV9YX29uZV9ZX2xvc3Rfb25lX1ogPC0gZnVuY3Rpb24oYW5jZXN0b3JzLCBzaGFyZWQsIGxvc3QpewogIGFuY2VzdHJhbF9zcGVjaWVzIDwtIG9ydGhvX2JpbmFyeSAlPiUgc2VsZWN0KGFuY2VzdG9ycykKICBzaGFyZWRfc3BlY2llcyA8LSBvcnRob19iaW5hcnkgJT4lIHNlbGVjdChzaGFyZWQpCiAgbG9zdF9udW1iZXIgPC0gbGVuZ3RoKGxvc3QpCiAgZm9yIChzcGVjaWVzIGluIGxvc3QpewogICAgaWYgKHNwZWNpZXMgJWluJSBjb2xuYW1lcyhzaGFyZWQpKXsKICAgICAgc2hhcmVkX3NwZWNpZXMgPC0gc2hhcmVkX3NwZWNpZXMgJT4lICgtc3BlY2llcykKICAgIH0KICB9CiAgbG9zdF9zcGVjaWVzIDwtIG9ydGhvX2JpbmFyeSAlPiUgc2VsZWN0KGxvc3QpCiAgbG9zdF9vcnRob2dyb3VwcyA8LSBvcnRob19iaW5hcnlbcm93U3VtcyhhbmNlc3RyYWxfc3BlY2llcykgPj0gMSAmIHJvd1N1bXMoc2hhcmVkX3NwZWNpZXMpID49MSAmIHJvd1N1bXMobG9zdF9zcGVjaWVzKSA8IGxvc3RfbnVtYmVyLF0KICBsb3N0X251bSA8LSBucm93KGxvc3Rfb3J0aG9ncm91cHMpCiAgYW5jX25hbWVzIDwtIHBhc3RlKGFuY2VzdG9ycywgY29sbGFwc2UgPSAiLCIpCiAgc2hhcmVkX25hbWVzIDwtIHBhc3RlKGNvbG5hbWVzKHNoYXJlZF9zcGVjaWVzKSwgY29sbGFwc2UgPSAiLCIpCiAgbG9zdF9uYW1lcyA8LSBwYXN0ZShsb3N0LCBjb2xsYXBzZSA9ICIsIikKICBvdXRwdXQgPC0gcGFzdGUoYygiUHJlc2VudCBpbiBhdCBsZWFzdCBvbmUgb2Y6ICIsIGFuY19uYW1lcywgIlxuYW5kIGluIGF0IGxlYXN0IG9uZSBvZjogIiwgc2hhcmVkX25hbWVzLCAiXG5hbmQgbG9zdCBpbiBhdCBsZWFzdCBvbmUgb2Y6ICIsIGxvc3RfbmFtZXMsICI6ICIsIGxvc3RfbnVtKSwgY29sbGFwc2UgPSAiIikKICBjYXQob3V0cHV0KQp9CmBgYAoKKioqCgojIyMjIENvdW50IG9ydGhvZ3JvdXBzIGluIGF0IGxlYXN0IHggc3BlY2llcyBpbiB0YXhvbgoKYGBge3J9CmNvdW50X29ydGhvZ3JvdXBzIDwtIGZ1bmN0aW9uKHNwZWNpZXMpewogIG51bV9zcGVjaWVzIDwtIGxlbmd0aChzcGVjaWVzKQogIG9nX2NvdW50cyA8LSBpbnRlZ2VyKDApCiAgZ2VuZV9jb3VudHMgPC0gaW50ZWdlcigwKQogIGZvciAoaSBpbiAxOm51bV9zcGVjaWVzKXsKICAgIGNvbXAgPC0gc3BlY2llc19jb21wX251bShzcGVjaWVzLCBudW1iZXJfc2VsZWN0X3NwZWNpZXMgPSBpKQogICAgbnVtIDwtIGNvbXBbMV0KICAgIGdlbmVzIDwtIGNvbXBbMl0KICAgIG9nX2NvdW50c1tpXSA8LSBudW0KICAgIGdlbmVfY291bnRzW2ldIDwtIGdlbmVzCiAgICBwcmludChwYXN0ZSgiTnVtYmVyIG9mIHNwZWNpZXM6ICIsIGksICIgOyBOdW1iZXIgb2Ygb3J0aG9ncm91cHM6ICIsIG51bSwgIiA7IE51bWJlciBvZiBnZW5lczogIiwgZ2VuZXMpKQogIH0KICByZXZfb2dfY291bnQgPC0gcmV2KG9nX2NvdW50cykKICByZXZfZ2VuZV9jb3VudHMgPC0gcmV2KGdlbmVfY291bnRzKQogIGZvciAoaSBpbiAxOm51bV9zcGVjaWVzKXsKICAgIGogPC0gIG51bV9zcGVjaWVzIC0gKGkgLSAxKQogICAgb2dfY3VtdWxhdGl2ZV9zdW0gPC0gc3VtKHJldl9vZ19jb3VudFsxOmldKQogICAgZ2VuZV9jdW11bGF0aXZlX3N1bSA8LSBzdW0ocmV2X2dlbmVfY291bnRzWzE6aV0pCiAgICBwcmludChwYXN0ZSgiUHJlc2VudCBpbiBhdCBsZWFzdCAiLCBqLCAiIHNwZWNpZXM6ICIsIG9nX2N1bXVsYXRpdmVfc3VtLCAiIG9ydGhvZ3JvdXBzOyAiLCAgZ2VuZV9jdW11bGF0aXZlX3N1bSwgIiBnZW5lcyIgKSkKICB9Cn0KYGBgCgoqKioKCiMjIyMgQ291bnRzIG9ydGhvZ3JvdXBzIHByZXNlbnQgaW4gYSB0YXhvbiwgcmVnYXJkbGVzcyBvZiBvdGhlciB0YXhhCgpgYGB7cn0Kc2hhcmVkX29ydGhvZ3JvdXBzIDwtIGZ1bmN0aW9uKHNwZWNpZXNfaW4pewogIHNlbGVjdF9zcGVjaWVzIDwtIG9ydGhvX2JpbmFyeSAlPiUgc2VsZWN0KHNwZWNpZXNfaW4pCiAgb3RoZXJfc3BlY2llcyA8LSBvcnRob19iaW5hcnkgJT4lIHNlbGVjdCgtc3BlY2llc19pbikKICAjbnVtYmVyX3NlbGVjdF9zcGVjaWVzIDwtIGxlbmd0aChzcGVjaWVzX2luKQogIHNwZWNpZmljX29ydGhvZ3JvdXBzIDwtIG9ydGhvX2JpbmFyeVtyb3dTdW1zKHNlbGVjdF9zcGVjaWVzKSA9PSBsZW5ndGgoc3BlY2llc19pbiksXQogIG9ydGhvX251bWJlciA8LSBucm93KHNwZWNpZmljX29ydGhvZ3JvdXBzKQogIGluX3NwZWNpZXNfbmFtZXMgPC0gcGFzdGUoY29sbmFtZXMoc2VsZWN0X3NwZWNpZXMpLCBjb2xsYXBzZSA9ICIsIikKICBvdXRwdXQgPC0gcGFzdGUoInByZXNlbnQgaW46ICIsIGluX3NwZWNpZXNfbmFtZXMsICJcbiIsICJudW1iZXIgb2Ygb3J0aG9ncm91cHM6ICIsIG9ydGhvX251bWJlciwgIlxuIiwgc2VwID0gIiIpCiAgY2F0KG91dHB1dCkKfQpgYGAKCioqKgoKIyMjIyBTdWJzZXQgYW5kIGdyb3VwIHNwZWNpZXMgZnJvbSB0YXhvbgoKYGBge3J9CnNlbGVjdF9jbGFkZSA8LSBmdW5jdGlvbihjbGFkZV9uYW1lKXsKICBjbGFkZSA8LSB0YXhvbm9teSAlPiUgZmlsdGVyKGNsYWRlID09IGNsYWRlX25hbWUpCiAgc2VsZWN0X3NwZWNpZXMgPC0gY2xhZGUkc3BlY2llcwogIHNlbGVjdF9zcGVjaWVzCn0KYGBgCgpgYGB7cn0KZnVzZV90YXhhIDwtIGZ1bmN0aW9uKGNsYWRlX25hbWVzKXsKICBzZWxlY3Rfc3BlY2llcyA8LSB0YXhvbm9teSAlPiUgZmlsdGVyKGNsYWRlICVpbiUgY2xhZGVfbmFtZXMpCiAgc3BlY2llc19uYW1lcyA8LSBzZWxlY3Rfc3BlY2llcyRzcGVjaWVzCiAgc3BlY2llc19uYW1lcwp9CmBgYAoqKioKKioqCgojIyMgUXVlcnkgb3J0aG9maW5kZXIgb3V0cHV0CgoqKioKIyMjIyBPcnRob2dyb3VwcyBzcGVjaWZpYyB0byBIeWRyYWN0aW5pYQoKIyMjIyMgZ2VudXMgSHlkcmFjdGluaWEgb25seQoKYGBge3J9Cmh5ZHJhY3RpbmlhIDwtIGMoImh5ZHJhY3RpbmlhX2VjaGluYXRhIiwgImh5ZHJhY3RpbmlhX3N5bWJpb2xvbmdpY2FycHVzIikKc3BlY2llc19jb21wKGh5ZHJhY3RpbmlhKQpgYGAKCioqKgoKIyMjIyMgSHlkcmFjdGluaWEgZWNoaW5hdGEgb25seQoKYGBge3J9CnNwZWNpZXNfY29tcCgiaHlkcmFjdGluaWFfZWNoaW5hdGEiKQpgYGAKCioqKgoKIyMjIyMgSHlkcmFjdGluaWEgc3ltYmlvbG9uZ2ljYXJwdXMgb25seQoKYGBge3J9CnNwZWNpZXNfY29tcCgiaHlkcmFjdGluaWFfc3ltYmlvbG9uZ2ljYXJwdXMiKQpgYGAKKioqCgojIyMjIyBPcnRob2dyb3VwcyBzcGVjaWZpYyB0byBvbmUgY25pZGFyaWFuCgpgYGB7cn0KY25pZGFyaWEgPC0gZnVzZV90YXhhKGMoIkFjdGluYXJpYSIsICJIZXhhY29yYWxsaWEiLCAiT2N0b2NvcmFsbGlhIiwgIlNjeXBob3pvYSIsICJDdWJvem9hIiwgIkh5ZHJvem9hIiwgICJNeXhvem9hIikpCmNuaWRhcmlhCmBgYAoKCmBgYHtyfQpmb3IgKHNwZWNpZXMgaW4gY25pZGFyaWEpewogIHNwZWNpZXNfY29tcChzcGVjaWVzKQogIGNhdCgiXG5cbiIpCn0KYGBgCioqKgojIyMjIEh5ZHJvem9hIHF1ZXJpZXMKKioqCiMjIyMjIE9ydGhvZ3JvdXBzIHByZXNlbnQgaW4gYWxsIGh5ZHJvem9hbnMgYnV0IG5vIG90aGVyIHRheGEgCgpgYGB7cn0KaHlkcm96b2EgPC0gc2VsZWN0X2NsYWRlKCJIeWRyb3pvYSIpCnNwZWNpZXNfY29tcChoeWRyb3pvYSkKYGBgCioqKgoKIyMjIyMgT3J0aG9ncm91cHMgcHJlc2VudCBpbiAzIGh5ZHJvem9hbnMgYnV0IG5vIG90aGVyIHRheGEgCgoKYGBge3J9CmZvciAoY29tYiBpbiBjb21ibihoeWRyb3pvYSwgMywgc2ltcGxpZnkgPSBGKSl7CiAgc3BlY2llc19jb21wKGNvbWIpCiAgY2F0KCJcblxuIikKfQpgYGAKCioqKgoKIyMjIyMgT3J0aG9ncm91cHMgcHJlc2VudCBpbiAyIGh5ZHJvem9hbnMgYnV0IG5vIG90aGVyIHRheGEgCgpgYGB7cn0KZm9yIChjb21iIGluIGNvbWJuKGh5ZHJvem9hLCAyLCBzaW1wbGlmeSA9IEYpKXsKICBzcGVjaWVzX2NvbXAoY29tYikKICBjYXQoIlxuXG4iKQp9CmBgYAoqKioKCiMjIyMjIE9ydGhvZ3JvdXBzIHByZXNlbnQgb25seSBpbiBOZW1hdG9zdGVsbGEgYW5kIEh5ZHJhCgpgYGB7cn0Kc3BlY2llc19jb21wKGMoIm5lbWF0b3N0ZWxsYV92ZWN0ZW5zaXMiLCAiaHlkcmFfbWFnbmlwYXBpbGxhdGEiKSkKYGBgCgoqKioKKioqCgojIyMjIEFudGhvem9hIHF1ZXJpZXMKCmBgYHtyfQphbnRob3pvYSA8LSBmdXNlX3RheGEoYygiT2N0b2NvcmFsbGlhIiwgIkhleGFjb3JhbGxpYSIsICJBY3RpbmFyaWEiKSkKYW50aG96b2EKYGBgCioqKgojIyMjIyBPcnRob2dyb3VwcyBzaGFyZWQgYnkgYWxsIGFudGhvem9hbnMKCmBgYHtyfQpzaGFyZWRfb3J0aG9ncm91cHMoYW50aG96b2EpCmBgYAoqKioKCiMjIyMjIE9ydGhvZ3JvdXBzIHNoYXJlZCBieSBhbGwgYW50aG96b2FucyBhbmQgYWJzZW50IGluIGFsbCBvdGhlciB0YXhhCgpgYGB7cn0Kc3BlY2llc19jb21wKGFudGhvem9hKQpgYGAKKioqCiMjIyMjIE9ydGhvZ3JvdXBzIHByZXNlbnQgaW4gYXQgbGVhc3Qgb25lIGFudGhvem9hbiBhbmQgYWJzZW50IGluIGFsbCBvdGhlciB0YXhhCgpgYGB7cn0KY291bnRfb3J0aG9ncm91cHMoYW50aG96b2EpCmBgYAoqKioKKioqCiMjIyMgTWVkdXNvem9hIHF1ZXJpZXMKKioqCmBgYHtyfQptZWR1c296b2EgPC0gZnVzZV90YXhhKGMoIkh5ZHJvem9hIiwgIlNjeXBob3pvYSIsICJDdWJvem9hIikpCm1lZHVzb3pvYQpgYGAKKioqCiMjIyMjIE9ydGhvZ3JvdXBzIHNoYXJlZCBieSBhbGwgbWVkdXNvem9hbnMKCmBgYHtyfQpzaGFyZWRfb3J0aG9ncm91cHMobWVkdXNvem9hKQpgYGAKKioqCiMjIyMjIE9ydGhvZ3JvdXBzIHNoYXJlZCBieSBhbGwgbWVkdXNvem9hbnMgYW5kIGFic2VudCBpbiBhbGwgb3RoZXIgdGF4YQoKYGBge3J9CnNwZWNpZXNfY29tcChtZWR1c296b2EpCmBgYAoqKioKIyMjIyMgT3J0aG9ncm91cHMgcHJlc2VudCBpbiBhdCBsZWFzdCBvbmUgbWVkdXNvem9hbiBhbmQgYWJzZW50IGluIGFsbCBvdGhlciB0YXhhCgpgYGB7cn0KY291bnRfb3J0aG9ncm91cHMobWVkdXNvem9hKQpgYGAKCioqKgoqKioKIyMjIyBDbmlkYXJpYSBxdWVyaWVzCgpgYGB7cn0KY25pZGFyaWEgPC0gZnVzZV90YXhhKGMoIkFjdGluYXJpYSIsICJIZXhhY29yYWxsaWEiLCAiT2N0b2NvcmFsbGlhIiwgIlNjeXBob3pvYSIsICJDdWJvem9hIiwgIkh5ZHJvem9hIiwgICJNeXhvem9hIikpCmNuaWRhcmlhCmBgYAoKKioqCiMjIyMjIE9ydGhvZ3JvdXBzIHByZXNlbnQgaW4gb25lIG9yIG1vcmUgY25pZGFyaWFuIGFuZCBhYnNlbnQgaW4gYWxsIG90aGVyIHNwZWNpZXMKCgpgYGB7cn0KY291bnRfb3J0aG9ncm91cHMoY25pZGFyaWEpCmBgYAoKKioqCioqKgoKIyMjIyMgT3J0aG9ncm91cHMgcHJlc2VudCBpbiBvbmUgbm9uLW1ldGF6b2FuIGFuZCBhYnNlbnQgaW4gYWxsIGNuaWRhcmlhbnMKCgpgYGB7cn0Kbm9uX21ldGF6b2FucyA8LSBzZWxlY3RfY2xhZGUoIk5vbi1tZXRhem9hbnMiKQpub25fbWV0YXpvYW5zCmBgYAoKCmBgYHtyfQpvbmVfWF9ub25lX1kobm9uX21ldGF6b2FucywgY25pZGFyaWEpCmBgYAoKKioqCgojIyMjIyBPcnRob2dyb3VwcyBwcmVzZW50IGluIGF0IGxlYXN0IG9uZSBub24tbWV0YXpvYW4gYW5kIGF0IGxlYXN0IG9uZSBjbmlkYXJpYW4gYnV0IGFic2VudCBpbiBIeWRyYWN0aW5pYSBlY2hpbmF0YQoKYGBge3J9Cm9uZV9YX29uZV9ZX25vbmVfWihub25fbWV0YXpvYW5zLCBjbmlkYXJpYSwgImh5ZHJhY3RpbmlhX2VjaGluYXRhIikKYGBgCioqKgojIyMjIyBPcnRob2dyb3VwcyBwcmVzZW50IGluIGF0IGxlYXN0IG9uZSBub24tbWV0YXpvYW4gYW5kIGF0IGxlYXN0IG9uZSBjbmlkYXJpYW4gYnV0IGFic2VudCBpbiBIeWRyYWN0aW5pYSBzeW1iaW9sb25naWNhcnB1cwoKYGBge3J9Cm9uZV9YX29uZV9ZX25vbmVfWihub25fbWV0YXpvYW5zLCBjbmlkYXJpYSwgImh5ZHJhY3RpbmlhX3N5bWJpb2xvbmdpY2FycHVzIikKYGBgCioqKgojIyMjIyBPcnRob2dyb3VwcyBwcmVzZW50IGluIGF0IGxlYXN0IG9uZSBub24tbWV0YXpvYW4gYW5kIGF0IGxlYXN0IG9uZSBjbmlkYXJpYW4gYnV0IGFic2VudCBpbiBIeWRyYQoKYGBge3J9Cm9uZV9YX29uZV9ZX25vbmVfWihub25fbWV0YXpvYW5zLCBjbmlkYXJpYSwgImh5ZHJhX21hZ25pcGFwaWxsYXRhIikKYGBgCioqKgojIyMjIyBPcnRob2dyb3VwcyBwcmVzZW50IGluIGF0IGxlYXN0IG9uZSBub24tbWV0YXpvYW4gYW5kIGF0IGxlYXN0IG9uZSBjbmlkYXJpYW4gYnV0IGFic2VudCBpbiBib3RoIEh5ZHJhY3RpbmlhCgoKYGBge3J9Cm9uZV9YX29uZV9ZX25vbmVfWihub25fbWV0YXpvYW5zLCBjbmlkYXJpYSwgaHlkcmFjdGluaWEpCmBgYAoqKioKIyMjIyMgT3J0aG9ncm91cHMgcHJlc2VudCBpbiBhdCBsZWFzdCBvbmUgbm9uLW1ldGF6b2FuIGFuZCBhdCBsZWFzdCBvbmUgY25pZGFyaWFuIGJ1dCBhYnNlbnQgaW4gQ2x5dGlhCgpgYGB7cn0Kb25lX1hfb25lX1lfbm9uZV9aKG5vbl9tZXRhem9hbnMsIGNuaWRhcmlhLCAiY2x5dGlhX2hlbWlzcGhhZXJpY2EiKQpgYGAKKioqCgojIyMjIyBPcnRob2dyb3VwcyBwcmVzZW50IGluIGF0IGxlYXN0IG9uZSBub24tbWV0YXpvYW4gYW5kIEh5ZHJhIGJ1dCBhYnNlbnQgaW4gYm90aCBIeWRyYWN0aW5pYQoKYGBge3J9Cm9uZV9YX29uZV9ZX25vbmVfWihub25fbWV0YXpvYW5zLCAiaHlkcmFfbWFnbmlwYXBpbGxhdGEiLCBoeWRyYWN0aW5pYSkKYGBgCioqKgojIyMjIyBPcnRob2dyb3VwcyBwcmVzZW50IGluIGF0IGxlYXN0IG9uZSBub24tbWV0YXpvYW4gYW5kIGF0IGxlYXN0IG9uZSBIeWRyYWN0aW5pYSBidXQgYWJzZW50IGluIEh5ZHJhCgpgYGB7cn0Kb25lX1hfb25lX1lfbm9uZV9aKG5vbl9tZXRhem9hbnMsIGh5ZHJhY3RpbmlhLCAiaHlkcmFfbWFnbmlwYXBpbGxhdGEiKQpgYGAKKioqCiMjIyMjIE9ydGhvZ3JvdXBzIHByZXNlbnQgaW4gYXQgbGVhc3Qgb25lIG5vbi1tZXRhem9hbiBidXQgYWJzZW50IGluIGFsbCBjbmlkYXJpYW5zCgoKYGBge3J9Cm9uZV9YX25vbmVfWShub25fbWV0YXpvYW5zLCBjbmlkYXJpYSkKYGBgCioqKgojIyMjIyBPcnRob2dyb3VwcyBwcmVzZW50IGluIGF0IGxlYXN0IG9uZSBub24tbWV0YXpvYW4gYW5kIG9uZSBiaWxhdGVyaWFuIGJ1dCBhYnNlbnQgaW4gYWxsIGNuaWRhcmlhbnMJCgpgYGB7cn0Kbm9uX2JpbGF0ZXJpYW5zIDwtIGMoY25pZGFyaWEsICJQb3JpZmVyYSIsICJDdGVub3Bob3JhIiwgIlBsYWNvem9hIiwgIk5vbi1tZXRhem9hbnMiKQpub25fYmlsYXRlcmlhbnMKYGBgCgpgYGB7cn0KYmlsYXRlcmlhIDwtIHRheG9ub215ICU+JSBmaWx0ZXIoIWNsYWRlICVpbiUgbm9uX2JpbGF0ZXJpYW5zKQpiaWxhdGVyaWEgPC0gYmlsYXRlcmlhJHNwZWNpZXMKYmlsYXRlcmlhCmBgYAoKCmBgYHtyfQpvbmVfWF9vbmVfWV9ub25lX1oobm9uX21ldGF6b2FucywgYmlsYXRlcmlhLCBjbmlkYXJpYSkKYGBgCioqKgoKIyMjIyMgT3V0cHV0IHRoZXNlIG9ydGhvZ3JvdXBzCgpgYGB7cn0KeCA8LSBvcnRob19iaW5hcnkgJT4lIHNlbGVjdChub25fbWV0YXpvYW5zKQp5IDwtIG9ydGhvX2JpbmFyeSAlPiUgc2VsZWN0KGJpbGF0ZXJpYSkKeiA8LSBvcnRob19iaW5hcnkgJT4lIHNlbGVjdChjbmlkYXJpYSkKbG9zdF9vcnRob2dyb3VwcyA8LSBvcnRob19iaW5hcnlbcm93U3Vtcyh4KSA+PSAxICYgcm93U3Vtcyh5KSA+PTEgJiByb3dTdW1zKHopID09IDAsXQpsb3N0X29ydGhvZ3JvdXBzCmBgYAoqKioKCiMjIyMjIE9ydGhvZ3JvdXBzIHByZXNlbnQgaW4gbm9uLW1ldGF6b2FucyBhbmQgYXQgbGVhc3Qgb25lIGJpbGF0ZXJpYW4gYnV0IGFic2VudCBpbiBhdCBsZWFzdCBvbmUgSHlkcmFjdGluaWEKCgpgYGB7cn0Kb25lX1hfb25lX1lfbG9zdF9vbmVfWihub25fbWV0YXpvYW5zLCBiaWxhdGVyaWEsIGh5ZHJhY3RpbmlhKQpgYGAKCioqKgojIyMjIyBPcnRob2dyb3VwcyBwcmVzZW50IGluIG5vbi1tZXRhem9hbnMgYW5kIGF0IGxlYXN0IG9uZSBiaWxhdGVyaWFuIGJ1dCBhYnNlbnQgaW4gYm90aCBIeWRyYWN0aW5pYQoKYGBge3J9Cm9uZV9YX29uZV9ZX25vbmVfWihub25fbWV0YXpvYW5zLCBiaWxhdGVyaWEsIGh5ZHJhY3RpbmlhKQpgYGAKKioqCiMjIyMjIE9ydGhvZ3JvdXBzIHByZXNlbnQgaW4gYXQgbGVhc3Qgb25lIG5vbi1tZXRhem9hbiBhbmQgb25lIGJpbGF0ZXJpYW4gYnV0IGFic2VudCBpbiBvbmUgaHlkcm96b2FuCQoKYGBge3J9CmZvciAoc3BlY2llcyBpbiBoeWRyb3pvYSl7CiAgb25lX1hfb25lX1lfbm9uZV9aKG5vbl9tZXRhem9hbnMsIGJpbGF0ZXJpYSwgc3BlY2llcykKICBjYXQoIlxuXG4iKQp9IApgYGAKCioqKgojIyMjIyBPcnRob2dyb3VwcyBwcmVzZW50IGluIGF0IGxlYXN0IG9uZSBub24tbWV0YXpvYW4gYW5kIG9uZSBiaWxhdGVyaWFuIGJ1dCBhYnNlbnQgaW4gYWxsIGh5ZHJvem9hbnMKCmBgYHtyfQpvbmVfWF9vbmVfWV9ub25lX1oobm9uX21ldGF6b2FucywgYmlsYXRlcmlhLCBoeWRyb3pvYSkKYGBgCgoqKioKCiMjIyMjIE9ydGhvZ3JvdXBzIHByZXNlbnQgaW4gYXQgbGVhc3Qgb25lIG5vbi1tZXRhem9hbiBhbmQgb25lIGJpbGF0ZXJpYW4gYnV0IGFic2VudCBpbiBhbGwgbWVkdXNvem9hbnMKCmBgYHtyfQptZWR1c296b2EgPC0gZnVzZV90YXhhKGMoIkh5ZHJvem9hIiwgIlNjeXBob3pvYSIsICJDdWJvem9hIikpCm1lZHVzb3pvYQpgYGAKCmBgYHtyfQpvbmVfWF9vbmVfWV9ub25lX1oobm9uX21ldGF6b2FucywgYmlsYXRlcmlhLCBtZWR1c296b2EpCmBgYAoKKioqCgojIyMjIyBPcnRob2dyb3VwcyBwcmVzZW50IGluIGF0IGxlYXN0IG9uZSBub24tbWV0YXpvYW4gYW5kIG9uZSBiaWxhdGVyaWFuIGJ1dCBhYnNlbnQgaW4gTmVtYXRvc3RlbGxhCgpgYGB7cn0Kb25lX1hfb25lX1lfbm9uZV9aKG5vbl9tZXRhem9hbnMsIGJpbGF0ZXJpYSwgIm5lbWF0b3N0ZWxsYV92ZWN0ZW5zaXMiKQpgYGAKCioqKgojIyMjIyBPcnRob2xvZ3MgcHJlc2VudCBpbiBhdCBsYXN0IG9uZSBiaWxhdGVyaWFuIGFuZCBvbmUgbm9uLW1ldGF6b2FuCgpgYGB7cn0Kb25lX1hfb25lX1lfbm9uZV9aKGJpbGF0ZXJpYSwgbm9uX21ldGF6b2FucykKYGBgCgoqKioKIyMjIyMgT3J0aG9sb2dzIHByZXNlbnQgaW4gYXQgbGFzdCBvbmUgYmlsYXRlcmlhbiBhbmQgb25lIEh5ZHJhY3RpbmlhCgoKYGBge3J9Cm9uZV9YX29uZV9ZX25vbmVfWihiaWxhdGVyaWEsIGh5ZHJhY3RpbmlhKQpgYGAKKioqCiMjIyMjIE9ydGhvbG9ncyBwcmVzZW50IGluIGF0IGxlYXN0IG9uZSBiaWxhdGVyaWFuIGFuZCBNbmVtaW9wc2lzIG9yIEFtcGhpbWVkb24KCmBgYHtyfQpjdGVub19zcG9uZ2UgPC1jKCJtbmVtaW9wc2lzX2xlaWR5aSIsICJhbXBoaW1lZG9uX3F1ZWVuc2xhbmRpY2EiKQpvbmVfWF9vbmVfWV9ub25lX1ooYmlsYXRlcmlhLCBjdGVub19zcG9uZ2UpCmBgYAoqKioKIyMjIyMgT3J0aG9sb2dzIHByZXNlbnQgaW4gSHlkcmFjdGluaWEgYW5kIGh1bWFuCgpgYGB7cn0Kb25lX1hfb25lX1lfbm9uZV9aKGh5ZHJhY3RpbmlhLCAiaG9tb19zYXBpZW5zIikKYGBgCioqKgojIyMjIEN0ZW5vcGhvcmEgcXVlcmllcwoqKioKIyMjIyMgT3J0aG9ncm91cHMgcHJlc2VudCBpbiBjdGVub3Bob3JlcyBhbmQgYmlsYXRlcmlhbnMgYnV0IGFic2VudCBpbiBjbmlkYXJpYW5zCgpgYGB7cn0Kb25lX1hfb25lX1lfbm9uZV9aKCJtbmVtaW9wc2lzX2xlaWR5aSIsIGJpbGF0ZXJpYSwgY25pZGFyaWEpCmBgYAoqKioKIyMjIyMgT3J0aG9ncm91cHMgcHJlc2VudCBpbiBjdGVub3Bob3JlcyBhbmQgYmlsYXRlcmlhbnMgYnV0IG5vdCBpbiBtZWR1c296b2FucwoKYGBge3J9Cm9uZV9YX29uZV9ZX25vbmVfWigibW5lbWlvcHNpc19sZWlkeWkiLCBiaWxhdGVyaWEsIG1lZHVzb3pvYSkKYGBgCgoqKioKCiMjIyMjIE9ydGhvZ3JvdXBzIHByZXNlbnQgaW4gY3Rlbm9waG9yZXMgYW5kIGF0IGxlYXN0IG9uZSBiaWxhdGVyaWFuIGJ1dCBhYnNlbnQgaW4gaHlkcm96b2FucwoKYGBge3J9Cm9uZV9YX29uZV9ZX25vbmVfWigibW5lbWlvcHNpc19sZWlkeWkiLCBiaWxhdGVyaWEsIGh5ZHJvem9hKQpgYGAKKioqCiMjIyMjIE9ydGhvZ3JvdXBzIHByZXNlbnQgaW4gY3Rlbm9waG9yZXMgYW5kIGJpbGF0ZXJpYW5zIGJ1dCBhYnNlbnQgaW4gSHlkcmFjdGluaWEKCmBgYHtyfQpvbmVfWF9vbmVfWV9ub25lX1ooIm1uZW1pb3BzaXNfbGVpZHlpIiwgYmlsYXRlcmlhLCBoeWRyYWN0aW5pYSkKYGBgCioqKgojIyMjIyBPcnRob2dyb3VwcyBwcmVzZW50IGluIGN0ZW5vcGhvcmVzIGFuZCBiaWxhdGVyaWFucyBidXQgYWJzZW50IGluIEh5ZHJhY3RpbmlhIGVjaGluYXRhCgpgYGB7cn0Kb25lX1hfb25lX1lfbm9uZV9aKCJtbmVtaW9wc2lzX2xlaWR5aSIsIGJpbGF0ZXJpYSwgImh5ZHJhY3RpbmlhX2VjaGluYXRhIikKYGBgCgoqKioKCiMjIyMjIE9ydGhvZ3JvdXBzIHByZXNlbnQgaW4gY3Rlbm9waG9yZXMgYW5kIGJpbGF0ZXJpYW5zIGJ1dCBhYnNlbnQgaW4gSHlkcmFjdGluaWEgc3ltYmlvbG9uZ2ljYXJwdXMKCmBgYHtyfQpvbmVfWF9vbmVfWV9ub25lX1ooIm1uZW1pb3BzaXNfbGVpZHlpIiwgYmlsYXRlcmlhLCAiaHlkcmFjdGluaWFfc3ltYmlvbG9uZ2ljYXJwdXMiKQpgYGAKCiMjIyMjIE9ydGhvZ3JvdXBzIHByZXNlbnQgaW4gY3Rlbm9waG9yZXMgYW5kIGJpbGF0ZXJpYW5zIGJ1dCBhYnNlbnQgaW4gQ2x5dGlhIAoKYGBge3J9Cm9uZV9YX29uZV9ZX25vbmVfWigibW5lbWlvcHNpc19sZWlkeWkiLCBiaWxhdGVyaWEsICJjbHl0aWFfaGVtaXNwaGFlcmljYSIpCmBgYAoKKioqCgojIyMjIyBPcnRob2dyb3VwcyBwcmVzZW50IGluIGN0ZW5vcGhvcmVzIGFuZCBiaWxhdGVyaWFucyBidXQgYWJzZW50IGluIEh5ZHJhCgpgYGB7cn0Kb25lX1hfb25lX1lfbm9uZV9aKCJtbmVtaW9wc2lzX2xlaWR5aSIsIGJpbGF0ZXJpYSwgImh5ZHJhX21hZ25pcGFwaWxsYXRhIikKYGBgCioqKgojIyMjIFBvcmlmZXJhIHF1ZXJpZXMKKioqCiMjIyMjIE9ydGhvZ3JvdXBzIHByZXNlbnQgaW4gQW1waGltZWRvbiBhbmQgYXQgbGVhc3Qgb25lIGJpbGF0ZXJpYW4gYnV0IGFic2VudCBpbiBjbmlkYXJpYW5zCgpgYGB7cn0Kb25lX1hfb25lX1lfbm9uZV9aKCJhbXBoaW1lZG9uX3F1ZWVuc2xhbmRpY2EiLCBiaWxhdGVyaWEsIGNuaWRhcmlhKQpgYGAKCioqKgoKIyMjIyMgT3J0aG9ncm91cHMgcHJlc2VudCBpbiBBbXBoaW1lZG9uIGFuZCBhdCBsZWFzdCBvbmUgYmlsYXRlcmlhbiBidXQgYWJzZW50IGluIG1lZHVzb3pvYW5zCgpgYGB7cn0Kb25lX1hfb25lX1lfbm9uZV9aKCJhbXBoaW1lZG9uX3F1ZWVuc2xhbmRpY2EiLCBiaWxhdGVyaWEsIG1lZHVzb3pvYSkKYGBgCioqKgojIyMjIyBPcnRob2dyb3VwcyBwcmVzZW50IGluIEFtcGhpbWVkb24gYW5kIGF0IGxlYXN0IG9uZSBiaWxhdGVyaWFuIGJ1dCBhYnNlbnQgaW4gYm90aCBIeWRyYWN0aW5pYQoKYGBge3J9Cm9uZV9YX29uZV9ZX25vbmVfWigiYW1waGltZWRvbl9xdWVlbnNsYW5kaWNhIiwgYmlsYXRlcmlhLCBoeWRyYWN0aW5pYSkKYGBgCioqKgoKIyMjIyMgIE9ydGhvZ3JvdXBzIHByZXNlbnQgaW4gQW1waGltZWRvbiBhbmQgYXQgbGVhc3Qgb25lIGJpbGF0ZXJpYW4gYnV0IGFic2VudCBpbiBvbmUgaHlkcm96b2FuCgpgYGB7cn0KZm9yICh0YXhvbiBpbiBjKCJoeWRyYWN0aW5pYV9lY2hpbmF0YSIsICJoeWRyYWN0aW5pYV9zeW1iaW9sb25naWNhcnB1cyIsICJjbHl0aWFfaGVtaXNwaGFlcmljYSIsICJoeWRyYV9tYWduaXBhcGlsbGF0YSIpKXsKICBvbmVfWF9vbmVfWV9ub25lX1ooImFtcGhpbWVkb25fcXVlZW5zbGFuZGljYSIsIGJpbGF0ZXJpYSwgdGF4b24pCiAgY2F0KCJcblxuIikKfQpgYGAKKioqCiMjIyMgUGxhY296b2EgcXVlcmllcwoKYGBge3J9CnBsYWNvem9hIDwtIHNlbGVjdF9jbGFkZSgiUGxhY296b2EiKQpwbGFjb3pvYQpgYGAKCioqKgoKIyMjIyMgIE9ydGhvZ3JvdXBzIHByZXNlbnQgaW4gYXQgbGVhc3Qgb25lIHBsYWNvem9hbiBhbmQgb25lIGJpbGF0ZXJpYW4gYnV0IGFic2VudCBpbiBhbGwgY25pZGFyaWFucwoKYGBge3J9Cm9uZV9YX29uZV9ZX25vbmVfWihwbGFjb3pvYSwgYmlsYXRlcmlhLCBjbmlkYXJpYSkKYGBgCgoqKioKCiMjIyMjIE9ydGhvZ3JvdXBzIHByZXNlbnQgaW4gYXQgbGVhc3Qgb25lIHBsYWNvem9hbiBhbmQgb25lIGJpbGF0ZXJpYW4gYnV0IGFic2VudCBpbiBhbGwgbWVkdXNvem9hbnMKCmBgYHtyfQpvbmVfWF9vbmVfWV9ub25lX1oocGxhY296b2EsIGJpbGF0ZXJpYSwgbWVkdXNvem9hKQpgYGAKKioqCgojIyMjIyBPcnRob2dyb3VwcyBwcmVzZW50IGluIGF0IGxlYXN0IG9uZSBwbGFjb3pvYW4gYW5kIG9uZSBiaWxhdGVyaWFuIGJ1dCBhYnNlbnQgaW4gYWxsIG1lZHVzb3pvYW5zCgpgYGB7cn0Kb25lX1hfb25lX1lfbm9uZV9aKHBsYWNvem9hLCBiaWxhdGVyaWEsIGh5ZHJvem9hKQpgYGAKCioqKgoKIyMjIyMgT3J0aG9ncm91cHMgcHJlc2VudCBpbiBhdCBsZWFzdCBvbmUgcGxhY296b2FuIGFuZCBvbmUgYmlsYXRlcmlhbiBidXQgYWJzZW50IGluIGJvdGggSHlkcmFjdGluaWEKCgpgYGB7cn0Kb25lX1hfb25lX1lfbm9uZV9aKHBsYWNvem9hLCBiaWxhdGVyaWEsIGh5ZHJhY3RpbmlhKQpgYGAKKioqCiMjIyMjIE9ydGhvZ3JvdXBzIHByZXNlbnQgaW4gYXQgbGVhc3Qgb25lIHBsYWNvem9hbiBhbmQgb25lIGJpbGF0ZXJpYW4gYnV0IGFic2VudCBpbiBvbmUgaHlkcm96b2FuCgpgYGB7cn0KZm9yICh0YXhvbiBpbiBjKCJoeWRyYWN0aW5pYV9lY2hpbmF0YSIsICJoeWRyYWN0aW5pYV9zeW1iaW9sb25naWNhcnB1cyIsICJjbHl0aWFfaGVtaXNwaGFlcmljYSIsICJoeWRyYV9tYWduaXBhcGlsbGF0YSIpKXsKICBvbmVfWF9vbmVfWV9ub25lX1oocGxhY296b2EsIGJpbGF0ZXJpYSwgdGF4b24pCiAgY2F0KCJcblxuIikKfQpgYGAKCioqKgoKIyMjIyBQb3RlbnRpYWwgbG9zc2VzIHNpbmNlIHNwbGl0IHdpdGggQ3Rlbm9vcGhvcmEgKyBzcG9uZ2UgKyBQbGFjb3pvYSAoQ1NQKQoKYGBge3J9CmNzcCA8LSBmdXNlX3RheGEoYygiQ3Rlbm9waG9yYSIsICJQb3JpZmVyYSIsICJQbGFjb3pvYSIpKQpjc3AKYGBgCgoqKioKCiMjIyMjIE9ydGhvZ3JvdXBzIHByZXNlbnQgaW4gYXQgbGVhc3Qgb25lIG9mIENTUCBhbmQgb25lIGJpbGF0ZXJpYW4gYnV0IGFic2VudCBpbiBhbGwgY25pZGFyaWFucwoKYGBge3J9Cm9uZV9YX29uZV9ZX25vbmVfWihjc3AsIGJpbGF0ZXJpYSwgY25pZGFyaWEpCmBgYAoKKioqCgojIyMjIyBPcnRob2dyb3VwcyBwcmVzZW50IGluIGF0IGxlYXN0IG9uZSBvZiBDU1AgYW5kIG9uZSBiaWxhdGVyaWFuIGJ1dCBhYnNlbnQgaW4gYWxsIG1lZHVzb3pvYW5zCgoKYGBge3J9Cm9uZV9YX29uZV9ZX25vbmVfWihjc3AsIGJpbGF0ZXJpYSwgbWVkdXNvem9hKQpgYGAKCioqKgoKIyMjIyMgT3J0aG9ncm91cHMgcHJlc2VudCBpbiBhdCBsZWFzdCBvbmUgb2YgQ1NQIGFuZCBvbmUgYmlsYXRlcmlhbiBidXQgYWJzZW50IGluIGFsbCBoeWRyb3pvYW5zCgpgYGB7cn0Kb25lX1hfb25lX1lfbm9uZV9aKGNzcCwgYmlsYXRlcmlhLCBoeWRyb3pvYSkKYGBgCioqKgojIyMjIyBPcnRob2dyb3VwcyBwcmVzZW50IGluIGF0IGxlYXN0IG9uZSBvZiBDU1AgYW5kIG9uZSBiaWxhdGVyaWFuIGJ1dCBhYnNlbnQgaW4gYm90aCBoeWRyYWN0aW5pYQoKYGBge3J9Cm9uZV9YX29uZV9ZX25vbmVfWihjc3AsIGJpbGF0ZXJpYSwgaHlkcmFjdGluaWEpCmBgYAoKIyMjIyMgT3J0aG9ncm91cHMgcHJlc2VudCBpbiBhdCBsZWFzdCBvbmUgb2YgQ1NQIGFuZCBvbmUgYmlsYXRlcmlhbiBidXQgYWJzZW50IGluIG9uZSBoeWRyb3pvYW4KCmBgYHtyfQpmb3IgKHRheG9uIGluIGMoImh5ZHJhY3RpbmlhX2VjaGluYXRhIiwgImh5ZHJhY3RpbmlhX3N5bWJpb2xvbmdpY2FycHVzIiwgImNseXRpYV9oZW1pc3BoYWVyaWNhIiwgImh5ZHJhX21hZ25pcGFwaWxsYXRhIikpewogIG9uZV9YX29uZV9ZX25vbmVfWihjc3AsIGJpbGF0ZXJpYSwgdGF4b24pCiAgY2F0KCJcblxuIikKfQpgYGAKCioqKgoKIyMjIyMgT3J0aG9ncm91cHMgcHJlc2VudCBpbiBhdCBsZWFzdCBvbmUgb2YgQ1NQIGJ1dCBhYnNlbnQgaW4gYWxsIGNuaWRhcmlhbnMKCmBgYHtyfQpvbmVfWF9ub25lX1koY3NwLCBjbmlkYXJpYSkKYGBgCgoqKioKCiMjIyMjICBPcnRob2dyb3VwcyBwcmVzZW50IGluIGF0IGxlYXN0IG9uZSBvZiBDU1AgYnV0IGFic2VudCBpbiBhbGwgbWVkdXNvem9hbnMKCmBgYHtyfQpvbmVfWF9ub25lX1koY3NwLCBtZWR1c296b2EpCmBgYAoKKioqCgojIyMjIyAgT3J0aG9ncm91cHMgcHJlc2VudCBpbiBhdCBsZWFzdCBvbmUgb2YgQ1NQIGJ1dCBhYnNlbnQgaW4gYWxsIGh5ZHJvem9hbnMKCmBgYHtyfQpvbmVfWF9ub25lX1koY3NwLCBoeWRyb3pvYSkKYGBgCgoqKioKCiMjIyMjICBPcnRob2dyb3VwcyBwcmVzZW50IGluIGF0IGxlYXN0IG9uZSBvZiBDU1AgYnV0IGFic2VudCBpbiBib3RoIEh5ZHJhY3RpbmlhCgpgYGB7cn0Kb25lX1hfbm9uZV9ZKGNzcCwgaHlkcmFjdGluaWEpCmBgYAoKIyMjIyMgIE9ydGhvZ3JvdXBzIHByZXNlbnQgaW4gYXQgbGVhc3Qgb25lIG9mIENTUCBidXQgYWJzZW50IGluIG9uZSBoeWRyb3pvYW4gCgpgYGB7cn0KZm9yICh0YXhvbiBpbiBjKCJoeWRyYWN0aW5pYV9lY2hpbmF0YSIsICJoeWRyYWN0aW5pYV9zeW1iaW9sb25naWNhcnB1cyIsICJoeWRyYV9tYWduaXBhcGlsbGF0YSIsICJjbHl0aWFfaGVtaXNwaGFlcmljYSIpKXsKICBvbmVfWF9ub25lX1koY3NwLCB0YXhvbikKICBjYXQoIlxuXG4iKQp9CmBgYAoKKioqCgojIyMjIFBvdGVudGlhbCBsb3NzZXMgc2luY2Ugc3BsaXQgYmV0d2VlbiAobm9uLW1ldGF6b2FucyArIEN0ZW5vcGhvcmEgKyBQb3JpZmVyYSArIFBsYWNvem9hKSAgYW5kIChDbmlkYXJpYStCaWxhdGVyaWEpCgpgYGB7cn0KYW5jZXN0b3IgPC0gZnVzZV90YXhhKGMoIk5vbi1tZXRhem9hbnMiLCJDdGVub3Bob3JhIiwgIlBvcmlmZXJhIiwgIlBsYWNvem9hIikpCmFuY2VzdG9yCmBgYAoqKioKIyMjIyMgT3J0aG9ncm91cHMgcHJlc2VudCBpbiBhdCBsZWFzdCBvbmUgZWFybHktYnJhbmNoaW5nIHRheG9uIGFuZCBhYnNlbnQgaW4gYWxsIGNuaWRhcmlhbnMKCmBgYHtyfQpvbmVfWF9ub25lX1koYW5jZXN0b3IsIGNuaWRhcmlhKQpgYGAKKioqCiMjIyMjIE9ydGhvZ3JvdXBzIHByZXNlbnQgaW4gYXQgbGVhc3Qgb25lIGVhcmx5LWJyYW5jaGluZyB0YXhvbiBhbmQgYXQgbGVhc3Qgb25lIGNuaWRhcmlhbiBidXQgYWJzZW50IGluIEguIGVjaGluYXRhCgpgYGB7cn0Kb25lX1hfb25lX1lfbm9uZV9aKGFuY2VzdG9yLCBjbmlkYXJpYSwgImh5ZHJhY3RpbmlhX2VjaGluYXRhIikKYGBgCioqKgojIyMjIyBPcnRob2dyb3VwcyBwcmVzZW50IGluIGF0IGxlYXN0IG9uZSBlYXJseS1icmFuY2hpbmcgdGF4b24gYW5kIGF0IGxlYXN0IG9uZSBjbmlkYXJpYW4gYnV0IGFic2VudCBpbiBILiBzeW1iaW9vbmdpY2FycHVzCgpgYGB7cn0Kb25lX1hfb25lX1lfbm9uZV9aKGFuY2VzdG9yLCBjbmlkYXJpYSwgImh5ZHJhY3RpbmlhX3N5bWJpb2xvbmdpY2FycHVzIikKYGBgCgoqKioKIyMjIyMgT3J0aG9ncm91cHMgcHJlc2VudCBpbiBhdCBsZWFzdCBvbmUgZWFybHktYnJhbmNoaW5nIHRheG9uIGFuZCBhdCBsZWFzdCBvbmUgY25pZGFyaWFuIGJ1dCBhYnNlbnQgaW4gSHlkcmEKCgpgYGB7cn0Kb25lX1hfb25lX1lfbm9uZV9aKGFuY2VzdG9yLCBjbmlkYXJpYSwgImh5ZHJhX21hZ25pcGFwaWxsYXRhIikKYGBgCgoqKioKCiMjIyMjIE9ydGhvZ3JvdXBzIHByZXNlbnQgaW4gYXQgbGVhc3Qgb25lIGVhcmx5LWJyYW5jaGluZyB0YXhvbiBhbmQgYXQgbGVhc3Qgb25lIGNuaWRhcmlhbiBidXQgYWJzZW50IGluIGJvdGggSHlkcmFjdGluaWEKCgpgYGB7cn0Kb25lX1hfb25lX1lfbm9uZV9aKGFuY2VzdG9yLCBjbmlkYXJpYSwgaHlkcmFjdGluaWEpCmBgYAoKKioqCgojIyMjIyBPcnRob2dyb3VwcyBwcmVzZW50IGluIGF0IGxlYXN0IG9uZSBlYXJseS1icmFuY2hpbmcgdGF4b24gYW5kIGF0IGxlYXN0IG9uZSBjbmlkYXJpYW4gYnV0IGFic2VudCBpbiBDbHl0aWEKCmBgYHtyfQpvbmVfWF9vbmVfWV9ub25lX1ooYW5jZXN0b3IsIGNuaWRhcmlhLCAiY2x5dGlhX2hlbWlzcGhhZXJpY2EiKQpgYGAKCioqKgojIyMjIyBPcnRob2dyb3VwcyBwcmVzZW50IGluIGF0IGxlYXN0IG9uZSBlYXJseS1icmFuY2hpbmcgdGF4b24gYW5kIEh5ZHJhIGJ1dCBhYnNlbnQgaW4gYm90aCBIeWRyYWN0aW5pYQoKYGBge3J9Cm9uZV9YX29uZV9ZX25vbmVfWihhbmNlc3RvciwgImh5ZHJhX21hZ25pcGFwaWxsYXRhIiwgaHlkcmFjdGluaWEpCmBgYAoKIyMjIyMgT3J0aG9ncm91cHMgcHJlc2VudCBpbiBhdCBsZWFzdCBvbmUgZWFybHktYnJhbmNoaW5nIHRheG9uIGFuZCBvbmUgSHlkcmFjdGluaWEgYnV0IGFic2VudCBpbiBIeWRyYQoKYGBge3J9Cm9uZV9YX29uZV9ZX25vbmVfWihhbmNlc3RvciwgaHlkcmFjdGluaWEsICJoeWRyYV9tYWduaXBhcGlsbGF0YSIpCmBgYAoKKioqCgojIyMjIyBPcnRob2dyb3VwcyBwcmVzZW50IGluIGF0IGxlYXN0IG9uZSBlYXJseS1icmFuY2hpbmcgdGF4b24gYW5kIG9uZSBiaWxhdGVyaWFuIGJ1dCBhYnNlbnQgaW4gYWxsIGNuaWRhcmlhbnMJCgpgYGB7cn0Kb25lX1hfb25lX1lfbm9uZV9aKGFuY2VzdG9yLCBiaWxhdGVyaWEsIGNuaWRhcmlhKQpgYGAKCioqKgojIyMjIyBPcnRob2dyb3VwcyBwcmVzZW50IGluIGF0IGxlYXN0IG9uZSBlYXJseS1icmFuY2hpbmcgdGF4b24gYW5kIG9uZSBiaWxhdGVyaWFuIGJ1dCBhYnNlbnQgaW4gYm90aCBIeWRyYWN0aW5pYQoKYGBge3J9Cm9uZV9YX29uZV9ZX2xvc3Rfb25lX1ooYW5jZXN0b3IsIGJpbGF0ZXJpYSwgaHlkcmFjdGluaWEpCmBgYAoKKioqCiMjIyMjIE9ydGhvZ3JvdXBzIHByZXNlbnQgaW4gYXQgbGVhc3Qgb25lIGVhcmx5LWJyYW5jaGluZyB0YXhvbiBhbmQgb25lIGJpbGF0ZXJpYW4gYnV0IGFic2VudCBpbiBvbmUgaHlkcm96b2FuCgpgYGB7cn0KZm9yIChzcGVjaWVzIGluIGh5ZHJvem9hKXsKICBvbmVfWF9vbmVfWV9ub25lX1ooYW5jZXN0b3IsIGJpbGF0ZXJpYSwgc3BlY2llcykKICBjYXQoIlxuXG4iKQp9IApgYGAKCioqKgoKIyMjIyMgT3J0aG9ncm91cHMgcHJlc2VudCBpbiBhdCBsZWFzdCBvbmUgZWFybHktYnJhbmNoaW5nIHRheG9uIGFuZCBvbmUgYmlsYXRlcmlhbiBidXQgYWJzZW50IGluIGFsbCBoeWRyb3pvYW5zCgpgYGB7cn0Kb25lX1hfb25lX1lfbm9uZV9aKGFuY2VzdG9yLCBiaWxhdGVyaWEsIGh5ZHJvem9hKQpgYGAKKioqCgojIyMjIyBPcnRob2dyb3VwcyBwcmVzZW50IGluIGF0IGxlYXN0IG9uZSBlYXJseS1icmFuY2hpbmcgdGF4b24gYW5kIG9uZSBiaWxhdGVyaWFuIGJ1dCBhYnNlbnQgaW4gYWxsIG1lZHVzb3pvYW5zCgpgYGB7cn0Kb25lX1hfb25lX1lfbm9uZV9aKGFuY2VzdG9yLCBiaWxhdGVyaWEsIG1lZHVzb3pvYSkKYGBgCioqKgoKIyMjIyMgT3J0aG9ncm91cHMgcHJlc2VudCBpbiBhdCBsZWFzdCBvbmUgZWFybHktYnJhbmNoaW5nIHRheG9uIGFuZCBvbmUgYmlsYXRlcmlhbiBidXQgYWJzZW50IGluIE5lbWF0b3N0ZWxsYQoKYGBge3J9Cm9uZV9YX29uZV9ZX25vbmVfWihhbmNlc3RvciwgYmlsYXRlcmlhLCAibmVtYXRvc3RlbGxhX3ZlY3RlbnNpcyIpCmBgYAoKCioqKgoKIyMjIyMgT3J0aG9ncm91cHMgcHJlc2VudCBpbiBhdCBsZWFzdCBvbmUgZWFybHktYnJhbmNoaW5nIHRheG9uIGFuZCBvbmUgYmlsYXRlcmlhbgoKYGBge3J9Cm9uZV9YX29uZV9ZX25vbmVfWihiaWxhdGVyaWEsIGFuY2VzdG9yKQpgYGAKCioqKgoKIyMjIyBHZW5lIGNvdW50cyBpbiBjbmlkYXJpYW4tc3BlY2lmaWMgb3J0aG9ncm91cHMKCmBgYHtyfQpjb3VudF9vcnRob2dyb3VwcyhjbmlkYXJpYSkKYGBgCgoKCg==
