# Supplementary material for: The genome of the colonial hydroid Hydractinia reveals that their stem cells use a toolkit of evolutionarily shared genes with all animals
Source: Genome Res. 2024 Mar;34(3):498–513. doi: 10.1101/gr.278382.123 (PMC11067881; doi:10.1101/gr.278382.123)
Supplement: Supplement 10 [file Supplemental_Data.zip › Supplemental_Data/Supplemental_Data_S14.html]

Supplemental\_Data\_S14.knit


<!DOCTYPE html>

Orthogroup analysis - Phylum-specific and unassigned genes


Code 

- Show All Code
- Hide All Code
- Download Rmd

# Orthogroup analysis - Phylum-specific and unassigned genes

##### Goal: Calculate number of phylum-specific and unassigned genes, and plot their distribution across species


```
library(tidyverse)
```


```
Registered S3 method overwritten by 'dplyr':
  method           from
  print.rowwise_df     
Registered S3 methods overwritten by 'dbplyr':
  method         from
  print.tbl_lazy     
  print.tbl_sql      
[30m── [1mAttaching packages[22m ───────────────────────────────────────────────────────────────────────── tidyverse 1.3.0 ──[39m
[30m[32m✓[30m [34mggplot2[30m 3.2.1     [32m✓[30m [34mpurrr  [30m 0.3.3
[32m✓[30m [34mtibble [30m 2.1.3     [32m✓[30m [34mdplyr  [30m 0.8.3
[32m✓[30m [34mtidyr  [30m 1.0.0     [32m✓[30m [34mstringr[30m 1.4.0
[32m✓[30m [34mreadr  [30m 1.3.1     [32m✓[30m [34mforcats[30m 0.4.0[39m
[30m── [1mConflicts[22m ──────────────────────────────────────────────────────────────────────────── tidyverse_conflicts() ──
[31mx[30m [34mdplyr[30m::[32mfilter()[30m masks [34mstats[30m::filter()
[31mx[30m [34mdplyr[30m::[32mlag()[30m    masks [34mstats[30m::lag()[39m
```


```
library(viridis)
```


```
Loading required package: viridisLite
```


---

#### Read orthogroup table


```
ortho_counts <- read.table("Orthogroups.GeneCount_dec_20.csv")
ortho_binary <- ortho_counts[,1:49] #Remove the Totals column
ortho_binary[ortho_binary >= 1] <- 1 #Convert to presence/absence
```


---

#### Read table of phyla


```
taxonomy_phyla <- read_csv("taxonomy_run3_phyla.csv")
```


```
Parsed with column specification:
cols(
  species = [31mcol_character()[39m,
  clade = [31mcol_character()[39m,
  phylum = [31mcol_character()[39m
)
```


```
taxonomy_phyla$phylum <- factor(taxonomy_phyla$phylum)
#taxonomy_phyla$species <- factor(taxonomy_phyla$species)
taxonomy_phyla
```


---

#### Calculate number of phylum-specific genes for each species


```
phylum_specific_genes <- data.frame(species = character(0), phylum_specific_genes = double(0))
for (i in seq_along(levels(taxonomy_phyla$phylum))){
  phylum <- levels(taxonomy_phyla$phylum)[i]
  species_in <- taxonomy_phyla[taxonomy_phyla$phylum == phylum, "species"]
  species_in <- species_in$species
  select_species <- ortho_binary %>% select(species_in)
  other_species <- ortho_binary %>% select(-species_in)
  specific_ortho_at_least_one <- row.names(ortho_binary[rowSums(select_species) >= 1  & rowSums(other_species) == 0,])
  phylum_specific_counts <- numeric(0)
  for (i in 1: length(species_in)){
  phylum_specific_counts[i] <- (sum(ortho_counts[specific_ortho_at_least_one, species_in[i]]))
  }
  phylum_df <- data.frame(species = species_in, phylum_specific_genes = phylum_specific_counts)
  phylum_specific_genes <- rbind(phylum_specific_genes, phylum_df)
}
phylum_specific_genes
```


---

#### Read unassigned genes data


```
stats_per_species <- read_tsv("Statistics_PerSpecies_Dec_20.csv")
stats_per_species
```


```
species <- colnames(stats_per_species)
species <- species[2:50]
total_genes <- stats_per_species %>% slice(1) %>% unlist(., use.names=FALSE)
total_genes <- as.numeric(total_genes[2:50])
unassigned_genes <- stats_per_species %>% slice(3) %>% unlist(., use.names=FALSE)
unassigned_genes <- as.numeric(unassigned_genes[2:50])
unassigned_genes
```


```
 [1]  5278  1201 16167  4339  2543  5260  7513  6581  2050  1654  4397   819  2527  5314  1383  2966  1124   815
[19]  6451  8696   538  2092  7236  2919  1246  6818  2414  5716  4571  1925  5987  4343  2014  8375  1744  1005
[37]  3093   550 12100  4972  2081  2012  2775  2040  8586  6924  1635   972  1277
```


```
percent_unassigned <- stats_per_species %>% slice(5) %>% unlist(., use.names=FALSE)
percent_unassigned <- as.numeric(percent_unassigned[2:50])
unassigned <- tibble(species, total_genes, unassigned_genes, percent_unassigned)
unassigned
```

#### Reorder levels of species factor according to original table (for plotting)


```
phylum_specific_genes$species <- factor(phylum_specific_genes$species, levels = rev(taxonomy_phyla$species))
unassigned$species <- factor(unassigned$species, levels = rev(taxonomy_phyla$species))
```


---

#### Combine phylum-specific and unassigned


```
phylum_spec_and_unassigned <- left_join(phylum_specific_genes, unassigned, by = "species")
phylum_spec_and_unassigned
```


---

#### calculate “other genes” counts (neither phylum-specific or unassigned)


```
df_new <- phylum_spec_and_unassigned %>%
  mutate(other_genes = total_genes - phylum_specific_genes - unassigned_genes) %>% 
  select(species, phylum_specific_genes, unassigned_genes, other_genes)
df_new
```


---

#### Gather variables for plotting


```
df_long <- df_new %>% 
  gather(`phylum_specific_genes`, `unassigned_genes`, `other_genes`,
         key = "gene_category",
         value = "gene_count") %>%
  mutate(gene_category = factor(gene_category, levels = c("other_genes","phylum_specific_genes", "unassigned_genes" )))
df_long
```


---

#### Plot


```
gene_count_plot <- ggplot(data = df_long, aes(x = species, y = gene_count, fill = gene_category)) +
  geom_bar(stat="identity") +
  coord_flip()
```


---

#### Make human-readable species names, clean up labels, change colors, and color species names by phylum.


```
species_names <- gsub("_", " ", taxonomy_phyla$species)
species_names <- paste(toupper(substr(species_names, 1, 1)), substr(species_names, 2, nchar(species_names)), sep="")
species_names
```


```
 [1] "Danio rerio"                   "Xenopus tropicalis"            "Gallus gallus"                
 [4] "Homo sapiens"                  "Ciona intestinalis"            "Branchiostoma floridae"       
 [7] "Saccoglossus kowalevskii"      "Acanthaster planci"            "Pristionchus pacificus"       
[10] "Caenorhabditis elegans"        "Drosophila melanogaster"       "Tribolium castaneum"          
[13] "Daphnia pulex"                 "Ixodes scapularis"             "Lottia gigantea"              
[16] "Octopus bimaculoides"          "Phoronis australis"            "Notospermus geniculatus"      
[19] "Schistosoma mansoni"           "Schmidtea mediterranea"        "Capitella teleta"             
[22] "Helobdella robusta"            "Exaiptasia pallida"            "Nematostella vectensis"       
[25] "Orbicella faveolata"           "Acropora digitifera"           "Pocillopora damicornis"       
[28] "Renilla muelleri"              "Dendronephthya gigantea"       "Aurelia aurita atlantic"      
[31] "Aurelia aurita pacific"        "Nemopilema nomurai"            "Hydractinia echinata"         
[34] "Hydractinia symbiolongicarpus" "Hydra magnipapillata"          "Clytia hemisphaerica"         
[37] "Morbakka virulenta"            "Kudoa iwatai"                  "Hofstenia miamia"             
[40] "Hoilungia hongkongensis"       "Trichoplax adhaerens"          "Amphimedon queenslandica"     
[43] "Mnemiopsis leidyi"             "Capsaspora owczarzaki"         "Creolimax fragrantissima"     
[46] "Monosiga brevicolis"           "Saccharomyces cerevisiae"      "Salpingoeca rosetta"          
[49] "Sphaeroforma arctica"
```


```
gene_count_plot +
  scale_fill_viridis(option="viridis",discrete = TRUE, labels = c("Other", "Phylum-specific", "Unassigned")) + # Color + legend names spelled out
  guides(fill = guide_legend(reverse = TRUE)) + # Reverse legend labels so that other is not first
  labs(y = "Gene count") + # y axis label
  scale_x_discrete(name = "Species", labels = rev(species_names)) +  # clean up x-axis label
  theme(axis.text.y  = element_text(size=7, color = rev(taxonomy_phyla$phylum))) # color species names by phylum (ugly but useful)
```


---

### Plot the same data as percentage


```
df_percent <- phylum_spec_and_unassigned %>% 
  mutate(percent_phylum_specific = (phylum_specific_genes/total_genes)*100, 
         percent_other_genes = (total_genes - phylum_specific_genes - unassigned_genes)*100/total_genes) %>% 
  select(species, percent_phylum_specific, percent_unassigned, percent_other_genes)
df_percent
```


```
df_percent_long <- df_percent %>% 
  gather(`percent_phylum_specific`, `percent_unassigned`, `percent_other_genes`,
         key = "gene_category",
         value = "percent_genes") %>%
  mutate(gene_category = factor(gene_category, levels = c("percent_other_genes","percent_phylum_specific", "percent_unassigned" )))
df_percent_long
```


```
percent_plot <- ggplot(data = df_percent_long, aes(x = species, y = percent_genes, fill = gene_category)) +
  geom_bar(stat="identity") +
  coord_flip()
```


```
percent_plot +
  scale_fill_viridis(option="viridis",discrete = TRUE, labels = c("Other", "Phylum-specific", "Unassigned")) +
  labs(y = "%") +
  guides(fill = guide_legend(reverse = TRUE)) +
  scale_x_discrete(name = "Species", labels = rev(species_names)) +
  theme(axis.text.y  = element_text(size=7, color = rev(taxonomy_phyla$phylum)))
```

LS0tCnRpdGxlOiAiT3J0aG9ncm91cCBhbmFseXNpcyAtIFBoeWx1bS1zcGVjaWZpYyBhbmQgdW5hc3NpZ25lZCBnZW5lcyIKb3V0cHV0OiBodG1sX25vdGVib29rCi0tLQoKIyMjIyMgR29hbDogQ2FsY3VsYXRlIG51bWJlciBvZiBwaHlsdW0tc3BlY2lmaWMgYW5kIHVuYXNzaWduZWQgZ2VuZXMsIGFuZCBwbG90IHRoZWlyIGRpc3RyaWJ1dGlvbiBhY3Jvc3Mgc3BlY2llcwoKYGBge3J9CmxpYnJhcnkodGlkeXZlcnNlKQpsaWJyYXJ5KHZpcmlkaXMpCmBgYAoKKioqCgojIyMjIFJlYWQgb3J0aG9ncm91cCB0YWJsZQoKYGBge3J9Cm9ydGhvX2NvdW50cyA8LSByZWFkLnRhYmxlKCJPcnRob2dyb3Vwcy5HZW5lQ291bnRfZGVjXzIwLmNzdiIpCm9ydGhvX2JpbmFyeSA8LSBvcnRob19jb3VudHNbLDE6NDldICNSZW1vdmUgdGhlIFRvdGFscyBjb2x1bW4Kb3J0aG9fYmluYXJ5W29ydGhvX2JpbmFyeSA+PSAxXSA8LSAxICNDb252ZXJ0IHRvIHByZXNlbmNlL2Fic2VuY2UKYGBgCgoqKioKCiMjIyMgUmVhZCB0YWJsZSBvZiBwaHlsYQoKYGBge3J9CnRheG9ub215X3BoeWxhIDwtIHJlYWRfY3N2KCJ0YXhvbm9teV9ydW4zX3BoeWxhLmNzdiIpCnRheG9ub215X3BoeWxhJHBoeWx1bSA8LSBmYWN0b3IodGF4b25vbXlfcGh5bGEkcGh5bHVtKQojdGF4b25vbXlfcGh5bGEkc3BlY2llcyA8LSBmYWN0b3IodGF4b25vbXlfcGh5bGEkc3BlY2llcykKdGF4b25vbXlfcGh5bGEKYGBgCioqKgojIyMjIENhbGN1bGF0ZSBudW1iZXIgb2YgcGh5bHVtLXNwZWNpZmljIGdlbmVzIGZvciBlYWNoIHNwZWNpZXMKCmBgYHtyfQpwaHlsdW1fc3BlY2lmaWNfZ2VuZXMgPC0gZGF0YS5mcmFtZShzcGVjaWVzID0gY2hhcmFjdGVyKDApLCBwaHlsdW1fc3BlY2lmaWNfZ2VuZXMgPSBkb3VibGUoMCkpCmZvciAoaSBpbiBzZXFfYWxvbmcobGV2ZWxzKHRheG9ub215X3BoeWxhJHBoeWx1bSkpKXsKICBwaHlsdW0gPC0gbGV2ZWxzKHRheG9ub215X3BoeWxhJHBoeWx1bSlbaV0KICBzcGVjaWVzX2luIDwtIHRheG9ub215X3BoeWxhW3RheG9ub215X3BoeWxhJHBoeWx1bSA9PSBwaHlsdW0sICJzcGVjaWVzIl0KICBzcGVjaWVzX2luIDwtIHNwZWNpZXNfaW4kc3BlY2llcwogIHNlbGVjdF9zcGVjaWVzIDwtIG9ydGhvX2JpbmFyeSAlPiUgc2VsZWN0KHNwZWNpZXNfaW4pCiAgb3RoZXJfc3BlY2llcyA8LSBvcnRob19iaW5hcnkgJT4lIHNlbGVjdCgtc3BlY2llc19pbikKICBzcGVjaWZpY19vcnRob19hdF9sZWFzdF9vbmUgPC0gcm93Lm5hbWVzKG9ydGhvX2JpbmFyeVtyb3dTdW1zKHNlbGVjdF9zcGVjaWVzKSA+PSAxICAmIHJvd1N1bXMob3RoZXJfc3BlY2llcykgPT0gMCxdKQogIHBoeWx1bV9zcGVjaWZpY19jb3VudHMgPC0gbnVtZXJpYygwKQogIGZvciAoaSBpbiAxOiBsZW5ndGgoc3BlY2llc19pbikpewogIHBoeWx1bV9zcGVjaWZpY19jb3VudHNbaV0gPC0gKHN1bShvcnRob19jb3VudHNbc3BlY2lmaWNfb3J0aG9fYXRfbGVhc3Rfb25lLCBzcGVjaWVzX2luW2ldXSkpCiAgfQogIHBoeWx1bV9kZiA8LSBkYXRhLmZyYW1lKHNwZWNpZXMgPSBzcGVjaWVzX2luLCBwaHlsdW1fc3BlY2lmaWNfZ2VuZXMgPSBwaHlsdW1fc3BlY2lmaWNfY291bnRzKQogIHBoeWx1bV9zcGVjaWZpY19nZW5lcyA8LSByYmluZChwaHlsdW1fc3BlY2lmaWNfZ2VuZXMsIHBoeWx1bV9kZikKfQpwaHlsdW1fc3BlY2lmaWNfZ2VuZXMKYGBgCgoKKioqCiMjIyMgUmVhZCB1bmFzc2lnbmVkIGdlbmVzIGRhdGEKCmBgYHtyfQpzdGF0c19wZXJfc3BlY2llcyA8LSByZWFkX3RzdigiU3RhdGlzdGljc19QZXJTcGVjaWVzX0RlY18yMC5jc3YiKQpzdGF0c19wZXJfc3BlY2llcwpgYGAKCmBgYHtyfQpzcGVjaWVzIDwtIGNvbG5hbWVzKHN0YXRzX3Blcl9zcGVjaWVzKQpzcGVjaWVzIDwtIHNwZWNpZXNbMjo1MF0KCnRvdGFsX2dlbmVzIDwtIHN0YXRzX3Blcl9zcGVjaWVzICU+JSBzbGljZSgxKSAlPiUgdW5saXN0KC4sIHVzZS5uYW1lcz1GQUxTRSkKdG90YWxfZ2VuZXMgPC0gYXMubnVtZXJpYyh0b3RhbF9nZW5lc1syOjUwXSkKCnVuYXNzaWduZWRfZ2VuZXMgPC0gc3RhdHNfcGVyX3NwZWNpZXMgJT4lIHNsaWNlKDMpICU+JSB1bmxpc3QoLiwgdXNlLm5hbWVzPUZBTFNFKQp1bmFzc2lnbmVkX2dlbmVzIDwtIGFzLm51bWVyaWModW5hc3NpZ25lZF9nZW5lc1syOjUwXSkKdW5hc3NpZ25lZF9nZW5lcwoKcGVyY2VudF91bmFzc2lnbmVkIDwtIHN0YXRzX3Blcl9zcGVjaWVzICU+JSBzbGljZSg1KSAlPiUgdW5saXN0KC4sIHVzZS5uYW1lcz1GQUxTRSkKcGVyY2VudF91bmFzc2lnbmVkIDwtIGFzLm51bWVyaWMocGVyY2VudF91bmFzc2lnbmVkWzI6NTBdKQoKdW5hc3NpZ25lZCA8LSB0aWJibGUoc3BlY2llcywgdG90YWxfZ2VuZXMsIHVuYXNzaWduZWRfZ2VuZXMsIHBlcmNlbnRfdW5hc3NpZ25lZCkKdW5hc3NpZ25lZApgYGAKCgojIyMjIFJlb3JkZXIgbGV2ZWxzIG9mIHNwZWNpZXMgZmFjdG9yIGFjY29yZGluZyB0byBvcmlnaW5hbCB0YWJsZSAoZm9yIHBsb3R0aW5nKQoKYGBge3J9CnBoeWx1bV9zcGVjaWZpY19nZW5lcyRzcGVjaWVzIDwtIGZhY3RvcihwaHlsdW1fc3BlY2lmaWNfZ2VuZXMkc3BlY2llcywgbGV2ZWxzID0gcmV2KHRheG9ub215X3BoeWxhJHNwZWNpZXMpKQp1bmFzc2lnbmVkJHNwZWNpZXMgPC0gZmFjdG9yKHVuYXNzaWduZWQkc3BlY2llcywgbGV2ZWxzID0gcmV2KHRheG9ub215X3BoeWxhJHNwZWNpZXMpKQpgYGAKKioqCiMjIyMgQ29tYmluZSBwaHlsdW0tc3BlY2lmaWMgYW5kIHVuYXNzaWduZWQKCmBgYHtyfQpwaHlsdW1fc3BlY19hbmRfdW5hc3NpZ25lZCA8LSBsZWZ0X2pvaW4ocGh5bHVtX3NwZWNpZmljX2dlbmVzLCB1bmFzc2lnbmVkLCBieSA9ICJzcGVjaWVzIikKcGh5bHVtX3NwZWNfYW5kX3VuYXNzaWduZWQKYGBgCioqKgojIyMjIGNhbGN1bGF0ZSAib3RoZXIgZ2VuZXMiIGNvdW50cyAobmVpdGhlciBwaHlsdW0tc3BlY2lmaWMgb3IgdW5hc3NpZ25lZCkKCmBgYHtyfQpkZl9uZXcgPC0gcGh5bHVtX3NwZWNfYW5kX3VuYXNzaWduZWQgJT4lCiAgbXV0YXRlKG90aGVyX2dlbmVzID0gdG90YWxfZ2VuZXMgLSBwaHlsdW1fc3BlY2lmaWNfZ2VuZXMgLSB1bmFzc2lnbmVkX2dlbmVzKSAlPiUgCiAgc2VsZWN0KHNwZWNpZXMsIHBoeWx1bV9zcGVjaWZpY19nZW5lcywgdW5hc3NpZ25lZF9nZW5lcywgb3RoZXJfZ2VuZXMpCmRmX25ldwpgYGAKCioqKiAKIyMjIyBHYXRoZXIgdmFyaWFibGVzIGZvciBwbG90dGluZwoKYGBge3J9CmRmX2xvbmcgPC0gZGZfbmV3ICU+JSAKICBnYXRoZXIoYHBoeWx1bV9zcGVjaWZpY19nZW5lc2AsIGB1bmFzc2lnbmVkX2dlbmVzYCwgYG90aGVyX2dlbmVzYCwKICAgICAgICAga2V5ID0gImdlbmVfY2F0ZWdvcnkiLAogICAgICAgICB2YWx1ZSA9ICJnZW5lX2NvdW50IikgJT4lCiAgbXV0YXRlKGdlbmVfY2F0ZWdvcnkgPSBmYWN0b3IoZ2VuZV9jYXRlZ29yeSwgbGV2ZWxzID0gYygib3RoZXJfZ2VuZXMiLCJwaHlsdW1fc3BlY2lmaWNfZ2VuZXMiLCAidW5hc3NpZ25lZF9nZW5lcyIgKSkpCmRmX2xvbmcKYGBgCioqKgoKIyMjIyBQbG90CgpgYGB7cn0KZ2VuZV9jb3VudF9wbG90IDwtIGdncGxvdChkYXRhID0gZGZfbG9uZywgYWVzKHggPSBzcGVjaWVzLCB5ID0gZ2VuZV9jb3VudCwgZmlsbCA9IGdlbmVfY2F0ZWdvcnkpKSArCiAgZ2VvbV9iYXIoc3RhdD0iaWRlbnRpdHkiKSArCiAgY29vcmRfZmxpcCgpCmBgYAoKCioqKgojIyMjIE1ha2UgaHVtYW4tcmVhZGFibGUgc3BlY2llcyBuYW1lcywgY2xlYW4gdXAgbGFiZWxzLCBjaGFuZ2UgY29sb3JzLCBhbmQgY29sb3Igc3BlY2llcyBuYW1lcyBieSBwaHlsdW0uIAoKYGBge3J9CnNwZWNpZXNfbmFtZXMgPC0gZ3N1YigiXyIsICIgIiwgdGF4b25vbXlfcGh5bGEkc3BlY2llcykKc3BlY2llc19uYW1lcyA8LSBwYXN0ZSh0b3VwcGVyKHN1YnN0cihzcGVjaWVzX25hbWVzLCAxLCAxKSksIHN1YnN0cihzcGVjaWVzX25hbWVzLCAyLCBuY2hhcihzcGVjaWVzX25hbWVzKSksIHNlcD0iIikKc3BlY2llc19uYW1lcwpgYGAKCgpgYGB7cn0KZ2VuZV9jb3VudF9wbG90ICsKICBzY2FsZV9maWxsX3ZpcmlkaXMob3B0aW9uPSJ2aXJpZGlzIixkaXNjcmV0ZSA9IFRSVUUsIGxhYmVscyA9IGMoIk90aGVyIiwgIlBoeWx1bS1zcGVjaWZpYyIsICJVbmFzc2lnbmVkIikpICsgIyBDb2xvciArIGxlZ2VuZCBuYW1lcyBzcGVsbGVkIG91dAogIGd1aWRlcyhmaWxsID0gZ3VpZGVfbGVnZW5kKHJldmVyc2UgPSBUUlVFKSkgKyAjIFJldmVyc2UgbGVnZW5kIGxhYmVscyBzbyB0aGF0IG90aGVyIGlzIG5vdCBmaXJzdAogIGxhYnMoeSA9ICJHZW5lIGNvdW50IikgKyAjIHkgYXhpcyBsYWJlbAogIHNjYWxlX3hfZGlzY3JldGUobmFtZSA9ICJTcGVjaWVzIiwgbGFiZWxzID0gcmV2KHNwZWNpZXNfbmFtZXMpKSArICAjIGNsZWFuIHVwIHgtYXhpcyBsYWJlbAogIHRoZW1lKGF4aXMudGV4dC55ICA9IGVsZW1lbnRfdGV4dChzaXplPTcsIGNvbG9yID0gcmV2KHRheG9ub215X3BoeWxhJHBoeWx1bSkpKSAjIGNvbG9yIHNwZWNpZXMgbmFtZXMgYnkgcGh5bHVtICh1Z2x5IGJ1dCB1c2VmdWwpCmBgYAoKKioqCgojIyMgUGxvdCB0aGUgc2FtZSBkYXRhIGFzIHBlcmNlbnRhZ2UKCmBgYHtyfQpkZl9wZXJjZW50IDwtIHBoeWx1bV9zcGVjX2FuZF91bmFzc2lnbmVkICU+JSAKICBtdXRhdGUocGVyY2VudF9waHlsdW1fc3BlY2lmaWMgPSAocGh5bHVtX3NwZWNpZmljX2dlbmVzL3RvdGFsX2dlbmVzKSoxMDAsIAogICAgICAgICBwZXJjZW50X290aGVyX2dlbmVzID0gKHRvdGFsX2dlbmVzIC0gcGh5bHVtX3NwZWNpZmljX2dlbmVzIC0gdW5hc3NpZ25lZF9nZW5lcykqMTAwL3RvdGFsX2dlbmVzKSAlPiUgCiAgc2VsZWN0KHNwZWNpZXMsIHBlcmNlbnRfcGh5bHVtX3NwZWNpZmljLCBwZXJjZW50X3VuYXNzaWduZWQsIHBlcmNlbnRfb3RoZXJfZ2VuZXMpCmRmX3BlcmNlbnQKYGBgCgpgYGB7cn0KZGZfcGVyY2VudF9sb25nIDwtIGRmX3BlcmNlbnQgJT4lIAogIGdhdGhlcihgcGVyY2VudF9waHlsdW1fc3BlY2lmaWNgLCBgcGVyY2VudF91bmFzc2lnbmVkYCwgYHBlcmNlbnRfb3RoZXJfZ2VuZXNgLAogICAgICAgICBrZXkgPSAiZ2VuZV9jYXRlZ29yeSIsCiAgICAgICAgIHZhbHVlID0gInBlcmNlbnRfZ2VuZXMiKSAlPiUKICBtdXRhdGUoZ2VuZV9jYXRlZ29yeSA9IGZhY3RvcihnZW5lX2NhdGVnb3J5LCBsZXZlbHMgPSBjKCJwZXJjZW50X290aGVyX2dlbmVzIiwicGVyY2VudF9waHlsdW1fc3BlY2lmaWMiLCAicGVyY2VudF91bmFzc2lnbmVkIiApKSkKZGZfcGVyY2VudF9sb25nCmBgYAoKYGBge3J9CnBlcmNlbnRfcGxvdCA8LSBnZ3Bsb3QoZGF0YSA9IGRmX3BlcmNlbnRfbG9uZywgYWVzKHggPSBzcGVjaWVzLCB5ID0gcGVyY2VudF9nZW5lcywgZmlsbCA9IGdlbmVfY2F0ZWdvcnkpKSArCiAgZ2VvbV9iYXIoc3RhdD0iaWRlbnRpdHkiKSArCiAgY29vcmRfZmxpcCgpCmBgYAoKYGBge3J9CnBlcmNlbnRfcGxvdCArCiAgc2NhbGVfZmlsbF92aXJpZGlzKG9wdGlvbj0idmlyaWRpcyIsZGlzY3JldGUgPSBUUlVFLCBsYWJlbHMgPSBjKCJPdGhlciIsICJQaHlsdW0tc3BlY2lmaWMiLCAiVW5hc3NpZ25lZCIpKSArCiAgbGFicyh5ID0gIiUiKSArCiAgZ3VpZGVzKGZpbGwgPSBndWlkZV9sZWdlbmQocmV2ZXJzZSA9IFRSVUUpKSArCiAgc2NhbGVfeF9kaXNjcmV0ZShuYW1lID0gIlNwZWNpZXMiLCBsYWJlbHMgPSByZXYoc3BlY2llc19uYW1lcykpICsKICB0aGVtZShheGlzLnRleHQueSAgPSBlbGVtZW50X3RleHQoc2l6ZT03LCBjb2xvciA9IHJldih0YXhvbm9teV9waHlsYSRwaHlsdW0pKSkKYGBgCgoKCgoKCgo=
