# Supplementary material for: The genome of the colonial hydroid Hydractinia reveals that their stem cells use a toolkit of evolutionarily shared genes with all animals
Source: Genome Res. 2024 Mar;34(3):498–513. doi: 10.1101/gr.278382.123 (PMC11067881; doi:10.1101/gr.278382.123)
Supplement: Supplement 10 [file Supplemental_Data.zip › Supplemental_Data/Supplemental_Data_S15.html]

6006bf2b8a8d40529c6a0c8bf82fa58e


<!DOCTYPE html>

Orthogroup annotation


Code 

- Show All Code
- Hide All Code
- Download Rmd

# Orthogroup annotation


```
library("tidyverse")
```


---

### Read orthogroups


```
orthogroups <- read_tsv("Orthogroups_Dec_20.csv")
```


```
Missing column names filled in: 'X1' [1]Parsed with column specification:
cols(
  .default = col_character()
)
See spec(...) for full column specifications.
```


```
colnames(orthogroups)[1] <- "orthogroup_id"
orthogroups
```


---

### Read PANNZER annotations


```
descriptions_original <- read_tsv("~/research/orthogroup_parsing/pannzer_annotations/combined_descriptions.tsv", col_names = TRUE, skip_empty_rows = TRUE)
```


```
descriptions <- descriptions_original %>% select(qpid, desc, genename)
colnames(descriptions) <- c("gene_id", "pannzer_description", "pannzer_gene_name")
descriptions
```


```
go_terms <- read_tsv("~/research/orthogroup_parsing/pannzer_annotations/combined_GO_terms.tsv", col_names = TRUE, skip_empty_rows = TRUE)
```


```
go_MF <- go_terms[go_terms$ARGOT_rank == 1,] %>% filter(ontology == "MF") %>% select(qpid, desc)
colnames(go_MF) <-  c("gene_id", "molecular_function")
go_BP <-go_terms[go_terms$ARGOT_rank == 1,] %>% filter(ontology == "BP") %>% select(qpid, desc)
colnames(go_BP) <-  c("gene_id", "biological_processes")
go_CC <- go_terms[go_terms$ARGOT_rank == 1,] %>% filter(ontology == "CC") %>% select(qpid, desc)
colnames(go_CC) <-  c("gene_id", "cellular_component")
annotations <- left_join(descriptions, go_MF, by = "gene_id") %>% left_join(go_BP, by = "gene_id") %>% left_join(go_CC, by = "gene_id")
annotations
```


```
remove(go_terms)
```


---

### Read diamond annotations


```
diamond_annotations <-read_tsv("all_best_hits.diamond.blastp", col_names = c("gene_id", "best_BLAST_hit", "eval"))
```


```
diamond_annotations
```


---

### Read unassigned orthogroups


```
unassigned_orthogroups <- read_tsv("Orthogroups_UnassignedGenes_Dec_20.csv", col_types = cols(.default = "c"))
```


```
Missing column names filled in: 'X1' [1]
```


```
colnames(unassigned_orthogroups)[1] <- "orthogroup_id"
unassigned_orthogroups
```


---

### Annotate all unassigned genes (all species)


```
species <- "acanthaster_planci"
unassigned_orthogroups[,species]
```


```
acanthaster_unassigned <- unassigned_orthogroups[!is.na(unassigned_orthogroups$acanthaster_planci), "acanthaster_planci"]
colnames(acanthaster_unassigned) <- "gene_id"
acanthaster_unassigned
```


```
acanthaster_unassigned <- left_join(acanthaster_unassigned, annotations, by = "gene_id") %>% left_join(diamond_annotations, by = "gene_id")
```


---

#### Calculate: Number of genes with no BLAST hit, genes with uncharacterized blast hit, and others.


```
nrow(acanthaster_unassigned)
```


```
[1] 5278
```


```
sum(!is.na(acanthaster_unassigned$best_BLAST_hit))
```


```
[1] 1944
```


```
acanthaster_unassigned[grep("uncharacterized", tolower(acanthaster_unassigned$best_BLAST_hit)), ]
```


```
count_unassigned <- function(unassigned){
  species_names <- colnames(unassigned[,-1])
  print(species_names)
  output <- data.frame()
  for(species_name in species_names){
    #print(species_name)
    species_unassigned <- unassigned[!is.na(unassigned[,species_name]), species_name]
    colnames(species_unassigned) <- "gene_id"
    species_unassigned <- left_join(species_unassigned, annotations, by = "gene_id") %>% left_join(diamond_annotations, by = "gene_id")
    unassigned_num <- nrow(species_unassigned)
    no_blast <- sum(is.na(species_unassigned$best_BLAST_hit))
    percent_no_blast <- round(100*no_blast/unassigned_num, digits = 1)
    blast_hit_uncharacterized <- nrow(species_unassigned[grep("uncharacterized", tolower(species_unassigned$best_BLAST_hit)), ])
    blast_hit_hypothetical <- nrow(species_unassigned[grep("hypothetical", tolower(species_unassigned$best_BLAST_hit)), ])
    uncharacterized = sum(blast_hit_uncharacterized,blast_hit_hypothetical )
    percent_uncharacterized <- round(100*uncharacterized/unassigned_num, digits = 1)
    characterized <- unassigned_num - (no_blast + uncharacterized)
    percent_characterized <- round(100*characterized/unassigned_num, digits = 1)
    df <- data.frame(species = species_name, unassigned_genes = unassigned_num, no_blast_hit = no_blast, uncharacterized_blast_hit = uncharacterized, characterized_blast_hit = characterized,
                     percent_no_blast_hit = percent_no_blast, percent_uncharacterized = percent_uncharacterized, percent_characterized = percent_characterized)
    output <- rbind(output, df)
  }
  output
}
```


```
unassigned_stats <- count_unassigned(unassigned_orthogroups)
```


```
 [1] "acanthaster_planci"            "acropora_digitifera"           "amphimedon_queenslandica"     
 [4] "aurelia_aurita_atlantic"       "aurelia_aurita_pacific"        "branchiostoma_floridae"       
 [7] "caenorhabditis_elegans"        "capitella_teleta"              "capsaspora_owczarzaki"        
[10] "ciona_intestinalis"            "clytia_hemisphaerica"          "creolimax_fragrantissima"     
[13] "danio_rerio"                   "daphnia_pulex"                 "dendronephthya_gigantea"      
[16] "drosophila_melanogaster"       "exaiptasia_pallida"            "gallus_gallus"                
[19] "helobdella_robusta"            "hofstenia_miamia"              "hoilungia_hongkongensis"      
[22] "homo_sapiens"                  "hydra_magnipapillata"          "hydractinia_echinata"         
[25] "hydractinia_symbiolongicarpus" "ixodes_scapularis"             "kudoa_iwatai"                 
[28] "lottia_gigantea"               "mnemiopsis_leidyi"             "monosiga_brevicolis"          
[31] "morbakka_virulenta"            "nematostella_vectensis"        "nemopilema_nomurai"           
[34] "notospermus_geniculatus"       "octopus_bimaculoides"          "orbicella_faveolata"          
[37] "phoronis_australis"            "pocillopora_damicornis"        "pristionchus_pacificus"       
[40] "renilla_muelleri"              "saccharomyces_cerevisiae"      "saccoglossus_kowalevskii"     
[43] "salpingoeca_rosetta"           "schistosoma_mansoni"           "schmidtea_mediterranea"       
[46] "sphaeroforma_arctica"          "tribolium_castaneum"           "trichoplax_adhaerens"         
[49] "xenopus_tropicalis"
```


```
unassigned_stats
```


```
#write_tsv(unassigned_stats, path = "unassigned_stats.tsv")
```

#### Who are the species with > 50 % characterized unassigned genes.

### Look at species with a lot of characterized blast hits


```
danio <- unassigned_orthogroups[!is.na(unassigned_orthogroups[,"danio_rerio"]), "danio_rerio"]
colnames(danio) <- "gene_id"
danio_annot <- left_join(danio, annotations, by = "gene_id") %>% left_join(diamond_annotations, by = "gene_id")
```


#### Output hydractinia unassigned genes


```
symbio_unassigned <- unassigned_orthogroups[!is.na(unassigned_orthogroups[,"hydractinia_symbiolongicarpus"]), "hydractinia_symbiolongicarpus"]
write_tsv(symbio_unassigned, "symbio_unassigned.txt")
symbio_unassigned
```


```
colnames(symbio_unassigned) <- "gene_id"
symbio_unassigned_annot <- left_join(symbio_unassigned, annotations, by = "gene_id") %>% left_join(diamond_annotations, by = "gene_id")
symbio_unassigned_annot
```


```
echinata_unassigned <- unassigned_orthogroups[!is.na(unassigned_orthogroups[,"hydractinia_echinata"]), "hydractinia_echinata"]
write_tsv(echinata_unassigned, "echinata_unassigned.txt")
echinata_unassigned
```

### Assess transcriptome evidence for unassigned genes


```
unassigned_overlap_hsym <- read_tsv("symbio_overl_transcript")
```


```
Parsed with column specification:
cols(
  `#gene` = [31mcol_character()[39m,
  gene_start = [32mcol_double()[39m,
  gene_end = [32mcol_double()[39m,
  overl_length = [32mcol_double()[39m,
  `overl_%` = [32mcol_double()[39m,
  transcript_start = [32mcol_double()[39m,
  transcript_end = [32mcol_double()[39m,
  transcript = [31mcol_character()[39m
)
```


```
unassigned_overlap_hsym
```


#### Only keep gene/transcript overlap with the largest overlap for each gene


```
column_names <- colnames(unassigned_overlap_hsym)
column_names[1] <- c("gene_id")
colnames(unassigned_overlap_hsym) <- column_names
unassigned_longest_overlap_hsym <- unassigned_overlap_hsym %>% group_by(gene_id) %>% slice(which.max(`overl_%`))
```


```
mean(unassigned_longest_overlap_hsym$`overl_%`)
```


```
[1] 80.14421
```


##### Percentage of unassigned genes that overlap at 90% with transcript


```
100*nrow(unassigned_longest_overlap_hsym[unassigned_longest_overlap_hsym$`overl_%` >= 90, ])/nrow(unassigned_longest_overlap_hsym)
```


```
[1] 71.91781
```

##### Percentage of unassigned genes that overlap at 50% with transcript


```
100*nrow(unassigned_longest_overlap_hsym[unassigned_longest_overlap_hsym$`overl_%` >= 50, ])/nrow(unassigned_longest_overlap_hsym)
```


```
[1] 78.76712
```

### Assess transcriptome evidence for all genes in genome (for comparison)


```
all_genes_overlap_hsym <- read_tsv("Hsym_overl_transcript")
```


```
Parsed with column specification:
cols(
  `#gene` = [31mcol_character()[39m,
  gene_start = [32mcol_double()[39m,
  gene_end = [32mcol_double()[39m,
  overl_length = [32mcol_double()[39m,
  `overl_%` = [32mcol_double()[39m,
  transcript_start = [32mcol_double()[39m,
  transcript_end = [32mcol_double()[39m,
  transcript = [31mcol_character()[39m
)
```


```
column_names <- colnames(all_genes_overlap_hsym)
column_names[1] <- c("gene_id")
column_names
```


```
[1] "gene_id"          "gene_start"       "gene_end"         "overl_length"     "overl_%"         
[6] "transcript_start" "transcript_end"   "transcript"
```


```
colnames(all_genes_overlap_hsym) <- column_names
all_genes_overlap_hsym
```


#### Only keep gene/transcript overlap with the largest overlap for each gene


```
mean(all_genes_overlap_hsym$`overl_%`)
```


```
[1] 56.33483
```


```
100*nrow(all_genes_overlap_hsym[all_genes_overlap_hsym$`overl_%` >= 90, ])/nrow(all_genes_overlap_hsym)
```


```
[1] 49.98591
```


---

#### Same with Hydractinia echinata


```
unassigned_overlap_hech <- read_tsv("echinata_overl_transcript")
```


```
Parsed with column specification:
cols(
  `#gene` = [31mcol_character()[39m,
  gene_start = [32mcol_double()[39m,
  gene_end = [32mcol_double()[39m,
  overl_length = [32mcol_double()[39m,
  `overl_%` = [32mcol_double()[39m,
  transcript_start = [32mcol_double()[39m,
  transcript_end = [32mcol_double()[39m,
  transcript = [31mcol_character()[39m
)
```


```
unassigned_overlap_hech
```


```
column_names <- colnames(unassigned_overlap_hech)
column_names[1] <- c("gene_id")
colnames(unassigned_overlap_hech) <- column_names
unassigned_overlap_hech <- unassigned_overlap_hech %>% group_by(gene_id) %>% slice(which.max(`overl_%`))
```


```
mean(unassigned_overlap_hech$`overl_%`)
```


```
[1] 80.90112
```


```
100*nrow(unassigned_overlap_hech[unassigned_overlap_hech$`overl_%` >= 90, ])/nrow(unassigned_overlap_hech)
```


```
[1] 73.02417
```


```
100*nrow(unassigned_overlap_hech[unassigned_overlap_hech$`overl_%` >= 50, ])/nrow(unassigned_overlap_hech)
```


```
[1] 79.62116
```


```
all_genes_overlap_hech <- read_tsv("Hech_overl_transcript")
```


```
Parsed with column specification:
cols(
  `#gene` = [31mcol_character()[39m,
  gene_start = [32mcol_double()[39m,
  gene_end = [32mcol_double()[39m,
  overl_length = [32mcol_double()[39m,
  `overl_%` = [32mcol_double()[39m,
  transcript_start = [32mcol_double()[39m,
  transcript_end = [32mcol_double()[39m,
  transcript = [31mcol_character()[39m
)
```


```
all_genes_overlap_hech
```


```
column_names <- colnames(all_genes_overlap_hech)
column_names[1] <- c("gene_id")
colnames(all_genes_overlap_hech) <- column_names
all_genes_overlap_hech <- all_genes_overlap_hech %>% group_by(gene_id) %>% slice(which.max(`overl_%`))
```


```
mean(all_genes_overlap_hech$`overl_%`)
```


```
[1] 85.43459
```


```
100*nrow(all_genes_overlap_hech[all_genes_overlap_hech$`overl_%` >= 90, ])/nrow(all_genes_overlap_hech)
```


```
[1] 78.8996
```

#### Identify all genes with transcript evidence (for downstream analyses)

#### All genes with > 90% overlap:


```
hsym_90_overlap <- all_genes_longest_overlap_hsym[all_genes_longest_overlap_hsym$`overl_%` >= 90, ]
hsym_90_overlap <- hsym_90_overlap %>% select(gene_id) %>% mutate(overlap_90 = "yes")
hsym_90_overlap$gene_id <- paste("Hsym|", hsym_90_overlap$gene_id, sep = "" )
hsym_90_overlap
```

#### All genes with 50% overlap


```
hsym_50_overlap <- all_genes_longest_overlap_hsym[all_genes_longest_overlap_hsym$`overl_%` >= 50, ]
hsym_50_overlap <- hsym_50_overlap %>% select(gene_id) %>% mutate(overlap_50 = "yes")
hsym_50_overlap$gene_id <- paste("Hsym|", hsym_50_overlap$gene_id, sep = "" )
hsym_50_overlap
```

LS0tCnRpdGxlOiAiT3J0aG9ncm91cCBhbm5vdGF0aW9uIgpvdXRwdXQ6IGh0bWxfbm90ZWJvb2sKLS0tCgpgYGB7cn0KbGlicmFyeSgidGlkeXZlcnNlIikKYGBgCgoqKioKCiMjIyBSZWFkIG9ydGhvZ3JvdXBzCgpgYGB7cn0Kb3J0aG9ncm91cHMgPC0gcmVhZF90c3YoIk9ydGhvZ3JvdXBzX0RlY18yMC5jc3YiKQpjb2xuYW1lcyhvcnRob2dyb3VwcylbMV0gPC0gIm9ydGhvZ3JvdXBfaWQiCm9ydGhvZ3JvdXBzCmBgYAoKKioqCgojIyMgUmVhZCBQQU5OWkVSIGFubm90YXRpb25zCgpgYGB7ciBlY2hvID0gVCwgcmVzdWx0cyA9ICdoaWRlJ30KZGVzY3JpcHRpb25zX29yaWdpbmFsIDwtIHJlYWRfdHN2KCJ+L3Jlc2VhcmNoL29ydGhvZ3JvdXBfcGFyc2luZy9wYW5uemVyX2Fubm90YXRpb25zL2NvbWJpbmVkX2Rlc2NyaXB0aW9ucy50c3YiLCBjb2xfbmFtZXMgPSBUUlVFLCBza2lwX2VtcHR5X3Jvd3MgPSBUUlVFKQpkZXNjcmlwdGlvbnMgPC0gZGVzY3JpcHRpb25zX29yaWdpbmFsICU+JSBzZWxlY3QocXBpZCwgZGVzYywgZ2VuZW5hbWUpCmNvbG5hbWVzKGRlc2NyaXB0aW9ucykgPC0gYygiZ2VuZV9pZCIsICJwYW5uemVyX2Rlc2NyaXB0aW9uIiwgInBhbm56ZXJfZ2VuZV9uYW1lIikKZGVzY3JpcHRpb25zCgpnb190ZXJtcyA8LSByZWFkX3Rzdigifi9yZXNlYXJjaC9vcnRob2dyb3VwX3BhcnNpbmcvcGFubnplcl9hbm5vdGF0aW9ucy9jb21iaW5lZF9HT190ZXJtcy50c3YiLCBjb2xfbmFtZXMgPSBUUlVFLCBza2lwX2VtcHR5X3Jvd3MgPSBUUlVFKQpnb19NRiA8LSBnb190ZXJtc1tnb190ZXJtcyRBUkdPVF9yYW5rID09IDEsXSAlPiUgZmlsdGVyKG9udG9sb2d5ID09ICJNRiIpICU+JSBzZWxlY3QocXBpZCwgZGVzYykKY29sbmFtZXMoZ29fTUYpIDwtICBjKCJnZW5lX2lkIiwgIm1vbGVjdWxhcl9mdW5jdGlvbiIpCmdvX0JQIDwtZ29fdGVybXNbZ29fdGVybXMkQVJHT1RfcmFuayA9PSAxLF0gJT4lIGZpbHRlcihvbnRvbG9neSA9PSAiQlAiKSAlPiUgc2VsZWN0KHFwaWQsIGRlc2MpCmNvbG5hbWVzKGdvX0JQKSA8LSAgYygiZ2VuZV9pZCIsICJiaW9sb2dpY2FsX3Byb2Nlc3NlcyIpCmdvX0NDIDwtIGdvX3Rlcm1zW2dvX3Rlcm1zJEFSR09UX3JhbmsgPT0gMSxdICU+JSBmaWx0ZXIob250b2xvZ3kgPT0gIkNDIikgJT4lIHNlbGVjdChxcGlkLCBkZXNjKQpjb2xuYW1lcyhnb19DQykgPC0gIGMoImdlbmVfaWQiLCAiY2VsbHVsYXJfY29tcG9uZW50IikKCmFubm90YXRpb25zIDwtIGxlZnRfam9pbihkZXNjcmlwdGlvbnMsIGdvX01GLCBieSA9ICJnZW5lX2lkIikgJT4lIGxlZnRfam9pbihnb19CUCwgYnkgPSAiZ2VuZV9pZCIpICU+JSBsZWZ0X2pvaW4oZ29fQ0MsIGJ5ID0gImdlbmVfaWQiKQphbm5vdGF0aW9ucwpyZW1vdmUoZ29fdGVybXMpCmBgYAoKKioqCgojIyMgUmVhZCBkaWFtb25kIGFubm90YXRpb25zCgpgYGB7ciBlY2hvID0gVCwgcmVzdWx0cyA9ICdoaWRlJ30KZGlhbW9uZF9hbm5vdGF0aW9ucyA8LXJlYWRfdHN2KCJhbGxfYmVzdF9oaXRzLmRpYW1vbmQuYmxhc3RwIiwgY29sX25hbWVzID0gYygiZ2VuZV9pZCIsICJiZXN0X0JMQVNUX2hpdCIsICJldmFsIikpCmRpYW1vbmRfYW5ub3RhdGlvbnMKYGBgCgoqKioKCiMjIyBSZWFkIHVuYXNzaWduZWQgb3J0aG9ncm91cHMKCmBgYHtyfQp1bmFzc2lnbmVkX29ydGhvZ3JvdXBzIDwtIHJlYWRfdHN2KCJPcnRob2dyb3Vwc19VbmFzc2lnbmVkR2VuZXNfRGVjXzIwLmNzdiIsIGNvbF90eXBlcyA9IGNvbHMoLmRlZmF1bHQgPSAiYyIpKQpjb2xuYW1lcyh1bmFzc2lnbmVkX29ydGhvZ3JvdXBzKVsxXSA8LSAib3J0aG9ncm91cF9pZCIKdW5hc3NpZ25lZF9vcnRob2dyb3VwcwpgYGAKCioqKgoKIyMjIEFubm90YXRlIGFsbCB1bmFzc2lnbmVkIGdlbmVzIChhbGwgc3BlY2llcykKCmBgYHtyfQpzcGVjaWVzIDwtICJhY2FudGhhc3Rlcl9wbGFuY2kiCnVuYXNzaWduZWRfb3J0aG9ncm91cHNbLHNwZWNpZXNdCmFjYW50aGFzdGVyX3VuYXNzaWduZWQgPC0gdW5hc3NpZ25lZF9vcnRob2dyb3Vwc1shaXMubmEodW5hc3NpZ25lZF9vcnRob2dyb3VwcyRhY2FudGhhc3Rlcl9wbGFuY2kpLCAiYWNhbnRoYXN0ZXJfcGxhbmNpIl0KY29sbmFtZXMoYWNhbnRoYXN0ZXJfdW5hc3NpZ25lZCkgPC0gImdlbmVfaWQiCmFjYW50aGFzdGVyX3VuYXNzaWduZWQKYWNhbnRoYXN0ZXJfdW5hc3NpZ25lZCA8LSBsZWZ0X2pvaW4oYWNhbnRoYXN0ZXJfdW5hc3NpZ25lZCwgYW5ub3RhdGlvbnMsIGJ5ID0gImdlbmVfaWQiKSAlPiUgbGVmdF9qb2luKGRpYW1vbmRfYW5ub3RhdGlvbnMsIGJ5ID0gImdlbmVfaWQiKQpgYGAKCioqKgoKIyMjIyBDYWxjdWxhdGU6IE51bWJlciBvZiBnZW5lcyB3aXRoIG5vIEJMQVNUIGhpdCwgZ2VuZXMgd2l0aCB1bmNoYXJhY3Rlcml6ZWQgYmxhc3QgaGl0LCBhbmQgb3RoZXJzLgoKYGBge3J9Cm5yb3coYWNhbnRoYXN0ZXJfdW5hc3NpZ25lZCkKc3VtKCFpcy5uYShhY2FudGhhc3Rlcl91bmFzc2lnbmVkJGJlc3RfQkxBU1RfaGl0KSkKYWNhbnRoYXN0ZXJfdW5hc3NpZ25lZFtncmVwKCJ1bmNoYXJhY3Rlcml6ZWQiLCB0b2xvd2VyKGFjYW50aGFzdGVyX3VuYXNzaWduZWQkYmVzdF9CTEFTVF9oaXQpKSwgXQoKYGBgCgpgYGB7cn0KY291bnRfdW5hc3NpZ25lZCA8LSBmdW5jdGlvbih1bmFzc2lnbmVkKXsKICBzcGVjaWVzX25hbWVzIDwtIGNvbG5hbWVzKHVuYXNzaWduZWRbLC0xXSkKICBwcmludChzcGVjaWVzX25hbWVzKQogIG91dHB1dCA8LSBkYXRhLmZyYW1lKCkKICBmb3Ioc3BlY2llc19uYW1lIGluIHNwZWNpZXNfbmFtZXMpewogICAgI3ByaW50KHNwZWNpZXNfbmFtZSkKICAgIHNwZWNpZXNfdW5hc3NpZ25lZCA8LSB1bmFzc2lnbmVkWyFpcy5uYSh1bmFzc2lnbmVkWyxzcGVjaWVzX25hbWVdKSwgc3BlY2llc19uYW1lXQogICAgY29sbmFtZXMoc3BlY2llc191bmFzc2lnbmVkKSA8LSAiZ2VuZV9pZCIKICAgIHNwZWNpZXNfdW5hc3NpZ25lZCA8LSBsZWZ0X2pvaW4oc3BlY2llc191bmFzc2lnbmVkLCBhbm5vdGF0aW9ucywgYnkgPSAiZ2VuZV9pZCIpICU+JSBsZWZ0X2pvaW4oZGlhbW9uZF9hbm5vdGF0aW9ucywgYnkgPSAiZ2VuZV9pZCIpCiAgICB1bmFzc2lnbmVkX251bSA8LSBucm93KHNwZWNpZXNfdW5hc3NpZ25lZCkKICAgIG5vX2JsYXN0IDwtIHN1bShpcy5uYShzcGVjaWVzX3VuYXNzaWduZWQkYmVzdF9CTEFTVF9oaXQpKQogICAgcGVyY2VudF9ub19ibGFzdCA8LSByb3VuZCgxMDAqbm9fYmxhc3QvdW5hc3NpZ25lZF9udW0sIGRpZ2l0cyA9IDEpCiAgICBibGFzdF9oaXRfdW5jaGFyYWN0ZXJpemVkIDwtIG5yb3coc3BlY2llc191bmFzc2lnbmVkW2dyZXAoInVuY2hhcmFjdGVyaXplZCIsIHRvbG93ZXIoc3BlY2llc191bmFzc2lnbmVkJGJlc3RfQkxBU1RfaGl0KSksIF0pCiAgICBibGFzdF9oaXRfaHlwb3RoZXRpY2FsIDwtIG5yb3coc3BlY2llc191bmFzc2lnbmVkW2dyZXAoImh5cG90aGV0aWNhbCIsIHRvbG93ZXIoc3BlY2llc191bmFzc2lnbmVkJGJlc3RfQkxBU1RfaGl0KSksIF0pCiAgICB1bmNoYXJhY3Rlcml6ZWQgPSBzdW0oYmxhc3RfaGl0X3VuY2hhcmFjdGVyaXplZCxibGFzdF9oaXRfaHlwb3RoZXRpY2FsICkKICAgIHBlcmNlbnRfdW5jaGFyYWN0ZXJpemVkIDwtIHJvdW5kKDEwMCp1bmNoYXJhY3Rlcml6ZWQvdW5hc3NpZ25lZF9udW0sIGRpZ2l0cyA9IDEpCiAgICBjaGFyYWN0ZXJpemVkIDwtIHVuYXNzaWduZWRfbnVtIC0gKG5vX2JsYXN0ICsgdW5jaGFyYWN0ZXJpemVkKQogICAgcGVyY2VudF9jaGFyYWN0ZXJpemVkIDwtIHJvdW5kKDEwMCpjaGFyYWN0ZXJpemVkL3VuYXNzaWduZWRfbnVtLCBkaWdpdHMgPSAxKQogICAgZGYgPC0gZGF0YS5mcmFtZShzcGVjaWVzID0gc3BlY2llc19uYW1lLCB1bmFzc2lnbmVkX2dlbmVzID0gdW5hc3NpZ25lZF9udW0sIG5vX2JsYXN0X2hpdCA9IG5vX2JsYXN0LCB1bmNoYXJhY3Rlcml6ZWRfYmxhc3RfaGl0ID0gdW5jaGFyYWN0ZXJpemVkLCBjaGFyYWN0ZXJpemVkX2JsYXN0X2hpdCA9IGNoYXJhY3Rlcml6ZWQsCiAgICAgICAgICAgICAgICAgICAgIHBlcmNlbnRfbm9fYmxhc3RfaGl0ID0gcGVyY2VudF9ub19ibGFzdCwgcGVyY2VudF91bmNoYXJhY3Rlcml6ZWQgPSBwZXJjZW50X3VuY2hhcmFjdGVyaXplZCwgcGVyY2VudF9jaGFyYWN0ZXJpemVkID0gcGVyY2VudF9jaGFyYWN0ZXJpemVkKQogICAgb3V0cHV0IDwtIHJiaW5kKG91dHB1dCwgZGYpCiAgfQogIG91dHB1dAp9CmBgYAoKYGBge3J9CnVuYXNzaWduZWRfc3RhdHMgPC0gY291bnRfdW5hc3NpZ25lZCh1bmFzc2lnbmVkX29ydGhvZ3JvdXBzKQp1bmFzc2lnbmVkX3N0YXRzCndyaXRlX3Rzdih1bmFzc2lnbmVkX3N0YXRzLCBwYXRoID0gInVuYXNzaWduZWRfc3RhdHMudHN2IikKYGBgCgojIyMjIFdobyBhcmUgdGhlIHNwZWNpZXMgd2l0aCA+IDUwICUgY2hhcmFjdGVyaXplZCB1bmFzc2lnbmVkIGdlbmVzLgoKYGBge3J9CnVuYXNzaWduZWRfc3RhdHNbdW5hc3NpZ25lZF9zdGF0cyRwZXJjZW50X2NoYXJhY3Rlcml6ZWQgPj0gNTAsXQpgYGAKCgoKIyMjIExvb2sgYXQgc3BlY2llcyB3aXRoIGEgbG90IG9mIGNoYXJhY3Rlcml6ZWQgYmxhc3QgaGl0cwoKYGBge3J9CmRhbmlvIDwtIHVuYXNzaWduZWRfb3J0aG9ncm91cHNbIWlzLm5hKHVuYXNzaWduZWRfb3J0aG9ncm91cHNbLCJkYW5pb19yZXJpbyJdKSwgImRhbmlvX3JlcmlvIl0KY29sbmFtZXMoZGFuaW8pIDwtICJnZW5lX2lkIgpkYW5pb19hbm5vdCA8LSBsZWZ0X2pvaW4oZGFuaW8sIGFubm90YXRpb25zLCBieSA9ICJnZW5lX2lkIikgJT4lIGxlZnRfam9pbihkaWFtb25kX2Fubm90YXRpb25zLCBieSA9ICJnZW5lX2lkIikKCmBgYAoKYGBge3J9CmRhbmlvX2Fubm90CmBgYAoKIyMjIyBPdXRwdXQgaHlkcmFjdGluaWEgdW5hc3NpZ25lZCBnZW5lcwoKYGBge3J9CnN5bWJpb191bmFzc2lnbmVkIDwtIHVuYXNzaWduZWRfb3J0aG9ncm91cHNbIWlzLm5hKHVuYXNzaWduZWRfb3J0aG9ncm91cHNbLCJoeWRyYWN0aW5pYV9zeW1iaW9sb25naWNhcnB1cyJdKSwgImh5ZHJhY3RpbmlhX3N5bWJpb2xvbmdpY2FycHVzIl0Kd3JpdGVfdHN2KHN5bWJpb191bmFzc2lnbmVkLCAic3ltYmlvX3VuYXNzaWduZWQudHh0IikKc3ltYmlvX3VuYXNzaWduZWQKYGBgCgoKYGBge3J9CmNvbG5hbWVzKHN5bWJpb191bmFzc2lnbmVkKSA8LSAiZ2VuZV9pZCIKc3ltYmlvX3VuYXNzaWduZWRfYW5ub3QgPC0gbGVmdF9qb2luKHN5bWJpb191bmFzc2lnbmVkLCBhbm5vdGF0aW9ucywgYnkgPSAiZ2VuZV9pZCIpICU+JSBsZWZ0X2pvaW4oZGlhbW9uZF9hbm5vdGF0aW9ucywgYnkgPSAiZ2VuZV9pZCIpCnN5bWJpb191bmFzc2lnbmVkX2Fubm90CmBgYAoKCmBgYHtyfQplY2hpbmF0YV91bmFzc2lnbmVkIDwtIHVuYXNzaWduZWRfb3J0aG9ncm91cHNbIWlzLm5hKHVuYXNzaWduZWRfb3J0aG9ncm91cHNbLCJoeWRyYWN0aW5pYV9lY2hpbmF0YSJdKSwgImh5ZHJhY3RpbmlhX2VjaGluYXRhIl0Kd3JpdGVfdHN2KGVjaGluYXRhX3VuYXNzaWduZWQsICJlY2hpbmF0YV91bmFzc2lnbmVkLnR4dCIpCmVjaGluYXRhX3VuYXNzaWduZWQKYGBgCgojIyMgQXNzZXNzIHRyYW5zY3JpcHRvbWUgZXZpZGVuY2UgZm9yIHVuYXNzaWduZWQgZ2VuZXMKCmBgYHtyfQp1bmFzc2lnbmVkX292ZXJsYXBfaHN5bSA8LSByZWFkX3Rzdigic3ltYmlvX292ZXJsX3RyYW5zY3JpcHQiKQp1bmFzc2lnbmVkX292ZXJsYXBfaHN5bQpgYGAKCiMjIyMgT25seSBrZWVwIGdlbmUvdHJhbnNjcmlwdCBvdmVybGFwIHdpdGggdGhlIGxhcmdlc3Qgb3ZlcmxhcCBmb3IgZWFjaCBnZW5lCgpgYGB7cn0KY29sdW1uX25hbWVzIDwtIGNvbG5hbWVzKHVuYXNzaWduZWRfb3ZlcmxhcF9oc3ltKQpjb2x1bW5fbmFtZXNbMV0gPC0gYygiZ2VuZV9pZCIpCmNvbG5hbWVzKHVuYXNzaWduZWRfb3ZlcmxhcF9oc3ltKSA8LSBjb2x1bW5fbmFtZXMKdW5hc3NpZ25lZF9sb25nZXN0X292ZXJsYXBfaHN5bSA8LSB1bmFzc2lnbmVkX292ZXJsYXBfaHN5bSAlPiUgZ3JvdXBfYnkoZ2VuZV9pZCkgJT4lIHNsaWNlKHdoaWNoLm1heChgb3ZlcmxfJWApKQpgYGAKCmBgYHtyfQp1bmFzc2lnbmVkX2xvbmdlc3Rfb3ZlcmxhcF9oc3ltCmBgYAoKCmBgYHtyfQptZWFuKHVuYXNzaWduZWRfbG9uZ2VzdF9vdmVybGFwX2hzeW0kYG92ZXJsXyVgKQpgYGAKCiMjIyMjIFBlcmNlbnRhZ2Ugb2YgdW5hc3NpZ25lZCBnZW5lcyB0aGF0IG92ZXJsYXAgYXQgOTAlIHdpdGggdHJhbnNjcmlwdAoKYGBge3J9CjEwMCpucm93KHVuYXNzaWduZWRfbG9uZ2VzdF9vdmVybGFwX2hzeW1bdW5hc3NpZ25lZF9sb25nZXN0X292ZXJsYXBfaHN5bSRgb3ZlcmxfJWAgPj0gOTAsIF0pL25yb3codW5hc3NpZ25lZF9sb25nZXN0X292ZXJsYXBfaHN5bSkKYGBgCgojIyMjIyBQZXJjZW50YWdlIG9mIHVuYXNzaWduZWQgZ2VuZXMgdGhhdCBvdmVybGFwIGF0IDUwJSB3aXRoIHRyYW5zY3JpcHQKCmBgYHtyfQoxMDAqbnJvdyh1bmFzc2lnbmVkX2xvbmdlc3Rfb3ZlcmxhcF9oc3ltW3VuYXNzaWduZWRfbG9uZ2VzdF9vdmVybGFwX2hzeW0kYG92ZXJsXyVgID49IDUwLCBdKS9ucm93KHVuYXNzaWduZWRfbG9uZ2VzdF9vdmVybGFwX2hzeW0pCmBgYAoKIyMjIEFzc2VzcyB0cmFuc2NyaXB0b21lIGV2aWRlbmNlIGZvciBhbGwgZ2VuZXMgaW4gZ2Vub21lIChmb3IgY29tcGFyaXNvbikKCgpgYGB7cn0KYWxsX2dlbmVzX292ZXJsYXBfaHN5bSA8LSByZWFkX3RzdigiSHN5bV9vdmVybF90cmFuc2NyaXB0IikKY29sdW1uX25hbWVzIDwtIGNvbG5hbWVzKGFsbF9nZW5lc19vdmVybGFwX2hzeW0pCmNvbHVtbl9uYW1lc1sxXSA8LSBjKCJnZW5lX2lkIikKY29sdW1uX25hbWVzCmNvbG5hbWVzKGFsbF9nZW5lc19vdmVybGFwX2hzeW0pIDwtIGNvbHVtbl9uYW1lcwphbGxfZ2VuZXNfb3ZlcmxhcF9oc3ltCmBgYAoKCiMjIyMgT25seSBrZWVwIGdlbmUvdHJhbnNjcmlwdCBvdmVybGFwIHdpdGggdGhlIGxhcmdlc3Qgb3ZlcmxhcCBmb3IgZWFjaCBnZW5lCgpgYGB7cn0KYWxsX2dlbmVzX2xvbmdlc3Rfb3ZlcmxhcF9oc3ltIDwtIGFsbF9nZW5lc19vdmVybGFwX2hzeW0gJT4lIGdyb3VwX2J5KGdlbmVfaWQpICU+JSBzbGljZSh3aGljaC5tYXgoYG92ZXJsXyVgKSkKYWxsX2dlbmVzX2xvbmdlc3Rfb3ZlcmxhcF9oc3ltICU+JSBhcnJhbmdlKGBvdmVybF8lYCkKYGBgCgoKYGBge3J9Cm1lYW4oYWxsX2dlbmVzX292ZXJsYXBfaHN5bSRgb3ZlcmxfJWApCmBgYAoKYGBge3J9CjEwMCpucm93KGFsbF9nZW5lc19vdmVybGFwX2hzeW1bYWxsX2dlbmVzX292ZXJsYXBfaHN5bSRgb3ZlcmxfJWAgPj0gOTAsIF0pL25yb3coYWxsX2dlbmVzX292ZXJsYXBfaHN5bSkKYGBgCgoKKioqCgojIyMjIFNhbWUgd2l0aCBIeWRyYWN0aW5pYSBlY2hpbmF0YQoKCmBgYHtyfQp1bmFzc2lnbmVkX292ZXJsYXBfaGVjaCA8LSByZWFkX3RzdigiZWNoaW5hdGFfb3ZlcmxfdHJhbnNjcmlwdCIpCnVuYXNzaWduZWRfb3ZlcmxhcF9oZWNoCmBgYAoKCgoKYGBge3J9CmNvbHVtbl9uYW1lcyA8LSBjb2xuYW1lcyh1bmFzc2lnbmVkX292ZXJsYXBfaGVjaCkKY29sdW1uX25hbWVzWzFdIDwtIGMoImdlbmVfaWQiKQpjb2xuYW1lcyh1bmFzc2lnbmVkX292ZXJsYXBfaGVjaCkgPC0gY29sdW1uX25hbWVzCnVuYXNzaWduZWRfb3ZlcmxhcF9oZWNoIDwtIHVuYXNzaWduZWRfb3ZlcmxhcF9oZWNoICU+JSBncm91cF9ieShnZW5lX2lkKSAlPiUgc2xpY2Uod2hpY2gubWF4KGBvdmVybF8lYCkpCmBgYAoKCmBgYHtyfQptZWFuKHVuYXNzaWduZWRfb3ZlcmxhcF9oZWNoJGBvdmVybF8lYCkKYGBgCgpgYGB7cn0KMTAwKm5yb3codW5hc3NpZ25lZF9vdmVybGFwX2hlY2hbdW5hc3NpZ25lZF9vdmVybGFwX2hlY2gkYG92ZXJsXyVgID49IDkwLCBdKS9ucm93KHVuYXNzaWduZWRfb3ZlcmxhcF9oZWNoKQpgYGAKCgpgYGB7cn0KMTAwKm5yb3codW5hc3NpZ25lZF9vdmVybGFwX2hlY2hbdW5hc3NpZ25lZF9vdmVybGFwX2hlY2gkYG92ZXJsXyVgID49IDUwLCBdKS9ucm93KHVuYXNzaWduZWRfb3ZlcmxhcF9oZWNoKQpgYGAKCgpgYGB7cn0KYWxsX2dlbmVzX292ZXJsYXBfaGVjaCA8LSByZWFkX3RzdigiSGVjaF9vdmVybF90cmFuc2NyaXB0IikKYWxsX2dlbmVzX292ZXJsYXBfaGVjaApgYGAKCgpgYGB7cn0KY29sdW1uX25hbWVzIDwtIGNvbG5hbWVzKGFsbF9nZW5lc19vdmVybGFwX2hlY2gpCmNvbHVtbl9uYW1lc1sxXSA8LSBjKCJnZW5lX2lkIikKY29sbmFtZXMoYWxsX2dlbmVzX292ZXJsYXBfaGVjaCkgPC0gY29sdW1uX25hbWVzCmFsbF9nZW5lc19vdmVybGFwX2hlY2ggPC0gYWxsX2dlbmVzX292ZXJsYXBfaGVjaCAlPiUgZ3JvdXBfYnkoZ2VuZV9pZCkgJT4lIHNsaWNlKHdoaWNoLm1heChgb3ZlcmxfJWApKQpgYGAKCgpgYGB7cn0KbWVhbihhbGxfZ2VuZXNfb3ZlcmxhcF9oZWNoJGBvdmVybF8lYCkKYGBgCgpgYGB7cn0KMTAwKm5yb3coYWxsX2dlbmVzX292ZXJsYXBfaGVjaFthbGxfZ2VuZXNfb3ZlcmxhcF9oZWNoJGBvdmVybF8lYCA+PSA5MCwgXSkvbnJvdyhhbGxfZ2VuZXNfb3ZlcmxhcF9oZWNoKQpgYGAKCgoKIyMjIyBJZGVudGlmeSBhbGwgZ2VuZXMgd2l0aCB0cmFuc2NyaXB0IGV2aWRlbmNlIChmb3IgZG93bnN0cmVhbSBhbmFseXNlcykKCiMjIyMgQWxsIGdlbmVzIHdpdGggPiA5MCUgb3ZlcmxhcDoKCmBgYHtyfQpoc3ltXzkwX292ZXJsYXAgPC0gYWxsX2dlbmVzX2xvbmdlc3Rfb3ZlcmxhcF9oc3ltW2FsbF9nZW5lc19sb25nZXN0X292ZXJsYXBfaHN5bSRgb3ZlcmxfJWAgPj0gOTAsIF0KaHN5bV85MF9vdmVybGFwIDwtIGhzeW1fOTBfb3ZlcmxhcCAlPiUgc2VsZWN0KGdlbmVfaWQpICU+JSBtdXRhdGUob3ZlcmxhcF85MCA9ICJ5ZXMiKQpoc3ltXzkwX292ZXJsYXAkZ2VuZV9pZCA8LSBwYXN0ZSgiSHN5bXwiLCBoc3ltXzkwX292ZXJsYXAkZ2VuZV9pZCwgc2VwID0gIiIgKQpoc3ltXzkwX292ZXJsYXAKYGBgCgojIyMjIEFsbCBnZW5lcyB3aXRoIDUwJSBvdmVybGFwCgpgYGB7cn0KaHN5bV81MF9vdmVybGFwIDwtIGFsbF9nZW5lc19sb25nZXN0X292ZXJsYXBfaHN5bVthbGxfZ2VuZXNfbG9uZ2VzdF9vdmVybGFwX2hzeW0kYG92ZXJsXyVgID49IDUwLCBdCmhzeW1fNTBfb3ZlcmxhcCA8LSBoc3ltXzUwX292ZXJsYXAgJT4lIHNlbGVjdChnZW5lX2lkKSAlPiUgbXV0YXRlKG92ZXJsYXBfNTAgPSAieWVzIikKaHN5bV81MF9vdmVybGFwJGdlbmVfaWQgPC0gcGFzdGUoIkhzeW18IiwgaHN5bV81MF9vdmVybGFwJGdlbmVfaWQsIHNlcCA9ICIiICkKaHN5bV81MF9vdmVybGFwCmBgYAoKCgoKCgoKCgoKCg==
