# Supplementary material for: The genome of the colonial hydroid Hydractinia reveals that their stem cells use a toolkit of evolutionarily shared genes with all animals
Source: Genome Res. 2024 Mar;34(3):498–513. doi: 10.1101/gr.278382.123 (PMC11067881; doi:10.1101/gr.278382.123)
Supplement: Supplement 10 [file Supplemental_Data.zip › Supplemental_Data/Supplemental_Data_S49.docx]

SA01 – Initial Clustering and Visualization

*March 20^th^, 2020, updated February 20^th^, 2023*

Summary

This section shows the first version of the *Hydractinia symbiolongicarpus* single-cell atlas, using the R package ‘Seurat’ (1). It includes how we created the Seurat data object for our raw 10X single-cell chromium data, visualizing the data using either tSNE and UMAP methods, and our first attempt at annotating clusters as cell types and cell states. The atlas shown in this section is not the final version of our cell atlas and does not include any of the filtering steps used to improve the data (shown in later sections of this document). In the final atlas, cluster labels were finalized after further literature review, as well as further bioinformatic and biological verification (e.g. *in situ hybridization*).

Input Files

- “3XPBS_filtered_feature_bc_matrix” *(raw 10X single-cell chromium data)*
- “Seawater_filtered_feature_bc_matrix” *(raw 10X single-cell chromium data)*

Output Files

- "aggr.RData" *(Our first Seurat data object)*

Program Versions

- Seurat version 4.3.0
- R version 4.2.1

Merging 10x Libraries and Creating the initial Seurat Object

*# Load the R package ‘Seurat’*

**library**(Seurat)

*# Set working directory to folder with 10x libraries “*1_Cell_rangerV7_libraries/”

setwd("~/Desktop/Updating_Seurat")

*# Create a Seurat object for each sample*

for (file in c("3XPBS_filtered_feature_bc_matrix", "Seawater_filtered_feature_bc_matrix")){

seurat_data <- Read10X(data.dir = paste0("1_Cell_rangerV7_libraries/", file))

seurat_obj <- CreateSeuratObject(counts = seurat_data,

min.features = 100,

project = file)

assign(file, seurat_obj)

}

*# Merge libraries into a single Seurat object “aggr”*

aggr <- merge(x=`3XPBS_filtered_feature_bc_matrix`,

y=Seawater_filtered_feature_bc_matrix,

add.cell.id = c("3XPBS", "Seawater"))

*# Look at number of genes and cells in initial Seurat data object*

aggr

## An object of class Seurat

## 22034 features across 10237 samples within 1 assay

## Active assay: RNA (22034 features, 0 variable features)

*## Standard pre-processing workflow*

*# Removing cells with high mitochondrial RNA*

*# HyS0613. is the Mitochondrial scaffold in the H. symbiolongicarpus genome*

aggr[["percent.mt"]] <- PercentageFeatureSet(aggr, pattern = "^HyS0613.")

*# Subset data to get cells with less than 5% mitochondrial counts*

aggr <- subset(aggr, subset = percent.mt < 5)

*# Normalizing the data*

aggr <- NormalizeData(aggr, normalization.method = "LogNormalize", scale.factor = 10000)

*# Set number of highly variable features to be used in downstream analyses*

aggr <- FindVariableFeatures(aggr, selection.method = "vst", nfeatures = 4000)

*# Scale the data*

all.genes <- rownames(aggr)

aggr <- ScaleData(aggr, features = all.genes)

*# Perform linear dimensional reduction using Principal Component Analysis*

aggr <- RunPCA(aggr, features = VariableFeatures(object = aggr))

*# Cluster the cells*

aggr <- FindNeighbors(aggr, dims = 1:16)

aggr <- FindClusters(aggr, resolution = 0.9, verbose = FALSE)

*# Run non-linear dimensional reduction (UMAP)*

aggr <- RunUMAP(aggr, dims = 1:16)

*## Visualize the clusters using UMAP (figure 1)*

DimPlot(aggr, reduction = "umap", label = TRUE)

It is important to note that we only show the mitochondrial pre-processing filtration method in our code, though we tried other common filters, like excluding cells that express below or above a certain threshold of genes. We found that none of these filters significantly affected our data, with only 1 cell being filtered out when we used a combination of filters taken from the Seurat tutorial (1) and *Hydra* single-cell analysis (2), which includes removing cells that express fewer than 200 genes, more than 7000 genes, and consist of more than 5% mitochondrial genes.


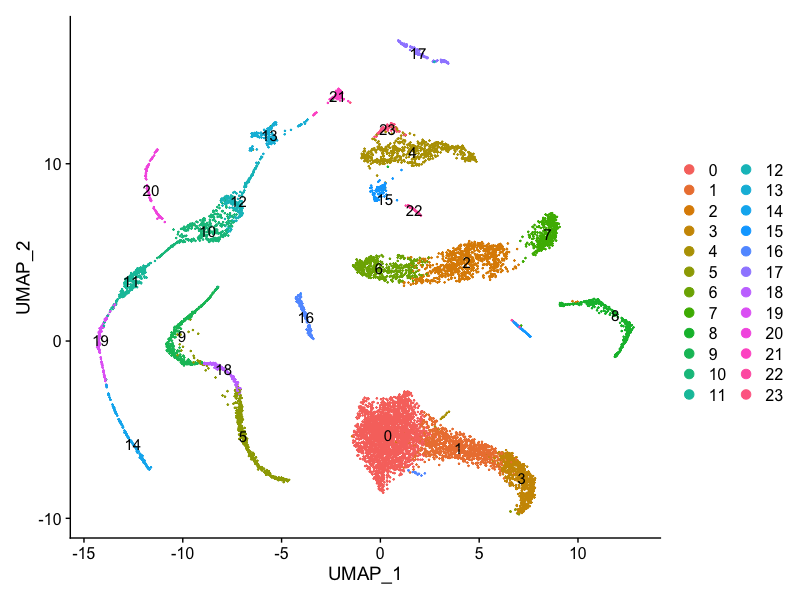


**Figure 1:** UMAP visualization of our 10X single-cell data. Cluster numbers (C0-C23) are shown in the legend on the right.

*## Annotate UMAP clusters with cell type and cell state labels*

*# Creating a copy of the Seurat data object for the new cluster labels*

aggr_ID <- aggr

*# Creating a vector with the new cell type and cell state labels*

*# New labels in the vector are ordered according to their cluster number*

*# E.g. The first label in our vector, “sprm_5,” corresponds to our first cluster, C0*

new.cluster.ids <- c("sprm_5", "sprm_4", "nem_2", "sprm_3", "enEp/ecEp", "sprm_2", "nem_1", "nem_3", "nem_4", "ISC/germ","ISC", "nb_1", "ISC/Neuronal", "Neurons", "nb_3", "ecEp_1", "ecEp_2", "Zgc", "sprm_1", "nb_2", "nb_4", "Mgc", "ecEp_3", "ecEp_4", "ecEp_1")

*# Change the cluster labels from numbers to cell type and cell state labels*

names(new.cluster.ids) <- levels(aggr_ID)

aggr_ID <- RenameIdents(aggr_ID, new.cluster.ids)

*# Visualize the clusters using tSNE and UMAP methods* *with the new labels*

DimPlot(aggr_ID, reduction = "umap", label = TRUE, label.size = 4) + NoLegend()


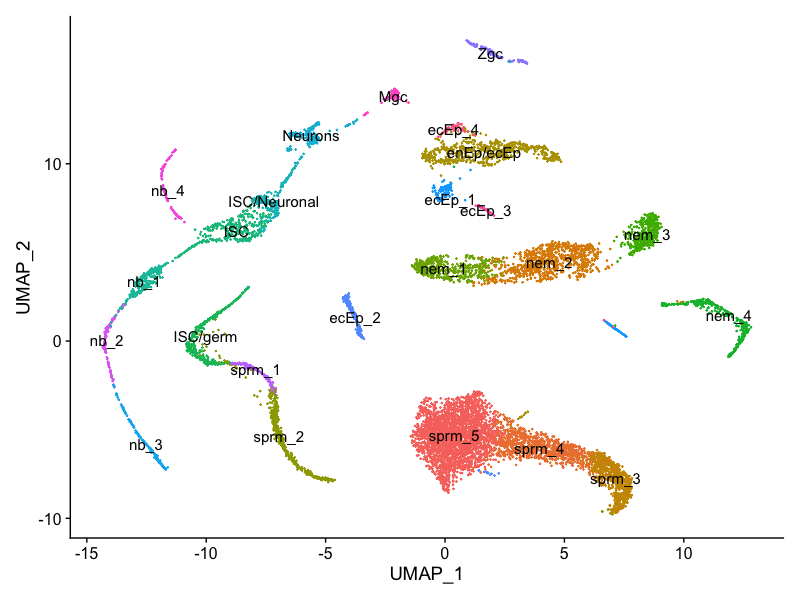


**Figure 2:** UMAP visualizations with cell type and cell state labels of the different clusters in our data. Abbreviations: ecEP: ectodermal epithelial cell, enEP: endodermal epithelial cell, germ: germ cell, ISC: interstitial stem cell, Mgc: mucous gland cell, nb: nematoblast, nem: nematocyte, sprm: sperm, Zgc: zymogen gland cell.

*## Save ‘aggr’ Seurat data object*

save(aggr, file = "aggr.RData")

Conclusions

Overall, our first version of the *H. symbiolongicarpus* single-cell atlas was quite promising. The main parameters, including variable features, PCA dimensions, and resolution, were all optimized to create a cell type and cell state atlas that reflects the known biology of the male adult animal including all known major cell types (epithelial cells, neurons, nematocytes, gland cells, i-cells, germ cells, and sperm cells) and some progenitor cell types (nematoblasts, stages of spermatogenesis). Initial annotations for each cluster were obtained using known cell type markers from the literature (2-4) and troubleshooting different Seurat parameters (i.e. adjusting parameters until each cluster had unique markers that differentiated it from all other clusters). After creating our first cell atlas for *H. symbiolongicarpus*, we continued with subcluster analysis to see our map at a higher resolution for potential heterogeneity within the major cell types and cell states.

References

1. Stuart and Butler et al. Comprehensive Integration of Single-Cell Data. Cell (2019) [Seurat V3].
2. Siebert, S. et al. Stem cell differentiation trajectories in *Hydra* resolved at single-cell resolution. Science 365, eaav9314 (2019).
3. Bradshaw, B., Thompson, K. & Frank, U. Distinct mechanisms underlie oral vs aboral regeneration in the cnidarian *Hydractinia echinata*. Elife 4, e05506 (2015).
4. Fincher, C. T., Wurtzel, O., Hoog, T. de, Kravarik, K. M. & Reddien, P. W. Cell type transcriptome atlas for the planarian *Schmidtea mediterranea*. Science 360, eaaq1736 (2018).

SA02 – Subcluster Analysis of interstitial stem cells

*March 27^th^, 2020, updated February 20^th^, 2023*

Summary

This section shows the interstitial stem cell (i-cell) subcluster analysis using our *H. symbiolongicarpus* single-cell atlas. It demonstrates how we extracted the i-cell clusters from the Seurat data object and then re-clustered them to show potential subclusters within this cell type lineage. This analysis does not use the final version of our cell atlas. Our initial subcluster analyses of i-cells and other cell lineages (e.g. epithelial, nematocytes, etc.) showed that further filtration steps in our dataset were required to remove biological artifacts from the data (see section SA03) before generating a final atlas.

Input Files

- " aggr.RData " *(Seurat data object from initial cell cluster analysis)*

Output Files

- " aggr_ic.RData " *(Seurat data object with only i-cell clusters)*

Program Versions

- Seurat version 4.3.0
- R version 4.2.1

i-cell Subcluster Analysis

**library**(Seurat)

**library**(cowplot)

**library**(ggplot2)

**library**(dplyr)

*# Load Seurat data object, make sure file is on your desktop*

load("~/Desktop/aggr.RData")

*# Look at number of genes and cells in the first version of our Seurat data object*

aggr

## An object of class Seurat

## 22034 features across 10237 samples within 1 assay

## Active assay: RNA (22034 features, 0 variable features)

## 3 dimensional reductions calculated: pca, umap

*## Visualize version H. symbiolongicarpus cell type atlas version 1.0*

DimPlot(aggr, reduction = "umap", label = TRUE)


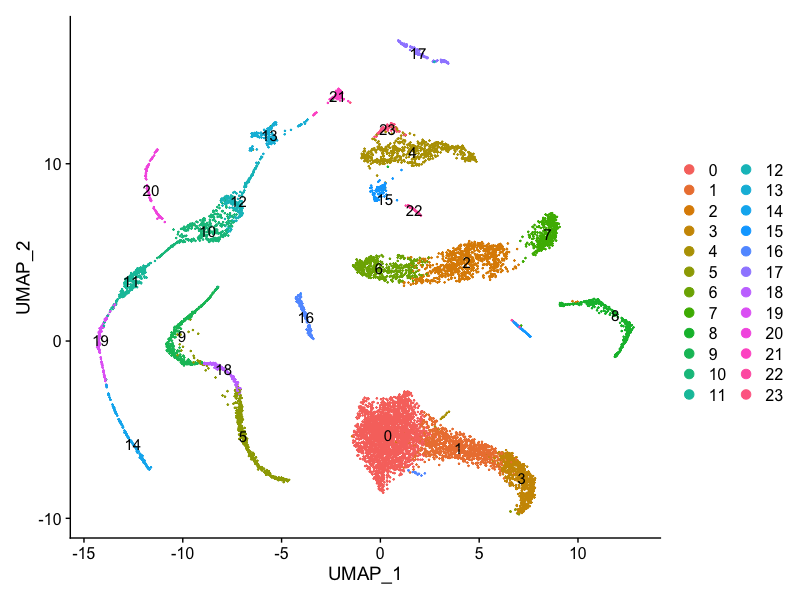


**Figure 1:** UMAP visualization of the H. symbiolongicarpus single-cell atlas version 1.0 labeled with the cluster numbers from the Seurat data object.

We began by extracting the putative i-cell clusters, C10 and C9, from our original single-cell atlas to create a subset Seurat data object called ‘aggr_ic’.

*## Extract i-cell clusters, C10 and C9, and name subset Seurat data object ‘aggr_ic’*

aggr_ic <- subset(aggr, idents = c("10", "9"))

*# Compare original UMAP and subset atlas’*

p1 <- DimPlot(aggr, reduction = "umap", label = TRUE, label.size = 5) + NoLegend()

p2 <- DimPlot(aggr_ic, reduction = "umap", label = TRUE, label.size = 5) + NoLegend()

plot_grid(p1, p2, ncol=2, labels="AUTO", label_size = 20, align = "h")

Cluster C12 was not included in the i-cell subcluster analysis, though our previous UMAP cell atlas shows this cluster as an i-cell/neuronal cluster (ISC/Neuronal). This is because further literature searches showed that the differentially expressed marker genes in this cluster were more neuronal or neuronal progenitor-like, rather than i-cell-like (1-4).


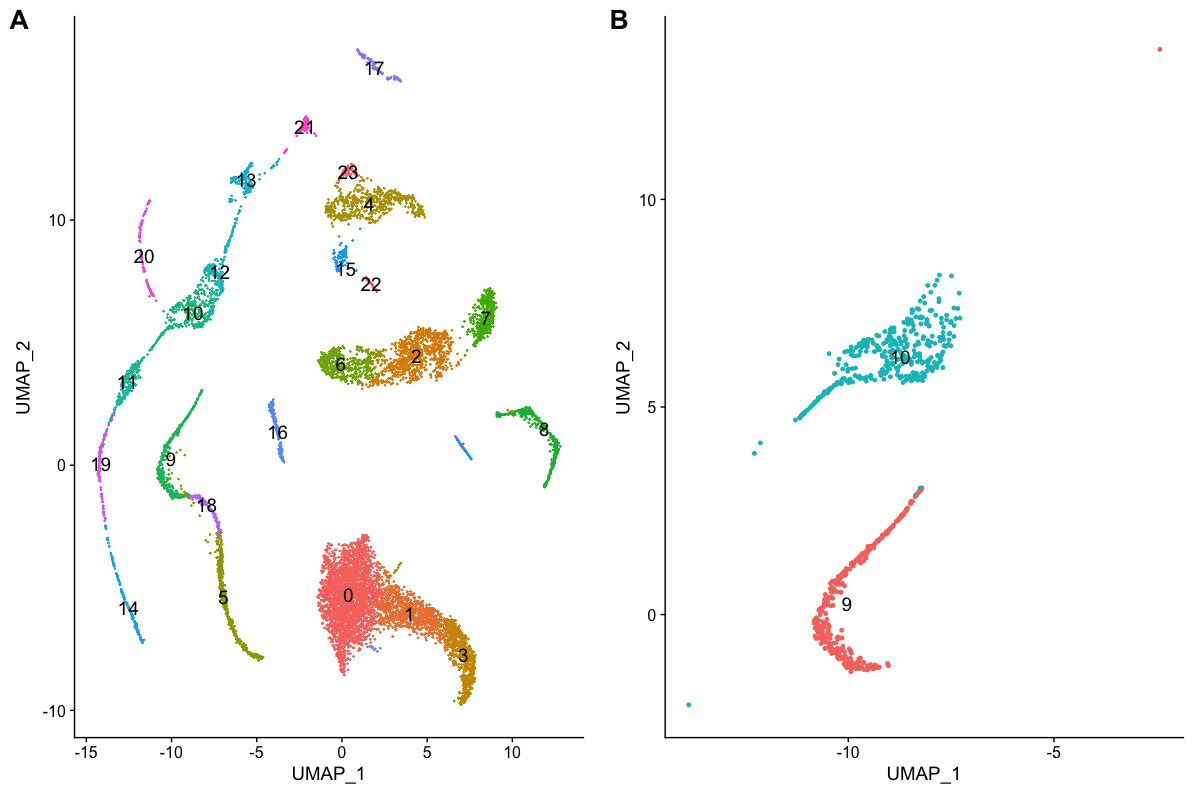


**Figure 2:** (A) UMAP visualization of the H. symbiolongicarpus single-cell atlas version 1.0 labeled with the cluster numbers from the Seurat data object. (B) UMAP visualization showing the subset Seurat data object (aggr_ic) that includes only the putative i-cell clusters, C10 and C9.

Next, the subset i-cell clusters were reanalyzed to create an i-cell subcluster single-cell atlas.

*## Initial re-cluster analysis for i-cell clusters*

*# Lowered variable features from 4000 to 600*

aggr_ic <- FindVariableFeatures(aggr_ic, selection.method = "vst", nfeatures = 600)

all.genes_ic <- rownames(aggr_ic)

aggr_ic <- ScaleData(aggr_ic, features = all.genes_ic)

aggr_ic <- RunPCA(aggr_ic, features = VariableFeatures(object = aggr_ic))

*# Lowered the number of PCA dimensions to use from to 16 to 11*

aggr_ic <- FindNeighbors(aggr_ic, dims = 1:11)

*# Lowered the resolution of our clusters from 0.9 to 0.4*

aggr_ic <- FindClusters(aggr_ic, resolution = 0.4, verbose = FALSE)

aggr_ic <- RunUMAP(aggr_ic, dims = 1:11)

*# Look at number of genes and cells in i-cell subset Seurat data object*

aggr_ic

## An object of class Seurat

## 22034 features across 702 samples within 1 assay

## Active assay: RNA (22034 features, 600 variable features)

## 2 dimensional reductions calculated: pca, umap

The clustering settings had to be lowered to optimize the i-cell subcluster single-cell atlas. This is because we were clustering significantly fewer cells (702 cells in clusters C10 and C9 vs 10,237 cells in the whole dataset) and this data had lower variability (i.e. fewer dimensions in the data were required to accurately show the potential heterogeneity in the i-cell lineage).

*## Save ‘aggr_ic’ Seurat data object*

save(aggr, file = "aggr_ic.RData")

*## Compare original UMAP single-cell atlas with i-cell subcluster single-cell atlas*

p1 <- DimPlot(aggr, reduction = "umap", label = TRUE, label.size = 5, pt.size = 2) + NoLegend() + ggtitle("Original Atlas")

p2 <- subset(aggr, idents = c("10", "9"))

p3 <- DimPlot(p2, reduction = "umap", label = TRUE, label.size = 5, pt.size = 2) + NoLegend() + ggtitle("I-Cell Clusters")

p4 <- DimPlot(aggr_ic, reduction = "umap", label = TRUE, label.size = 5, pt.size = 2) + NoLegend() + ggtitle("I-Cell Subcluster")

plot_grid(p1, p3, p4, ncol=3, labels="AUTO", label_size = 20, align = "h")


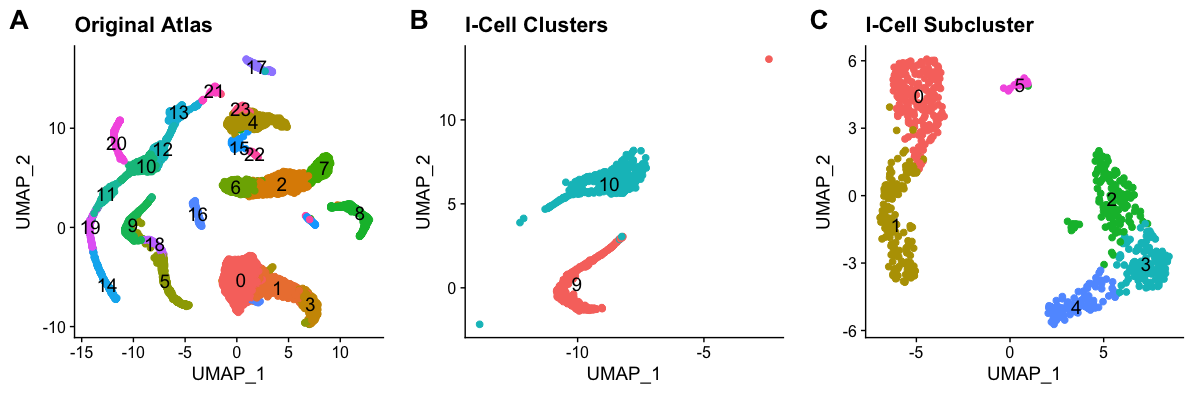


**Figure 3:** (A) UMAP visualization of the H. symbiolongicarpus single-cell atlas version 1.0 labeled with the cluster numbers. (B) UMAP visualization showing only the putative i-cell clusters, C10 and C9. (C) I-cell subcluster single-cell atlas showing six separate clusters.

We investigated the top markers from each of the i-cell subclusters shown in Figure 3C to try to understand if the different clusters were progenitors or pre-progenitor stem cell states for the other cell lineages. The expression pattern of these top i-cell subcluster markers were examined in the *H. symbiolongicarpus* single-cell atlas to see if they could provide clues as to which cell lineage they might be a precursor.

Exploring Top Markers from i-Cell Subcluster Analysis

*## Finding the top markers from each cluster in the i-cell subcluster cell atlas*

aggr_ic.markers <- FindAllMarkers(aggr_ic, only.pos = TRUE, min.pct = 0.25, logfc.threshold = 0.25)

*# Top 50 markers from each cluster*

top_50_markers_per_cluster <- aggr_ic.markers %>% group_by(cluster) %>% top_n(n = 50, wt = avg_log2FC)

*## Top six marker genes for cluster 0*

Top6_C0_ic <- top_50_markers_per_cluster$gene[1:6]

p1 <- FeaturePlot(aggr_ic, features = Top6_C0_ic, reduction = "umap", ncol = 3, label = TRUE, label.size = 5)

p2 <- FeaturePlot(aggr, features = Top6_C0_ic, reduction = "umap", ncol = 3)

plot_grid(p1, p2, ncol=1, labels="AUTO", label_size = 20, align = "h")


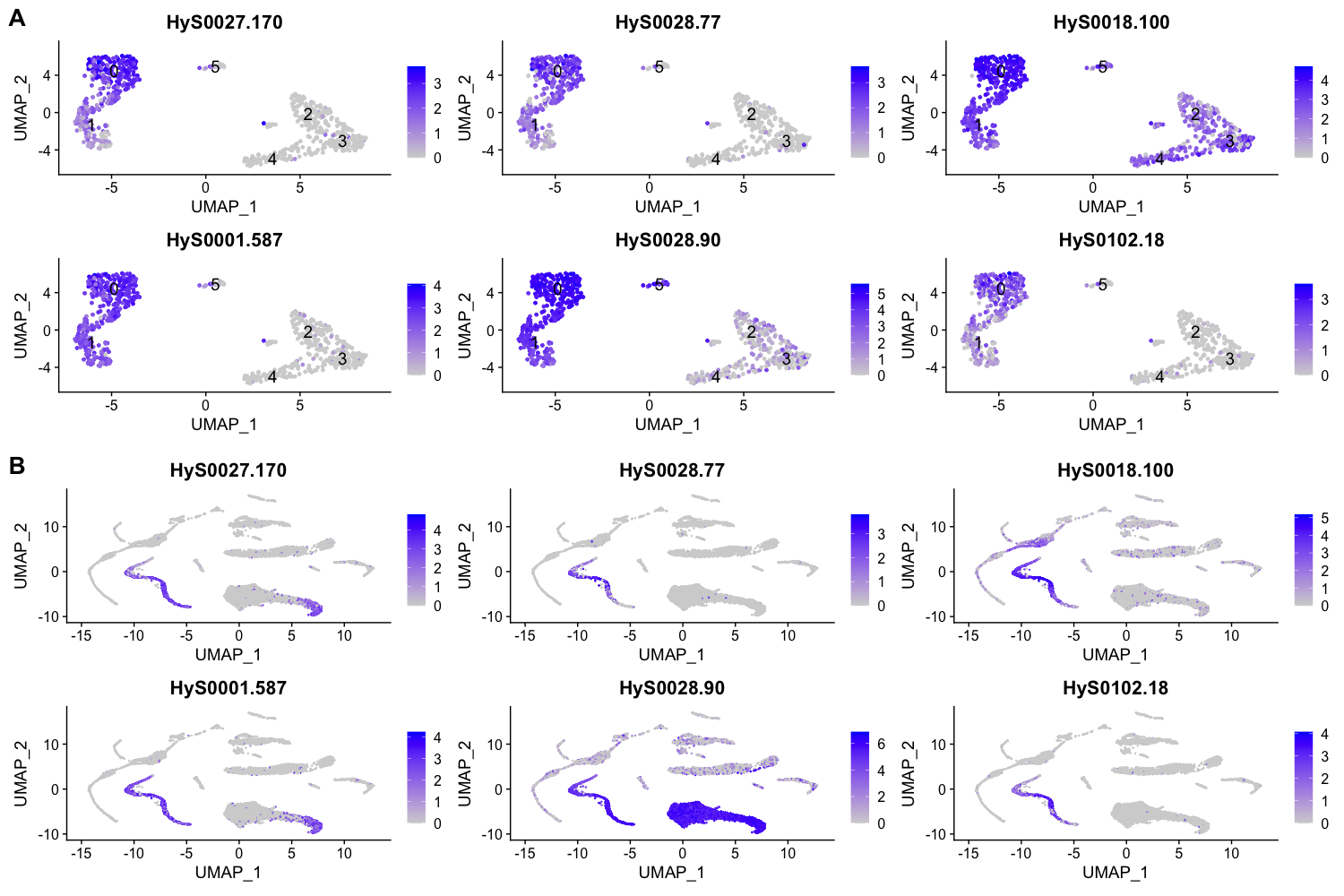


**Figure 4:** (A) The expression pattern of the top six markers from cluster C0 in the i-cell subcluster cell atlas. (B) The same markers from (A), but their expression pattern in the overall H. symbiolongicarpus single-cell atlas.

The comparison of the top six markers from cluster C0 in the i-cell subcluster cell atlas with the *H. symbiolongicarpus* single-cell atlas in Figure 4 hints that this subcluster is involved in germ cells through early spermatogenesis.

*# Top 6 six marker genes for cluster 1*

Top6_C1_ic <- top_50_markers_per_cluster$gene[51:56]

p1 <- FeaturePlot(aggr_ic, features = Top6_C1_ic, reduction = "umap", ncol = 3, label = TRUE, label.size = 5)

p2 <- FeaturePlot(aggr, features = Top6_C1_ic, reduction = "umap", ncol = 3)

plot_grid(p1, p2, ncol=1, labels="AUTO", label_size = 20, align = "h")


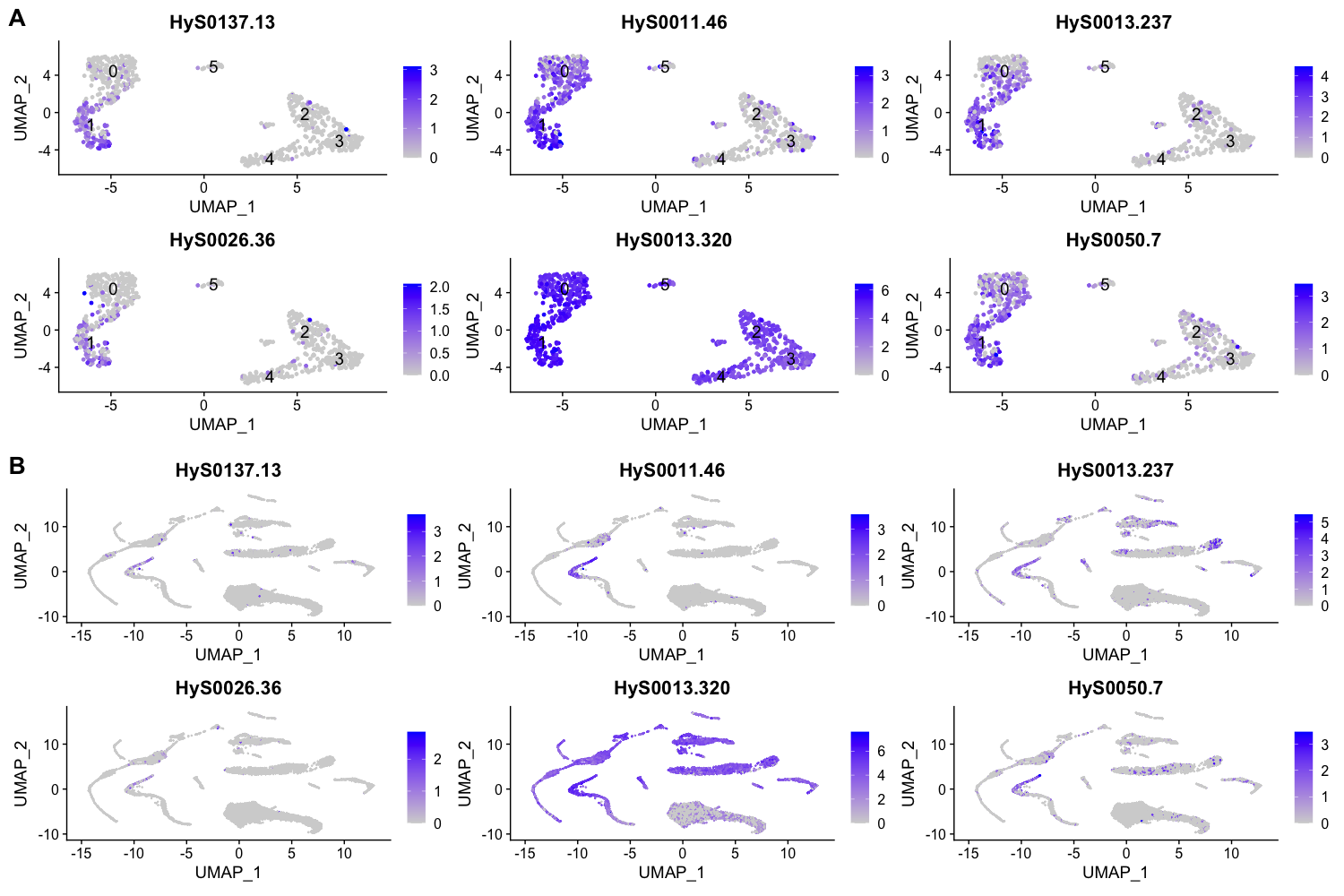


**Figure 5:** (A) The expression pattern of the top six markers from cluster C1 in the i-cell subcluster cell atlas. (B) The same markers from (A), but their expression pattern in the overall H. symbiolongicarpus single-cell atlas.

Like C0, the C1 i-cell subcluster might be involved in germ cells to early spermatogenesis (Figure 5). However, some of its top markers seem to be expressed in all other cell clusters, hinting that these stem cells could have a greater potency to create other cell lineages.

*# Top 6 six marker genes for cluster 2*

Top6_C2_ic <- top_50_markers_per_cluster$gene[101:106]

p1 <- FeaturePlot(aggr_ic, features = Top6_C2_ic, reduction = "umap", ncol = 3, label = TRUE, label.size = 5)

p2 <- FeaturePlot(aggr, features = Top6_C2_ic, reduction = "umap", ncol = 3)

plot_grid(p1, p2, ncol=1, labels="AUTO", label_size = 20, align = "h")


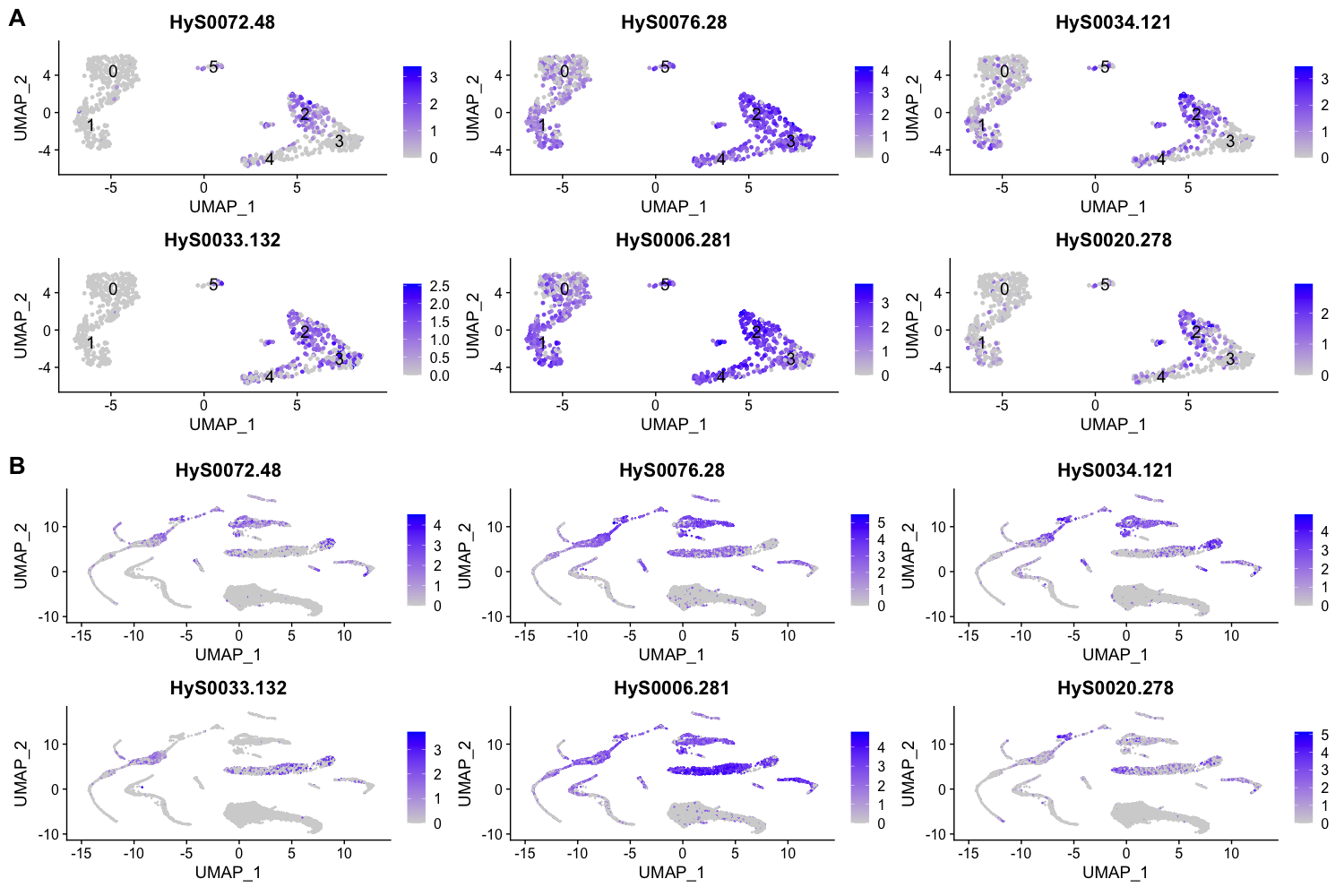


**Figure 6:** (A) The expression pattern of the top six markers from cluster C2 in the i-cell subcluster cell atlas. (B) The same markers from (A), but their expression pattern in the overall H. symbiolongicarpus single-cell atlas.

C2 is like C1, where several of its top markers are expressed in all clusters from the *H. symbiolongicarpus* single-cell atlas, possibly indicating that it is a cluster of stem cells with greater potency.

*# Top 6 six marker genes for cluster 3*

Top6_C3_ic <- top_50_markers_per_cluster$gene[151:156]

p1 <- FeaturePlot(aggr_ic, features = Top6_C3_ic, reduction = "umap", ncol = 3, label = TRUE, label.size = 5)

p2 <- FeaturePlot(aggr, features = Top6_C3_ic, reduction = "umap", ncol = 3)

plot_grid(p1, p2, ncol=1, labels="AUTO", label_size = 20, align = "h")


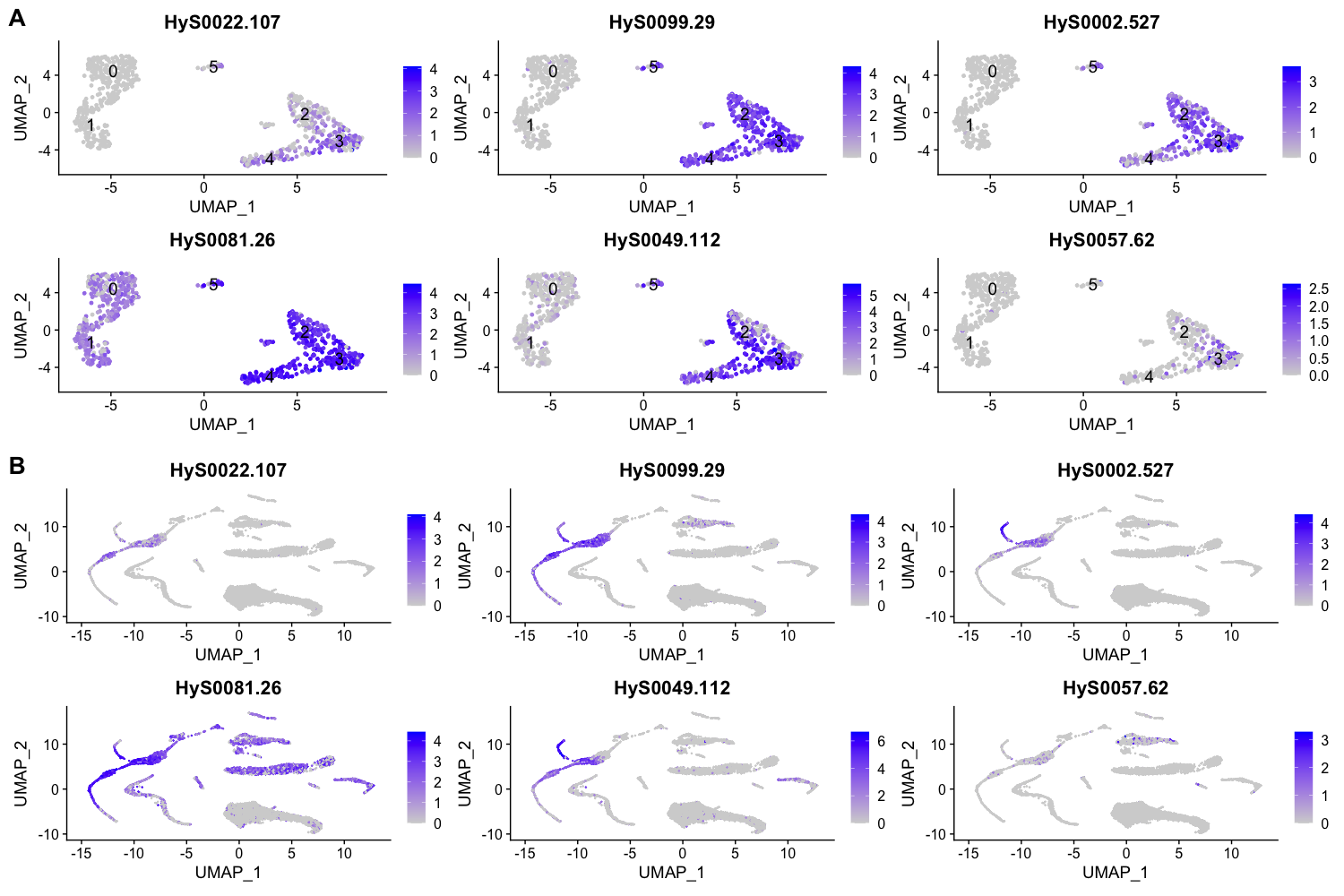


**Figure 7:** (A) The expression pattern of the top six markers from cluster C3 in the i-cell subcluster cell atlas. (B) The same markers from (A), but their expression pattern in the overall H. symbiolongicarpus single-cell atlas.

C3 is presumably i-cells that are committed to becoming nematoblasts. Interestingly, some C3 marker genes are specifically expressed primarily in one nematoblast cluster (Cluster 19 from the version 1 *H. symbiolongicarpus* single-cell atlas) which may mean this subcluster includes progenitors heading toward a specific nematocyte fate, as there are several different nematocyte types in hydrozoan cnidarians (5). This hypothesis would need to be tested experimentally to confirm what this subcluster really represents.

*# Top 6 six marker genes for cluster 4*

Top6_C4_ic <- top_50_markers_per_cluster$gene[201:206]

p1 <- FeaturePlot(aggr_ic, features = Top6_C4_ic, reduction = "umap", ncol = 3, label = TRUE, label.size = 5)

p2 <- FeaturePlot(aggr, features = Top6_C4_ic, reduction = "umap", ncol = 3)

plot_grid(p1, p2, ncol=1, labels="AUTO", label_size = 20, align = "h")


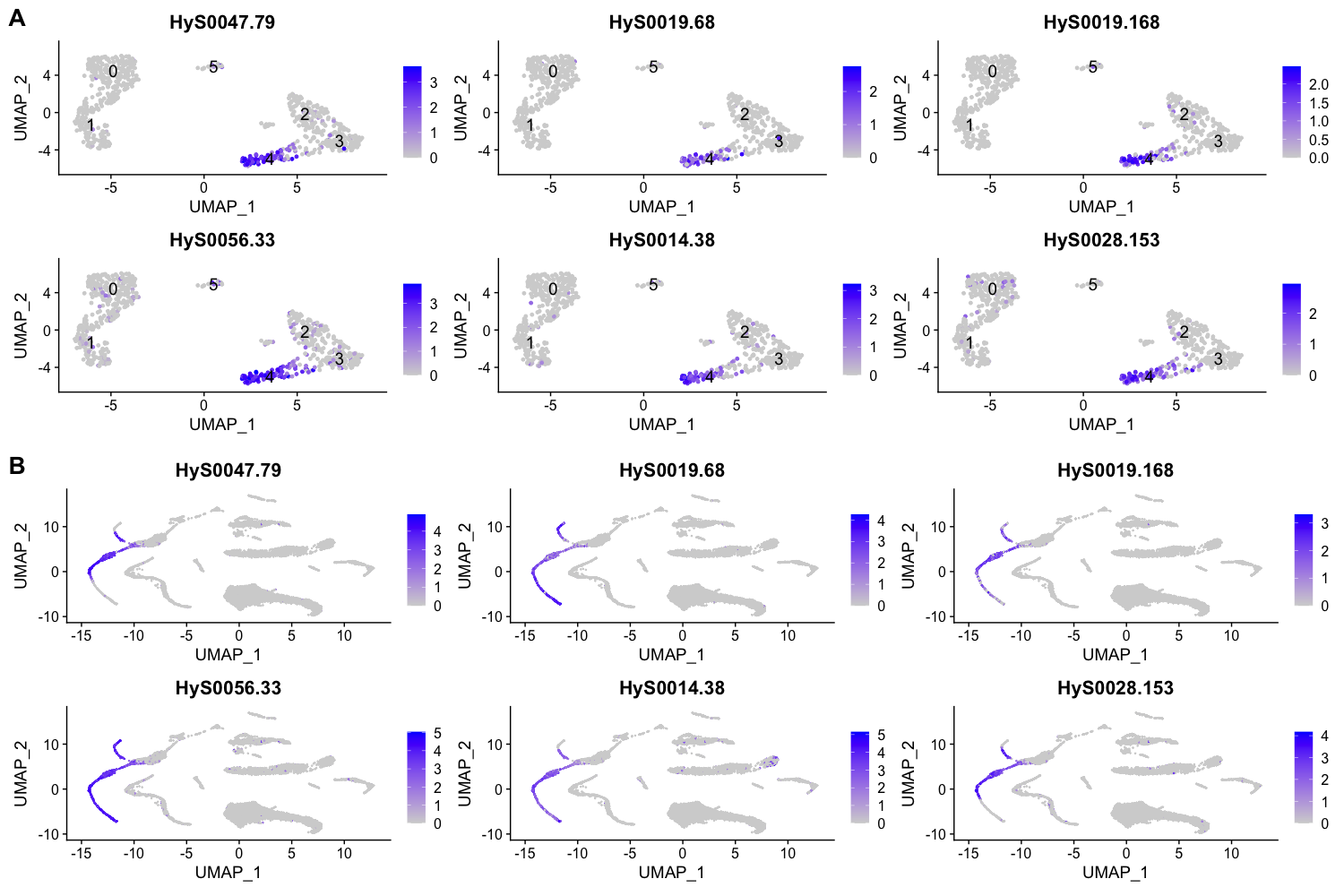


**Figure 8:** (A) The expression pattern of the top six markers from cluster C4 in the i-cell subcluster cell atlas. (B) The same markers from (A), but their expression pattern in the H. symbiolongicarpus single-cell atlas.

C4 is another i-cell subcluster that seems to be committed to a nematoblast fate. Further experimental confirmation would be necessary to fully interpret what these subclusters represent.

*# Top 6 six marker genes for cluster 5*

Top6_C5_ic <- top_50_markers_per_cluster$gene[251:256]

p1 <- FeaturePlot(aggr_ic, features = Top6_C5_ic, reduction = "umap", ncol = 3, label = TRUE, label.size = 5)

p2 <- FeaturePlot(aggr, features = Top6_C5_ic, reduction = "umap", ncol = 3)

plot_grid(p1, p2, ncol=1, labels="AUTO", label_size = 20, align = "h")


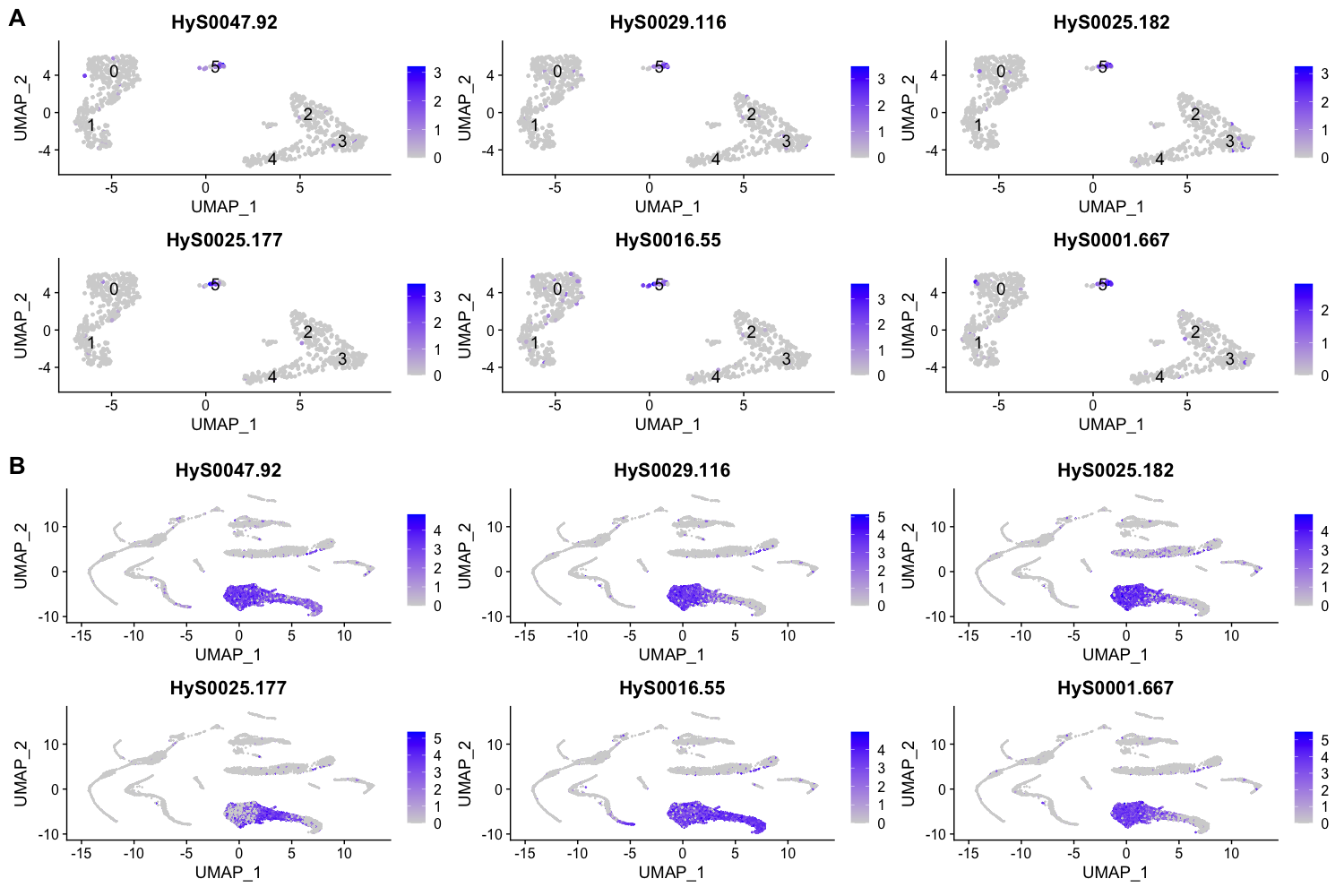


**Figure 9:** (A) The expression pattern of the top six markers from cluster C5 in the i-cell subcluster cell atlas. (B) The same markers from (A), but their expression pattern in the H. symbiolongicarpus single-cell atlas.

C5 was initially a cryptic i-cell subcluster since the top marker genes were all expressed in the putative mature sperm cell cluster C0 from the *H. symbiolongicarpus* single-cell atlas. We determined that this phenomenon was an artifact in the data after subcluster analyses from other cell lineages, including terminally differentiated cell types like nematocytes, also had small clusters whose top markers were mature sperm cell markers. This led us to further filter our dataset to get rid of these small mature sperm clusters from our subcluster analyses (see section SA03).

Conclusions

The initial i-cell subcluster analysis looked promising. The main parameters, including variable features, PCA dimensions, and resolution, were all lowered/optimized since we were repeating the analysis with a subset of cells (e.g. lower heterogeneity). The subcluster analysis was performed with different Seurat parameters to create realistic clusters (i.e. each subcluster had unique markers that differentiated it from all other clusters). However, one issue we discovered in the i-cell subcluster analysis was concerning C5. It was fairly small compared to the others and all the top markers had expression in our mature sperm cell clusters. We then repeated subclustering analysis with several other cell lineages and saw similar results (a small cluster whose top markers were expressed in mature sperm cells). This observation, and the fact that a large portion of our dataset was mature sperm cells, led us to believe there might be a biological doublet problem within our data. A small, but significant, portion of cells in our single-cell atlas are likely a combination of a small sperm cell that was encapsulated with larger cells from other lineages during the 10X single-cell chromium run. We call these cells ‘mature sperm doublets.’ See section SA03, “*Hydractinia* Single-Cell Atlas Filtration,” for more details and our strategy to filter mature sperm doublets from the dataset.

References

1. Gajewski, M., Leitz, T., Schloßherr, J. & Plickert, G. LWamides from Cnidaria constitute a novel family of neuropeptides with morphogenetic activity. Roux’s archives of developmental biology 205, 232–242 (1996).
2. Katsukura, Y., David, C. N., Grimmelikhuijzen, C. J. P. & Sugiyama, T. Inhibition of metamorphosis by RFamide neuropeptides in planula larvae of Hydractinia echinata. Development Genes and Evolution 213, 579–586 (2003).
3. Nakanishi, N., Renfer, E., Technau, U. & Rentzsch, F. Nervous systems of the sea anemone Nematostella vectensis are generated by ectoderm and endoderm and shaped by distinct mechanisms. Development 139, 347–357 (2012).
4. Siebert, S. et al. Stem cell differentiation trajectories in *Hydra* resolved at single-cell resolution. Science 365, eaav9314 (2019).
5. David, C. N. et al. Evolution of complex structures: minicollagens shape the cnidarian nematocyst. Trends in Genetics 24, 431–438 (2008).

SA03 – *Hydractinia* Single-Cell Atlas Filtration

*April 14^th^, 2020, updated February 20^th^, 2023*

Summary

This section shows how we filtered the *H. symbiolongicarpus* single-cell atlas to remove mature sperm doublets from the original dataset. Mature sperm doublets are 10X single-cell samples (i.e. cells) where a mature sperm cell was encapsulated with a larger cell (see Figure 1 below), causing some samples in our dataset to have a mixture of cell type markers. We discovered this when subclustering different cell lineages and found that all had a small subcluster where the top markers were mature sperm cell markers (see section SA02). Here we explore different methods to correct this issue, as well as the final version of our *H. symbiolongicarpus* single-cell atlas.

Input Files

- " aggr.RData " *(Seurat data object from initial cell cluster analysis)*
- " aggr_ic.RData " *(Seurat data object with only i-cell clusters)*

Output Files

- " aggr_fltd.RData " *(Seurat data object of our* ***final H. symbiolongicarpus single-cell atlas****)*
- "aggr_cutoff.RData" *(Seurat data object of an alternate filtration method for our data)*

Program Versions

- Seurat version 4.3.0
- R version 4.2.1


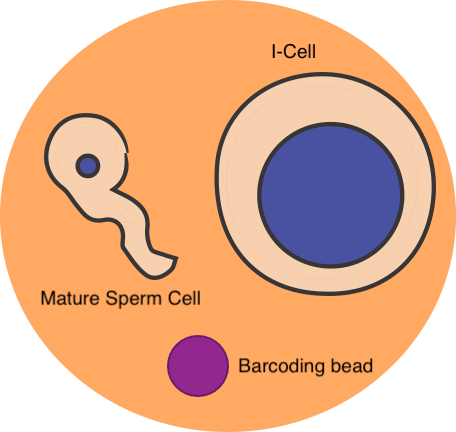


**Figure 1:** Schematic showing a possible mature sperm doublet in our H. symbiolongicarpus single-cell atlas. In this example, a mature sperm cell was encapsulated with an i-cell during the 10X single-cell Chromium run, with the barcoding bead eventually labeling transcripts from both cells, creating an artificial sample in our dataset.

Filtering Mature Sperm Doublets

There were two main methods used to remove mature sperm doublets from our single-cell dataset:

1. Searching for non-mature sperm cells expressing mature sperm cell markers. We temporarily removed three sperm cell clusters (C0, C1, and C3 from the *H. symbiolongicarpus* single-cell atlas version 1.0) and searched the remaining clusters for expression of a select few chosen mature sperm cell markers. These ‘filter’ markers are HyS0001.667, HyS0011.54, HyS0052.25, HyS0003.260, HyS0010.433, and HyS0002.511. All filter markers were exclusively expressed in our mature sperm cell cluster C0, to be confident that we were only filtering out real biological doublets that contained mature sperm. We discarded the remaining cells that had any expression of the filter markers, combined the filtered clusters with the mature sperm clusters we temporarily removed, and repeated the cluster analysis to create an improved version of the *H. symbiolongicarpus* single-cell atlas.
2. Filtering out cells that had a high total of RNA features. We reasoned that cells that are encapsulated together should have a higher total number of RNA features (i.e. genes detected in a cell). We tested different RNA feature cutoffs to remove the doublets from our *H. symbiolongicarpus* single-cell atlas. An RNA feature cutoff of 1815 was chosen since it gave us roughly the same number of cells as the filtration method from the method described above, making them easier to compare.

Filtering Method 1: Filtering by Mature Sperm Cell Marker Expression

**library**(Seurat)

**library**(ggplot2)

*## Load the "aggr" Seurat Object*

*# Load Seurat data object, make sure file is on your desktop*

load("~/Desktop/aggr.RData")

aggr

## An object of class Seurat

## 22034 features across 10237 samples within 1 assay

## Active assay: RNA (22034 features, 0 variable features)

## 3 dimensional reductions calculated: pca, umap

*## Visualize the clusters using UMAP (figure 1)*

DimPlot(aggr, reduction = "umap", label = TRUE)


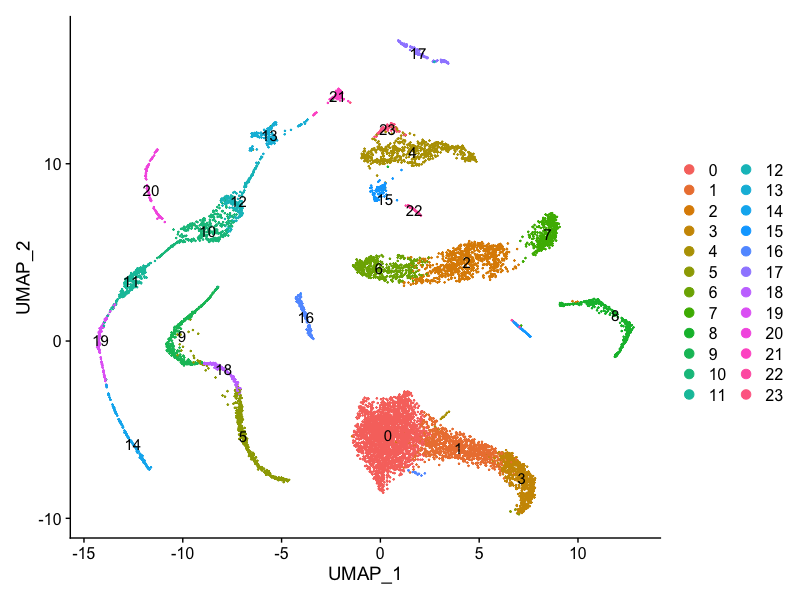


**Figure 2:** UMAP visualization of the H. symbiolongicarpus single-cell atlas version 1.0 labeled with the cluster numbers from the Seurat data object.

We originally chose five filter markers in our first attempt at getting rid of mature sperm doublets from our non-mature sperm clusters. The mature sperm clusters from our *H. symbiolongicarpus* single-cell atlas were removed (C0, C1, and C3) and the remaining clusters were filtered.

*## Filtering dataset method 1*

*# Temporarily remove mature sperm cell clusters 0/1/3 from original dataset*

aggr_rid <- subset(aggr, idents = c("2", "4", "5", "6", "7", "8", "9",

"10", "11", "12", "13", "14", "15",

"16", "17", "18", "19", "20", "21",

"22", "23"))

*# Grabbing mature sperm clusters that were removed*

sperm_cells <- WhichCells(aggr, idents = c("0", "1", "3"))

*# Removing non-mature sperm cells that expressed marker "HyS0001.667"*

rid_expression <- GetAssayData(object = aggr_rid, assay = "RNA", slot = "data")["HyS0001.667",]

neg_ids <- names(which(rid_expression == 0))

aggr_fltd <- subset(aggr, cells = c(neg_ids, sperm_cells))

*# Removing non-mature sperm cells that expressed marker "HyS0011.54"*

rid_expression <- GetAssayData(object = aggr_rid, assay = "RNA", slot = "data")["HyS0011.54",]

neg_ids <- names(which(rid_expression == 0))

aggr_fltd <- subset(aggr_fltd, cells = c(neg_ids, sperm_cells))

*# Removing non-mature sperm cells that expressed marker "HyS0052.25"*

rid_expression <- GetAssayData(object = aggr_rid, assay = "RNA", slot = "data")["HyS0052.25",]

neg_ids <- names(which(rid_expression == 0))

aggr_fltd <- subset(aggr_fltd, cells = c(neg_ids, sperm_cells))

*# Removing non-mature sperm cells that expressed marker "HyS0003.260"*

rid_expression <- GetAssayData(object = aggr_rid, assay = "RNA", slot = "data")["HyS0003.260",]

neg_ids <- names(which(rid_expression == 0))

aggr_fltd <- subset(aggr_fltd, cells = c(neg_ids, sperm_cells))

*# Removing non-mature sperm cells that expressed marker "HyS0010.433"*

rid_expression <- GetAssayData(object = aggr_rid, assay = "RNA", slot = "data")["HyS0010.433",]

neg_ids <- names(which(rid_expression == 0))

aggr_fltd <- subset(aggr_fltd, cells = c(neg_ids, sperm_cells))

aggr_fltd

## An object of class Seurat

## 22034 features across 9038 samples within 1 assay

## Active assay: RNA (22034 features, 4000 variable features)

## 2 dimensional reductions calculated: pca, umap

This method removed 1,199 cells from our *H. symbiolongicarpus* single-cell atlas, and we reclustered the remaining 9,038 cells to create version two of our atlas.

*## Reclustering filtered dataset aggr_fltd (1/2)*

aggr_fltd <- FindVariableFeatures(aggr_fltd, selection.method = "vst", nfeatures = 4000)

all.genes <- rownames(aggr_fltd)

aggr_fltd <- ScaleData(aggr_fltd, features = all.genes)

aggr_fltd <- RunPCA(aggr_fltd, features = VariableFeatures(object = aggr_fltd))

aggr_fltd <- FindNeighbors(aggr_fltd, dims = 1:16)

*# Changed from 0.9 to 0.7*

aggr_fltd <- FindClusters(aggr_fltd, resolution = 0.7, verbose = FALSE)

aggr_fltd <- RunUMAP(aggr_fltd, dims = 1:16)

DimPlot(aggr_fltd, reduction = "umap", pt.size = 1.5, label = TRUE)


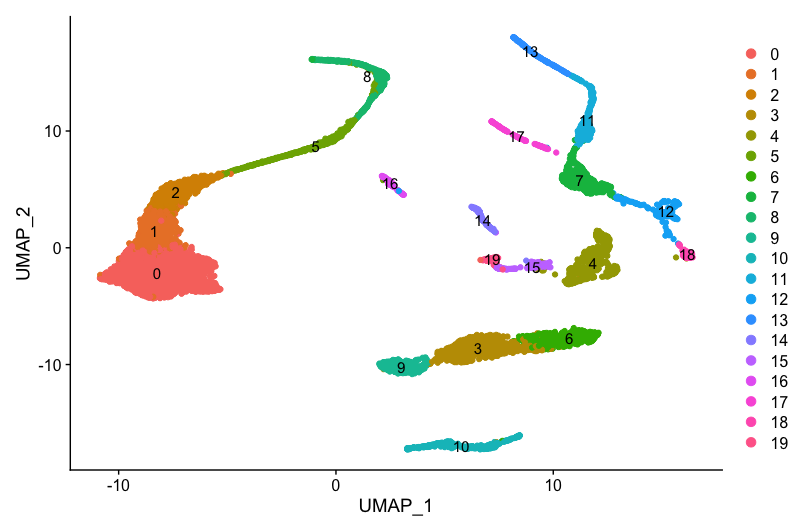


**Figure 3:** UMAP visualization of the H. symbiolongicarpus single-cell atlas version 2.0 labeled with the cluster numbers from the Seurat data object. Approximately 1,199 mature sperm doublets were removed from our original dataset, which now totals 9,038 cells.

The *H. symbiolongicarpus* single-cell atlas version 2.0 was an improvement over our first attempt at clustering our single-cell data. However, we still found some mature sperm cells in our cell lineage subcluster analyses. This led us to filter the dataset a second time with one more mature sperm cell marker gene (HyS0002.511). This time we removed all spermatogenesis clusters (C0, C1, C2, C5, and C8 from the *H. symbiolongicarpus* single-cell atlas version 2.0) to avoid excluding any real sperm or spermatogonia cells that might express this gene in earlier progenitor states.

*## Filter mature sperm doublets from version 2.0 of our atlas*

*# Temporarily remove sperm cell clusters 0/1/2/5/8 from filtered aggr_fltd dataset*

aggr_rid <- subset(aggr_fltd, idents = c("3", "4", "6", "7", "9", "10",

"11", "12", "13", "14", "15",

"16", "17", "18", “19”))

*# Grabbing mature sperm clusters that were removed*

sperm_cells <- WhichCells(aggr_fltd, idents = c("0", "1", "2", "5", "8"))

*# Removing non-mature sperm cells that expressed marker "HyS0002.511"*

rid_expression <- GetAssayData(object = aggr_rid, assay = "RNA", slot = "data")["HyS0002.511",]

neg_ids <- names(which(rid_expression == 0))

aggr_fltd <- subset(aggr_fltd, cells = c(neg_ids, sperm_cells))

aggr_fltd

## An object of class Seurat

## 22034 features across 8888 samples within 1 assay

## Active assay: RNA (22034 features, 4000 variable features)

## 2 dimensional reductions calculated: pca, umap

Our second filtration removed an additional 150 cells from our *H. symbiolongicarpus* single-cell dataset, now totaling 8,888 cells. We reclustered the new dataset and show version 3.0 of our atlas in Figure 4.

*## Reclustering filtered dataset aggr_fltd (2/2)*

aggr_fltd <- FindVariableFeatures(aggr_fltd, selection.method = "vst", nfeatures = 4000)

all.genes <- rownames(aggr_fltd)

aggr_fltd <- ScaleData(aggr_fltd, features = all.genes)

aggr_fltd <- RunPCA(aggr_fltd, features = VariableFeatures(object = aggr_fltd))

aggr_fltd <- FindNeighbors(aggr_fltd, dims = 1:15)

aggr_fltd <- FindClusters(aggr_fltd, resolution = 0.6, verbose = FALSE)

aggr_fltd <- RunUMAP(aggr_fltd, dims = 1:15)

DimPlot(aggr_fltd, reduction = "umap", pt.size = 1.5, label = TRUE)


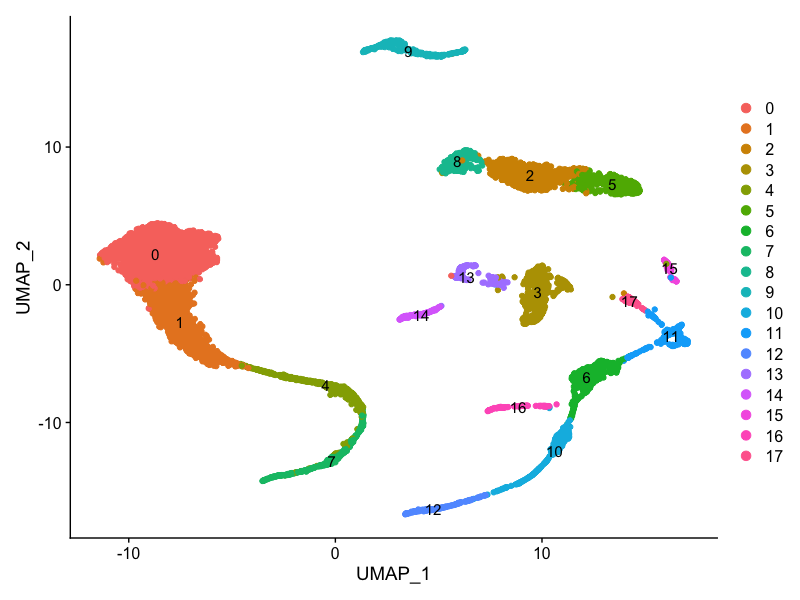


**Figure 4:** UMAP visualization of the H. symbiolongicarpus single-cell atlas version 3.0 labeled with the cluster numbers from the Seurat data object. Exactly 1,349 mature sperm doublets were removed from our original dataset, which now totals 8,888 cells.

The *H. symbiolongicarpus* single-cell atlas version 3.0 is our final version of our dataset and the version we discuss in the paper. We found no more small clusters that exclusively expressed mature sperm cell markers in our cell lineage subcluster analyses. See below for an alternative filtration method that we tried to improve our single-cell atlas dataset.

*## Save "aggr_fltd" Seurat object*

save(aggr_fltd, file = "aggr_fltd.RData")

Filtering Method 2: Removing Cells with Higher Content of Genes

**library**(Seurat)

**library**(ggplot2)

*# Start by loading raw data and creating a merged Seurat object*

# Set the working directory to the folder containing the data

setwd("~/Desktop/Updating_Seurat")

*# Create a Seurat object for each sample*

for (file in c("3XPBS_filtered_feature_bc_matrix", "Seawater_filtered_feature_bc_matrix")){

seurat_data <- Read10X(data.dir = paste0("1_Cell_rangerV7_libraries/", file))

seurat_obj <- CreateSeuratObject(counts = seurat_data,

min.features = 100,

project = file)

assign(file, seurat_obj)

}

*# Merge libraries into a single Seurat object*

aggr <- merge(x=`3XPBS_filtered_feature_bc_matrix`,

y=Seawater_filtered_feature_bc_matrix,

add.cell.id = c("3XPBS", "Seawater"))

*# Create a copy of the "aggr" raw dataset and name it "aggr_cutoff"*

aggr_cutoff <- aggr

*# Look at number of genes and cells in the first version of our Seurat data object*

aggr_cutoff

## An object of class Seurat

## 22034 features across 10237 samples within 1 assay

## Active assay: RNA (22034 features, 0 variable features)

We began by loading the raw data and pre-processing the dataset like we show above in the previous sections.

*# Removing cells with high mitochondrial RNA*

aggr_cutoff[["percent.mt"]] <- PercentageFeatureSet(aggr_cutoff, pattern = "^HyS0613.")

The subset function in Seurat was used to remove cells with a high number of RNA content (i.e genes). We aimed to set a cutoff that would give us a similar number of cells to our *H. symbiolongicarpus* single-cell version 3.0 atlas (8,888 cells). A new dataset called ‘aggr_cutoff’ we created using a cutoff of 1,900 or less nFeature_RNA (i.e. genes expressed in one cell). We reclustered the new dataset and show version 3.1 of our atlas in Figure 5.

*# Filter data based on total number of RNA features*

*# Aiming for total cell count like aggr_fltd (8,888 cells)*

aggr_cutoff <- subset(aggr_cutoff, subset = nFeature_RNA > 200 & nFeature_RNA < 1900 & percent.mt < 5)

aggr_cutoff

## An object of class Seurat

## 22034 features across 8887 samples within 1 assay

## Active assay: RNA (22034 features, 0 variable features)

*## Cell Cluster Settings for aggr_cutoff*

aggr_cutoff <- NormalizeData(aggr_cutoff, normalization.method = "LogNormalize", scale.factor = 10000)

aggr_cutoff <- FindVariableFeatures(aggr_cutoff, selection.method = "vst", nfeatures = 4000)

all.genes <- rownames(aggr_cutoff)

aggr_cutoff <- ScaleData(aggr_cutoff, features = all.genes)

aggr_cutoff <- RunPCA(aggr_cutoff, features = VariableFeatures(object = aggr_cutoff))

aggr_cutoff <- FindNeighbors(aggr_cutoff, dims = 1:15)

aggr_cutoff <- FindClusters(aggr_cutoff, resolution = 0.6, verbose = FALSE)

aggr_cutoff <- RunUMAP(aggr_cutoff, dims = 1:15)

DimPlot(aggr_cutoff, reduction = "umap", pt.size = 1.5, label = TRUE)


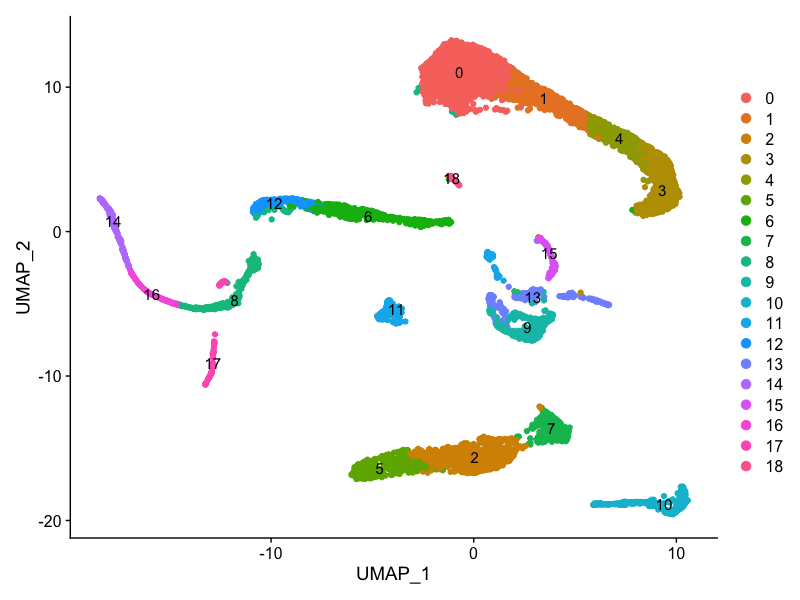


**Figure 5:** UMAP visualization of the H. symbiolongicarpus single-cell atlas version 3.1 labeled with the cluster numbers from the Seurat data object. Exactly 1,348 cells with high gene content were removed from our original dataset, which now totals 8,887 cells.

The *H. symbiolongicarpus* single-cell atlas version 3.1 is an alternate version of our dataset that shows a different attempt to remove mature sperm doublets. We did not end up choosing this version of our dataset because it likely filters out real cells that have a high gene content (number of different genes detected), such as gland cells. See below for comparison of our filtration methods and their respective i-cell subcluster analyses.

*## Save "aggr_cutoff" Seurat object*

save(aggr_cutoff, file = "aggr_cutoff.RData")

Comparing Filtering Methods and their i-cell Subcluster Analyses

**library**(Seurat)

**library**(cowplot)

**library**(ggplot2)

*# Load Seurat data objects, make sure files are on your desktop*

load("~/Desktop/aggr.RData")

load("~/Desktop/aggr_ic.RData")

load("~/Desktop/aggr_fltd.RData")

load("~/Desktop/aggr_cutoff.RData")

We began by loading previous Seurat data objects for each of the versions of our *H. symbiolongicarpus* single-cell atlas. I-cell subcluster analysis was performed for versions 3.0 and 3.1 of our single-cell atlas with the same settings used in our 1.0 version. We checked the i-cell subcluster analyses by looking at the expression of the mature sperm cell marker HyS0001.667.

*## Original I-cell Subclustering (no filtering)*

p1 <- DimPlot(aggr, reduction = "umap", pt.size = 1.5) + NoLegend()

p2 <- DimPlot(aggr_ic, reduction = "umap", label = TRUE)

p3 <- FeaturePlot(aggr_ic, features = c("HyS0001.667"), label = TRUE)

*## I-cell subcluster analysis for H. symbiolongicarpus single-cell atlas version 3.0*

aggr_ic2 <- subset(aggr_fltd, idents = c("7","6"))

aggr_ic2 <- FindVariableFeatures(aggr_ic2, selection.method = "vst", nfeatures = 600)

all.genes_ic <- rownames(aggr_ic2)

aggr_ic2 <- ScaleData(aggr_ic2, features = all.genes_ic)

aggr_ic2 <- RunPCA(aggr_ic2, features = VariableFeatures(object = aggr_ic2))

aggr_ic2 <- FindNeighbors(aggr_ic2, dims = 1:11)

aggr_ic2 <- FindClusters(aggr_ic2, resolution = 0.4, verbose = FALSE)

aggr_ic2 <- RunUMAP(aggr_ic2, dims = 1:11)

aggr_ic2 <- RunTSNE(aggr_ic2, dims = 1:11, check_duplicates = FALSE)

p4 <- DimPlot(aggr_fltd, reduction = "umap", pt.size = 1.5) + NoLegend()

p5 <- DimPlot(aggr_ic2, reduction = "umap", label = TRUE)

*# Expression chart colors were changed using "cols" argument since no cells have expression*

*# of HyS0001.667 and were lightly shaded purple with an expression value of 0*

p6 <- FeaturePlot(aggr_ic2, features = c("HyS0001.667"), label = TRUE)

*## I-cell subcluster analysis for H. symbiolongicarpus single-cell atlas version 3.1*

aggr_ic3 <- subset(aggr_cutoff, idents = c("12","8"))

aggr_ic3 <- FindVariableFeatures(aggr_ic3, selection.method = "vst", nfeatures = 600)

all.genes_ic <- rownames(aggr_ic3)

aggr_ic3 <- ScaleData(aggr_ic3, features = all.genes_ic)

aggr_ic3 <- RunPCA(aggr_ic3, features = VariableFeatures(object = aggr_ic3))

aggr_ic3 <- FindNeighbors(aggr_ic3, dims = 1:11)

aggr_ic3 <- FindClusters(aggr_ic3, resolution = 0.4, verbose = FALSE)

aggr_ic3 <- RunUMAP(aggr_ic3, dims = 1:11)

aggr_ic3 <- RunTSNE(aggr_ic3, dims = 1:11, check_duplicates = FALSE)

p7 <- DimPlot(aggr_cutoff, reduction = "umap", pt.size = 1.5) + NoLegend()

p8 <- DimPlot(aggr_ic3, reduction = "umap", label = TRUE)

p9 <- FeaturePlot(aggr_ic3, features = c("HyS0001.667"), label = TRUE)

*## Comparing the different versions of the H. symbiolongicarpus single-cell atlas’*

plot_grid(p1, p2, p3, p4, p5, p6, p7, p8, p9, ncol=3, align = "h", labels = "AUTO", label_size = 20)


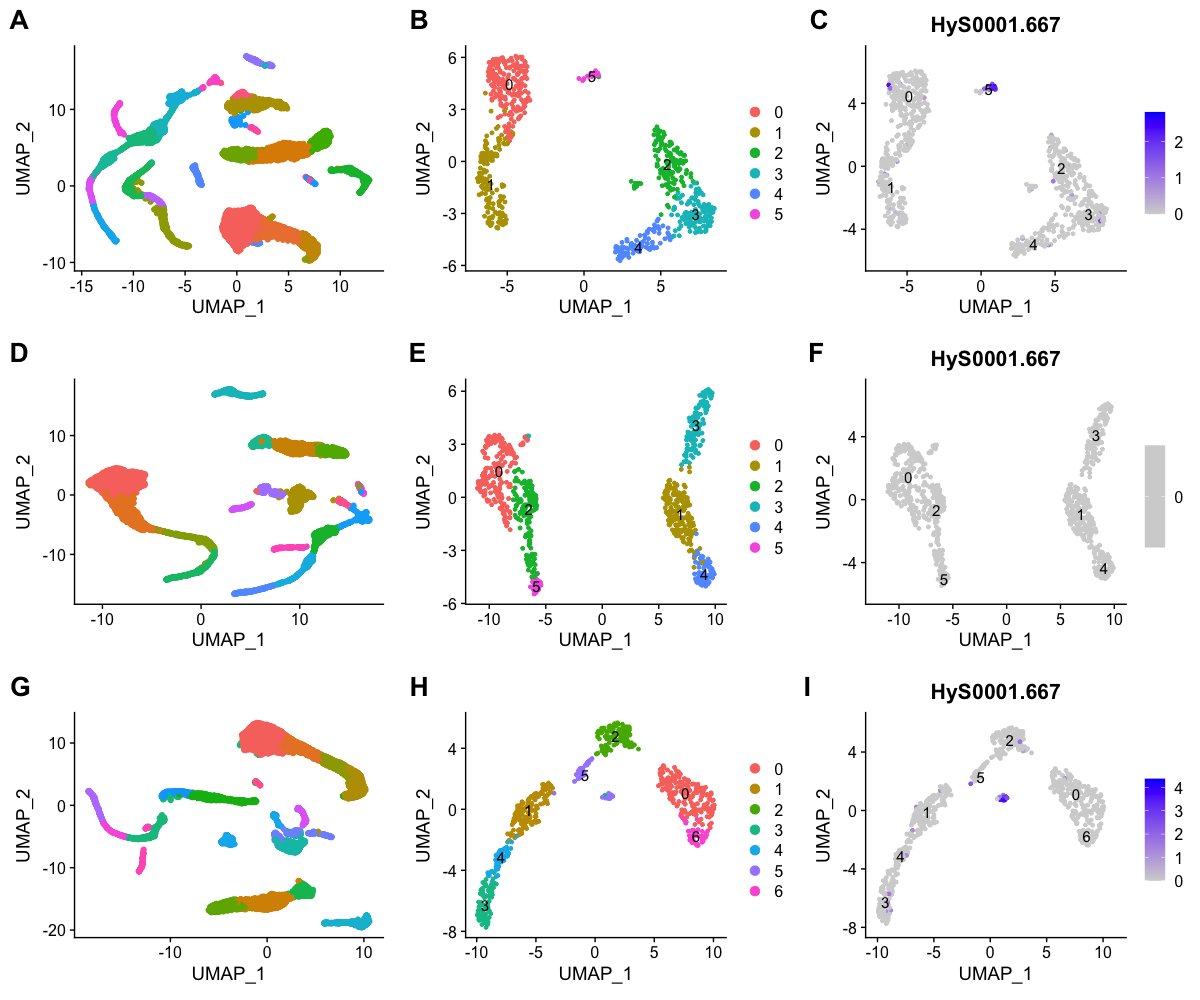


**Figure 6:** UMAP visualization of the different versions of our H. symbiolongicarpus single-cell atlas. (A-C) H. symbiolongicarpus single-cell atlas version 1.0. (D-F) H. symbiolongicarpus single-cell atlas version 3.0. (G-I) H. symbiolongicarpus single-cell atlas version 3.1. (A, D, G) Different versions of the H. symbiolongicarpus single-cell atlas with all clusters. (B, E, H) Different versions of the i-cell subcluster. (C, F, I) Expression of mature sperm cell marker HyS0001.667 in each i-cell subcluster derived from the different versions of the single-cell atlas.

Figure 6 shows three versions of our *H. symbiolongicarpus* single-cell atlas. All maps looked good, but we felt the most confident about *H. symbiolongicarpus* single-cell atlas version 3.0 after checking various subcluster analyses from different cell lineages. While there may still be some artifacts in this dataset, we felt like they did not significantly affect our downstream analyses and began to verify the clusters using molecular methods like *in situ* hybridization of top marker genes for different clusters to validate our cluster annotations.

Conclusions

Overall, we chose the mature sperm cell marker filter method over the total RNA feature filter method for our final atlas. While it removed a significant number of cells (1,349 cells), it was the most effective strategy to get rid of mature sperm cell doublets. The total RNA feature filter still had some of these doublets remaining based on our i-cell subcluster analysis shown above. Also, the final overall map for the total RNA feature filter method seemed to be less complex, likely due to removing real cells that had a high number of RNA features. The *H. symbiolongicarpus* cell type atlas V3.0 is the current version of our single-cell dataset and was used for all downstream analyses.

SA04 – Raw and Final Datasets

*July 14^th^, 2022*

The following is a list of our raw and final datasets used in this supplementary analysis. They are made available on the *Hydractinia* genome portal at [https://research.nhgri.nih.gov/hydractinia](https://research.nhgri.nih.gov/hydractinia/)/.

3XPBS_filtered_feature_bc_matrix (raw 10X single-cell chromium data)

Seawater_filtered_feature_bc_matrix (raw 10X single-cell chromium data)

aggr.RData (*H. symbiolongicarpus* single-cell atlas version 3.0 Seurat data object)

aggr_ic.RData (*H. symbiolongicarpus* single-cell atlas version 3.0 Seurat data object)

aggr_fltd.RData (*H. symbiolongicarpus* single-cell atlas version 3.0 Seurat data object)

aggr_cutoff.RData (*H. symbiolongicarpus* single-cell atlas version 3.0 Seurat data object)
