# Supplementary material for: The genome of the colonial hydroid Hydractinia reveals that their stem cells use a toolkit of evolutionarily shared genes with all animals
Source: Genome Res. 2024 Mar;34(3):498–513. doi: 10.1101/gr.278382.123 (PMC11067881; doi:10.1101/gr.278382.123)
Supplement: Supplement 11 [file Supplemental_Material.pdf]

Supplemental Materials

The genome of the colonial hydroid *Hydractinia* reveals their stem cells utilize a toolkit of evolutionarily shared genes with all animals

Christine E. Schnitzler*, E. Sally Chang, Justin Waletich, Gonzalo Quiroga-Artigas, Wai Yee Wong, Anh-Dao Nguyen, Sofia N. Barreira, Liam B. Doonan, Paul Gonzalez, Sergey Koren, James M. Gahan, Steven M. Sanders, Brian Bradshaw, Timothy Q. DuBuc, Febrimarsa, Danielle de Jong, Eric P. Nawrocki, Alexandra Larson, Samantha Klasfeld, Sebastian G. Gornik, R. Travis Moreland, Tyra G. Wolfsberg, Adam M. Phillippy, James C. Mullikin, Oleg Simakov, Paulyn Cartwright, Matthew Nicotra, Uri Frank, Andreas D. Baxevanis*

*Corresponding authors E-mail: christine.schnitzler@whitney.ufl.edu (C.E.S.); andy.baxevanis@nih.gov (A.D.B.)

Table of Contents:

Supplemental Materials and Methods

Supplemental Figures S1 to S32

Supplemental Tables S1 to S31 (with exceptions listed below)

Legends for Additional Supplemental Tables (S9, S11, S12, S18, S27, S29, S30, S31)

Legends for Supplemental Data S1 to S49 and Supplemental Code S1-S12

References

Supplemental Materials and Methods

Hydractinia strains and animal care

All *Hydractinia* strains were cultured using standard methods (Frank et al. 2020). Generally, colonies were grown on glass microscope slides and cultured in 38-L tanks filled with artificial seawater (Instant Ocean-Reef Crystals or Red Sea-CoralPro Salt) at 29-32 ppt and kept at 18–22°C under a light/dark regime (e.g. 10 h/14 h). Animals were fed three to five times a week with 3- or 4-day-old *Artemia* nauplii cultured at 25°C (Premium Grade, Brine Shrimp Direct). Some animals were supplemented twice a week with an oyster puree made from freshly caught, shucked, and blended oysters (stored at −20°C). The *H. symbiolongicarpus* 291-10 strain was bred in 2014 in the Nicotra laboratory by crossing two outbred colonies collected from New Haven Harbor, Connecticut by Leo Buss. The *H. echinata* F4 strain was originally collected from Galway Bay, Ireland.

Estimation of genome size

Genome size estimates were generated using the method of propidium iodide staining of isolated nuclei followed by flow cytometry for four hydrozoan species, including *Hydractinia symbiolongicarpus* male strain 291-10, *Hydractinia echinata* female strain F4, *Podocoryna carnea* male strain PcLH01, and *Hydra vulgaris* strain 105. (The *H. vulgaris* samples were kindly provided by Rob Steele.) Frozen polyps from each species were shipped to J. Spencer Johnston of Texas A&M University for analysis. Methods for nuclei isolation follow those described by Hare and Johnston (Hare and Johnston 2011) with the following modifications. Material was chopped with a fresh razor blade (50 chops), then ground very gently using 3-5 slow strokes with a B-size pestle in a Dounce tissue grinder. The nuclei of the sample and the DNA size standard (heads of *Drosophila melanogaster*) were released together by chopping and grinding and were run through a 20 µm nylon filter to remove debris, then stained at least 30 minutes in the cold and dark with 20 mg/ml propidium iodide. The relative red fluorescence of the 2C nuclei from the standard and sample were scored with a Partec Cyflo flow cytometer equipped with a laser emitting exciting light at 532 nm and the red fluorescence was scored after passing a long pass filter that blocked light below 590 nm. DNA content was determined as the ratio of the fluorescence of the sample (expressed as a

channel number) to that of the standard multiplied by the 1C amount of DNA in the standard (1C = 175). Supplemental Table S1 shows resulting genome size estimates for each species. The estimate for *H. vulgaris* was similar to other published estimates (Zacharias et al. 2004; Chapman et al. 2010).

Genomic DNA extraction, whole-genome sequencing, and genome asembly pipeline, including polishing

A combination of PacBio long-read and Illumina short-read genomic sequence data was generated for *Hydractinia symbiolonigcarpus* strain 291-10 and *Hydractinia echinata* strain F4. Slightly different protocols were used for extracting high molecular weight genomic DNA for each species.

For *Hydractinia symbiolongicarpus* 291-10, several animals were snap-frozen in a mortar filled with liquid nitrogen and the frozen tissue was ground into a fine powder. Additional liquid nitrogen was added along with 6 ml UEB1 extraction buffer (7M urea, 300 mM NaCl, 50 mM Tris pH 8, 20 mM EDTA, 1% SDS), which froze. The tissue/UEB1 mixture was ground into a fine powder, then allowed to thaw and transferred as liquid slurry into a 50 ml tube. The volume was brought to 25 ml with additional UEB1 buffer and slurry mixed by gentle inversion. Total nucleic acids were extracted twice with 1 volume phenol:chloroform, then precipitated with 1/10 volume sodium acetate (pH 5.2) + 0.7 volumes isopropyl alcohol (IPA), and pelleted by spinning at 3700 x g for 20 minutes. The pellet was washed twice with 70% EtOH, resuspended in 1X TE, and allowed to incubate overnight at 4°C. The following morning, RNA removal was performed by adding 1 ul RNAse cocktail (Ambion catalog #AM2286) per 100 ul sample and incubating at 37°C for 20 min. The DNA was then extracted twice with 1 volume phenol:chloroform, then precipitated with 1/10 volume sodium acetate (pH 5.2) + 0.7 volumes IPA, and pelleted by centrifugation at 3700 x g for 20 minutes. The DNA was then washed with 70% EtOH and resuspended in 1X TE.

For *Hydractinia echinata* F4, approximately 100 adult polyps per extraction were lysed in 1 ml extraction buffer (100mM TrisCl pH8, 1% SDS, 50mM EDTA). 2ul each RnaseA and RnaseT1 were added and then samples were incubated for 1 hour at 37°C. 2ul Proteinase K (25mg/ml stock) was then added along with SDS to a final

concentration of 1% and NaCl to a final concentration of 0.5M and the solution was incubated at 55°C for 1 hour. The DNA was then extracted using 1 volume phenol:chloroform and precipitated by the addition of 1/10 volume of 5M NaCl and two volumes of ethanol. The precipitated DNA was spooled on a pipette tip and transferred to a new tube and washed two times with 70% ethanol before being resuspended in nuclease-free water.

Both methods resulted in intact high molecular weight gDNA as visualized on a pulsed field gel with a high molecular weight marker. Bands ran slightly larger than the top 48.5 kb band of the GeneRuler High Range DNA ladder. For *H. echinata*, each extraction of about 100 polyps resulted in 7.5-14.7 µg gDNA. For *H. echinata*, a total of 5 PacBio libraries were constructed, which were sequenced over 83 SMRT cells run with P6-C4 chemistry (estimated 84X genomic coverage). For *H. symbiolongicarpus*, a total of 3 PacBio libraries were constructed, which were sequenced over 80 SMRT cells run with P6-C4 chemistry (estimated 94X genomic coverage). The estimated average insert size for the PacBio libraries and number of SMRT cells sequenced per library are shown in Supplemental Table S2.

Genomic DNA from the same extraction that was used for constructing the PacBio libraries was also used to construct PCR-free paired end dual index Illumina libraries that were sequenced on an Illumina HiSeq2500, run as 250 base paired-end reads. One Illumina library was constructed and sequenced for each *Hydractinia* species. These sequences were used mainly in the assembly polishing step after genome assembly was complete (see Genome Assembly Pipeline, below) and they were used to estimate heterozygosity for both species using the Jellyfish k-mer counting program (Marçais and Kingsford 2011) followed by GenomeScope 2.0 (Ranallo-Benavidez et al. 2020). The GenomeScope profiles gave an estimated heterozygosity of 1.33% for *H. symbiolongicarpus* and 0.85% for *H. echinata* at a k-mer of 31 (Supplemental Figure S1).

Genome assembly pipeline

PacBio filtered subreads were generated with the PacBio SMRTportal subread filtering protocol using default parameters. This process generated a single subread fastq file for each PacBio library sequenced. These filtered

subreads were used as input to our genome assembly pipeline. Canu (Koren et al. 2017) and Falcon_unzip (Chin et al. 2016) assemblers were each used to independently assemble the PacBio sequence data.

Canu assemblies were carried out using Canu v1.3 (https://github.com/marbl/canu) with default parameters. The program attempted to separate out contigs representing alternative haplotypes into primary and secondary assemblies via a filtering step. Due to the relatively high level of heterozygosity in both genomes, this filtering was not entirely successful, and the initial primary assemblies were larger than the expected haploid genome size with some contigs still representing duplicated loci from alternative alleles. The total assembly size for *H. symbiolongicarpus* was 731.169 Mbp. The total assembly size for *H. echinata* was 923.608 Mbp. The presence of duplicated loci in the initial primary assemblies was confirmed with BUSCO (Simão et al., 2015) v1.22, which indicated 42% and 29% duplicated genes in the *H. symbiolongicarpus* and *H. echinata* assemblies, respectively. To remove much of the duplication and attempt to better separate haplotypes, self-alignments of all contigs with >1 read was performed with MUMmer 3.23 (Kurtz et al. 2004) with the command "nucmer –maxmatch –l 100 –c 1000 asm.ctg.fasta asm.ctg.fasta". The number of matches > 5 kbp and 90% identity between all pairs of contigs was calculated and contig pairs were sorted by the number of matches. The contigs were greedily assigned to "primary" and "secondary" assemblies starting with the pair with the highest number of matches. Contigs with no alignments were then added to the secondary set. This generated a primary set of 395.756 Mbp and a secondary set of 335.412 Mbp for *H. symbiolongicarpus*. For *H. echinata* the primary set was 547.486 Mbp and the secondary set was 376.122 Mbp. Following this filtering procedure, the presence of duplicated loci in the primary set according to BUSCO was reduced to 11% (*H. symbiolongicarpus*) and 10% (*H. echinata*). Secondary contig assemblies represent the second of the two allelic copies of the heterozygous regions of the diploid genome as well as contigs that had no self-match in the MUMmer filtering step. These secondary assemblies were not scaffolded.

Falcon_unzip assemblies (Chin et al. 2016) (https://github.com/PacificBiosciences/FALCON_unzip) were carried out for both *Hydractinia* species. This generated a set of primary contigs and a set of "haplotigs" that are

equivalent to the “secondary” assemblies from Canu. Following the assembly step, quiver polishing (Chin et al. 2013) was run on both sets of contigs from both species.

Scaffolding was done by Dovetail HiRise scaffolding with Illumina Chicago libraries constructed from the same gDNA extracted for PacBio and Illumina sequencing described above. The primary sets of contigs from Canu and Falcon_unzip were sent to Dovetail for each species. For *H. symbiolongicarpus*, there were 5,591 input contigs from the primary Canu set. After Dovetail scaffolding, there were 4,611 scaffolds. For *H. echinata*, there were 8,112 input contigs from the primary Canu set. After Dovetail scaffolding, there were 7,095 scaffolds. For *H. symbiolongicarpus*, there were 2,719 input Falcon_unzip contigs and, after Dovetail scaffolding, there were 2,081 scaffolds. For *H. echinata* there were 1,701 input Falcon_unzip contigs and, after Dovetail scaffolding, there were 2,361 scaffolds.

To compare the two scaffolded assemblies, we mapped the Falcon_unzip assemblies to the Canu assemblies to check if they both had similar sequences in their primary sets using MUMmer v 3.23 (Kurtz et al. 2004) with the following settings: ./nucmer -mumref -l 50 -c 500 ./canu_assembly ./falcon_assembly. Then we ran dnadiff -d to generate a report file which reports the percentage of each assembly aligned to each other. We found that 14% of the primary set from Falcon_unzip was not present in the Canu assembly for *H. symbiolongicarpus* and 7% of the primary set from Falcon_unzip was not present in the Canu assembly for *H. echinata*. These results likely mean that there are repeats that were not aligned between the two genomes, so we did not pursue these differences between the assemblies further. After comparing the Canu and Falcon_unzip Dovetail-scaffolded assemblies, based on overall genome statistics and BUSCOv5 statistics using the Metazoa dataset (Supplemental Tables S3 and S4), Canu outperformed Falcon_unzip on all metrics so we decided to move forward with the Canu assemblies and abandon the Falcon_unzip assemblies.

Gap filling and polishing steps

PBJelly software (https://sourceforge.net/p/pb-jelly/wiki/Home/) from the PBSuite was used for gap filling the Canu-Dovetail assemblies using the PacBio reads. The program was run with gapInfo.bed files provided by Dovetail and the following parameters: -i --minGap=3. After the gap filling step, the assemblies had remarkably low percentages of remaining gaps: the *H. symbiolongicarpus* assembly had 0.007% gaps and the *H. echinata* assembly had 0.005% gaps.

The ArrowGrid parallel wrapper (https://github.com/skoren/ArrowGrid) was used for running the Arrow consensus framework (http://github.com/PacificBiosciences/GenomicConsensus/) within the PacBio SMRT Analysis Software to polish the gap-filled assemblies using the PacBio reads. Details on the original consensus model used for polishing can be found in Chin et al. (Chin et al. 2013). Following Arrow polishing, the PilonGrid parallel wrapper (https://github.com/skoren/PilonGrid) was used for running Pilon polishing (Walker et al. 2014) using the Illumina 2x250 genomic reads. After these steps, the *H. symbiolongicarpus* assembly had 4,509 scaffolds and the *H. echinata* assembly had 6,983 scaffolds.

Following the gap filling and polishing steps, we sought to determine whether all transcripts in our independently generated transcriptomes (see Transcriptome Sequencing, below) were represented in our primary assemblies or whether some sequences that are not represented in the primary assemblies had been filtered into the secondary assemblies when we were separating haplotypes. All transcripts from the transcriptomes were aligned to the primary and secondary sets using a BLAST approach based on the 'alien_index' and 'no_transcript_left_behind' perl scripts (https://github.com/josephryan/alien_index, https://github.com/josephryan/no_transcript_left_behind) using the following formula: AI = log((best E-value for primary) + 1x 10^{-200}) - log((best E-value for secondary) + 1 x 10^{-200}). Genomic sequence for any transcript that had a significant alignment to the secondary set but was missing from the primary set entirely was added back to the primary set, making the final size of the primary set for *H. symbiolongicarpus* 406.693 Mbp and 565.066 Mbp for *H. echinata* (Supplemental Table S3). We only added partial scaffolds that included only the genomic region of each transcript to the primary set to avoid

increasing the amount of duplicated sequence. However, we kept the complete scaffolds in the secondary set, including the partial scaffold sequence that we added to the primary set, so there is some redundancy between the primary and secondary sets for these sequences. This means the larger genomic context of those sequences that were added back can be found in the secondary set. For *H. symbiolongicarpus*, 331 sequences were added to the primary set, each as a separate scaffold. For *H. echinata*, 784 sequences were added to the primary set, each as a separate scaffold. We are aware that the primary assemblies are meant to be haploid representations of the genome and that adding these sequences back may result in having both allelic copies of some genes (especially in genomic regions with high heterozygosity) represented in the primary assembly. We felt that the small number of genes that were added back would not greatly inflate allelic duplications and that the benefit of adding some genes that were completely missing from the primary assemblies outweighed this potential cost of increasing false allelic duplication. BUSCO v5 statistics (using the Metazoa dataset) on the assemblies before and after adding sequences illustrate these points as the number of duplicated BUSCO genes does increase after adding the sequences but, at the same time, the number of completely missing BUSCO genes decreases:

H. symbiolongicarpus BUSCO before adding 331 sequences: C:88.9%[S:83.8%,D:5.1%],F:4.7%,M:6.4%

H. symbiolongicarpus BUSCO after adding 331 sequences: C:89.6%[S:83.8%,D:5.8%],F:4.6%,M:5.8%

H. echinata BUSCO before adding 784 sequences: C:89.1%[S:77.6%,D:11.5%],F:5.0%,M:5.9%

H. echinata BUSCO after adding 784 sequences: C:89.1%[S:75.8%,D:13.3%],F:5.2%,M:5.7%

The final genome assembly statistics, including BUSCO v5 statistics on the assemblies and on the gene models after this step, are shown in Supplemental Table S3.

Adult Transcriptome Sequencing and Assembly

Total RNA was isolated from the following tissues: *H. symbiolongicarpus* – gastrozooids and gonozooids dissected from an adult colony (strain 291-10, one sample); *H. echinata* – gastrozooids, gonozooids, and stolonal tissue from an adult colony (strain F4, three samples). RNA was extracted using standard Trizol/chloroform

extraction methods and cleaned with a RNeasy Mini Kit (Catalog #74104; Qiagen) according to the manufacturer's protocol. RNA samples were shipped to the NIH Sequencing Center on dry ice for strand-specific library construction and sequencing. The quality of each sample was checked with a BioAnalyzer or TapeStation prior to library construction. RNA-Seq libraries were constructed from 1 microgram RNA using the Illumina TruSeq Stranded mRNA kit. The resulting cDNA was fragmented using a Covaris E210 focused ultrasonicator. Library amplification was performed using 10 cycles to minimize the risk of over-amplification. Unique barcode adapters were applied to each library. Samples were sequenced on an Illumina HiSeq 4000 as paired end 75 bp reads (*H. symbiolongicarpus*) or run on an Illumina HiSeq 2500 as paired end 125 bp reads (*H. echinata*). RNAseq reads for each species are deposited in the SRA under PRJNA807936 (*H. symbiolongicarpus*) and BioProject PRJNA812777 (*H. echinata*).

These reads were assembled in multiple ways using the Trinity (Grabherr et al. 2011; Haas et al. 2013), TopHat/stringtie, and HISAT2/stringtie (Pertea et al. 2015) assemblers to attempt to recover the most comprehensive set of transcripts. Supplemental Data S1 details how all assemblies were generated for each species. Supplemental Tables S6 and S7 show statistics for each transcriptome generated for each species. The best Trinity transcriptome for each species according to N50 length statistics plus BUSCO v5 statistics is available as a download on the *Hydractinia* genome project portal website: https://research.nhgri.nih.gov/hydractinia/download/index.cgi?dl=tr

Generation of low-redundancy stranded transcriptomes

These alternate transcriptomes were subsequently processed and merged using script EvidentialGene tr2aacds.pl (v2017.12.21) to generate a final low-redundancy transcriptome for each species. The final *H. symbiolongicarpus* assembly was generated from merging twelve assemblies and includes 39,802 transcripts with a minimum sequence length of 200bp. The final *H. echinata* assembly was generated from merging five assemblies and includes 90,302 transcripts with a minimum sequence length of 200bp. Both merged assemblies are available as

downloads on the *Hydractinia* Genome Project Portal web site:

https://research.nhgri.nih.gov/hydractinia/download/index.cgi?dl=tr

Gene model prediction

Gene models were generated with a pipeline that involved both PASA (Haas et al. 2003) and Augustus (Stanke and Waack 2003). Strand-specific RNAseq data from each species (detailed in Transcriptome Sequencing and Assembly) was used as input at different points of the pipeline as reads and as assembled transcripts. To generate assembled transcripts, the first 12 nucleotides of the RNAseq data were trimmed using Trimmomatic v0.36 (Bolger et al. 2014) with the flag HEADCROP:12. Reads were error corrected with perl script ErrorCorrectReads.pl (ALLPATHS-LG release 48894 (Gnerre et al. 2011). The Trinity assembly pipeline (version 2.1.1) (Grabherr et al. 2011; Haas et al. 2013) was then run on the trimmed, error-corrected reads with default parameters. The resulting Trinity assemblies were used as input to PASA (version 2.2.0) to generate *ab initio* gene predictor training sets. These were then input into Augustus (version 3.2.2) following the multi-step "Incorporating Illumina RNAseq into AUGUSTUS with GSNAP" pipeline specified here: http://bioinf.uni-greifswald.de/bioinf/wiki/pmwiki.php?n=IncorporatingRNAseq.GSNAP.

This pipeline involved: (1) aligning RNAseq reads with GSNAP (Wu et al. 2016) and using the output to generate intron hints, (2) creating repeats hints with RepeatMasker, and (3) using the output of PASA as a training set. We also used the output of BLAT-aligned (Kent 2002) RNAseq reads as exon hints. After running Augustus with these inputs, we ran PASA again to add/update predicted UTRs to the Augustus gene models. A step-by-step description of the specific pipeline we used, including inputs and relevant code, is provided in File S2. The final number of predicted genes for *H. symbiolongicarpus* is 22,022. The final number of predicted genes for *H. echinata* is 28,825. Summary statistics for gene models are provided in Supplemental Table S5 and were generated with the custom perl script generate_stats.pl (Supplemental Code S1).

Evaluating completeness of predicted gene models

Completeness of the predicted gene models was evaluated via BUSCO v5 with the Metazoa dataset. *H. symbiolongicarpus* gave the following BUSCO percentages: 93.8% gene models were complete or fragmented, with 10.2% duplicated (C:92.5%[S:82.3%,D:10.2%],F:1.3%,M:6.2%,n:954). *H. echinata* gave BUSCO results of 93.4% gene models complete or fragmented, with 12.3% duplicated (C:90.7%[S:78.4%,D:12.3%],F:2.7%,M:6.6%,n:954).

We then determined the percentage of gene models that had assembled transcript support (and the level of overlap for each gene) and functional annotation. For our transcript support analysis, we combined the RNA-seq data from adult animals (detailed in 'Adult Transcriptome Sequencing and Assembly' of this supplemental methods**)** with additional RNA-seq data from four developmental stages for *H. symbiolongicarpus* (4-cell, 16-cell, 64-cell, 24 h larva, detailed below in 'Developmental Time series RNAseq') or polyp head regeneration timepoints for *H. echinata* (detailed in 'Genes involved in polyp head regeneration' of this supplemental methods). RNAseq reads from each dataset were independently aligned to either the *H. symbiolongicarpus* or the *H. echinata* genome using HISAT2. StringTie was run on the HISAT2 output to assemble the aligned reads into transcripts. StringTie in merge mode (--merge) was then run for all datasets from each species to generate a global, unified set of transcripts from the multiple RNA-Seq samples in each dataset. We then used bedtools intersect to generate a .bed file with overlap between the transcripts and the gene models. The intersection length for each gene model that had overlapping transcripts was calculated with a custom perl script 'overlaptranscripts.pl' (Supplemental Code S1). Another custom script 'calculate_overlap.pl' (Supplemental Code S1) was run to calculate the % transcript overlap for each gene in terms of length of the gene for each dataset. To process multiple overlap files from the different transcript datasets, we used the custom script 'calculate_multiple_overlap.pl' (Supplemental Code S1). Finally, from this output we determined the total percent of genes with different levels of overlapping transcripts (>99%, >90%, >50%, and <10% overlap). We summarized these results for each individual transcript dataset and for the combined transcript dataset for each species. Results are displayed for all genes and for genes that were

unassigned in our OrthoFinder analysis in Supplemental Table S8. Overall, most genes either had full (>99%) transcript support (75.72% *H. symbiolongicarpus* genes; 57.83% *H. echinata* genes) or no (<10%) transcript support (15% *H. symbiolongicarpus* genes; 23% *H. echinata* genes), which can be best visualized with a histogram (Supplemental Figures S2 and S4). Results specific to unassigned genes are discussed further in the *Identity of Genes Not Assigned to Orthogroups* section of this document (see below).

Developmental time series RNAseq

Total RNA was extracted from embryos at 2-4 cell, 16-32 cell, and 64-128 cell stages, and from 24 hours post fertilization larvae, using TRIzol solution (ThermoScientific #15596026) followed by binding RNA on a column (EpochLifeScience #1940) and on-column DNA digestion (Qiagen #79254). RNA was then eluted with nuclease free water, assessed with a Qubit RNA HS assay, and electrophoresed along with RNA loading dyes (ThermoScientific #R0641) in a denaturing formaldehyde agarose gel for visualization. RNA samples were shipped on dry ice to the NIH Intramural Sequencing Center (NISC) and quality checked with an Agilent 2100 Bioanalyzer prior to library construction. RNA-Seq libraries were constructed from 1 ug RNA (RIN >9.5) using the Illumina TruSeq RNA Sample Prep Kit, version 2. The resulting cDNA was fragmented using a Covaris E210 focused ultrasonicator. Library amplification was performed using 10 cycles to minimize the risk of over-amplification. Unique barcode adapters were applied to each library. Libraries were pooled in an equimolar ratio and sequenced together on three lanes of an Illumina HiSeq4000. At least 65 million paired end 75-base reads were generated for each individual library. Raw reads were subjected to quality control using FastQC v0.11.5. Overrepresented sequences and low-quality (<32) bases were trimmed using Trimmomatic v0.30. After trimming, unpaired reads and reads shorter than 25 bp were discarded.

HISAT2 v2.1.0 (Kim et al. 2015) was used to align reads to the *H. symbiolongicarpus* gene models. Alignment rates ranged from 92.13-94.55%. Alignments were sorted using ‘samtools sort’ v 1.10 and read count matrices were generated with htseq-count v0.11.2 (Anders et al. 2015) followed by generating normalized counts with the ‘MedianNorm’ method in EBSeqHMM (Leng and Kendziorski 2022). Developmental time series expression plots

for each gene, generated with the boxplot command in R, are displayed on *H. symbiolongicarpus* Gene Pages and can be searched on our Developmental Expression page (https://research.nhgri.nih.gov/hydractinia/developmental_expression/) with a gene id. RNAseq reads for the *H. symbiolongicarpus* developmental time series are deposited in the SRA under PRJNA807936.

Functional annotation

Functional annotation of gene models for the analyses described in this paper were performed with several programs including a DIAMOND search (Buchfink et al. 2015) using the default DIAMOND e-value cutoff of 0.001 of NCBI's nr database, and by running PANNZER2 (Koskinen et al. 2015; Törönen et al. 2018). A combined annotation file with these results can be found in Supplemental Table S9. We determined the number of gene models with either a DIAMOND hit or a PANNZER2 hit, or both, for each species.

For the *Hydractinia* Genome Portal Gene pages (https://research.nhgri.nih.gov/hydractinia/genewiki/), BLASTp searches against UniProt and against the NCBI nonredundant protein (nr) database were done for all *Hydractinia* predicted proteins and the top four results from each database is displayed. InterProScan version 5.33-72.0 (Jones et al. 2014) was run with default parameters and was used to assign protein domains and motifs and provide additional annotation (e.g. Gene ontology (GO) terms and pathways) to all predicted *Hydractinia* proteins and this information is displayed on the *Hydractinia* Genome Portal Gene pages. Pfam-A domains were predicted by running HMMscan from the HMMER suite version 3.1b1 (hmmer.org) using an E-value cutoff of 1×10^{-6} for all predicted *Hydractinia* proteins. All identified domains are displayed on the *Hydractinia* Genome Portal Gene pages. We also provide a Pfam Search page to identify all predicted *Hydractinia* proteins with selected domains (https://research.nhgri.nih.gov/hydractinia/pfam/).

For the *Hydractinia* Genome Portal Gene pages, GO term annotation of all *Hydractinia* proteins was done with the Argot$^{2.5}$ webserver (Falda et al. 2012; Lavezzo et al. 2016). Additional GO term annotation was performed

with PANNZER2 (Koskinen et al. 2015; Törönen et al. 2018). In this case, the program was run with the flag --PANZ_FILTER_PERMISSIVE to obtain the maximum number of annotations. Resulting gene descriptions, gene names, and GO terms are available on the *Hydractinia* gene pages. GO term annotation from Argot[2.5] and PANNZER2, as well as the combined annotation file (Supplemental Table S9) for each species are available as downloads on the *Hydractinia* Genome Portal (https://research.nhgri.nih.gov/hydractinia/download/index.cgi?dl=fa).

Mitochondrial genome

The mitochondrial genomes of *Hydractinia symbiolongicarpus* and *Hydractinia echinata* were identified by aligning the mtDNA chromosome of *Hydra oligactis* (NC_010214.1) against the genome assemblies of the two *Hydractinia* species using BLAST (command-line BLAST+ using default parameters). As observed in other hydrozoans, *H. symbiolongicarpus* and *H. echinata* possess a single linear mitochondrial chromosome (scaffolds HyS0613 and HyE5562 of our assembly, respectively), similar to what has been shown for other hydrozoan genomes (Supplemental Figure S7).

We identified protein-coding and ribosomal sequences in the mitochondrial scaffolds by aligning the sequences of the *H. symbiolongicarpus* mtDNA genes (Supplemental Table S10) and the 16S RNA (RNL) and 12S RNA (RNS) genes of *H. oligactis*, extracted from its mitochondrial chromosome (NCBI accession NC_010214.1), using BLAST (command-line BLAST+ using default parameters) (Altschul et al. 1990). The integrity of the open reading frames of all protein-coding genes was confirmed via translation using the minimally derived genetic code (translation table 4).

Transfer RNA (tRNA) genes were identified with ARWEN (using the invertebrate mitochondrial genetic code, not reporting low scoring tRNAs, and setting a linear topology), as well as with tRNAscan-SE (using the organelle module option) (Laslett and Canbäck 2008; Lowe and Eddy 1997). Only tRNAs identified by both programs were reported (Supplemental Figure S8).

We determined the origin of replication by analyzing intergenic spacer sequences using a combination of results from UNAFold and graphical representations of nucleotide distributions generated using the DNA Walker feature within GraphDNA. This approach allows for the identification of abrupt changes in nucleotide composition bias and stem-loop configurations containing T-rich loops (Supplemental Figure S9) (Markham and Zuker 2008; Thomas et al. 2007).

The analysis of inverted terminal repeats (ITRs) was performed by extracting the non-coding sequence from the 5' end of the mtDNA scaffold, then aligning this non-coding sequence against the scaffold using BLAST (command-line BLAST+ using default parameters). The non-coding sequence from the 3' end of the mitochondrial scaffold was also aligned against the scaffold, and the overlap of these alignments was used to determine the consensus region for the ITR sequences. The presence and type of secondary structures within ITRs was determined with UNAFold (Supplemental Figure S10).

The gene content and chromosomal architecture of the two *Hydractinia* species was compared with those of *H. oligactis* (NC_010214.1), *H. vulgaris* (NC_011220.1 and NC_011221.1), and *Clytia hemisphaerica* (scaffold CACVBU010001317.1 from genome assembly GCA_902728285.1). The mtDNA scaffold of *C. hemisphaerica* was identified by performing a BLAST search against its genome assembly (GCA_902728285.1), using the partial sequence of its mitochondrial 16S ribosomal RNA gene (KX665279.1) as the query. The gene content of scaffold CACVBU010001317.1 was characterized using tRNAscan-SE and ARWEN (for tRNA genes), protein translation with ExPAsy Translate (Gasteiger et al. 2003) using the coelenterate mitochondrial code (for protein-coding genes), and alignment of *Hydractinia* 12S and 16S sequences (for ribosomal genes).

Analysis of NUMTs was carried out by aligning *Hydractinia* mitochondrial genes against the genome assemblies using BLAST (command-line BLAST+ with default parameters). Only mtDNA scaffolds were reported to contain mitochondrial sequences. This result was further confirmed by aligning raw Illumina reads against the

mitochondrial genomes of both *Hydractinia* species using BWA (Li and Durbin 2009) and then assessing sequence variance with the SAMtools package (Li and Durbin 2009).

Orthology inference, phylogenetic analyses, and divergence time estimates

Taxon Selection for Orthology Analysis

Orthology-inference analysis was performed on a proteome dataset of 49 species from 15 metazoan phyla and four non-metazoan outgroups. Taxon selection for this analysis was initially based on the data set used by Maxwell et al. (Maxwell et al. 2014) to infer the evolutionary origins of human disease-associated gene families, which we then expanded to place the *Hydractinia* genomes in an evolutionary context with other cnidarian genomes. To that end, 16 cnidarian species, spread across the main cnidarian lineages (Anthozoa, Scyphozoa, Hydrozoa, and Cubozoa) were also included. This represents the largest sampling of cnidarians in any genome-wide orthology inference performed to date and provides increased resolution for characterizing genome-scale evolutionary dynamics within the cnidarians. We also included widely used model organisms, such as *D. melanogaster* and *C. elegans*, as well as a diverse sampling of the phylum Chordata. Additionally, we included the proteomes of several highly regenerative organisms such as *H. miamia* (Gehrke et al. 2019) and *S. mediterranea* (Grohme et al. 2018) to facilitate future comparative analyses regarding the genomic bases of regenerative ability.

Where possible, we opted to use the versions of these proteomes that are publicly available through NCBI or Ensembl unless a much more recent version of a particular species' proteome was available through another source (i.e., via a link from a publication to a lab website). We used this approach since NCBI and Ensembl versions tend to have standardized formats that facilitate downstream analyses and are often accompanied by information about isoform content (see Splice Filtering section, below). To assess the effect of proteome quality on our conclusions about gene gain or loss (i.e., incompleteness that could lead to an incorrect conclusion regarding gene loss), we ran each input proteome through BUSCO version 4.0.2 (Seppey et al. 2019) using the

core eukaryotic gene data set (accessed February 12, 2020) to obtain a rough measure of their completeness. We also ran each input proteome through BUSCO version 5.0 using the metazoan gene data set (accessed February 17, 2021). A detailed description of our dataset can be found in Supplemental Table S11 tab SM1. All 49 proteomes were annotated using the standalone version of PANNZER2 (Törönen et al. 2018) with default parameters. The complete results of this annotation process can be found in Supplemental Table S12.

Curation of Input Species Tree

While OrthoFinder2 (Emms and Kelly 2019) can generate a species tree based on the ortholog groups it infers, we opted instead to provide an input species tree based on the most up-to-date knowledge of intra- and inter-phylum relationships as of this writing. Effectively, our input species tree (Supplemental Figure S11) was a manually curated supertree based on a number of recent publications on metazoan phylogenomics (Kayal et al. 2018; Laumer et al. 2019; Marlétaz et al. 2019). For several analyses, including the OrthoFinder2 inference, we conducted additional runs with an alternative species tree that had the sponge *Amphimedon queenslandica* as the earliest diverging metazoan lineage to test the effect of this branching order on our downstream analyses.

Splice Filtering the Data Set

The authors of OrthoFinder recommend that, when possible, it is best to use a version of the input proteome containing just a single representative protein for each gene (Emms and Kelly 2019). To select a single isoform per gene, we created lists of proteins that correspond to specific genes in each proteome and developed a script that uses these lists, along with the input proteomes themselves, to select the longest isoform per gene (Supplemental Code S1). When there were multiple isoforms having the same longest length, the script randomly selected one of these isoforms. The creation of the proteins-to-genes lists depended on the source of the input proteome. For proteomes from NCBI, we used the Protein Table associated with each genome assembly to create this file, as this maps protein products to Gene IDs. For many other proteomes, we relied on information from the protein headers themselves (i.e., xx.g1.t1 and xx.g1.t2 were two transcripts of gene1). We were able to filter 28 of our 49 species for splice variation given available splice data. Information about these proteomes before and after

splice filtering and what information was used to associate genes and proteins can be found in Supplemental Table S11 tab SM1. Final splice filtered proteomes can be found as downloads on the *Hydractinia* Genome Project Portal at https://research.nhgri.nih.gov/hydractinia/download/index.cgi?dl=sd.

Running OrthoFinder

Orthology assignment with our splice-filtered input and custom species tree was performed using OrthoFinder version 2.2.7 using DIAMOND (Buchfink et al. 2015) as the sequence similarity algorithm and with the following parameters: -t 56 -a 4 -S diamond. A custom species tree was provided using the '-s' flag. The topology of the tree (Supplemental Fig. S11) was based on a review of recent literature, mentioned above. The raw output of OrthoFinder can be found in Supplemental Data S3-S9. Of note, the topology of the input tree does not affect initial orthogroup assignments produced by this version of OrthoFinder, but it does affect downstream inferences of gene gain or loss in particular lineages such as those we carried out to post-process the OrthoFinder output and in the Estimating Evolutionary Dynamics section below and is employed in orthogroup inference in the most recent versions of OrthoFinder (versions 2.4 and later).

Our analysis identified 33,325 orthogroups across the 49 species in our dataset. These orthogroups contain 81.2% (841,525/1,036,563) of the proteins in the dataset. Orthogroup size ranges from two to 5,349 sequences (mean = 25.3 and median = 5.0). There are 501 orthogroups which contain at least one sequence from all 49 species and 3,163 orthogroups that are multi-sequence but only contain sequences from one species (i.e., represent species-specific orthogroups). These species-specific orthogroups represent 9.5% of all orthogroups but contain only 1.7% of the input sequences. For full details describing our OrthoFinder run, see Supplemental Table S11 tabs X.1 and X.2.

Proteome Annotation

All 49 proteomes were annotated using the standalone version of PANNZER2 (Törönen et al. 2018) with default parameters, as well as with a DIAMOND (Buchfink et al. 2015) similarity search against nr with the option 'blastp'.

Identification of OMIM Gene Orthologs

To produce the tables found in the Online Mendelian Inheritance in Man (OMIM) subsection of each *Hydractinia* GeneWiki page (https://research.nhgri.nih.gov/hydractinia/genewiki/), we assessed which *Hydractinia* gene models represent orthologs of genes in the OMIM database (Hamosh et al. 2005) using both reciprocal BLAST and our OrthoFinder results. First, we downloaded a list of OMIM genes (accessed February 13, 2020), and subsetted this list to only those which have a Type 3 relationship with a disease phenotype, meaning that the phenotype has been linked to a specific mutation in that gene. For those genes, we extracted the proteins associated with these genes from the full human proteome dataset (see Supplemental Table S11 tab SM1 for version information). We then filtered this OMIM-associated subset of the proteome to the single longest isoform per gene as described in the Splice Filtering section, above, resulting in a data set of 4178 protein sequences. NCBI sequence IDs and other information about the final set of OMIM-associated genes can be found in Supplemental Table S11 tab SM2.

We carried out reciprocal BLASTP searches of this splice-filtered OMIM data set versus all gene models from each *Hydractinia* species using an E-value cutoff of 1×10^{-3} and without limiting the number of hits returned. We then post-processed the raw BLAST output in R to identify the reciprocal best BLAST hits (RBBHs) from these results. Where present, reciprocal best BLAST hits are marked by 'Recip-Blast-Hit' in the 'Evidence' column of the OMIM table on the GeneWiki pages. By analyzing the output from our OrthoFinder run, we also identified which *H. symbiolongicarpus* and *H. echinata* gene models were present in orthogroups found to contain an OMIM protein. All OMIM-associated protein sequences found in the same OrthoFinder orthogroup as a given *Hydractinia* sp. gene model are listed on each GeneWiki page marked with 'Orthogroup' in the 'Evidence'

column of the OMIM table. OMIM RBBHs and potential orthologs of each *Hydractinia* sequence can be found in Supplemental Table S11 tabs X.6-X.7 as well as on our GeneWiki pages on the *Hydractinia* Genome Project Portal.

Considering orthologous relationships inferred via both BLAST and OrthoFinder combined, approximately 25% (5,560 of 22,022 proteins) of the *H. symbiolongicarpus* proteome and 21% (6,321 of 28,825 proteins) of the *H. echinata* proteome have orthologous relationships with at least one of the 4,178 proteins in our splice-filtered data set of human-disease-associated proteins from the Online Mendelian in Man (OMIM) database. This difference in percentage is in line with *H. echinata*'s larger proteome and evidence that it has more species-specific proteins than *H. symbiolongicarpus*. Of these potentially orthologous relationships between a *Hydractinia* sp. protein and one or more of the OMIM proteins, 41% of the *H. symbiolongicarpus* and 36% of the *H. echinata* proteins have reciprocal best BLAST hits (RBBHs) with an OMIM protein, while > 99% of the *H. symbiolongicarpus* and 100% of the *H. echinata* proteins are in the same orthogroup as an OMIM protein. Except for three *H. symbiolongicarpus* proteins, all relationships inferred by BLAST were also inferred by OrthoFinder.

In terms of OMIM-protein coverage, the vast majority of the 4,178 human-disease-associated proteins have orthologs with *Hydractinia*. Approximately 78% of the 4,178 OMIM-associated protein data set was inferred to have orthologs in each *Hydractinia* species. Of that percentage, nearly all those OMIM proteins (3,128) are shared by the two *Hydractinia* species. Only 56 and 58 of them have orthologs only in the proteome of *H. symbiolongicarpus* and *H. echinata*, respectively. In total, 3,242 of the 4178 OMIM proteins have orthologs in the proteome of at least one *Hydractinia* species, either inferred by BLAST, OrthoFinder, or both.

Creating a Data Set of Single-Copy Orthologs for Phylogeny Inference

As a first step for performing downstream analyses of genome-wide evolutionary dynamics, we estimated the divergence time between *H. echinata* and *H. symbiolongicarpus* and between other cnidarian lineages by inferring a time-calibrated maximum-likelihood phylogeny. To infer this phylogeny, we chose to use only single-copy

orthologs (SCOs) as inferred by OrthoFinder, only using orthogroups where each species of interest is represented by a single sequence to infer our phylogeny. Since none of the inferred single-copy orthologs were present in all 49 input proteomes, we focused on curating a data set that is broadly shared and present as single copies within the cnidarians.

First, we removed the parasitic cnidarian *K. iwatai* since it has a notoriously small genome and many missing genes (Chang et al. 2015), as its inclusion would greatly reduce the possible number of potential shared orthogroups amongst the cnidarians. We then selected orthogroups which are present as single copies in at least 12 of the 15 remaining cnidarians in our input data set. We then chose several bilaterian and non-bilaterian outgroup species that maximized the number of SCOs they possessed from the aforementioned set of orthogroups, employing a custom python script filter_filln_og.py (Supplemental Code S1) to filter out non-single-copy orthogroups in outgroup species. The script also checks each SCO file to determine whether all species of interest are present and to fill in any missing data for each species with Ns; the resulting output is a concatenated FASTA-formatted file labeled by the species ID. Sequences in this final set of SCOs were then aligned by MUSCLE (version 3.8.31) (Edgar 2004) using default parameters. Poorly aligned regions were then trimmed with TrimAl (version 1.4.1 with -gappyout option) (Capella-Gutiérrez et al. 2009). Our final data set that was subsequently used for ML tree inference consisted of 22 species from 216 orthogroups, resulting in an alignment of 50,457 nucleotides. This final alignment can be found in Supplemental Data S11.

Inferring Maximum Likelihood Phylogeny and Estimating Divergence Times

The topology of our maximum likelihood phylogenetic tree (Figure 1B) was inferred using IQ-Tree2 (Minh et al. 2020). The best substitution model (LG+F+R7) was automatically selected by ModelFinder (Kalyaanamoorthy et al. 2017) during our IQ-Tree analysis. Branch supports were calculated using the ultrafast bootstrap estimation with 1000 bootstrap replicates. Divergence date estimates (Fig. 1B) were calculated for major nodes on the tree using a Langley-Fitch approach together with the TN algorithm, using r8s version 1.8.1 (Sanderson 2003). We fixed the age of the common ancestor of cnidarians at 570 MYA and set the divergence time of Hydrozoa to a

minimum of 500 MYA as suggested from the fossil record (Cartwright and Collins 2007). The raw output from IQ-Tree and r8s can be found in Supplemental Data S12-S13.

The topology of the Cnidaria+Bilateria+Outgroup tree estimated from the final alignment of single-copy orthologs reflects our current knowledge of relationships within Cnidaria and amongst metazoan phyla (Kayal et al. 2018). Divergence times estimated using this tree (Fig. 1), provide different estimates for key nodes compared with the most recent estimate of divergence times within Cnidaria (Khalturin et al. 2019). In that study, ages were estimated using a much larger set of both non-cnidarian metazoans and a larger set of calibration points, but no minimum age was set for the most recent common ancestor (MRCA) of hydrozoans. In contrast, for this study, we used a fossil calibration point of 500 MYA for this node, as suggested by Cartwright and Collins (Cartwright and Collins 2007).

The Khalturin et al.(Khalturin et al. 2019) study estimates the age of the hydrozoan MRCA to be only 392 MYA, which is likely unrealistic given the presence of crown-group hydrozoans much earlier in the fossil record (Cartwright and Collins 2007). We estimated the age of Hydrozoa to be exactly at our minimum age cutoff for the clade (500 MYA), demonstrating the importance of setting this constraint to make divergence time estimates consistent with the fossil record. Similarly, our age estimates for the MRCAs of Anthozoa and Medusozoa are 496.6 MYA and 538.9 MYA, respectively, versus the 438.2 and 479 MYA reported in the Khalturin study (Khalturin et al. 2019).

Strikingly, although our estimated ages for clades within Cnidaria tend to be older than those reported in Khalturin et al. (Khalturin et al. 2019), we date the divergence time between the two species of *Hydractinia* to be just 19.16 MYA. This estimate is much shorter than the estimated divergence times between lineages of *Aurelia aurita*, as estimated by either this study (45.35 MYA) or in Khalturin et al. (Khalturin et al. 2019) (51-193 MYA, depending on the lineages compared); it is more comparable to the divergence between lineages of *Hydra vulgaris* (10-16 MYA) as estimated by Wong et al. (Wong et al. 2019). We also analyzed a tree with *A. queenslandica* as

the outgroup instead of *M. leidyi* to test the effect of the branching order of Ctenophora and Porifera on our estimates (Supplemental Figure S12). We found that this did not affect the estimations in any systematic way and produced divergence times extremely close to those for the ctenophore-outgroup tree at the within-Cnidaria nodes that we focus on in this work.

Synteny

We performed pairwise synteny analysis of *H. symbiolongicarpus* and *H. echinata*, and between each *Hydractinia* species and *C. hemisphaerica*, *H. vulgaris*, or *N. vectensis* by calculating the number of shared orthogroups for all pairwise scaffolds and clustering the resulting count matrix by hierarchical clustering. We further clustered the matrix by density-based spatial clustering and displayed the data in pairwise syntenic dot plots. To do this, first we calculated the number of gene copies of each orthogroup for each species using the perl scripts OrthoFinderToOrthogroup.pl (Supplemental Code S1) and prepMsynt.pl (Supplemental Code S1). This produced a file called species.msynt. The species.msynt is the input file for the custom R script plot_msynt.R (Supplemental Code S1). plot_msynt.R performs several major functions. First, it calculates the number of shared orthogroups for all pairwise scaffolds and clusters the resulting count matrix by hierarchical clustering (using the function 'hclust' with ward.D2 algorithm). Since the genome assemblies used in this comparative analysis are fragmented, we further clustered the matrix by density-based spatial clustering (using the R package dbscan (ver 1.1.5)) and the clusters were colored by the group label. To further examine and extract highly conserved clusters, we compared the genes in each cluster using the R script find_common_og.R (Supplemental Code S1). All R scripts were run on Rstudio (ver 1.2.1335) with R (ver 3.61). Synteny dot plots are shown in Figure 1C.

Repeat analysis

To annotate repeats, determine what percentage of the genome is repetitive, and to create masked genome assemblies, we ran a comprehensive pipeline to identify and mask repetitive sequences. First, known repeat sequences for *H. symbiolongicarpus* and *H. echinata* were predicted using RepeatMasker ver 4.0.7 (Chen, 2004) with the default Dfam database of transposable elements ("known" analysis). Next, *de novo* repeats were

predicted using RepeatMasker with *de novo* repeat libraries ("de novo" analysis). *De novo* repeat libraries were constructed using RepeatModeler (ver 1.0.10) (http://www.repeatmasker.org/RepeatModeler/), which included running RECON (ver 1.0.8), RepeatScout (ver 1.0.5) and rmblast (ver 2.6.0), with default parameters and the output was used to run the RepeatMasker "de novo" analysis. Details of how many bases were part of repetitive regions and the corresponding percentage of the genome that is in repeats is shown in Supplemental Table S13. Tables classifying the types of repetitive elements, how many bases were part of each region, and the corresponding percentage of the genome in each class of repeat are shown for each analysis in Supplemental Tables S14-S17. Additional output files from the repeats analysis are available as downloads on the *Hydractinia* Genome Project Portal at https://research.nhgri.nih.gov/hydractinia/download/index.cgi?dl=sd.

Example RepeatMasker command for "known" analysis of *H. symbiolongicarpus*:

```
RepeatMasker Hsym_primary_v1.0.fa -species metazoa -engine crossmatch -u -gff -x -gccalc -pa 60 -dir OUTPUT_PATH
```

Example RepeatModeler commands for constructing *de novo* repeat libraries for *H. symbiolongicarpus*:

```
BuildDatabase -name symbio.db -engine ncbi Hsym_primary_v1.0.fa
RepeatModeler -database symbio.db -pa 60
```

Example RepeatMasker command for "de novo" analysis of *H. symbiolongicarpus*:

```
RepeatMasker Hsym_primary_v1.0.fa -lib /OUTPUT_PATH_OF_REPEATMODELER/RM_3192 /consensi.fa.classified -engine crossmatch -gff -x -gccalc -pa 60 -dir OUTPUT_PATH
```

RepeatMasker for "de novo" analysis was rerun with the -a option to generate alignment files which were used in some downstream analyses.

Characterization of genomic repeats including transposable elements

Output from the RepeatMasker de novo analysis showing classes of TEs for each species is shown in Supplemental Tables S14 and S16. The stacked charts in Fig. 1D are based on this output from the "de novo" analysis only.

The Kimura substitution levels between the repeat consensus to its copies were calculated using a utility script: calcDivergenceFromAlign.pl that is bundled in RepeatMasker. The repeat landscape plots (Fig. 1E) were produced using the R script age_plot_out.R (Supplemental Code S1) and age_plot_divsum.R (Supplemental Code S1) using the divsum output from the RepeatMasker script calcDivergenceFromAlign.pl (https://github.com/rmhubley/RepeatMasker/blob/master/util/calcDivergenceFromAlign.pl).

Orthogroup lineage specificity and overall patterns

Output from our OrthoFinder run was processed using custom R scripts (Supplemental Data S13-S15) orthogroup_queries.Rmd, phylum_specific_and_unassigned.Rmd, orthogroup_annotation.Rmd to analyze patterns of presence and absence of orthogroups across taxa and characterize the taxon-specificity of each orthogroup. Taxon specificity and other related information for each *H. symbiolongicarpus* and *H. echinata* gene model can be found in Supplemental Table S11 tabs X.10 and X.11.

Details of our analysis of transcript support for genes unassigned to an orthogroup can be found in the 'Evaluating completeness of predicted gene models' section of this document. The summary of these results can be found in Supplemental Table S8.

Broad-Scale Patterns of Orthogroup Specificity and Overlap

Recent cnidarian genome sequencing projects (Khalturin et al. 2019; Gold et al. 2019; Leclère et al. 2019) have demonstrated the contribution of both taxon-restricted and shared ancestral gene families to cnidarian-specific cell-types such as those in the medusa. To further evaluate the contribution of such gene families to evolutionary

novelty in *Hydractinia* given the number of cnidarian genomes now available for comparison, we first identified taxon-specific subsets of orthogroups. Of the 20,192 orthogroups inferred to be present in at least one cnidarian species by OrthoFinder (Emms and Kelly 2019), roughly 43% (8,746) are cnidarian-specific while the rest are shared with other taxa. Of the cnidarian-specific orthogroups, 5,390 are medusozoan-specific, 2,662 are hydrozoan-specific, 2,060 are specific to *Hydractinia* + *Clytia*, and 1,625 are specific to the genus *Hydractinia*. *H. echinata* possesses 46 species-specific orthogroups, while *H. symbiolongicarpus* possesses just 15 such orthogroups. In comparison, there are 2,458 orthogroups specific to anthozoans. Additionally, based on our sampling of 23 bilaterian species from a variety of phyla, there are roughly the same number of orthogroups (19,555) present in at least one bilaterian genome but fewer bilaterian-specific orthogroups (7,998) as compared to Cnidaria.

To evaluate the contribution of conserved gene families to *Hydractinia*'s evolution and evaluate the broad suitability of cnidarians as animal models, we also calculated the overlap of orthogroups between major groups of cnidarians and bilaterians. At the broadest scale, cnidarians and bilaterians possess more shared orthogroups (10,634) than are unshared, with cnidarians possessing more unshared orthogroups (9,558 in cnidarians compared to 8,921 in bilaterians). This supports previous observations based on the genome sequences of *Hydra* (Chapman et al., 2010) and *Nematostella* (Putnam et al. 2007) that much of the cnidarian toolkit predates the divergence of Cnidaria and Bilateria. Splitting Cnidaria further into Medusozoa and Anthozoa (Supplemental Figure S17A), we see that the number of orthogroups unique to Medusozoa + Bilateria is nearly equal to that for Anthozoa + Bilateria, both of which are greater than the number for Medusozoa + Anthozoa. This is consistent with numerous observations of deep divergence between medusozoan and anthozoan genomes. Subdivided further (Supplemental Figure S17B), we see that Hydrozoa possesses a greater number of unique shared genes with Bilateria than it does with the rest of Medusozoa (Cubozoa+Scyphozoa). Hydrozoa also possesses more wholly unshared genes than either of the other medusozoan groups or Anthozoa.

We also examined whether particular cnidarian species have retained more shared ancestral orthogroups than others by calculating how many orthogroups each species shares with bilaterians but with no other cnidarians (Supplemental Table S11 tab X.5). *H. vulgaris* is the clear outlier, having nearly twice as many uniquely shared sequences (352) with Bilateria as the next highest cnidarian (*N. vectensis* with 183). There are no obvious patterns of conservation related to cnidarian taxonomy (i.e., that hydrozoan species always tend to have more of these orthogroups than scyphozoan species). However, the number of unique sequences shared with bilaterians does have a slight but significant linear relationship with the number of sequences in the input proteome (but not with BUSCO score), suggesting that this it may relate more to stochastic characteristics of individual genomes (and the content therein) instead of overall taxonomic patterns.

Per-Species Patterns of Orthogroup Taxon-Specificity

We were interested in the distribution and taxon specificity of these orthogroups across our input taxa to identify potential sources of evolutionary novelty, so we calculated several proportions on a per-species basis: the percentage of genes in the input proteome assigned to orthogroups that are species-specific, the percentage of phylum-specific and metazoan-specific genes, and the percentage of genes not assigned to any orthogroup. These five proportions are visualized in the right panel of Figure 2 for the 15 cnidarian species we analyzed further using CAFÉ (see below). These proportions are visualized in Supplemental Figure S18 for all metazoan species and the data used to create these figures can be found in Supplemental Table S11 tabs X.2 and X.3.

Considering all 49 species included in our OrthoFinder analysis, the percentage of species-specific genes that make up a species' genome ranges from just 0.1% to 4.6%. Across the cnidarians, this proportion ranges from 0.1% (*P. damicornis* and *O. faveolata*) to 2.6% (*H. vulgaris*). These estimates of species-specific genes may be over-estimates as most species included did not have a closely related species included in the analysis; therefore, some of these putatively species-specific genes may in fact be genus- or family-specific. For the species that are the only representatives of their phylum, and where this effect might be most pronounced, genes specific to that

species were reported conservatively as being phylum-specific. Taking this into account, phylum-specific genes made up from 0.5% (*B. floridae*) to 22.6% (*H. symbiolongicarpus*) of each metazoan genome.

Notably, the genomes of *H. symbiolongicarpus* and *H. echinata* contain the highest percentages of phylum-specific genes of all 43 metazoans we examined (23% and 22%, respectively), indicating that their genomes contain the highest percentage of cnidarian-specific genes in all included cnidarians. Coupled with the fact they possess relatively few species-specific orthogroups, this suggests that much of their proteomes may have evolved at the genus, family, or subphylum level, which are grouped together under 'Phylum-specific' in the bulk analysis featured in Fig. 2. It is worth noting that we may be underestimating the number of cnidarian-specific orthogroups in the genomes of the cnidarians that have no close relatives in this study (i.e., we included only one of multiple species of *Hydra*). For these species, orthogroups that would otherwise be genus- or family-specific may be inferred to be unassigned or species-specific, leading to a potential overestimate in those categories, as described above.

We further examined the evolutionary history of the two *Hydractinia* genomes by estimating the taxon specificity of each orthogroup that contains a sequence from that species. We then compared this distribution to those for the two *Aurelia* genomes (Khalturin et al. 2019; Gold et al. 2019) that share similar levels of evolutionary relatedness with other cnidarians than *Hydractinia* does (i.e., within a genus) (Supplemental Fig. S19). The overall chi-square test for a difference in these distributions was significant between all four taxa ($\chi^2 = 589.67$, df = 18, p-value < 2.2×10^{-16}). All post-hoc pairwise comparisons between taxa were significant, but the comparisons between *Aurelia* and *Hydractinia* were much more highly significant (all p-values < 2.2×10^{-16}) than the within-genus comparisons [p-value(*Hydractinia*) = 0.003, p-value(*Aurelia*)=.00004].

Further post-hoc comparisons showed significant differences between *Hydractinia* and *Aurelia* in specific evolutionary age categories (Supplemental Fig. S19), with the largest magnitude of difference observed in the genus-level taxon specificity category. Conversely, both *Aurelia* genomes have significantly more species-

specific orthogroups, as well as greater numbers of medusozoan-specific and metazoan-specific genes. These results suggest that the divergence of the genus *Hydractinia* was accompanied by a comparatively large amount of novelty and that *Hydractinia* may have lost some ancestral genes that *Aurelia* has retained. Further elaboration of the evolutionary ages of genes in other cnidarian genomes will be necessary to understand just how unusual the *Hydractinia sp*. genomes are, how much these distributions differ across cnidarians, and how much these results are affected by taxon sampling.

Identity of Genes Not Assigned to Orthogroups

The proportion of proteins not assigned to any orthogroup varies between 2.8% and 47.4% (mean 18.8%) across all 49 species in our orthology inference dataset, and between 2.8% (*P. damicornis*) and 43.6% (*K. iwatai*) across the cnidarians (white bars in Supplemental Fig. S18). We performed a sequence similarity search of these sequences against the NCBI nr database using DIAMOND and calculated how many of these query sequences yielded no hits at all to nr, and how many had hits to uncharacterized or hypothetical proteins with no further functional information (Supplemental Table S11 tab X.4). The percent of unassigned genes with no nr hits whatsoever varied widely, ranging from 0.3% (*Ciona intestinalis*) to 97% (*Kudoa iwatai*). Generally, 'traditional' model organisms, as well as organisms with small genomes (i.e., *Ciona*, *Saccharomyces*, *Caenorhabditis*, *Tribolium*, and *Drosophila*) have very few (< 5%) unassigned genes with no hits to sequences in the nr databases. On the other hand, seven out of the 10 species with the highest percentages (> 83%) of unassigned genes with no hits were cnidarians, with *H. symbiolongicarpus* and *H. echinata* both having > 88% of their unassigned genes not showing statistically significant similarity to any characterized proteins.

The percent of the remaining sequences (those with nr hits) that had top hits to uncharacterized proteins did not seem to correspond to the extent to which there were no nr hits for the same species. For example, > 99% of all unassigned proteins in the proteomes of *C. intestinalis* and *C. elegans* have hits to nr but, of these, 77% are uncharacterized in *C. intestinalis* versus virtually none in *C. elegans*. Of the small numbers of unassigned *H. symbiolongicarpus* and *H. echinata* sequences that had hits to nr, roughly 9% and 21% of these are

uncharacterized. The number of unassigned genes with no hits may be related to the absence of closely related species in nr, while the number of unassigned genes with uncharacterized top hits may have more to do with the genome completeness and annotation quality for the closely related organisms represented within the nr database.

We investigated whether the 1,246 unassigned proteins in the *H. symbiolongicarpus* data set – 91% of which have no hits or top hits to uncharacterized proteins – were computational artifacts, by assessing whether these unassigned protein models are supported by transcriptomic evidence (see the **Evaluating completeness of predicted gene models** section of this document for details). We calculated the overlap between the genomic coordinates of unassigned proteins and the coordinates of aligned RNA-seq reads and found that 51% of unassigned protein sequences overlapped over more than 90% of their length with a known transcript, suggesting that these proteins are not simply a computational artifact from the annotation or gene prediction processes. This suggests that the 1,128 *H. symbiolongicarpus* proteins unassigned to orthogroups that also have no DIAMOND hits to characterized proteins could represent true evolutionary novelty in *H. symbiolongicarpus*. Similar mappings of expression data to 'orphan' proteins in other taxa may yield similar results, meaning they should be examined carefully and not simply discarded in downstream analyses.

Estimating the evolutionary dynamics of gene families using CAFÉ

We characterized gene family size dynamics amongst the 15 cnidarian species by using the software package CAFE v. 4.2.1 (De Bie et al. 2006; Han et al. 2013) to estimate ancestral gene family sizes and changes in gene family size, as well as to infer which gene families are significantly faster evolving in specific cnidarian lineages. As input, CAFE uses our time-calibrated tree and the gene counts per species for a subset of the orthogroups inferred by OrthoFinder and selected to meet a set of criteria described below.

In the context of analyzing evolutionary dynamics, CAFE assumes that estimations are only calculated for orthogroups present in the common ancestor of the in-group. To analyze the evolutionary dynamics of the most gene families possible, we chose the non-cnidarian outgroup from those included in our phylogenetic analyses that would maximize the number of orthogroups present in the common ancestor of that taxon and cnidarians, as estimated by calculating the number of orthogroups present in both that outgroup and at least one cnidarian. Using this metric, we ended up focusing on genes inferred to be present in the common ancestor of Bilateria and Cnidaria – that is, gene families present in at least one bilaterian and one cnidarian. Therefore, our input data to CAFE consisted of the subtree of our time-calibrated tree estimated as above containing only members of Bilateria and Cnidaria, as well as the matrix of gene family sizes per species estimated by OrthoFinder for gene families present in the selected species (Supplemental Data S16). This matrix is then filtered by CAFE to include only gene families inferred to be in the common ancestor of the included species (8433 total families).

Before running CAFE to estimate ancestral gene family sizes and gene family gains/losses over the selected subtree, one first needs to estimate a value for lambda (λ), the symmetrical gene birth-death rate for the entire tree expressed in gains or losses per gene per million years. To estimate λ, it is recommended that only orthogroups with low variance in gene family size amongst taxa be used; this can be achieved by selecting those with fewer than 100 sequences per species. We were able to estimate λ using 8,391 of the 8,433 orthogroups meeting this criterion (Supplemental Data S17). As a test of how robust our results would be based on outgroup choice and the number of input orthogroups, we estimated λ using different sets of the possible non-bilaterian outgroups, the

relevant time-calibrated subtrees, and the input orthogroup matrices. We found that inclusion of the different non-bilaterian outgroups (and, therefore, different sets of gene families) did not appear to greatly affect the estimation of λ (Supplemental Table S11 tab SM3). Using our estimate of λ for the Cnidaria+Bilateria subtree, we then ran CAFE again on all 8,433 loci.

Post-processing and visualizing CAFE data

The CAFE output contains estimates of ancestral gene family sizes, the location and size of gene family expansions and contractions, and Viterbi p-values for these changes in gene family size that are used to determine which families are evolving significantly quickly on specific branches of the Cnidaria+Bilateria tree. For downstream analyses, we treated changes in gene family size with Viterbi p-values <= 0.05 as representing significant increases or decreases. The output from processing the raw CAFE reports using CAFE accessory scripts can be found in Supplemental Data S18-S27. These were further analyzed and visualized in R, specifically using the GGtree (Yu et al. 2017), Phytools (Revell, 2012) and DeepTime (https://github.com/willgearty/deeptime) R packages to create elements of Fig. 2.

Focusing just on the Cnidaria + Bilateria subtree inferred using RaxML+r8s (described above), we estimated the evolutionary dynamics (i.e., gene family expansions, contractions, and losses) of the 8,433 OrthoFinder-inferred orthogroups that are present in the ancestor of this subtree. Estimates of gene family dynamics estimated for each node and terminal taxon by the software CAFÉ (De Bie et al. 2006; Han et al. 2013) for our Cnidaria + Bilateria tree are summarized in Fig. 2 (left panel) and presented in more detail in Supplemental Table S11 tab X.8. Raw output files from the CAFE run, including estimated gene family size for every orthogroup at every node in the tree, are presented as Supplemental Data S16-S27. Our estimate of the birth-death rate across the entire tree, lambda (λ), is 0.001 gains and losses per gene per million years. Because λ is a symmetrical birth-death rate, this translates to a gain of approximately 157 gene copies and a loss of the same number of copies per million years for the 314,754 sequences in our 8,433 input orthogroups.

Across the whole tree (Fig. 2), more changes in gene family size take place on the terminal branches of the tree as compared with internal branches of the tree. Terminal branches have significantly more gene expansion or contraction as compared to internal branches [mean(terminal) = 2,375.7, mean(internal) = 1007, t = -8.5139, df = 33.99, p-value = 6.07×10^{-10}]. For the lineages leading to our *Hydractinia* species, this pattern is very clear: 131 gene families have expanded in Cnidaria as compared to the Cnidarian + Bilaterian ancestor, 87 have expanded in the ancestor to Medusozoa, and 112 have expanded in the Hydrozoan ancestor. This is in stark contrast to the ancestor to the genus *Hydractinia* (998 expanded) and either species of *Hydractinia* (1469 for *H. echinata* and 788 for *H. symbiolongicarpus*). Roughly a third (34%) of the 8,433 orthogroups present in the common ancestor are still present in all 19 of the extant taxa, the rest having been lost (that is, gene family size is estimated to be zero) sometime during the evolution of these taxa. For our taxonomic groups of interest, 57%, 46%, and 36% have been retained in all hydrozoans, medusozoans, and cnidarians, respectively.

Uniquely fast-evolving genes

We were particularly interested in the orthogroups that were only inferred to have significant changes in size on one or more of our lineages of interest – that is, those leading from the common ancestor of all cnidarians to our two *Hydractinia* species and nowhere else in our CAFE input tree. These might represent important lineage-specific evolutionary changes in ancient, conserved gene families present in the common ancestor of Cnidaria + Bilateria.

Here, we identified genes that were inferred by CAFE to only have significantly changed size on the branch leading from the ancestor of the genus *Hydractinia* to one of the two *Hydractinia* species, an observation that can be interpreted as either species-specific expansions or contractions. There are three such orthogroups on the branch leading to *H. echinata*, all of which represent gene family expansions, in contrast with non-significant losses for *H. symbiolongicarpus* for the same orthogroups. The main annotations for *H. echinata* sequences within these orthogroups are protein tyrosine phosphatase activity, exonuclease activity, and Fido domain-containing proteins, respectively. There are two significant size changes, both contractions, on the branch leading to *H.*

symbiolongicarpus, one annotated as a family of forkhead-box-domain-containing proteins and one annotated as having a putative microtubule activity/dynein complex-related function.

There is also a single such family on the branch leading to the genus *Hydractinia* from its MRCA with *C. hemisphaerica*, which was inferred to have gained 10 copies as compared with its MRCA. The *H. echinata* sequences in this orthogroup are annotated as aquaporin-like proteins with channel activity/transmembrane transport functions. In their divergence from their genus-level common ancestor, *H. symbiolongicarpus* subsequently lost one of these copies while *H. echinata* gained another two members of this orthogroup, neither of which are inferred to be significant changes.

There are no other orthogroups beyond those mentioned above that are uniquely fast-evolving on only one of the branches of our input tree. There are, however, a number of orthogroups inferred to be significantly changing not within just our lineages of interest, but along multiple branches. For example, there are 16 orthogroups that are inferred to have significantly changed size on the branches leading to *H. symbiolongicarpus* and *H. echinata* but nowhere else. Notably, 14 out of the 16 orthogroups represent significant gains for *H. echinata* but significant losses for *H. symbiolongicarpus*, while the other two represent the reverse scenario. There are no cases in which they are significantly changed in size in a parallel fashion, an observation that is in line with the overall patterns of evolution between these species, as discussed above. The annotated *H. symbiolongicarpus* and *H. echinata* sequences within these groups represent a variety of potential functions, including one annotated as a potential cnidarian-specific nematogalectin (OG0001542).

There are 10 orthogroups inferred to have undergone significant size changes on the branch leading to the genus *Hydractinia* and along both species-specific branches. Each of these is a significant expansion on the lineage leading to *Hydractinia* and, in all but one case, *H. echinata* has continued to gain gene copies while *H. symbiolongicarpus* has subsequently contracted. Like the orthogroups described above, the annotations for

sequences in these orthogroups represent a variety of putative functions, and in fact four of the orthogroups have no annotations available for any of the *H. symbiolongicarpus* and *H. echinata* sequences within it.

Finally, there is a single orthogroup that is evolving on all the descendent lineages from the MRCA of Hydrozoa leading to either of the *Hydractinia* species, including on the branch leading to the MRCA of *Hydractinia* + *Clytia*. Incredibly, this orthogroup is inferred to have expanded by 31 copies in *H. echinata*, adding to the 51 copies gained during the evolution of the genus *Hydractinia*. Once again, *H. symbiolongicarpus* has been inferred to have lost sequences in the same time frame as this gain in *H. echinata*. Given the number of *Hydractinia sp*. sequences in this orthogroup, there are a variety of annotations that are provided in Supplemental Table S11 tab X.9, along with information about all the aforementioned orthogroups. Tables with information about annotations, presence in rapidly evolving orthogroups, and orthogroup taxon-specificity for each gene model in the *H. symbiolongicarpus* and *H. echinata* genomes can be found in Supplemental Table S11 tabs X.10-X.11.

Comparing evolutionary dynamics of H. symbiolongicarpus and H. echinata

Roughly half of the orthogroups present in the genomes of *H. symbiolongicarpus* (0.50) and *H. echinata* (0.54) have undergone some change in size as compared to the ancestor of Cnidaria + Bilateria. Despite having a similar proportion of gene families that changed size at all on these terminal branches (24% in *H. echinata* vs. 21% in *H. symbiolongicarpus*), they have very different proportions of gains vs. losses over these branches. This implies that *H. symbiolongicarpus* and *H. echinata* appear to have undergone very different evolutionary trajectories since their divergence roughly 19 MYA. *H. echinata* has experienced 1.9 times as many expansions since divergence from *H. symbiolongicarpus*, with nearly 1.5 times as many gene copies gained per expansion. Taken together, this means that *H. echinata* has gained about twice as many (1.97x) individual gene copies in the past 19 million years. Conversely, *H. symbiolongicarpus* has about 70% more contracted gene families and has lost nearly 50% more genes per contraction, meaning that *H. symbiolongicarpus* has lost nearly 2.5 times more genes in total as has *H. echinata* has since their divergence. The actual gene families changing in size after divergence are also largely non-overlapping, with only three gene families that have changed size in the same direction for both

species. Similarly, although *H. echinata* and *H. symbiolongicarpus* have lost 248 and 252 gene families, respectively, the identities of the lost families are completely non-overlapping.

This pattern holds when examining only the significant rapidly evolving genes on each terminal branch. The number of total significant changes is roughly similar (177 for *H. echinata* vs. 162 for *H. symbiolongicarpus*), but they have nearly inverse proportions of expansions vs contractions (85% contractions for *H. symbiolongicarpus* vs. just 13.5% for *H. echinata*). These striking differences cannot be explained by *H. symbiolongicarpus* having a less complete genome than *H. echinata*, which would create both false *H. symbiolongicarpus*-specific gene losses and make detection of some putatively *H. echinata*-specific expansions in *H. symbiolongicarpus* difficult, as both proteomes are highly complete as assessed by BUSCO v. 5.0 (90.7% of single-copy orthologs present in *H. echinata* vs. 92.5% in *H. symbiolongicarpus*). It is possible that the pattern of greater expansion observed in *H. echinata* may mirror the overall difference in size between the two *Hydractinia* genomes or the slightly higher amount of duplication apparent in the *H. echinata* genome (10.2% duplicated BUSCO orthologs in *H. symbiolongicarpus* compared to 12.3% in *H. echinata*).

Comparing evolutionary dynamics of Hydractinia vs. H. vulgaris and C. hemisphaerica

The other hydrozoans we included in our analyses, *H. vulgaris* and *C. hemisphaerica*, have more taxon-specific orthogroup size changes than either species of *Hydractinia* (see analysis above). As is the case with most of the tree, most of the observed changes in gene family size are concentrated on the terminal branches within Hydrozoa as compared to the relevant internal branches (Fig. 2, Supplemental Table S11 tab X.8). *C. hemisphaerica* and *H. vulgaris* each have a much higher rate of genes acquired per expansion (3.4 and 5.2 genes/expansion, respectively) compared to *H. symbiolongicarpus* and *H. echinata* (1.3 and 1.9 genes/expansion, respectively). Ultimately, this results in *H. vulgaris* gaining over 8,219 putatively novel gene copies along the terminal branch, which is 2.2 times more than gained by *C. hemisphaerica*, 2.8 times more than in *H. echinata*, and nearly eight times as many gene copies gained compared with *H. symbiolongicarpus*. *H. vulgaris* and *C. hemisphaerica* also

have a greater number of lost gene copies compared to the *Hydractinia* species, in keeping with the larger overall amount of change on those terminal branches.

One explanation for the larger apparent amount of taxon-specific change for *C. hemisphaerica* and *H. vulgaris* is that those numbers represent changes unique to each of these species plus changes unique to that whole genus. For example, some of the size changes that are inferred to be on the *C. hemisphaerica* terminal branch may in fact have occurred earlier in evolutionary history and be common to all members of the *C. hemisphaerica* genus. Two observations suggest that this is the case. First, the number of changes on the lineage leading the *Hydractinia* genus plus the species-specific changes for either *Hydractinia* species roughly add up to the number of changes on either the *C. hemisphaerica* or *H. vulgaris* terminal branches. Second, when counting the number of significant changes in family size along a branch, more are inferred to have taken place on either of the *Hydractinia* terminal branches than is the case for both *C. hemisphaerica* or *H. vulgaris*.

Comparing gene family evolution in the genus Hydractinia to the genus Aurelia

Including the two lineages of *A. aurita* (Khalturin et al. 2019; Gold et al. 2019) in this analysis allows us to assess if the major differences in post-divergence gene family evolution seen in the two *Hydractinia* species is unusual. In contrast with *Hydractinia*, the *Aurelia* lineages have different overall percentages of genes that have changed size since their divergence 45 MYA (Khalturin (Baltic): 24%, Gold (Pacific): 39%, about 1.6 times more gene families). The relative importance of gains vs. losses in these post-divergence differences is much more similar between *Aurelia* genomes compared with the *Hydractinia* genomes. In keeping with the percentages of genes changing size at all, we infer that the Pacific lineage has experienced roughly 1.6 times more of both expansions and contractions compared with the Baltic lineage. The Baltic lineage has a higher average number of genes gained/expansion (1.4 times as many), while the Pacific lineage has 1.2 times more genes lost/contraction. Taken together with the higher number of contraction/expansion events for the Pacific lineage, this means that the lineages have roughly even numbers of total genes acquired (1.1x), but more total genes lost in the Pacific lineage (1.4x). The higher rate of genes/loss and number of losses has also resulted in the Pacific lineage having lost 3.2

times as many gene families as the Baltic lineage. As in *Hydractinia*, there is no overlap between the lost gene families in the two lineages, and there are only 14 gene families that have changed size in the same direction in both *Aurelia* lineages.

Differences in assembly quality may have contributed to the results described above. According to our BUSCO analyses, the proteome of the Pacific lineage (66.2%) is less complete than that of the Baltic lineage (74.2%), suggesting that the higher rate of gene copy loss in the Pacific lineage might be in part an artifact of incompleteness. Additionally, the Pacific lineage has almost four times the number of duplicated BUSCO genes than the Baltic lineage (8.3% vs. 0.9%), which could inflate the number of Pacific-specific expansions. Both factors could be contributing to the inferred higher rate of gene family size change overall in the Pacific lineage. For both pairs of species, *Aurelia* and *Hydractinia*, we have evidence for very stochastic and/or taxon-specific processes as there is very little concerted evolution of gene families in either pair of proteomes, as evidenced by the lack of overlap in evolving gene families. These results are also consistent with the overall pattern of increased rates of evolution in the terminal branches of the tree.

The non-coding RNA landscape: miRNAs

We isolated RNA from five samples of adult *H. echinata* polyps using a miRNeasy kit (Qiagen), froze the RNA, and shipped it on dry ice to the NIH Intramural Sequencing Center where small RNA-seq libraries were constructed with an Illumina TruSeq Small RNA library prep kit and sequenced on an Illumina HiSeq 2000 instrument as 125 base pair reads. About 30-80 million reads were obtained for each library. The resulting reads were trimmed with Cutadapt version 1.6 (Martin 2011) and mapped to the *H. echinata* genome using the miRDeep2 mapping algorithm (Friedländer et al. 2012). After mapping, the miRDeep2 algorithm predicted miRNAs. Predictions with a score of 5.0 or greater were retained. To find the highest quality predicted miRNAs, we filtered the miRDeep2 output to retain predicted miRNAs with (1) a miRDeep2 score of 5.0 or greater, (2) sequences with at least one mismatch in the mature/star duplex (creating a bulge), (3) mature strand homogeneity over 50%, (4) and star strand homogeneity above 50%. Following this, the predictions were manually screened.

Predictions were discarded if they were predicted to have an almost perfect duplex (with no bulges in the middle of the duplex; characteristic of siRNAs), if they contained predicted side bulges, or if the number of reads in the loop sequence region was equal to the number of reads in the mature sequence. 347 miRNAs were predicted by miRDeep2. Of those predictions, 104 passed our custom filtering. During manual screening, 49 of these were deemed high quality predictions and 23 were found to be low quality. There was some redundancy in the list of high-quality predictions. A final list of 38 unique high quality mature miRNA sequences is listed in Supplemental Table S18 and is available on the *Hydractinia* Genome Project portal: (https://research.nhgri.nih.gov/hydractinia/download/index.cgi?dl=mi).

The non-coding RNA landscape: rRNAs, tRNAs, snoRNAs

Annotation of ncRNAs

Candidate non-coding RNA (ncRNA) regions were identified computationally using Infernal (v1.1.2) (Nawrocki and Eddy 2013), Rfam (14.1) (Kalvari et al. 2018a), and tRNAscan-SE v2.0.5 (Chan et al. 2021). All Rfam 14.1 models except tRNA models RF0005 and RF01852 were used to search each genome sequence file separately, using Infernal's cmsearch program with the command-line options *--cut_ga*, *--rfam*, and *--nohmmonly* (Kalvari et al. 2018b). Hits with an E-value of $< 10^{-5}$ were kept and, in the case of overlapping hits, the hit with the higher bit score was retained. Across both genomes, 62 hits from 15 Rfam families not expected to identify homology in cnidarian genomes were removed after manual examination revealed they were likely false positives or contamination. The remaining final set of Infernal and Rfam-based annotations includes 3,596 predictions from 31 families in *H. echinata* and 2,980 predictions from 32 families in *H. symbiolongicarpus*. tRNAscan-SE was used with default parameters to identify 28,055 putative tRNAs in *H. echinata* and 24,077 putative tRNAs in *H. symbiolongicarpus*. Of these, 4389 (15.6%) putative tRNAs from *H. echinata* and 3333 (13.8%) putative tRNAs from *H. symbiolongicarpus* were classified as potential pseudogenes by tRNAscan-SE. The number of predictions per family is shown in Supplemental Table S19. Additional information on the RNA annotations, including GFF files of the predictions with data on tandem array membership and HydSINE1 overlap and pseudogene

information for tRNAs, can be found as downloads on the *Hydractinia* Genome Project Portal at https://research.nhgri.nih.gov/hydractinia/download/index.cgi?dl=sd. Tandem Repeat Finder (TRF, version 4.09) (Benson 1999) was run from the command line using the following command: *trf409.linux64 <fastafile> 2 2 7 80 10 50 2000 -d*. Results were filtered to report only repeat regions with a copy number of 7 or greater, a period length of 50 nt or greater, and an average percent match identity of 75% or greater. The resulting final predictions are shown in Supplemental Table S19.

Manual examination of candidate Rfam hits

To identify likely false positives and potential contamination, we scrutinized all candidate hits for Rfam families not expected to be present in cnidarian genomes based on the taxonomic distribution of those families across previously characterized genomes in the Rfam database. Such families were identified as any Rfam family for which no sequences in the so-called *seed* alignment that represents the family are from the Eukaryotic domain (Rfam families Bacteria_small_SRP, LSU_rRNA_archaea, LSU_rRNA_bacteria, Lysine, and SSU_rRNA_bacteria) or for which all Rfam seed sequences are from the same eukaryotic phylum and whose phylum is not *Cnidaria* (SSU_rRNA_microsporidia, mir-16, mir-191, mir214, and sn668). Ten families met these criteria, along with five additional families that did not satisfy the criteria above but are either primarily bacterial families with no seed sequences from *Cnidaria* (RNaseP_bact_a, RNAseP_bact_b, TPP, and tmRNA) or for which all hits were not deemed correct because they appeared to be simple inverted repeats (mir-598); we determined that these hits were likely false positives or contamination and removed them from our set of candidate hits. The remaining hits comprise the final set of Rfam predictions that are included in the GFF files available on the *Hydractinia* Genome Project Portal at https://research.nhgri.nih.gov/hydractinia/download/index.cgi?dl=sd. For *H. echinata*, there are 3,596 total predictions across 31 families. For *H. symbiolongicarpus*, there are 2,995 total predictions across 38 families (Supplemental Tables S19-S21).

All tRNAscan-SE hits were subsequently annotated. Metadata on those hits that were flagged as potential pseudogenes ('pseudo' string present in the 'Note' column of the -o output files) and that overlapped with RepeatMasker annotations of the *HydSINE1* model was included in the GFF files, as described below. For *H. echinata*, there are 28,055 total tRNA predictions across 24 tRNAscan-SE isotype models. For *H. symbiolongicarpus*, there are 24,077 tRNA predictions (Tables S19, S22-S23).

Definition of fragments and high-scoring predictions

We further classified Rfam and tRNAscan-SE predictions based on their score and lengths as follows. A prediction is deemed 'high scoring' if its cmsearch bit score is within 10% of the score of the highest scoring prediction for that family in the genome. A prediction is a 'fragment' if its length is less than 90% the length of that top-scoring prediction. Tables S19-S20 include counts of each class for the Rfam predictions. These score and length classifications are provided for all hits in the GFF files (Supplemental Data S28-S36).

Many RNA predictions occur in tandem arrays

We observed that many RNA predictions from several Rfam families occur in roughly evenly spaced tandem arrays of tens or even hundreds of nearly identical or similar copies. We describe below some statistics and notable characteristics about the tandem arrays, but the biological significance of these arrays remains unclear. In *H. echinata*, for six of the 12 Rfam families with at least one sequence with ten or more predictions on the same strand, more than half of the predictions are in tandem arrays of 10 or more predictions. In *H. symbiolongicarpus*, the same is true for six of the 10 Rfam families (Supplemental Table S19). The statistics are more striking if only *eligible* predictions, defined as predictions which occur on sequences and strands with at least 10 predictions, are considered. More than 90% of these eligible predictions are in tandem arrays for 11 of the 13 Rfam families in *H. echinata* and for seven of the 12 Rfam families in *H. symbiolongicarpus* that have eligible predictions (Supplemental Tables S24, S25). For both genomes, these sets include the 5S and SSU ribosomal RNAs, four of the five RNA components of the major spliceosome (U1, U2, U5, and U6), and the small nucleolar RNA U3. As an example, 111 of the 132 U6 *H. echinata* predictions are in a tandem array on the forward (+) strand of

sequence *HyE0823*, and 108 of the 110 intergenic regions between the 111 U6 RNAs are between 274 and 282 nucleotides long (Supplemental Table S25).

Most of the tRNA predictions also occur in tandem arrays. Considering each isotype model independently (that is, with no array containing predictions for more than one isotype), 55.6% of the tRNA predictions are in tandem arrays in *H. echinata*, while 63.0% of the tRNA predictions are in tandem arrays in *H. symbiolongicarpus*. Of the 24 tRNAscan-SE isotype possibilities – the 20 amino acids plus selenocysteine (SeC), initiator methionine (iMet), suppressor (Sup), and undetermined (Undet) – all but three (Sup, Undet, and SeC) have at least 10 predictions in tandem arrays in each genome, and for seven of the isotypes, there is at least one tandem array of size 100 or more in one of the two genomes. The largest tRNA tandem array occurs in the *HyE0174* sequence in *H. echinata*, with 254 predicted proline tRNAs where 83% of the intergenic regions are between 192 and 272 nucleotides in length (Supplemental Tables S19, S24). For this analysis, a tandem array is defined as 10 or more predictions of the same family on the same sequence and strand in which at least 75% of the lengths of intergenic regions between predictions are within 100 nucleotides of each other (see *Definition of Tandem Arrays*, below).

The majority of the tandem arrays consist of a single gene, but there are some examples of combinations of genes that co-occur in arrays. A tandem array of ten LSU rRNA, 5.8S rRNA, and SSU rRNA (the trio of LSU followed by 5.8S followed by SSU repeated 10 times) is observed in sequences *HyE0249* and *HyE0522* of *H. echinata*. The same configuration of nine copies of these genes is present in sequence *HyS0316* of *H. symbiolongicarpus*. Some sequences include arrays of multiple tRNA genes, which are sometimes encoded on both strands of the sequence. For example, sequence *HyS0042* includes about 70 consecutive sets of three tRNAs in order: tRNA-Met, tRNA-Val, and tRNA-Gln. The most complex example we found of co-occurring genes in an array is 20 copies of tRNA-Arg, U2, U1, 5S_rRNA, and three histone 3' UTR stem loop structures (Rfam family Histone3) along with multiple CDS with homology to Pfam domains from histone genes, in *H. echinata* on sequence *Hech0368*. While the tRNA-Arg, U2 and U1 genes are encoded on the positive strand, the 5S and two of the histone UTR stem-loops are encoded on the negative strand. This tandem array of co-occurring genes had been previously described

from an earlier draft of the *H. echinata* genome (Török et al. 2016). The same configuration of genes exists in an array of ten copies in *H. symbiolongicarpus* on sequence *Hsym0385*.

Definition of tandem arrays

Many of the ncRNA predictions within the same family occur in tandem arrays separated by roughly the same number of nucleotides. We define a tandem array as a set of $X \geq 10$ contiguous predictions of the same family on the same sequence and strand such that $N \geq (0.75 * (X - 1))$ of the spacer lengths $S_1, S_2, ... S_N$ between predictions are within 100 nt of each other ($(max_{i=1..N} S_i - min_{i=1..N} S_i) < 100$ nt), where S_i is the distance between the final nucleotide of prediction *i* and the first nucleotide of the next prediction. Further, the distances between the first two predictions and the final two predictions must both be within $(max_{i=1..N} S_i - min_{i=1..N} S_i)$ and no S_i value can be greater than $3*(max_{i=1..N} S_i)$. Supplemental Tables S19, S24 (*H. echinata*), and S25 (*H. symbiolongicarpus*) include tandem array data for all tRNAscan-SE isotype models and Rfam families with 10 or more predictions, also listing the sequence and strand with the largest array for each family. For the tRNAscan-SE predictions, each isotype is treated independently such that each tandem array identified only contains predictions from one isotype model.

Analysis of overlaps with known RepeatMasker repeats

Some transposable elements are derived from RNAs, including short interspersed nuclear elements (SINEs) that originate from RNA polymerase III-transcribed ncRNAs such as tRNA, 5S rRNA, and SRP RNA (Kapitonov and Jurka 2003; Kramerov and Vassetzky 2005; Sun et al. 2007). To determine if a significant fraction of the predictions of the high copy number ncRNAs were actually known SINEs or other repetitive elements, we analyzed overlaps between the RNA predictions and RepeatMasker predictions by considering all possible pairwise combinations of RNA family and repeat family for which there were more than 10 instances of overlap. For this analysis, we considered all tRNAscan-SE predictions, which are isotype-specific, as belonging to the same family of ‘tRNA’ and excluded the RepeatMasker RNA models with the ‘class/family’ value of ‘RNA’,

‘rRNA’, ‘scRNA’, ‘srpRNA’, and ‘tRNA’. We found *significant overlap* between only one ncRNA family and repeat family pair: tRNA and the HydSINE1 repeat. Specifically, two criteria were required for a pair to significantly overlap: (1) the average overlap length of all overlaps must be at least half of the RNA model length, and (2) on average, at least half of the repeat model *not* involved in the RNA overlap must be covered by the repeat prediction. For criterion 1, we used 71 as the tRNA model length as that is the length of the Rfam tRNA model (RF00005) covering all tRNA isotypes. Criterion 2 is important because many SINE repeat families have regions that are homologous to tRNA but include extra sequence as well (Sun et al. 2007). Consequently, RepeatMasker identifies tRNAs as high-scoring matches to these models, but the hits do not extend outside the region of tRNA homology; this indicates that they are not plausible full-length instances of the SINE family. Enforcing criterion 2 eliminates such repeat families by requiring that at least half of the additional repeat model region outside the tRNA be included in the repeat hits, on average. The tRNA/HydSINE1 criterion 2 ratio value is 0.877 for *H. echinata* and 0.859 for *H. symbiolongicarpus*, making it an outlier relative to all other RNA/repeat pairs. In *H. echinata*, the pair with the next highest criterion 2 ratio is tRNA/SINE22 PXu in *H. echinata* and tRNA/ALPINE2 (another SINE) in *H. symbiolongicarpus*, with values of 0.313 and 0.404, respectively. The highest criterion 2 ratio for an RNA/repeat pair for which the RNA is not tRNA is 0.244 for 5S rRNA and GymnSINE in *H. symbiolongicarpus*.

The significant overlap observed between tRNA and HydSINE1 is expected given that the HydSINE1 repeat was discovered in *H. symbiolongicarpus* (Nishihara et al. 2016). Of the 28,055 and 24,077 tRNA predictions in *H. echinata* and *H. symbiolongicarpus*, 924 from *H. echinata* and 403 from *H. symbiolongicarpus* overlap with HydSINE1 calls. Of those that overlap, nearly all are flagged as potential pseudogenes by tRNAscan-SE [896 (97.0%) in *H. echinata* and 394 (97.8%) in *H. symbiolongicarpus*]. In contrast, only 15.6% and 13.8% of all tRNA predictions in the two genomes, respectively, are flagged as potential pseudogenes. Only one tRNA prediction from either genome that overlaps with a HydSINE1 prediction is a member of a tandem array, whereas more than 50% of all tRNA predictions are. There are an additional 4,555 HydSINE1 RepeatMasker predictions in the *H. echinata* genome and 3,801 predictions in the *H. symbiolongicarpus* genome that were not detected by

tRNAscan-SE, most likely due to low scores resulting from the divergence of the tRNA homology region. The GFF annotation files (available through the *Hydractinia* Genome Project Portal at https://research.nhgri.nih.gov/hydractinia/download/index.cgi?dl=sd) include metadata that indicates which tRNA predictions overlap with HydSINE1, which are flagged as potential pseudogenes by tRNAscan-SE, and which exist in a tandem array.

It is possible that additional repetitive elements not represented in the set of RepeatMasker models (including SINEs) overlap significantly with our RNA predictions. However, the high fraction of our RNA predictions present in tandem arrays suggests that most of our predictions are not SINEs which, like the HydSINE1s we observe overlapping with tRNA predictions, would be expected to be more sporadically distributed and not organized in tandem arrays.

The homeobox gene complement of *Hydractinia*

Retrieving homeodomain sequences from the Hydractinia *gene models*

Homeodomain sequences from multiple metazoan species were downloaded from the HomeoDB website (http://homeodb.zoo.ox.ac.uk/) and the Homeodomain Resource Database (https://research.nhgri.nih.gov/homeodomain/). These sequences were concatenated into a single list and all redundant sequences were removed. Using this non-redundant sequence set, gene model databases (both protein and nucleotide) from each *Hydractinia* genome were mined for putative homeobox sequences using BLAST+ (2.2.31) (Camacho et al. 2009) using an E-value cut-off of 1 x 10-3. Sequences containing homeodomains were also identified using the HMMER (v. 3.1b1) program HMMSEARCH, using the 'Homeodomain' hidden Markov model (PFAM: PF00046) to detect remote homologs (Mistry et al. 2013). To identify putative homologs between both *Hydractinia* species, a sequence alignment of the homeodomain of all sequences from both *H. echinata* and *H. symbiolongicarpus* was generated using MAFFT (Katoh et al. 2002) with default settings (algorithm: auto;

scoring matrix: BLOSUM62; gap open penalty: 1.53; offset value: 0.123), and pairwise matches between each species were manually identified (Supplemental Table S27 tab 'Hech_v_Hsym_pairwise').

Homeodomain superfamily tree alignment

For each *Hydractinia* species, homeodomain sequences were initially aligned to the dataset from (Ryan et al. 2010) with the *Mnemiopsis leidyi* sequences removed. The resulting dataset contained sequences from human, *Drosophila melanogaster*, *Hydractinia* spp., and several species that contain representatives of homeodomain families that are not found in either human or *Drosophila*. Sequences were aligned using MAFFT (Katoh et al. 2002) with a gap penalty of 3. For the superfamily tree analysis, the three amino acid extensions between the first and second helices associated with TALE homeobox genes were removed. Gaps caused by uninformative/atypical residues were manually removed. This final 60 amino acid homeodomain alignment was used for subsequent phylogenetic analyses (Supplemental Data S37-S38).

Phylogenetic analysis of the homeodomain superfamily

Homeodomain superfamily trees were generated using RAxML v8 (Stamatakis 2014) and the MPI implementation of MrBayes (v3.2.6) (Altekar et al. 2004; Huelsenbeck and Ronquist 2001; Ronquist and Huelsenbeck 2003). The program Prottest3 (Darriba et al. 2011) was used to evaluate the best fitting evolutionary model for the dataset (LG + G). RAxML was carried out using 10 randomized maximum parsimony starting trees (the default setting). The tree with the best log likelihood score was then selected. 1000 bootstrap support values were calculated and applied to the maximum likelihood (ML) tree (Supplemental Data S39-S40). For the Bayesian analysis, two runs of five chains were carried out for a total of 5 million generations. Trees sampled from both runs were combined and summarized using the sumt command, discarding the first 25% as burn-in and generating the final consensus tree (Supplemental Data S41-S42). Both tree files were loaded into FigTree v1.4.3 for downstream editing. Finally, these trees were loaded into Inkscape for highlighting and final preparation.

Class-level annotation of the Hydractinia *homeobox complement*

The topologies of each superfamily tree were compared and the class-level phylogenetic position of all *Hydractinia* proteins were noted (Supplemental Table S26). Secondary domains were predicted using the InterProScan plugin of the Geneious software package (Jones et al. 2014; Kearse et al. 2012) (Supplemental Table S26). Phylogenetic information was combined with secondary structure domain annotation and atypical/identifying amino acids, and these data were used to classify each homeobox gene into its respective family (Supplemental Tables S26, S27). For example, homeobox genes that clustered with the clade containing LIM genes that also contained a LIM domain were classified as LIM homeobox genes. Similarly, genes that were grouped with known TALE class genes also contained the characteristic three amino acid extension between the first and second helix of the HD and were classified as TALE genes. Final class-level assignments for each homeobox protein can be found in Supplemental Table S26.

HOX-L subclass annotation of Hydractinia *ANTP homeobox genes*

For each *Hydractinia* species, proteins belonging to the ANTP class of homeobox proteins were aligned to ANTP proteins from human, *Drosophila*, *Nematostella vectensis* and other invertebrate homeodomains collected from (Pastrana et al. 2019) (Supplemental Data S43-S44). Maximum likelihood and Bayesian phylogenetic trees were run as above and *Hydractinia* sequences in the HOX-L subclass were identified (Supplemental Data S45-S48).

Synteny analysis of cnidarian HOX-L genes

Genomic locations for Hox and ParaHox proteins were examined in each *Hydractinia* species and compared to several cnidarian species (Khalturin et al. 2019; Leclère et al. 2019; DuBuc et al. 2012; Zimmermann et al. 2023). Putative orthologs were detected through a combination of BLAST analyses and previous annotations and analyses of Hox genomic loci.

The allorecognition complex

BLASTP searches with each reference gene model amino acid sequence as query sequences were performed against a local database of all known Alr1 and Alr2 alleles. For each gene with sequence similarity to Alr1 or Alr2, SignalP 5.0 (Almagro Armenteros et al. 2019) was used to determine whether it had a signal peptide followed by TMHMM version 2.0 (Krogh et al. 2001) to identify any transmembrane helices. The BLAST results were also used to determine which domains it shared with Alr1 and Alr2. This information was used to classify the reference gene models as likely single-pass transmembrane proteins with Alr1/2 homology. Each gene model's location was then plotted on a genomic scaffold (Supplemental Fig. S29). The figure and position of the genes is to scale.

Single-cell transcriptomics of adult animals

Cell dissociation and preparation

Ten feeding polyps, plus ten sexual polyps and stolon tissue was dissected from *H. symbiolongicarpus* clone 291-10. This material was split into two tubes with five feeding polyps and five sexual polyps in each tube. Tissue was rinsed with calcium- and magnesium-free artificial seawater (CMFASW) with EGTA three times. Tissue was dissociated enzymatically with 200µl of 1% Pronase E (Catalog# 97062-916, VWR) in CMFASW with EGTA with gentle pipetting about 6-8 times with a glass pipette coated with gelatin every 15 minutes for 90 minutes total. The cell suspension was filtered through a 70µm Flowmi cell filter (Catalog# H13680-0070, SP BEL-ART). The suspension was then spun and pelleted at 300rcf for 5 minutes at 4C. The supernatant was discarded, and the pellet gently resuspended with a p200 in either CMFASW without EGTA or 3XPBS and mixed well. This cell suspension was filtered through a 40µm Flowmi cell filter (Catalog# H13680-0040, SP BEL-ART) into a new tube and kept on ice. Cells were counted with a hemocytometer and diluted to a final concentration of $1x10^6$ cells/ml.

10X encapsulation, library preparation, and RNA sequencing

Approximately 9.6µl of the final cell suspension for each sample was loaded on a 10X Chromium Controller and subsequently used for 10X single cell 3' version 3 RNAseq library construction at UF's Interdisciplinary Center for Biotechnology Research. Library quality was verified with the Agilent High Sensitivity D5000 ScreenTape System. In total, ~9600 cells were loaded onto the 10X instrument for each sample. For the library preparation, 6000 cells were targeted, of which 4,526 (CMFASW) and 5,711 (3XPBS) were successfully captured for sequencing. Libraries were then shipped to NIH and sequenced at the NIH Intramural Sequencing Center using the Illumina NovaSeq 6000_SP sequencing system with 28,91 base paired end reads targeting 300 million reads per library.

Single-cell analysis

The 10X Cell Ranger pipeline version 7.0.1 (Zheng et al. 2017) was used to pre-process the sequencing data for downstream analysis. The R package Seurat version 4.3.0 (Stuart et al. 2019) was used to generate clusters, find marker genes for each cluster, and further analyze the data. Overall, the CMFASW library was run on a single flow cell and had a total of ~350 million reads, with 92.3% mapped to the *H. symbiolongicarpus* genome, while the 3XPBS library was run on two flow cells and had ~720 million reads, with 84.4% mapped to the genome (Supplemental Table S31). We included the mitochondrial genome scaffold with annotated mitochondrial genes in our mapping step so any cells with mitochondrial reads > 5% could be filtered because this may indicate a cell is stressed or dying.

After processing each library independently, we found that their overall statistics (Supplemental Table S31) were similar and their contribution to the initial clustering was similar with both libraries contributing cells to each cluster (Supplemental Fig. S32). Only 337 genes were uniquely expressed in the CMFASW library, and only 1,166 genes were uniquely expressed in the 3XPBS library. Therefore, we combined both libraries using the following command in Seurat:

```
aggr <- merge(x=`3XPBS_filtered_feature_bc_matrix`,
              y=Seawater_filtered_feature_bc_matrix,
              add.cell.id = c("3XPBS", "Seawater"))
```

There were a total of 10,237 cells after combining the two libraries. We filtered the remaining cells using the following filters in Seurat: keep all cells with min.features = 100 and remove all cells that had > 5% mitochondrial reads. The combined library was then pre-processed using the standard workflow provided by the Seurat tutorial (https://satijalab.org/seurat/articles/pbmc3k_tutorial.html) with minor adjustments. The first version and visualization of our data can be seen in Supplemental Data S49, SA01, including documentation of the settings we used to create this cell atlas. The raw and final datasets are available at https://research.nhgri.nih.gov/hydractinia/download/index.cgi?dl=sd.

After exploring an initial clustering and beginning to annotate clusters as cell types, we noticed that mature sperm cell marker genes were present in small subclusters for all major clusters (Supplemental Data S49, SA02). These were likely artifacts from the dissociation where small, sticky sperm cells were presumably encapsulated with other cell types, forming 'sperm doublets.' After excluding the real sperm and developing sperm cell clusters (C0,1,4), these sperm doublet cells were filtered out of the remaining dataset by removing any cells that expressed a select few mature sperm cell marker genes (Supplemental Data S49, SA02). The chosen filtered genes were highly expressed in our putative mature sperm cell cluster C0, to confidently remove cells in other clusters that had characteristic mature sperm cell markers. We repeated this filtering process one more time to remove any remaining sperm doublets present in the data. In total, 1,349 cells were removed after these filtering steps. The final clustering with Seurat resulted in 18 clusters from an overall pool of 8,888 cells. Full documentation of the steps and code used to create the final version of our dataset are shown in Supplemental Data S49, SA03. The clusters were classified as putative cell types or cell states through the annotation of marker genes using comparisons with the *Hydra* single-cell dataset (Siebert et al. 2019) and literature searches. Supplemental Table

S32 shows the genes that were used to annotate clusters and associated publications. Supplemental Fig. S33 shows a heatmap of the top five marker genes per cluster. Supplemental Fig. S34 shows gene expression plots for cell type markers for several cell types.

Fixation and fluorescent in situ *hybridization (FISH)*

Animals were first anesthetized in 4% MgCl2 (in 50% distilled water / 50% filtered seawater). Samples were then fixed in 0.2% glutaraldehyde, 4% paraformaldehyde in filtered seawater (FSW) for about 90 seconds. A second fixation in ice-cold 4% paraformaldehyde in FSW containing 0.1% Tween20 was then performed, and samples left rocking at 4°C for 90 minutes. After fixation, samples were washed with ice-cold phosphate buffered saline (PBS) supplemented with 0.1% Tween20 (PBST) and were then dehydrated through a series of 25%, 50%, 75% and 100% methanol steps, with each step incubated for about 1-2 minutes. Samples were stored at -20°C. The ORFs of selected *Hydractinia* genes were cloned by PCR into pGEM-T vector for synthesis of *in situ* hybridization probes. Primer sequences for PCR cloning of genes to synthesize riboprobes can be found in Supplemental Table S30. Probes were synthesized with an Ambion MEGAscript kit (Cat #AM1334 for MEGAScript T7; Cat #AM1330 for MEGAScript SP6) following the manufacturer's guidelines. Samples were rehydrated following an inverted methanol steps series, finishing in PBST. Samples were then incubated in PBST at 85°C for 20 minutes, followed by five-minute washes with 1x triethylamine (TEA), 0.06% acetic acid in 1X TEA, and 0.12% acetic acid in 1X TEA. An equal volume of hybridization buffer (4M urea, 0.1 mg/ml yeast tRNA, 0.05 mg/ml Heparin, 5x SCC, 0.1% Tween20, 1% SDS) was then added to perform a two hours-long pre-hybridization step, at hybridization temperature (55°C). DIG-labeled probes were diluted to a concentration of 0.5-2 ng/ul in hybridization buffer and hybridization was done for about 40 hours at 55°C. Following hybridization, animals were washed once in hybridization buffer at 55°C for 40 minutes followed by a series of post-hybridization washes where first the hybridization buffer concentration, and then the SSC concentration, were reduced. Samples were then blocked for one hour in Roche Blocking Buffer diluted to $1/10^{th}$ in MAB (Maleic Acid Buffer, 100 mM maleic acid pH 7.5, 150 mM NaCl). Probes were detected using an Anti-DIG-POD antibody (Roche, Catalog# 11207733910), diluted at 1:500 in blocking solution. Incubations with antibodies were

carried out overnight at 4°C. For fluorescent reaction detection, samples were incubated in Tyramide development solution (2% Dextran sulfate, 0.0015% hydrogen peroxide, 0.2mg/ml Iodophenol, 1:100 Alexa Fluor 594 Tyramide (Thermo Scientific, Cat. #B40925) in PBST) for eight minutes, followed by several PBST washes. Nuclei were stained using Hoechst dye 33342, and samples were mounted in Fluoromount (Sigma-Aldrich) and imaged using a Zeiss LSM 710 confocal microscope. Images were processed in Fiji.

OrthoMarker analyses

A marker gene list for each cluster was created using Seurat and the settings used in the *Hydra* single-cell study (Siebert et al. 2019). Positive biomarkers for clusters in the whole data set clustering were identified using the Seurat function FindAllMarkers using min.pct = 0.25 and default parameters otherwise. Genes were filtered to exclude markers expressed in more than 5% of cells outside their cluster (pct2 < 0.05). This list was further filtered to exclude any duplicated markers and any markers that had a cluster differential expression adjusted p-value (p-adj value) cutoff of 10^{-200}. This p-adj value cutoff was chosen so that marker genes that had the most support in the dataset would be included (the analysis was repeated with a cutoff of 10^{-100} and the results were similar). The filtered marker list has 1,625 genes across 18 different clusters (C0 to C17) or the 7 major cell types (Supplemental Table S33). Markers are annotated with top BLASTp hits against nr and PANNZER2 gene IDs and GO terms. The clusters that were combined to comprise the 7 major cell types are detailed in Figure 6.

The OrthoFinder results (Supplemental Data S3-S9) were used to annotate the following levels of taxon-specificity to our *Hydractinia symbiolongicarpus* marker gene list using R and the 'dyplr' package (Wickham et al. 2022): These include: (1) unassigned markers (genes that did not cluster with any other genes), (2) "Other multispecies orthogroup" (markers found in at least one animal outside of the Phylum Cnidaria), (3) "Cnidarian-specific" (markers found in at least one cnidarian outside of the clade Medusozoa), (4) "Medusozoa-specific" (markers found in at least one medusozoan outside of the class of Hydrozoa), (5) "Hydrozoa-specific" (markers found in *Hydra vulgaris* or in both *H. vulgaris* and *Clytia hemisphaerica*), (6) "HydractinaClytia-specific

(markers found in *Clytia hemisphaerica* but not *H. vulgaris*), (7) "Hydractinia-specific" (markers found in both *H. echinata* and *H. symbiolongicarpus*), and (8) "Symbio-specific" (markers only found in *H. symbiolongicarpus*). These annotations are included in Supplemental Table S33. We created a dataframe that showed different perspectives of our data (e.g., whole genome gene models versus individual cluster markers versus putative cell type markers, etc.) and how they were sorted amongst the different taxon-specificity categories. This dataframe was used to create the bar plot in Fig. 7a and the histogram in Fig. 7b with the R package 'ggplot'.

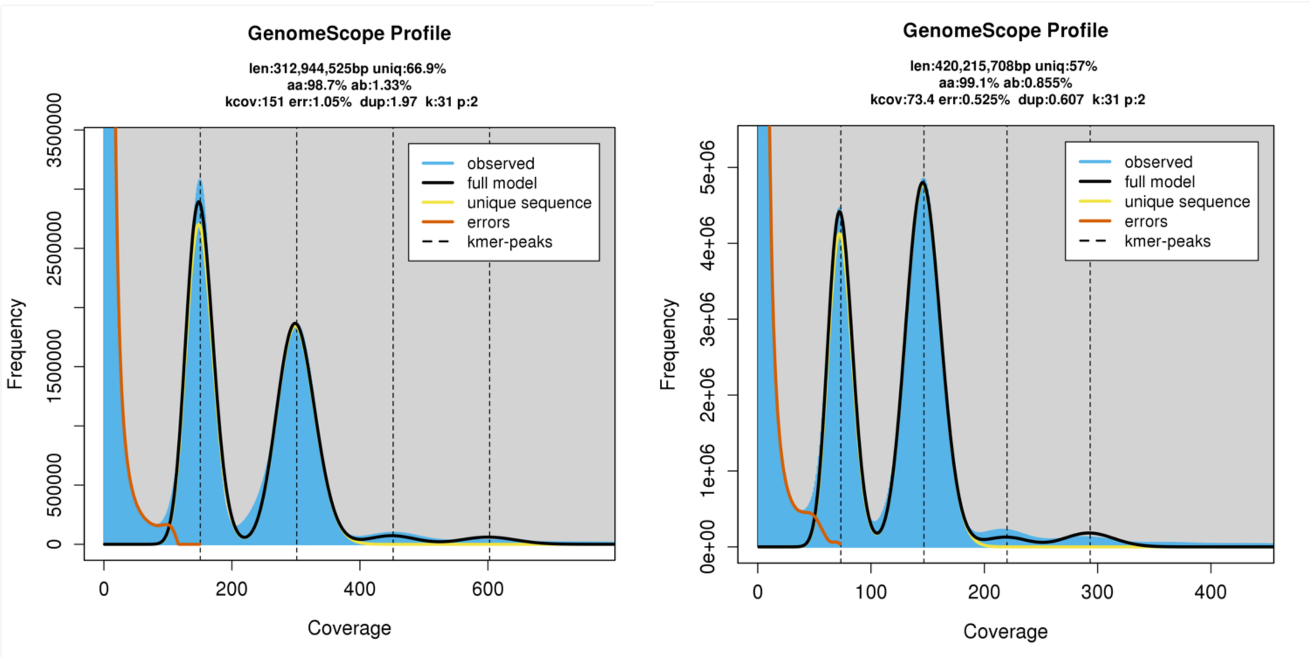

Figure S1: GenomeScope2.0 profiles for *H. symbiolongicarpus* (left panel) and *H. echinata* (right panel) using Illumina short read data and a k-mer value of 31. Note the two main peaks indicate these are diploid genomes. The estimated haploid genome sizes (312.9 Mb *H. symbiolongicarpus*, 420.2 Mb *H. echinata*) are much smaller than the propidium iodide-stained nuclei/FACS estimates (Supplemental Table S1). It is not unusual for GenomeScope2.0 to underestimate genome size. The estimated heterozygosity is 1.33% for *H. symbiolongicarpus* and 0.85% for *H. echinata*.

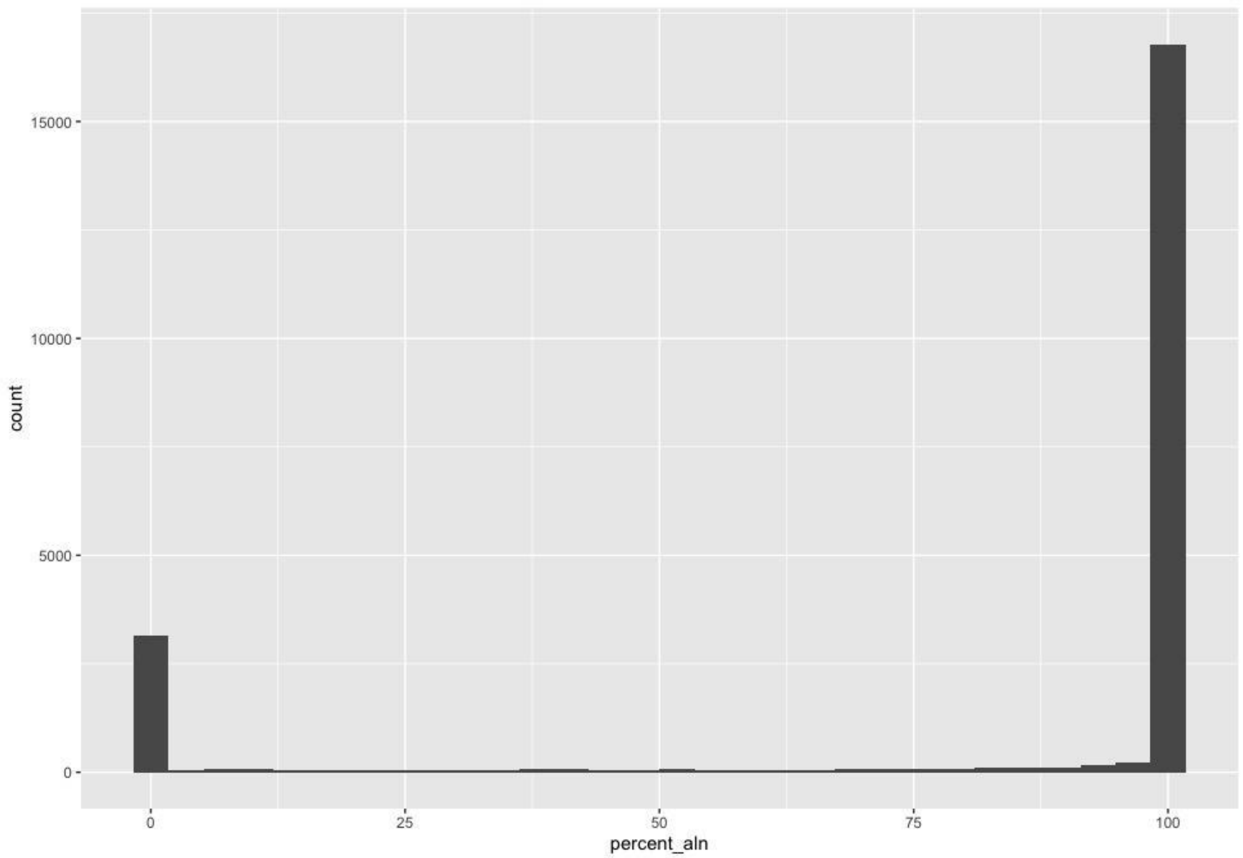

Figure S2: Combined transcript support for all *H. symbiolongicarpus* gene models. Support was bimodal with the majority of gene models receiving transcript support for the full length of the gene model while a minority of gene models had no support from aligned transcripts.

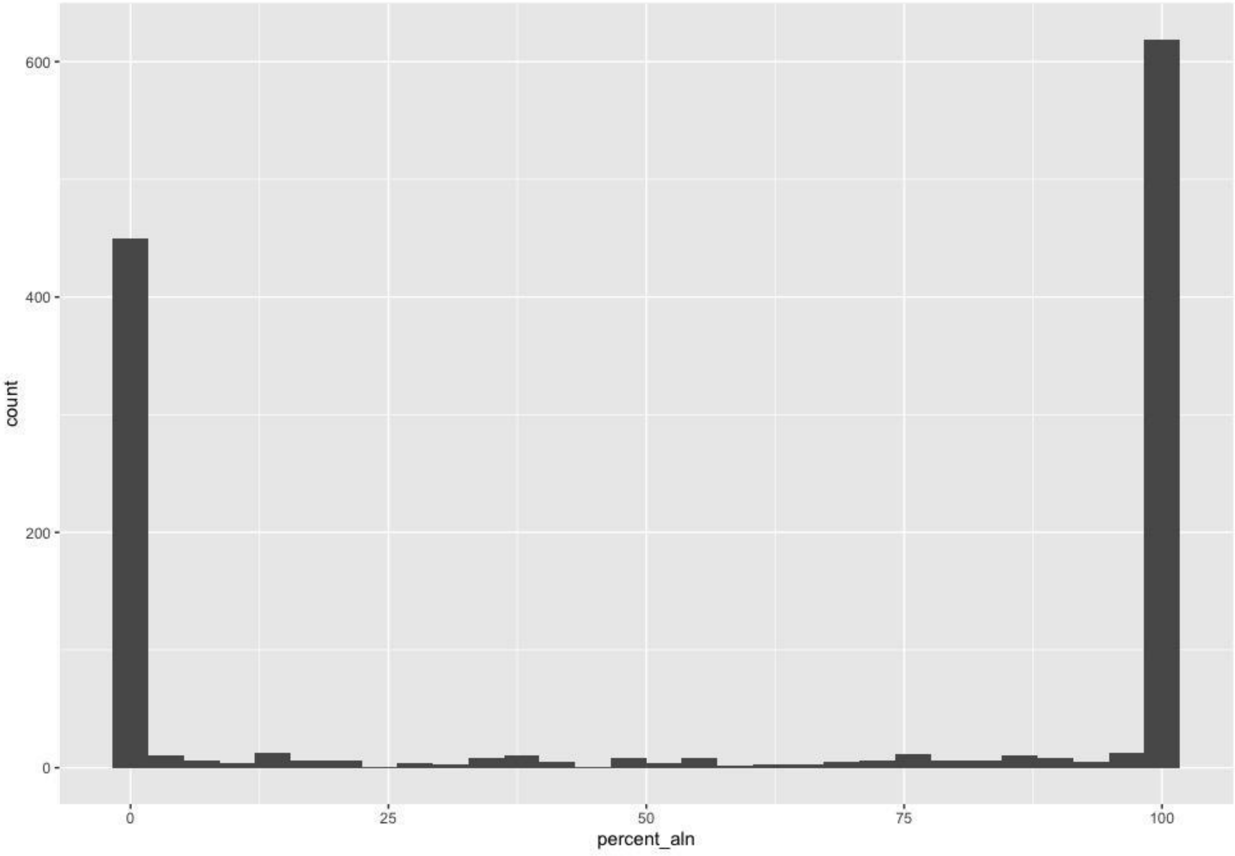

Figure S3: Combined transcript support for unassigned *H. symbiolongicarpus* gene models. Unassigned gene models refers to gene models that did not cluster with any other gene in our OrthoFinder results. Support was bimodal with many unassigned gene models receiving support for the full length of the gene model while the remaining unassigned gene models had no support from aligned transcripts.

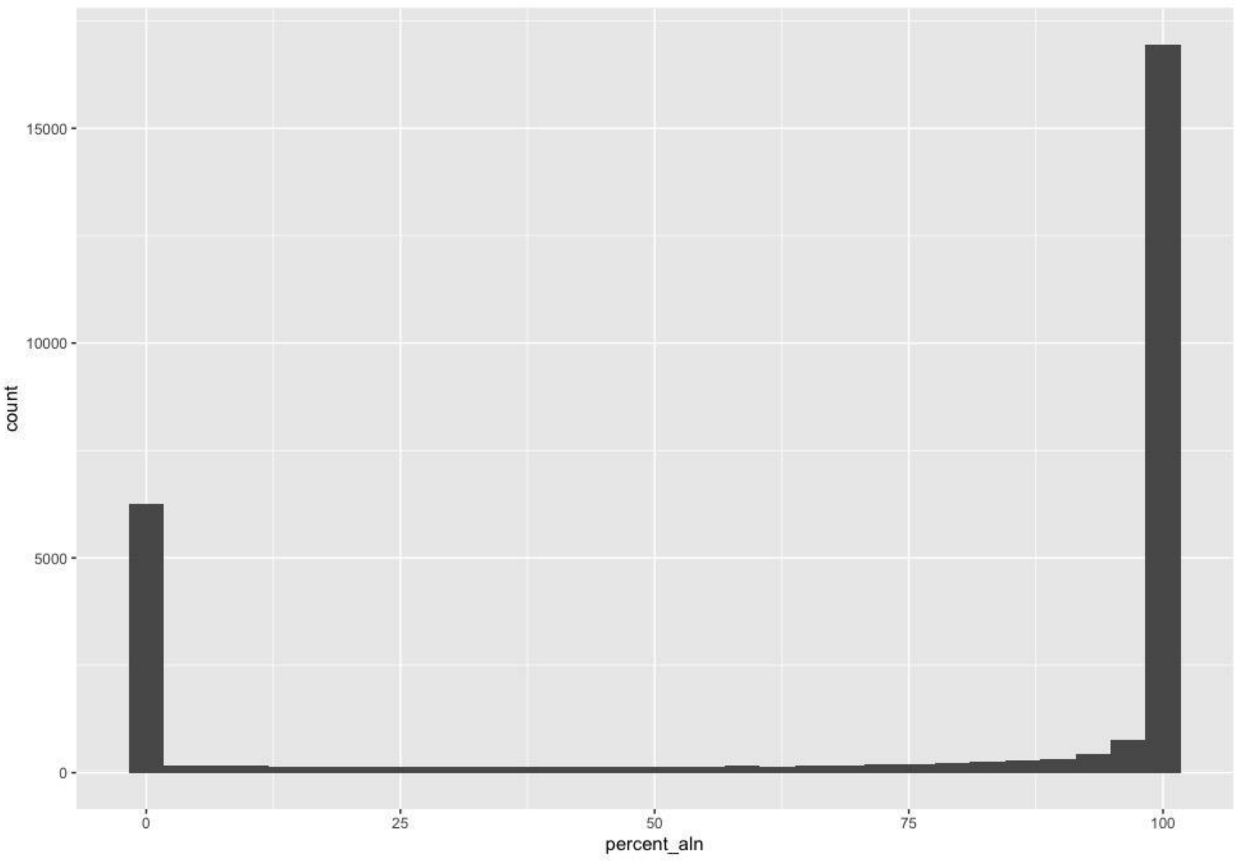

Figure S4: Combined transcript support for all *H. echinata* gene models. Support was bimodal with the majority of gene models receiving transcript support for the full length of the gene model while a minority of gene models had no support from aligned transcripts.

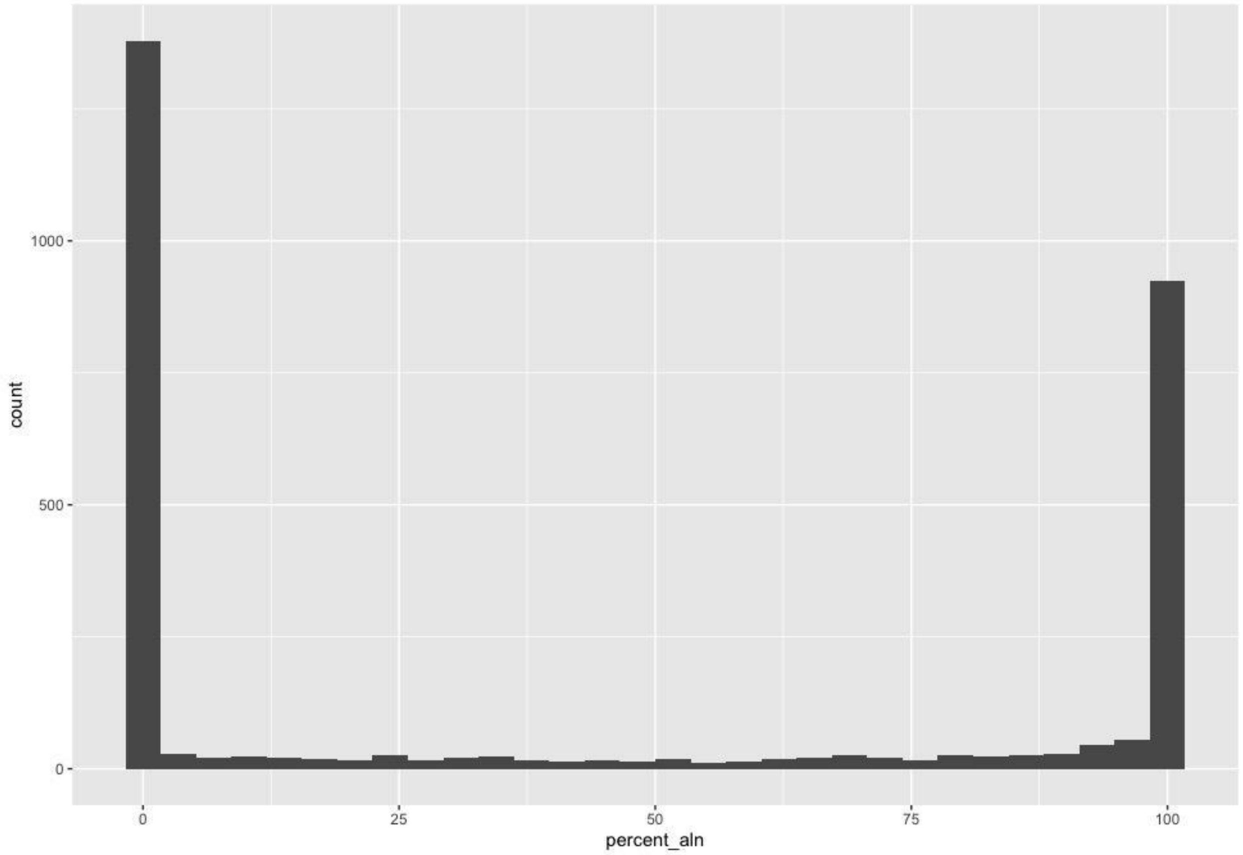

Figure S5: Combined transcript support for unassigned *H. echinata* gene models. Unassigned gene models refers to gene models that did not cluster with any other gene in our OrthoFinder results. Support was bimodal with some unassigned gene models receiving support for the full length of the gene model while several unassigned gene models had no support from aligned transcripts.

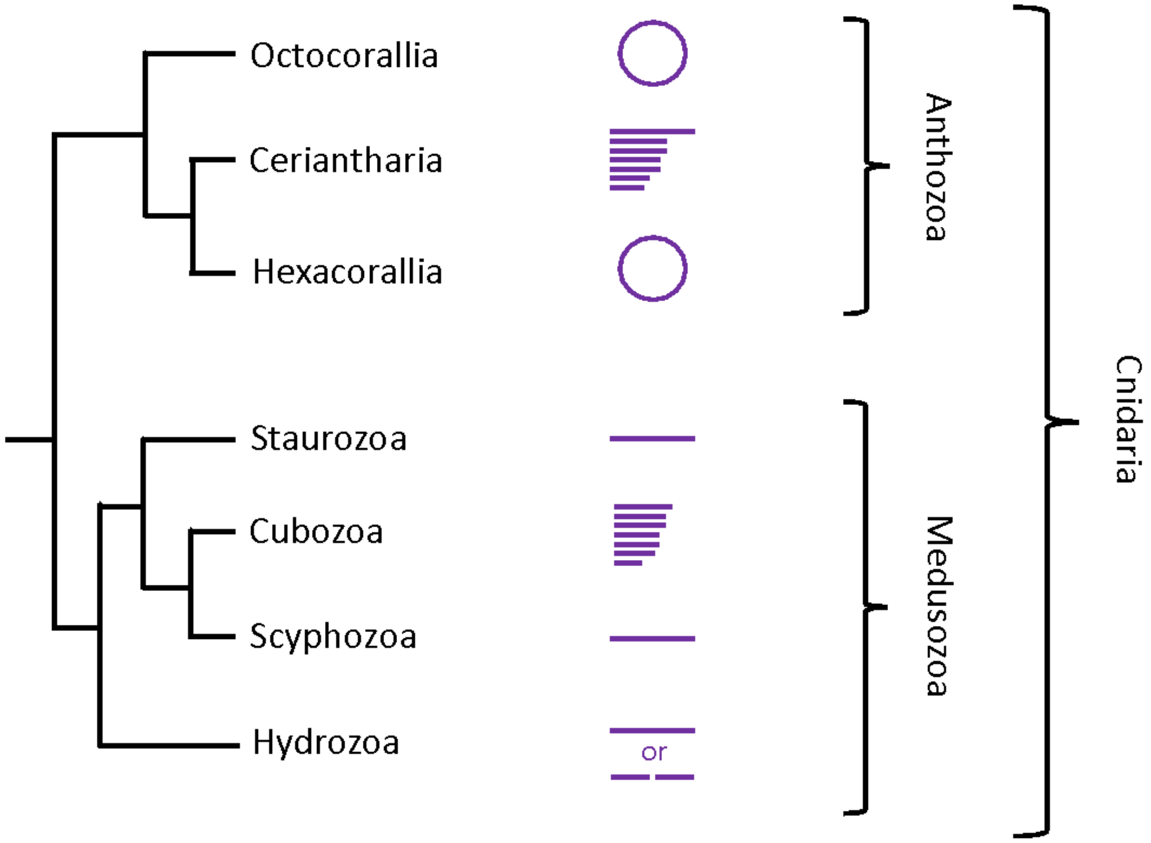

Figure S6. Mitochondrial architectures in Cnidaria. All medusozoans possess linear mono- or multimeric mtDNA. Anthozoa displays circular mitochondrial genomes, with the only two ceriantharian species studied to date possessing fragmented linear genomes.

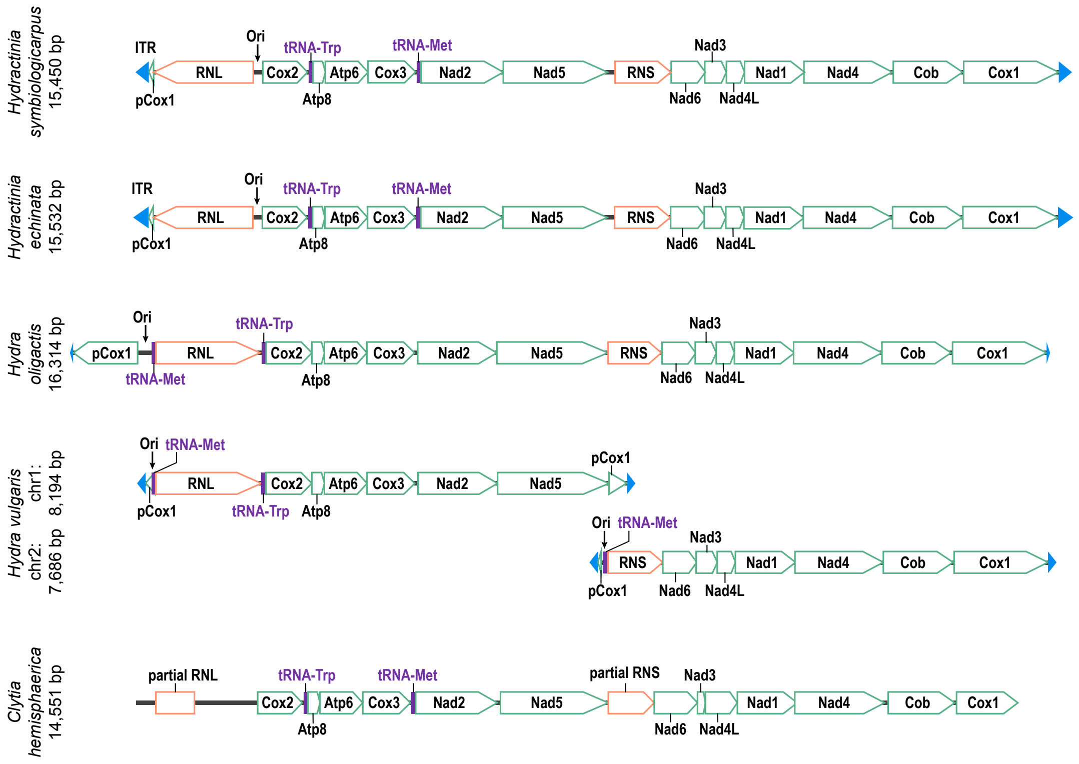

Figure S7: Mitochondrial organization in hydrozoans. All mtDNA chromosomes shown are contiguous sequences with no breaks within the chromosomes. Genes are color-coded as follows: green for proteins, orange for rRNAs, purple for tRNAs, and blue for inverted terminal repeats. Black arrows indicate origins of replication (Ori). The mitochondrial genomes displayed in this figure are aligned by their 16S RNA (RNL) sequences, with *H. vulgaris* chr2 aligned by its 12S RNA (RNS) sequence. Both *Hydractinia* species have an almost indistinguishable mtDNA architecture, with the size of their ITRs being the main contributor to the small difference in overall mtDNA length. The order of genes between the two rRNA sequences (RNL and RNS) is conserved in all species, although the tRNAs in the two Hydra species are located on either side of their RNL. *Hydra vulgaris* has a second tRNA-Met in chr2, upstream of its RNS sequence. In *Hydractinia*, the origin of replication is located between RNL and Cox2. In contrast, the origin of replication is located before the tRNA-Met sequence in both *Hydra* species.

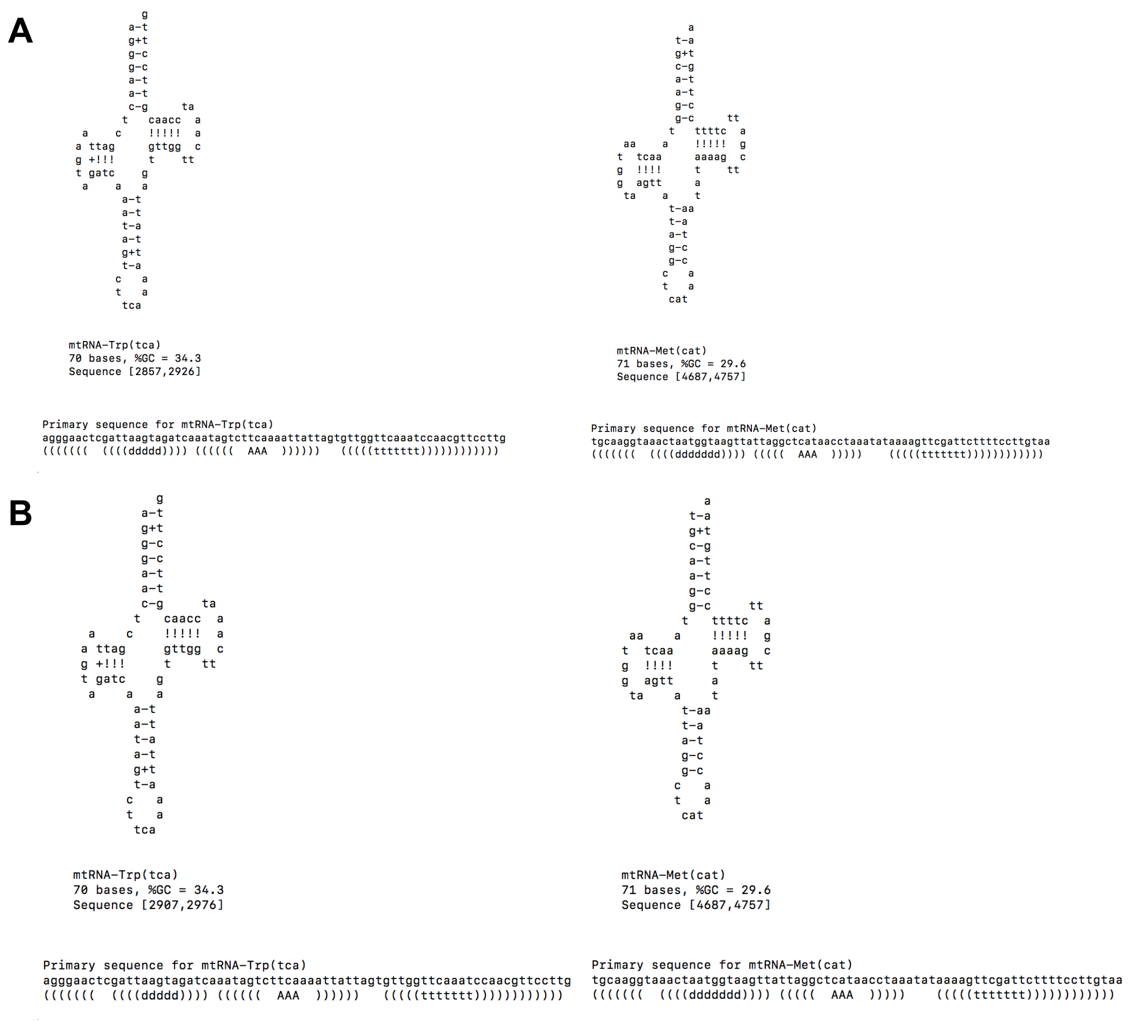

Figure S8: Predicted secondary structures of mitochondrial tRNA in *H. symbiolongicarpus* (A) and *H. echinata* (B). Both species contain a tRNA-Trp and a tRNA-Met. All tRNAs are located in non-coding regions.

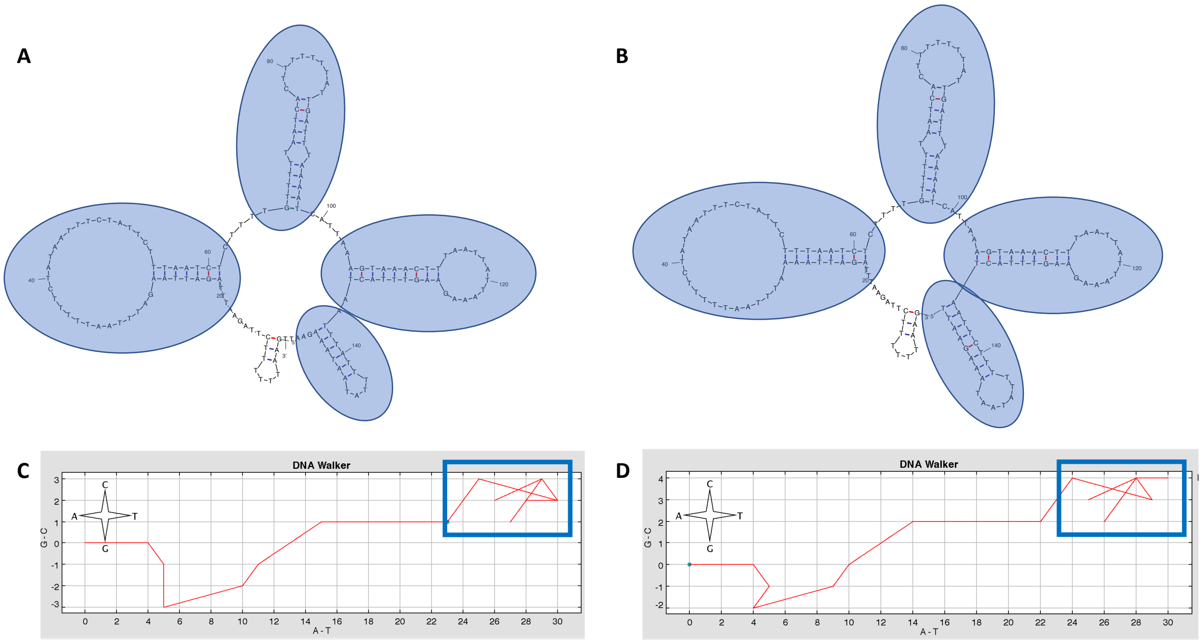

Figure S9: The origin of replication was identified in both *Hydractinia* species. The UNAFold analysis shows stem-loop configurations containing T-rich loops (blue ellipses) that are characteristic of origins of replication in both *H. symbiolongicarpus* (A) and *H. echinata* (B). Analyses using DNA Walker show abrupt changes in base composition bias that are also characteristic of origins of replication in both *H. symbiolongicarpus* (C) and *H. echinata* (D). The origin of replication is located between RNL and Cox2 genes in both species.

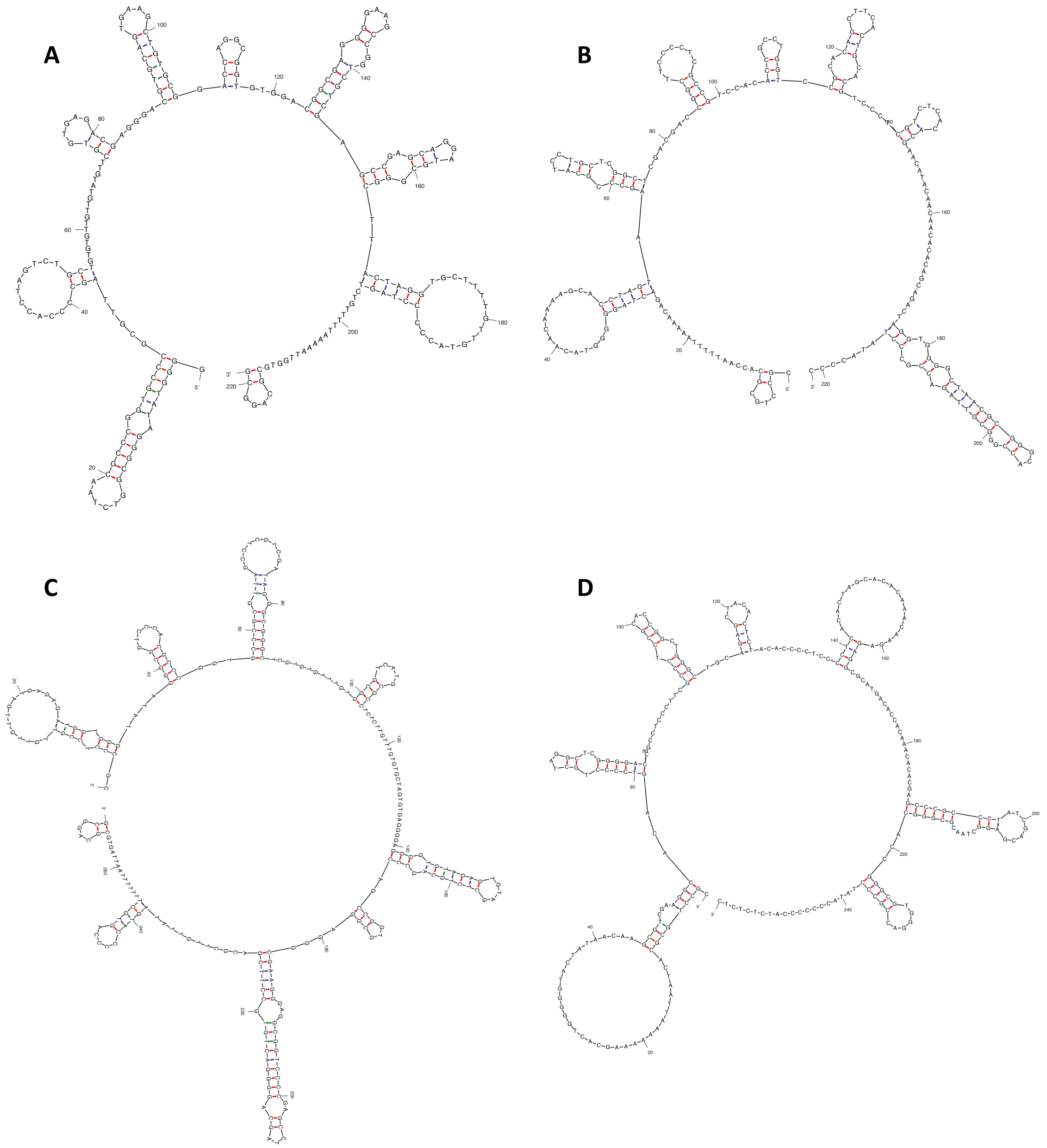

Figure S10: The mitochondrial genomes of both *Hydractinia* species contain inverted terminal repeats at each end of the chromosome that can act as telomeres. These repeats display G-rich loops (*H. symbiolongicarpus*: A - 5’ ITR and B - 3’ ITR; *H. echinata*: C - 5’ ITR; D - 3’ ITR) that help protect and prevent the deterioration of the mtDNA molecule.

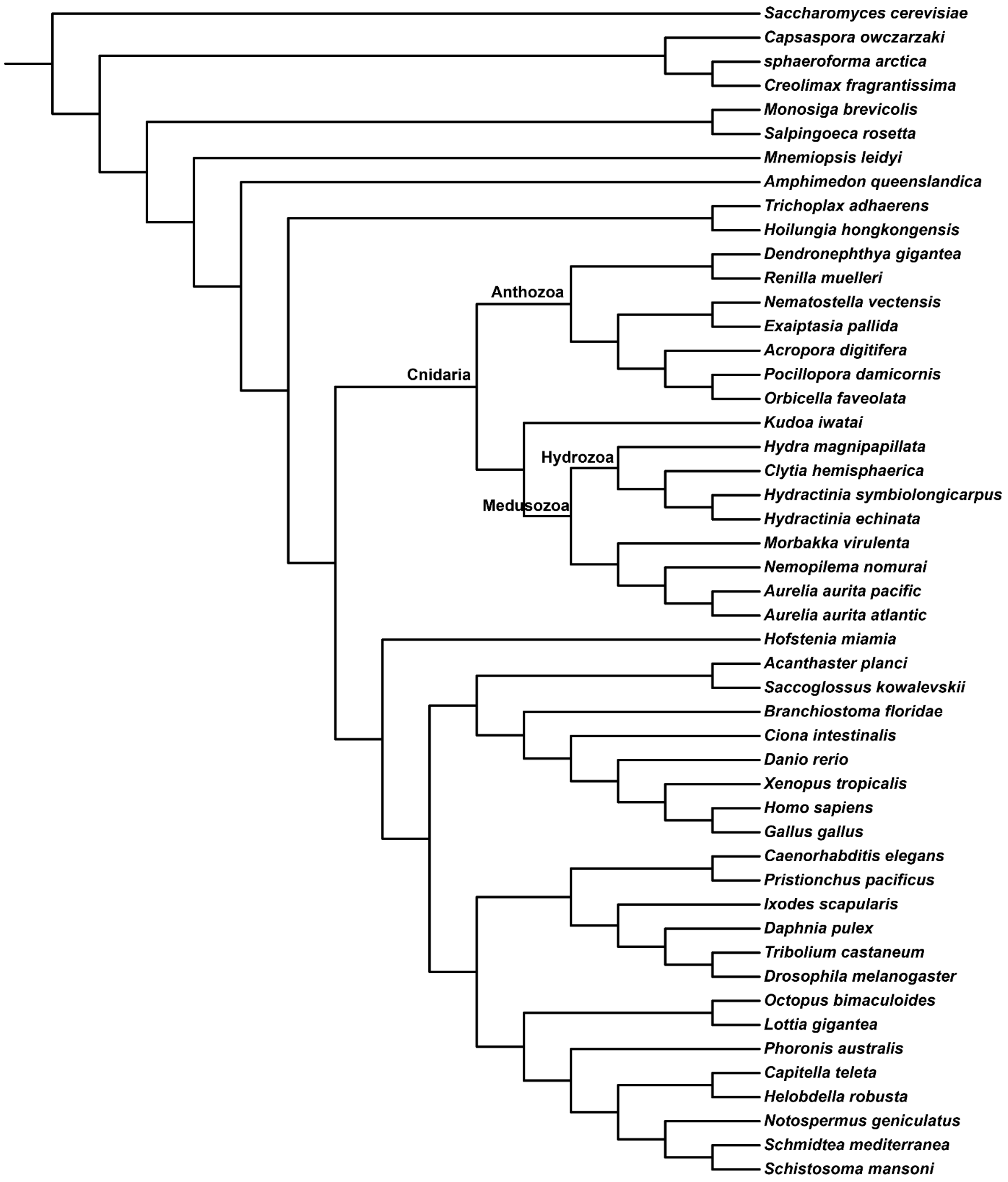

Figure S11: Input species tree used for OrthoFinder2 analysis. An input species tree with 49 species of animals and eukaryotic outgroups including 16 cnidarian species. Note that *Hydra magnipapillata* is the former name for *Hydra vulgaris*.

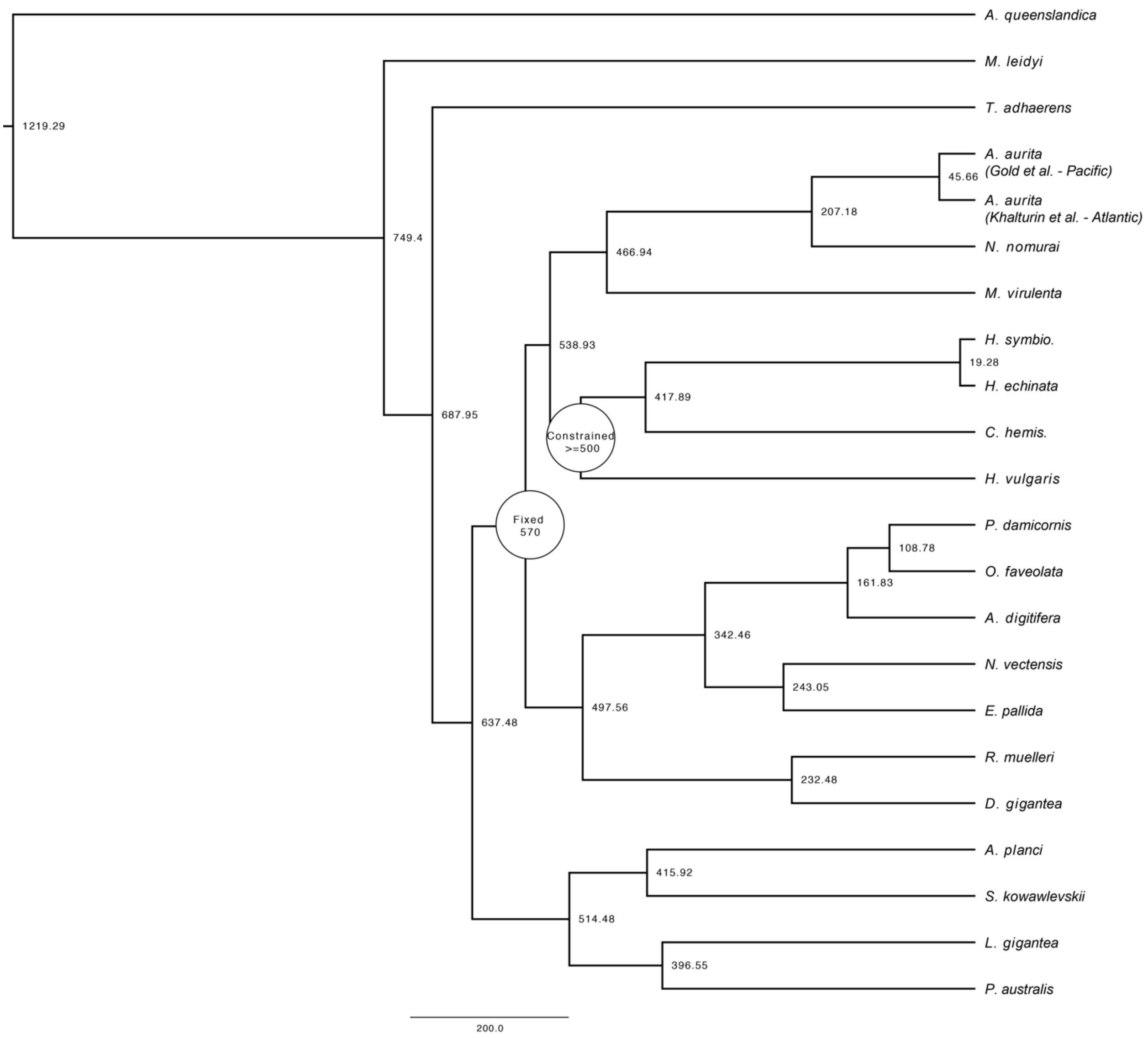

Figure S12: Divergence time estimates (MYA) for focal taxa with Porifera as outgroup. Divergence times were estimated using the r8s program on the RaxML topology from S1, except with the position of *A. queenslandica* and *M. leidyi* switched to test the effect of the branching order of Ctenophora and Porifera on our estimates. The age of Cnidaria is fixed and the age of Hydrozoa constrained based upon (Cartwright and Collins, 2007).

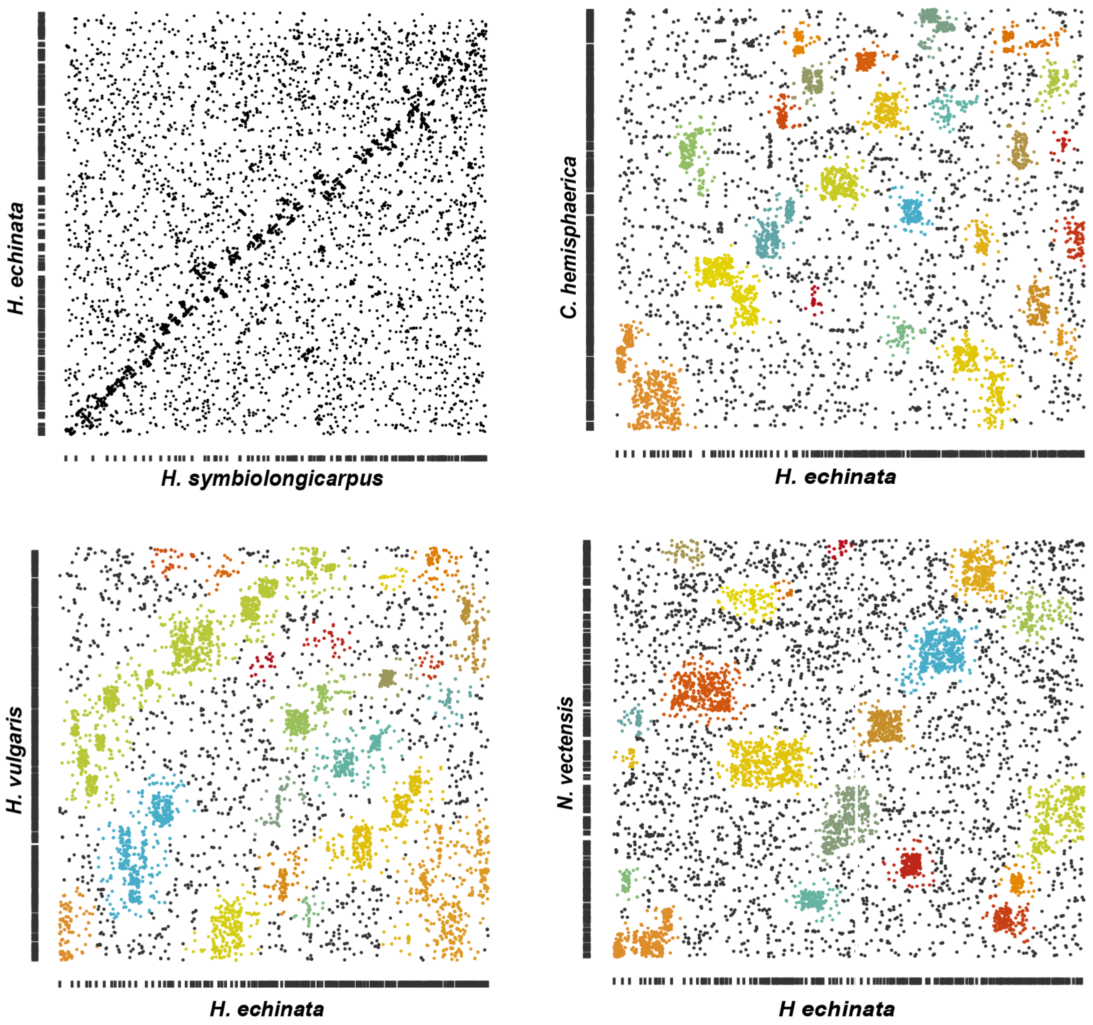

Figure S13: Syntenic dot plots comparing *H. echinata* with four cnidarian species: *H. symbiolongicarpus*; *Clytia hemisphaerica; Hydra vulgaris*; and *Nematostella vectensis*. The genomic scaffolds for each species were ordered according to hierarchical clustering and the matrix was further ordered by density-based spatial clustering. The clusters were colored by the group label and the data are displayed in pairwise syntenic dot plots.

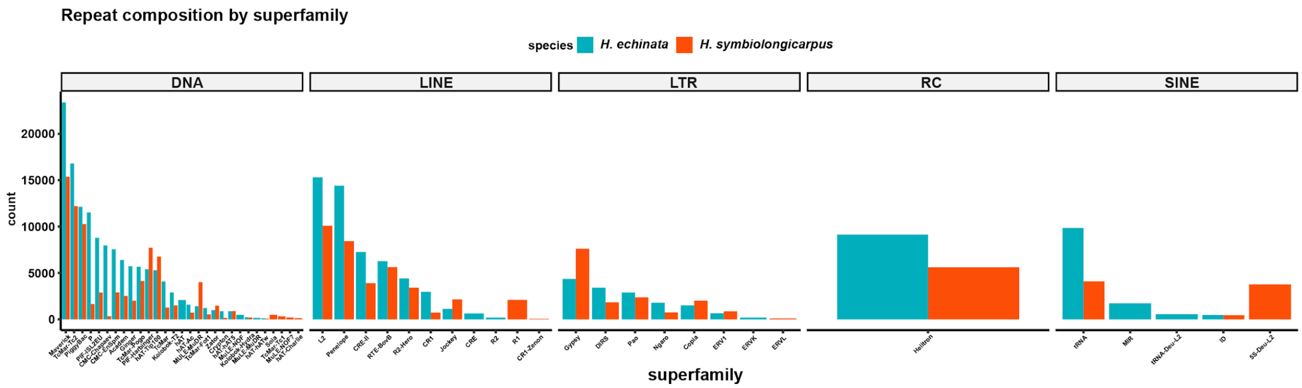

Figure S14: TE composition by superfamily groups in two species of *Hydractinia*. There are quite different compositions of some DNA transposon superfamilies between the two species of *Hydractinia*. Differences were less dramatic in other repeat classes and their superfamilies.

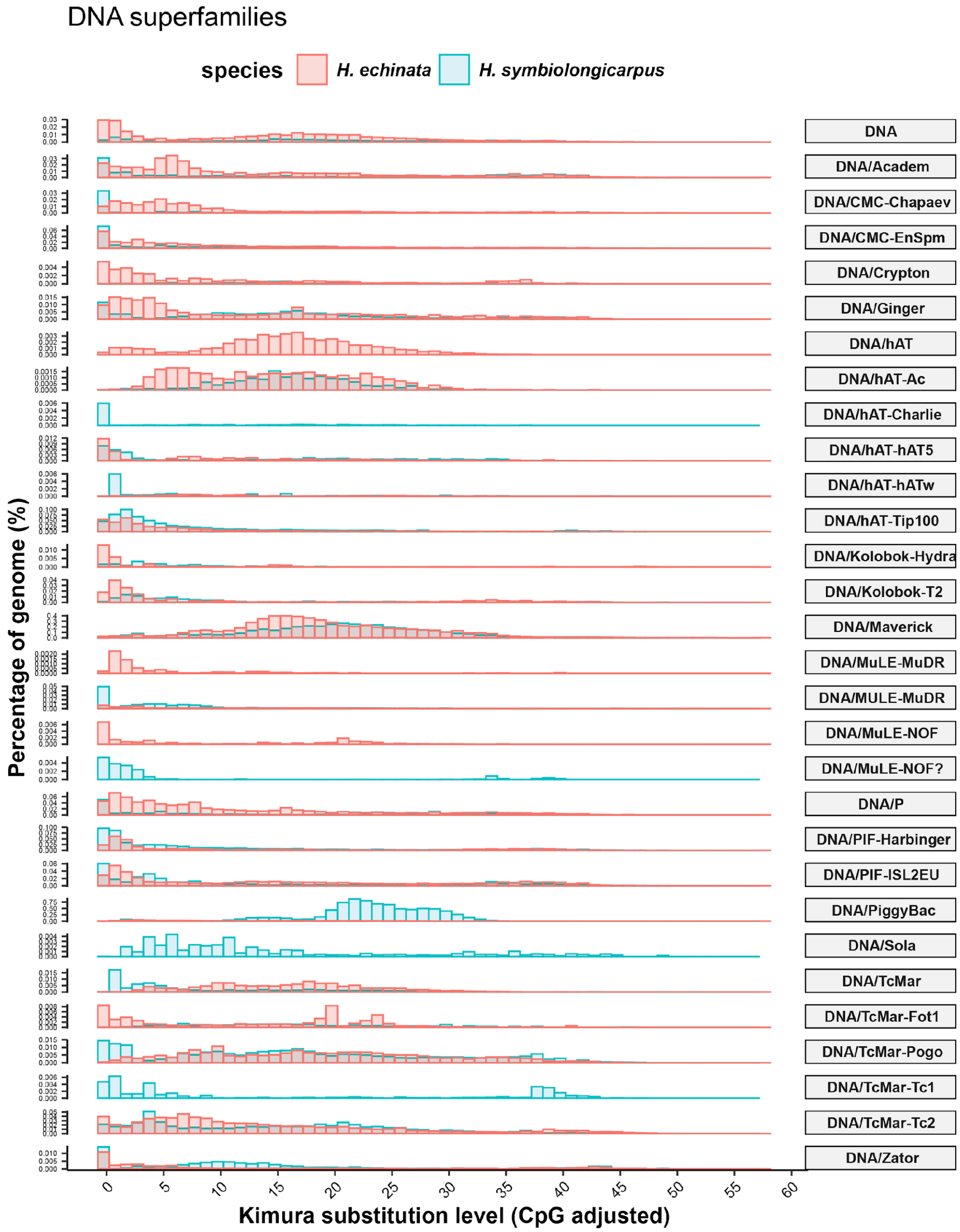

Figure S15: Age estimation for DNA transposon superfamilies for two species of *Hydractinia*. Some DNA superfamilies are exclusive to *H. symbiolongicarpus* like DNA/Sola or nearly exclusive like DNA/PiggyBac. Other TE superfamilies seem to have recently expanded in *H. symbiolongicarpus*, like DNA/hAT-Charlie. Superfamilies DNA/MuLE-NOF for *H. echinata* and DNA/MuLE-NOF? for *H. symbiolongicarpus* are likely similar structures but classified differently.

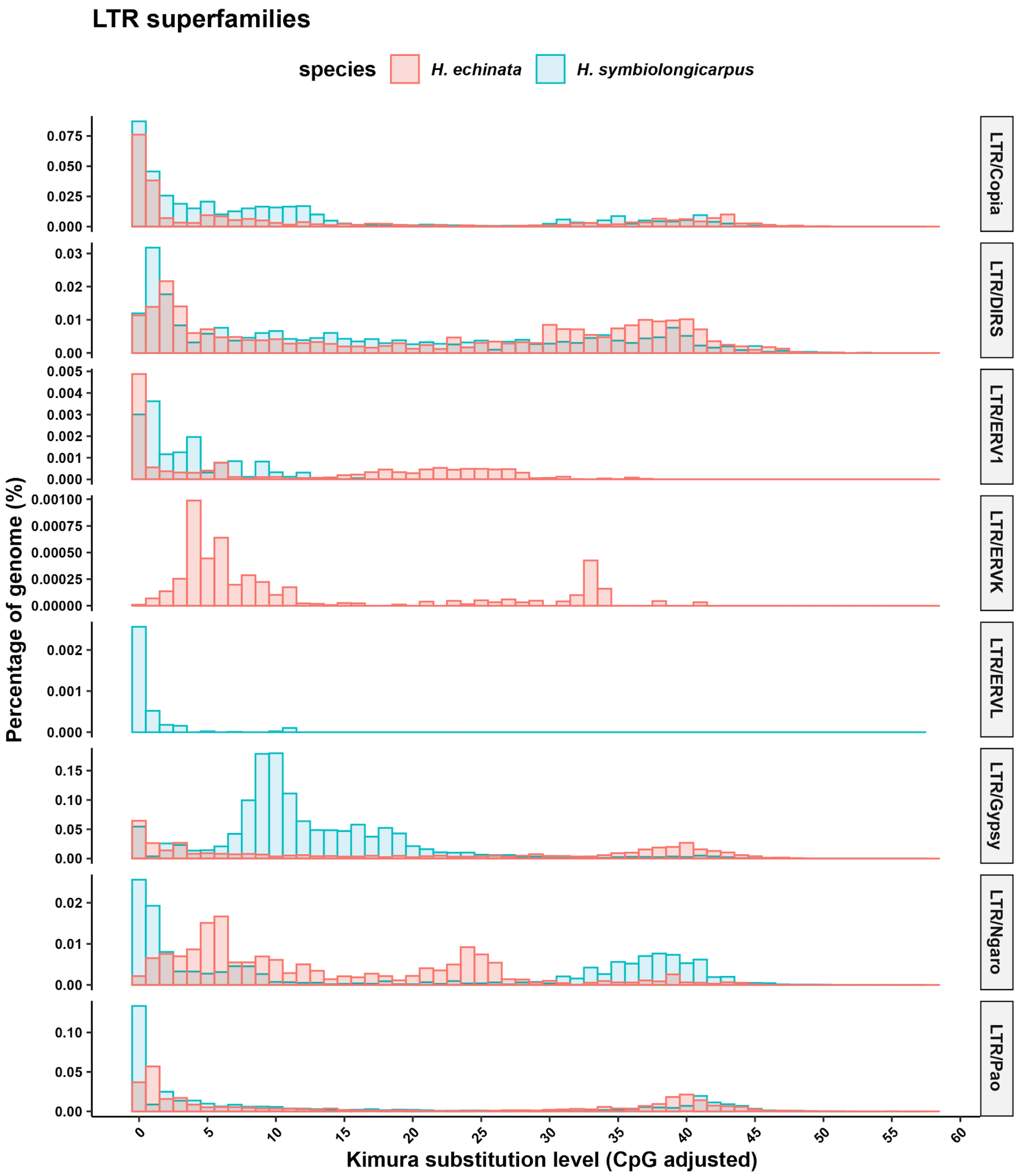

Figure S16: Age estimation for LTR transposon superfamilies for two species of *Hydractinia*. Some LTR superfamilies are exclusive to *H. echinata* like LTR/ERVK, while some are exclusive to *H. symbiolongicarpus* like LTR/ERVL. Some LTR superfamilies have a very different profile between the two species, like LTR/Gypsy, while others have more overlapping profiles.

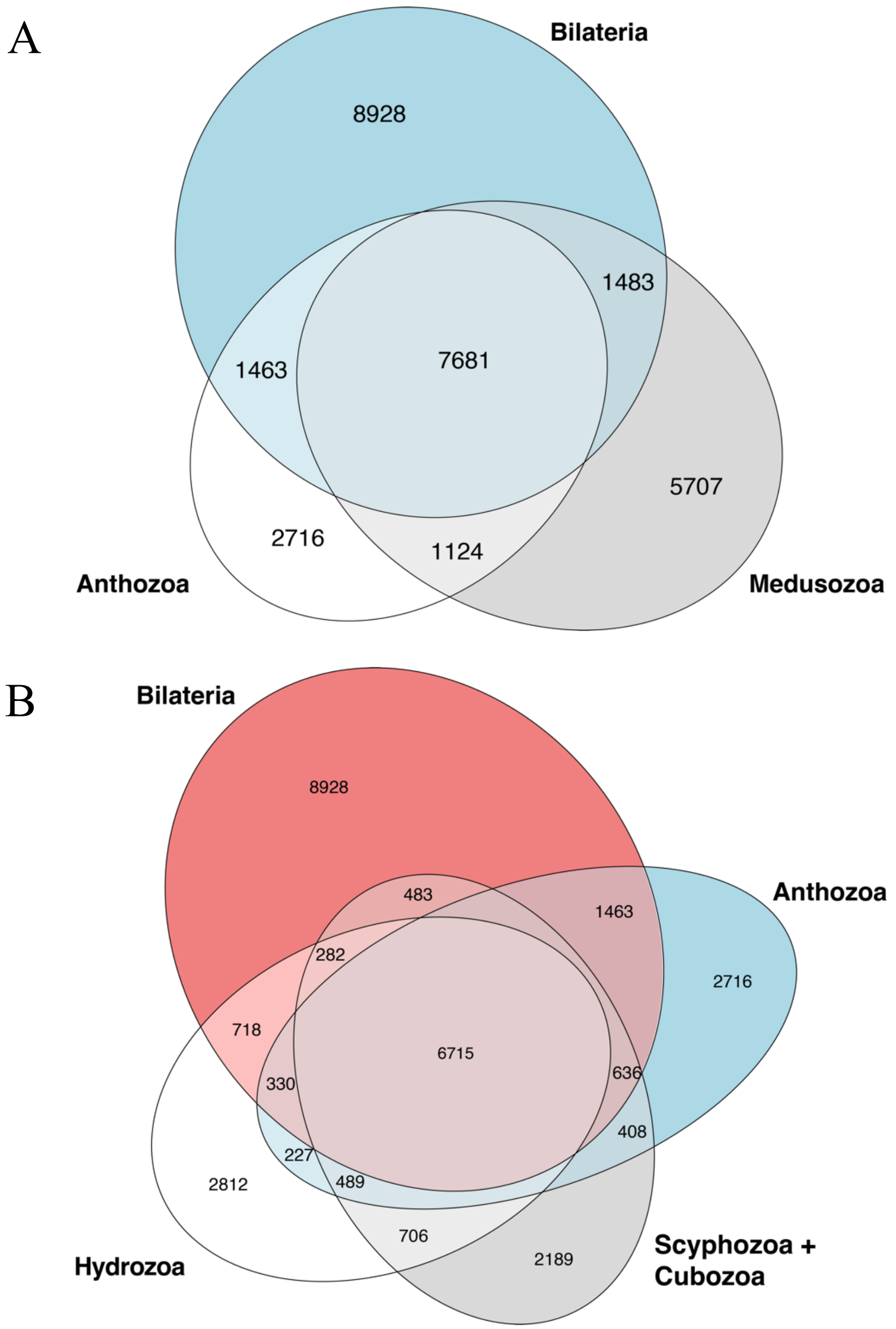

Figure S17A-B. Proportional Euler diagrams showing overlap between the sets of orthologous genes present in major cnidarian lineages and Bilateria. The numbers of ortholog groups were inferred using OrthoFinder, treating each orthogroup as either being present or absent. For each taxonomic group, an orthogroup was classified as present if it was found in at least one of the constituent species.

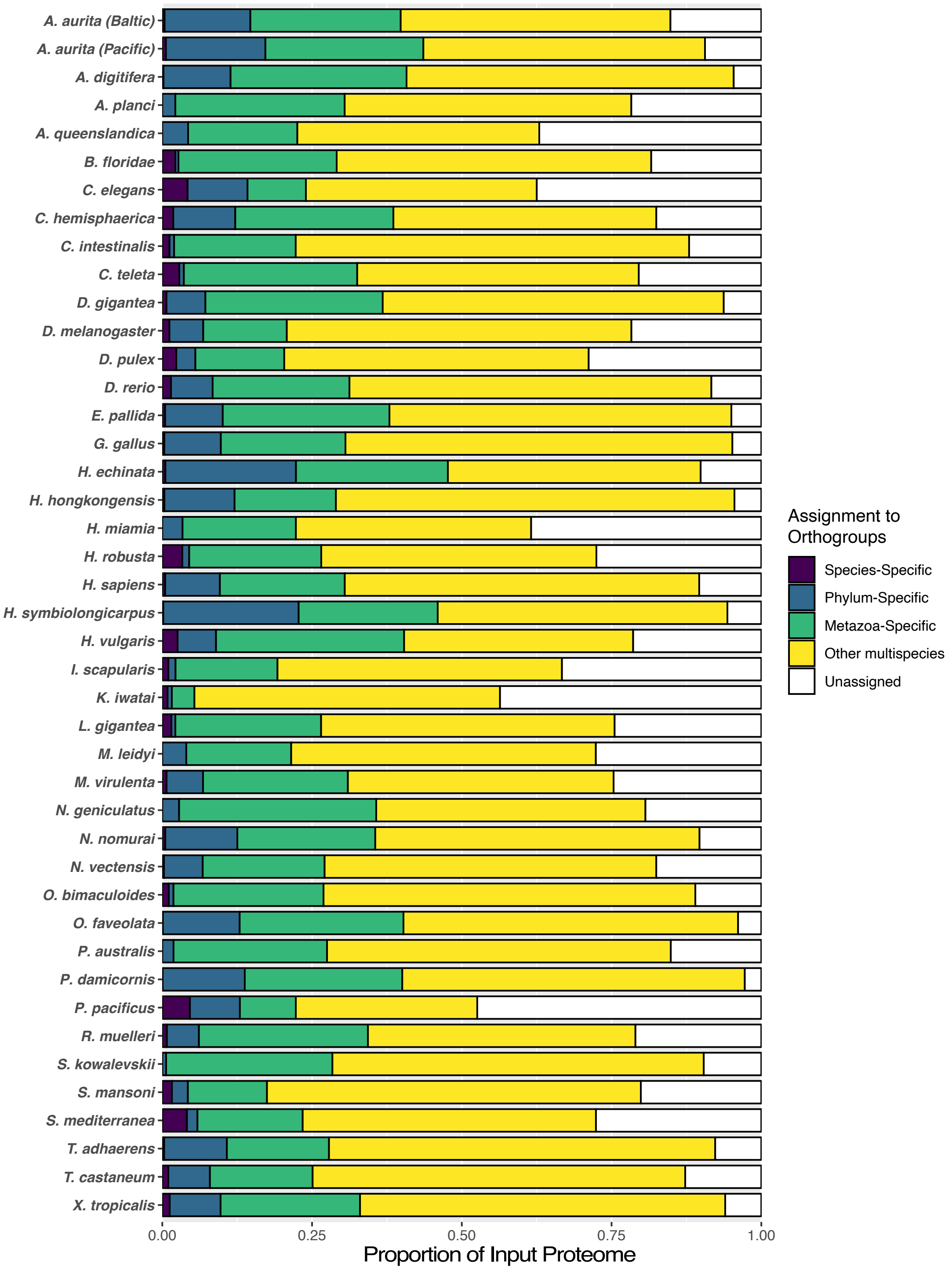

Figure S18. Summary of orthogroup assignments for all 43 metazoans. Proportion of input proteome sequences assigned to different orthogroup categories by OrthoFinder to different orthogroup categories for all metazoans included in our orthogroup inference. Number of input sequences for all proteomes including non-metazoan outgroups can be found in Supplemental Table S11.

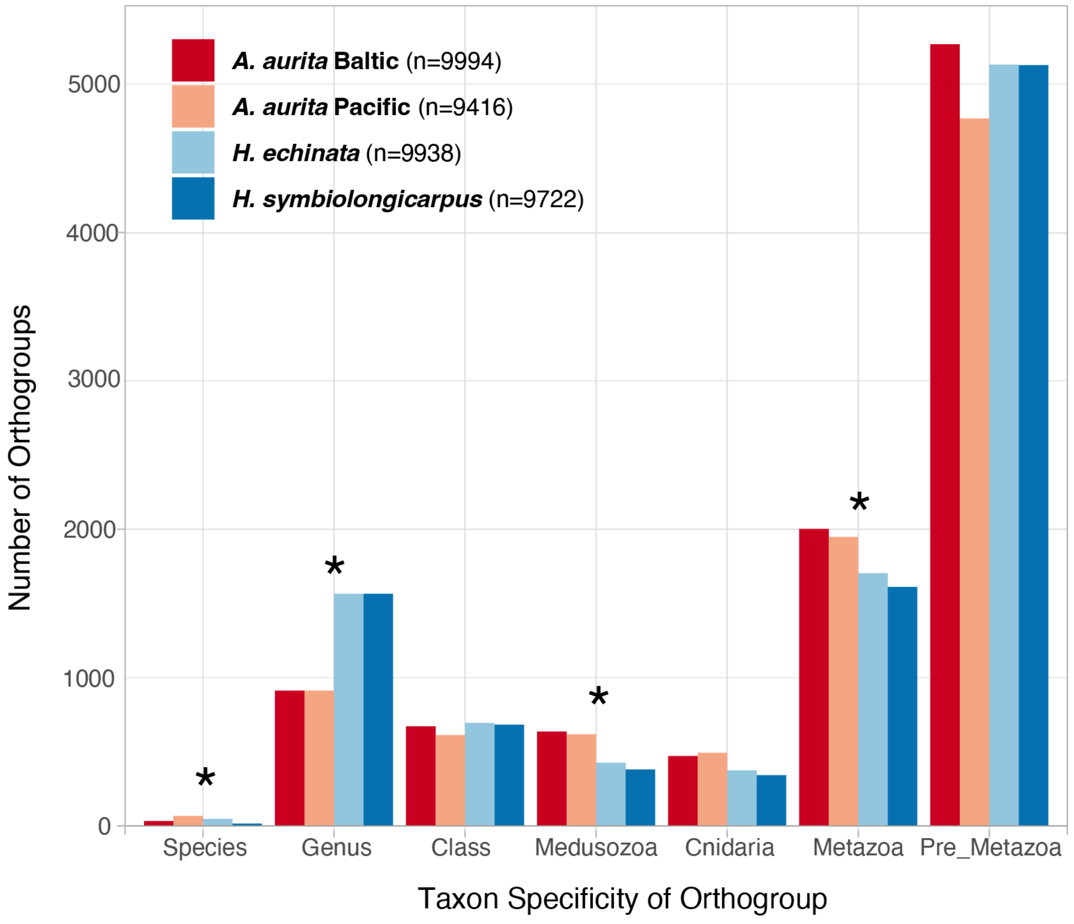

Figure S19. Distribution of orthogroup specificity for *Hydractinia* and *Aurelia.* Estimated evolutionary age of each orthogroup containing at least one sequence from the input proteome of a given species. For *Hydractinia* species, Class encapsulates taxonomic taxon-specificity between Genus-specific and Hydrozoa-specific, and for *Aurelia* species, Class includes levels between Genus-specific and Cubozoa+Scyphozoa-specific. Starred categories had significant differences amongst species in post-hoc comparisons made after overall significant Chi-square test.

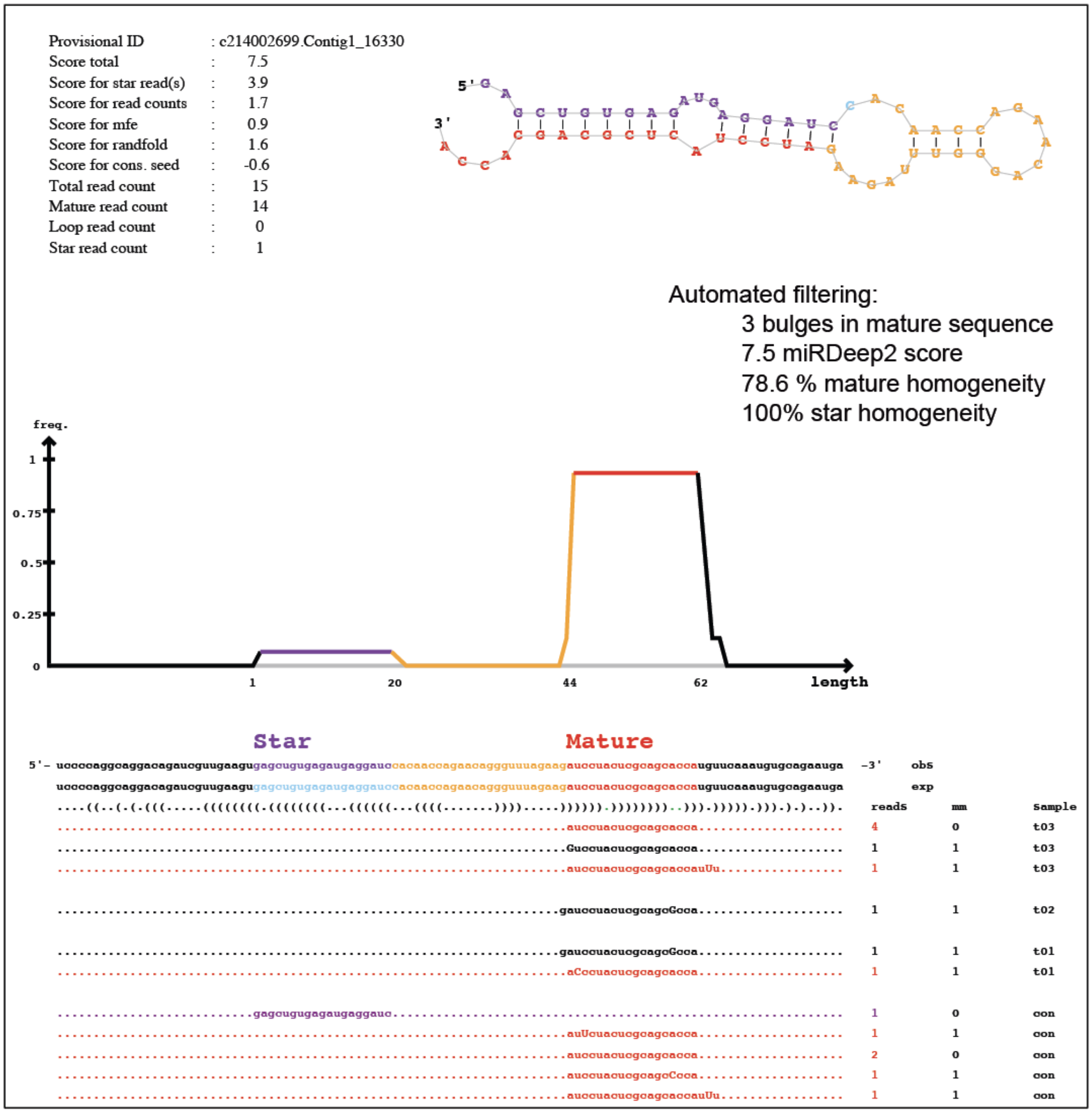

Figure S20: An example showing the automated filtering process for computationally predicted miRNAs. We used the following filtering criteria: (1) must contain at least one bulge found in the secondary structure (2) must have a miRDeep2 score above 5 (3) must have over 50% sequence homogeneity on the mature 5’ end (4) must have over 50% sequence homogeneity on the star 5’ end. Secondary structures are represented by dots (unpaired nucleotides), brackets (paired nucleotides), and dashes (alignment gaps). Unlike siRNAs, miRNAs have bulges in their mature sequence. The red sequences are mature reads with homogeneous 5’ ends and the purple sequences are star reads with homogenous 5’ ends.

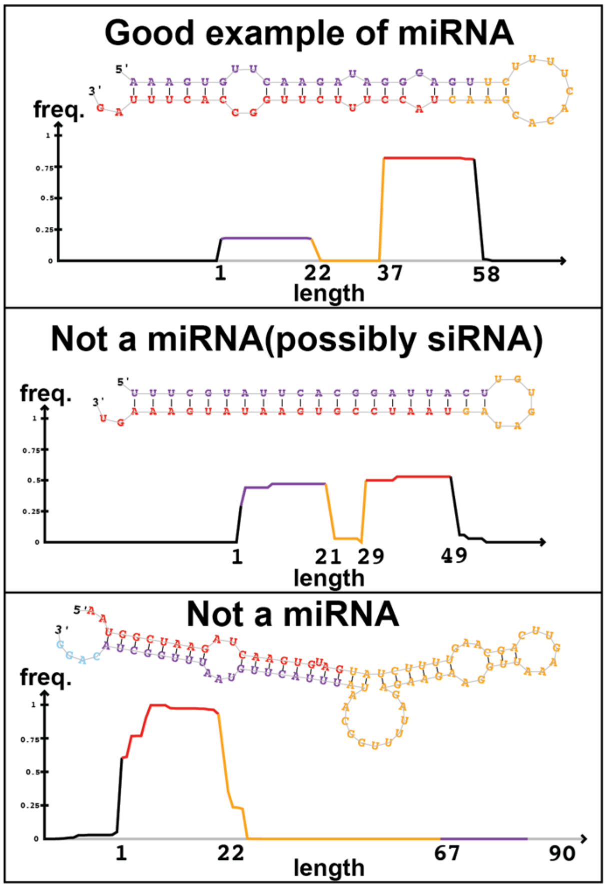

Figure S21. Manual screening of predicted miRNAs. Following automated filtering, we manually screened the predicted miRNAs to remove spurious predictions. Even with the automated filtering steps, some siRNAs and oddly shaped pre-miRNA structures were found and discarded from the final set of predictions. Here we show an example of a miRNA that passed our manual screening (top panel), and two examples of predictions that did not pass our manual screening including a potential siRNA (middle panel) and an oddly shaped pre-miRNA (bottom panel).

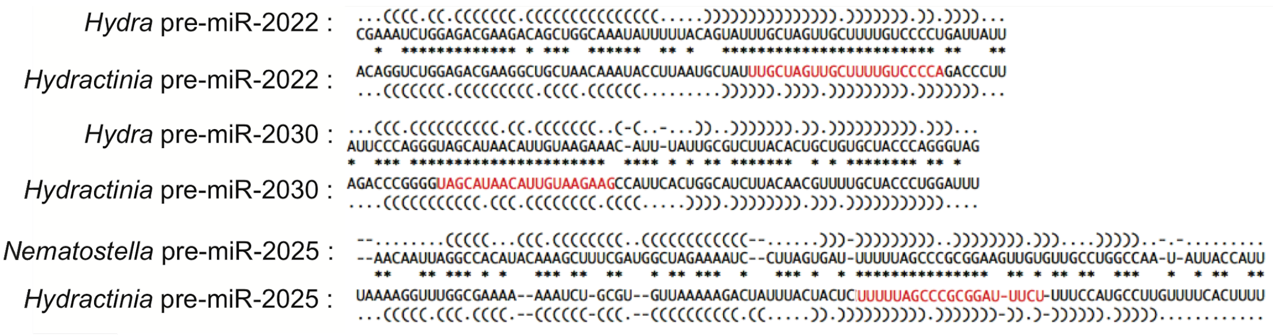

Figure S22. Sequence and secondary structure alignments of cnidarian pre-miR-2022, pre-miR-2030, pre-miR-2025. Mature miRNA sequences are shown in red. Conserved nucleotides are shown by asterisks. Secondary structures are represented respectively by dots (unpaired nucleotides), brackets (paired nucleotides), and dashes (alignment gaps). The alignment was calculated using T-Coffee (Notredame et al., 2000).

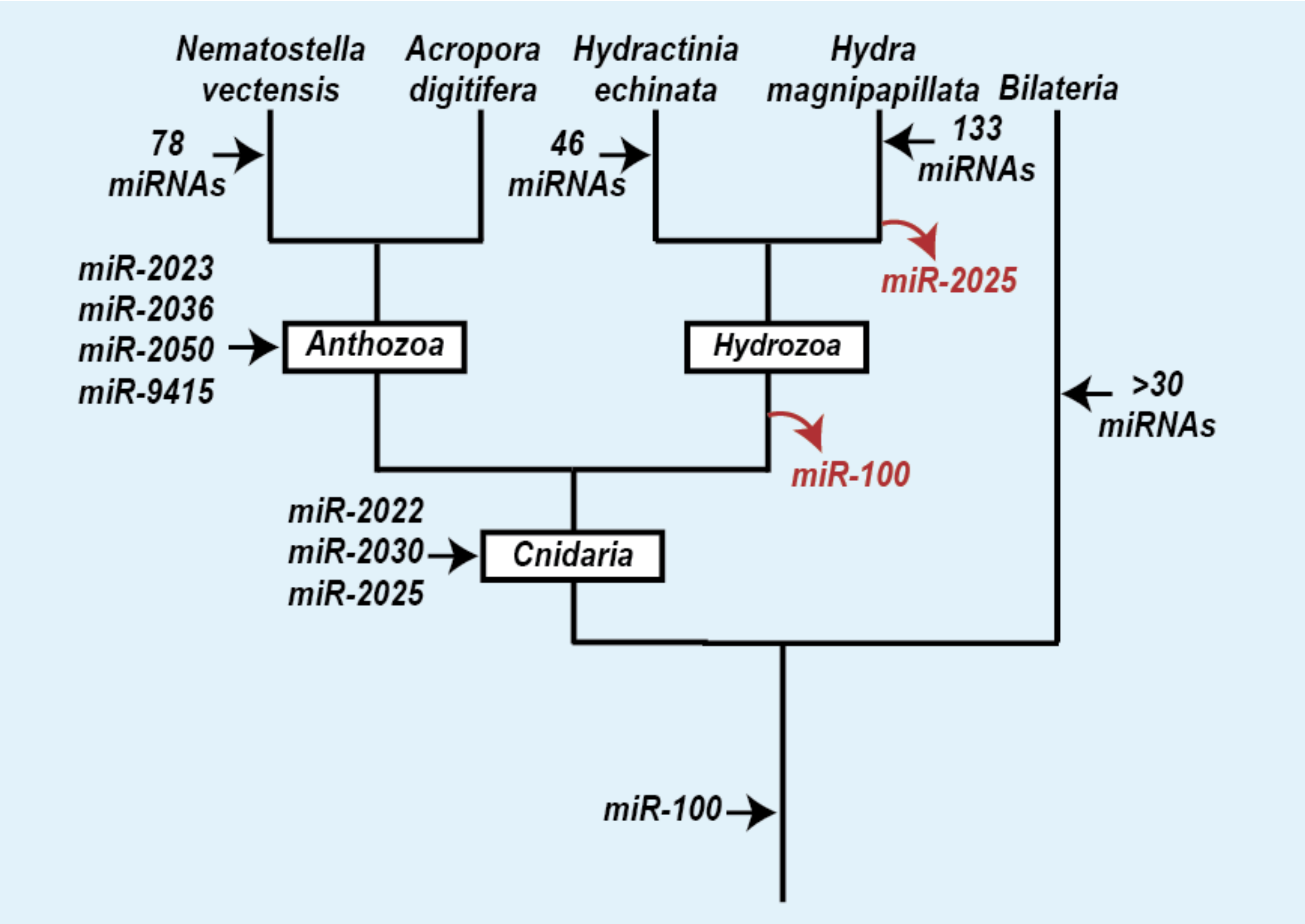

Figure S23. Proposed evolutionary scenario for miRNAs with a focus on gains and losses in cnidarians. *H. echinata* appears to have gained at least 46 miRNAs.

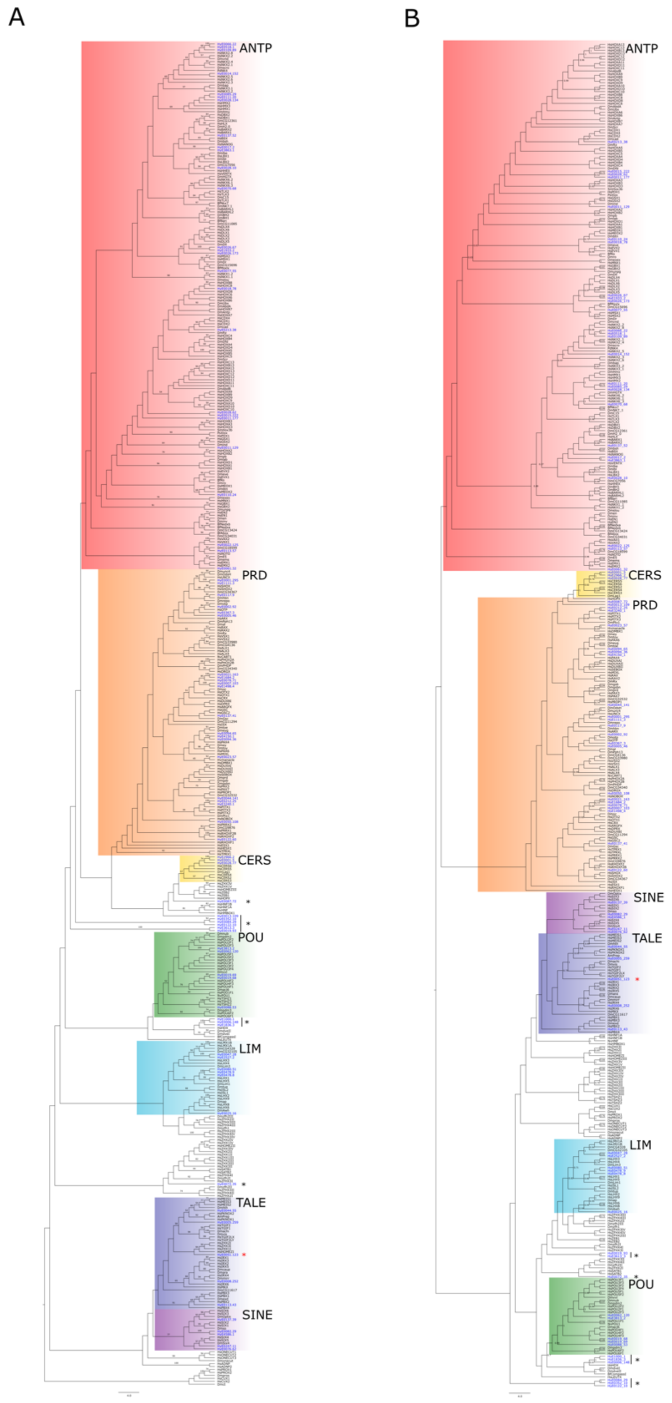

Figure S24. Homeodomain superfamily tree for *Hydractinia echinata*. A. Maximum likelihood tree of 82 *Hydractinia echinata* homeodomain proteins. Trees are midpoint rooted in cladogram format for display

purposes. Bootstraps > 50 are displayed. B. Bayesian phylogenetic tree of *Hydractinia echinata* homeodomain proteins. Trees are midpoint rooted in cladogram format for display purposes. Posterior probabilities > 70 are displayed. In both trees, *H. echinata* sequences are highlighted in blue text. Black stars represent unclassified sequences. The red star indicates sequence HyE0051.123 within the TALE class that was subsequently defined as a SINE class protein due to the presence of a secondary SIX domain.

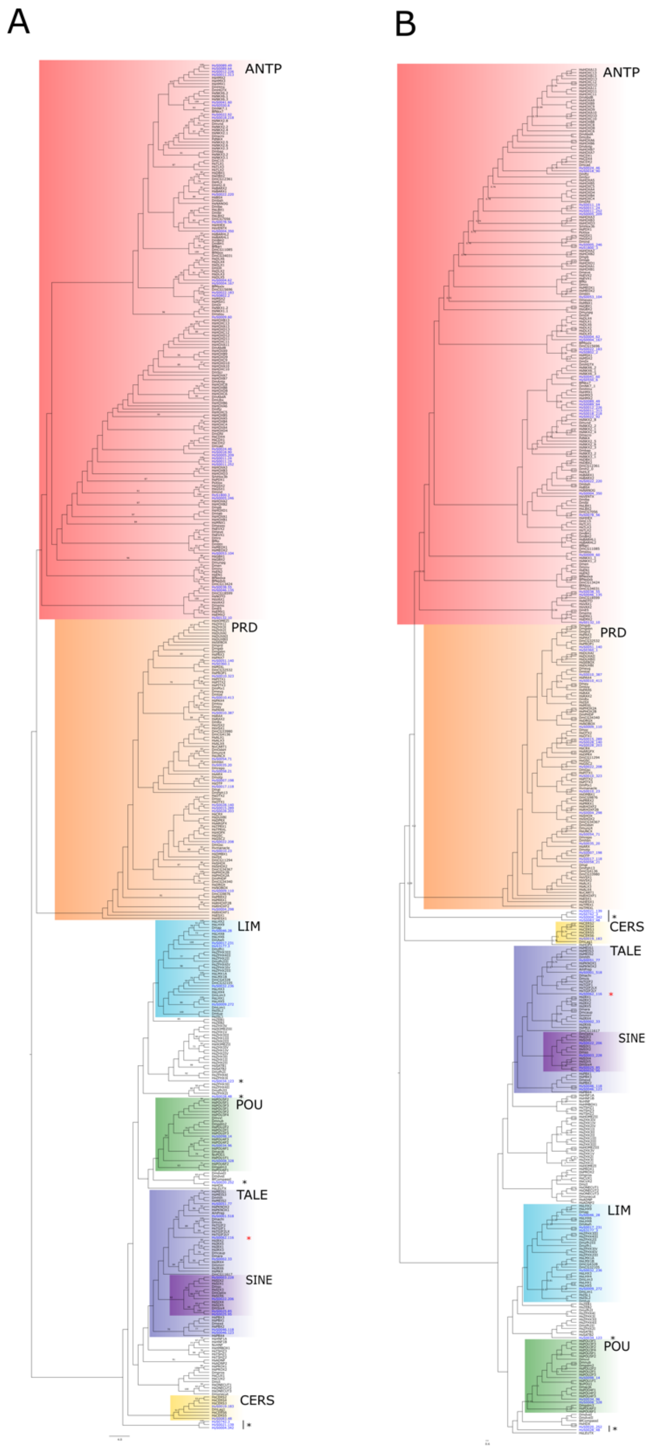

Figure S25: Homeodomain superfamily tree for *Hydractinia symbiolongicarpus.* A. Maximum likelihood tree of 71 *Hydractinia symbiolongicarpus* homeodomain proteins. Trees are midpoint rooted in cladogram format for

display purposes. Bootstraps > 50 are displayed. B. Bayesian phylogenetic tree of *Hydractinia symbiolongicarpus* homeodomain proteins. Trees are midpoint rooted in cladogram format for display purposes. Posterior probabilities > 70 are displayed. In both trees *H. symbiolongicarpus* sequences are highlighted in blue. Black stars represent unclassified sequences. The red star indicates the sequence HyS0062.116 within the TALE class that was subsequently defined as a SINE class protein due to the presence of a secondary SIX domain.

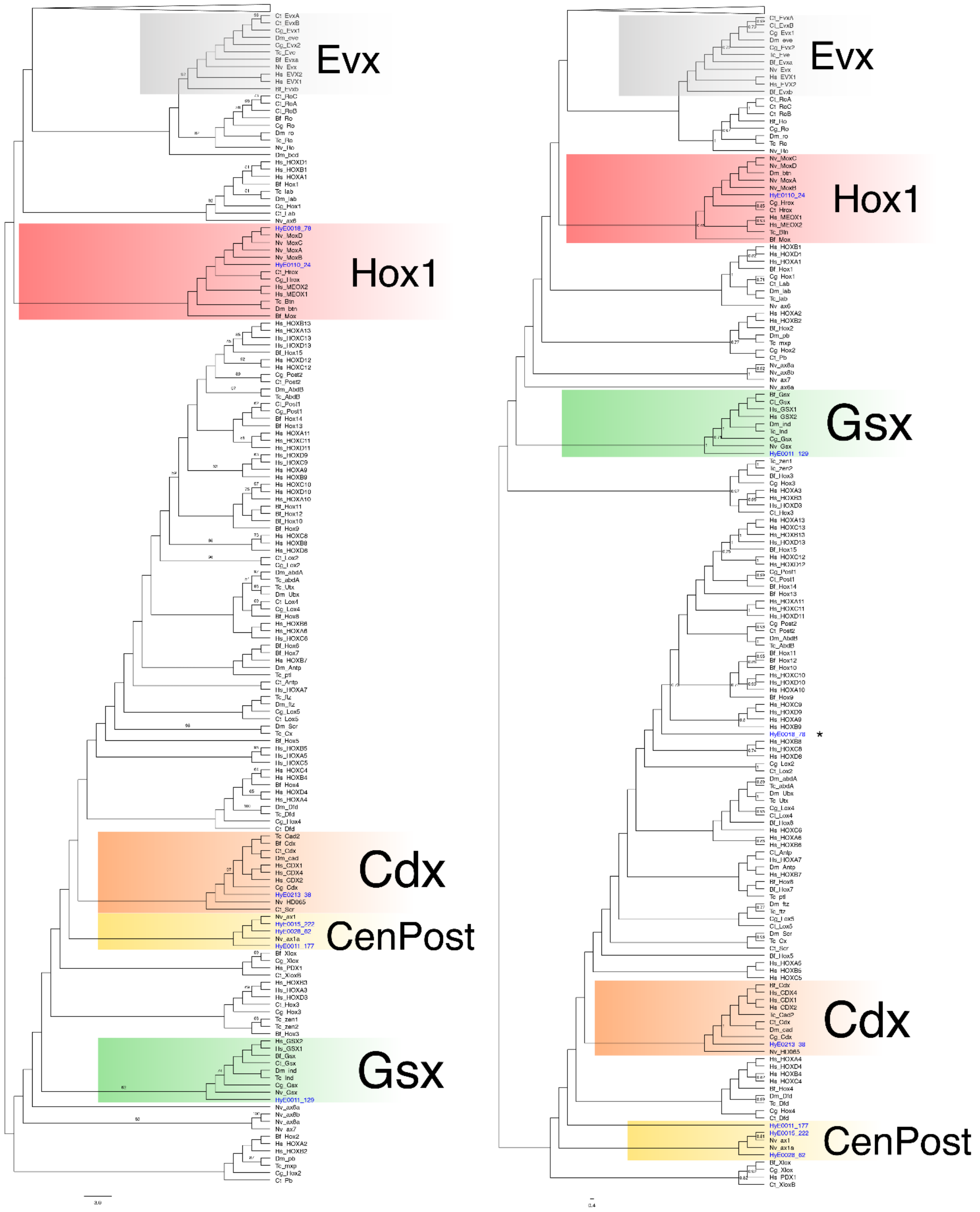

Figure S26: HOX-L tree for *Hydractinia echinata*. A. Maximum likelihood tree of *Hydractinia echinata* HOX-L subclass proteins. Trees are midpoint rooted in cladogram format for display purposes. Bootstraps > 50 are

displayed. B. Bayesian phylogenetic tree of *Hydractinia echinata* HOX-L proteins. Trees are midpoint rooted in cladogram format for display purposes. Posterior probabilities > 70 are displayed. In both trees, *H. echinata* sequences are highlighted in blue.

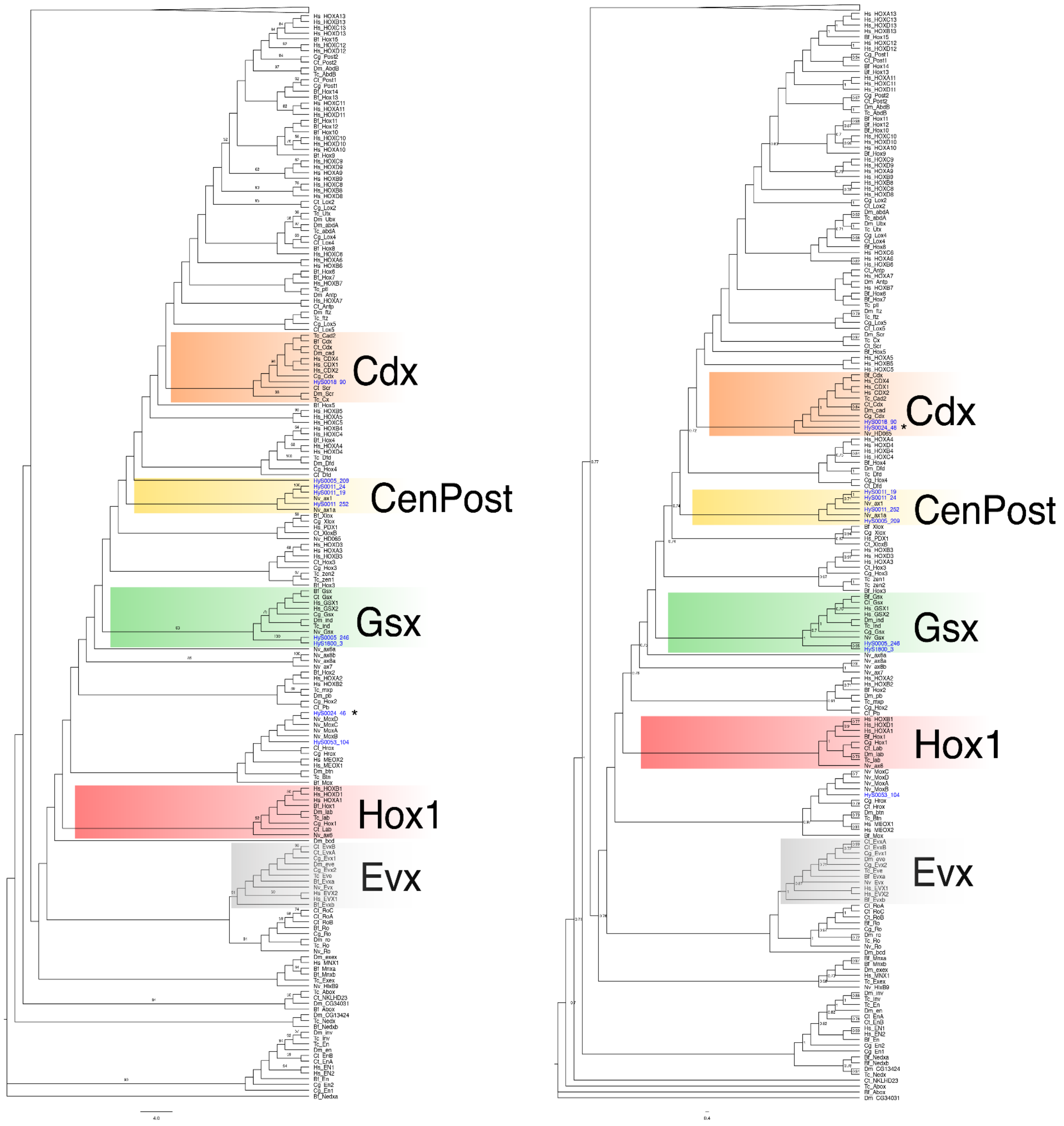

Figure S27: HOX-L subclass tree for *Hydractinia symbiolongicarpus*. A. Maximum likelihood tree of *Hydractinia symbiolongicarpus* HOX-L proteins. Trees are midpoint rooted in cladogram format for display purposes. Bootstraps > 50 are displayed. B. Bayesian phylogenetic tree of *Hydractinia symbiolongicarpus* HOX-

L proteins. Trees are midpoint rooted in cladogram format for display purposes. Posterior probabilities > 70 are displayed. In both trees, *H. symbiolongicarpus* sequences are highlighted in blue.

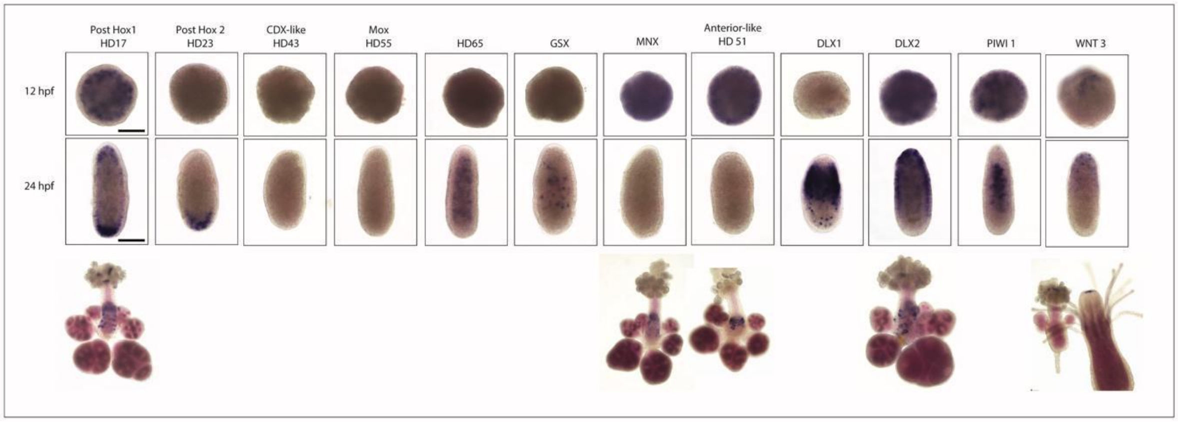

Figure S28: Colorimetric *in situ* hybridization expression patterns for a subset of Hox genes plus *Piwi1* and *Wnt3* in different stages of *Hydractinia*'s life cycle. For all selected genes, patterns are shown at 12 hours post fertilization (hpf) and 24 hpf. Sexual polyps are shown for *HD17*, *MNX*, *HD51*, *DLX2* and *Wnt3*. Expression in a feeding polyp is shown for *Wnt3* only. Scale bars = 100 µm.

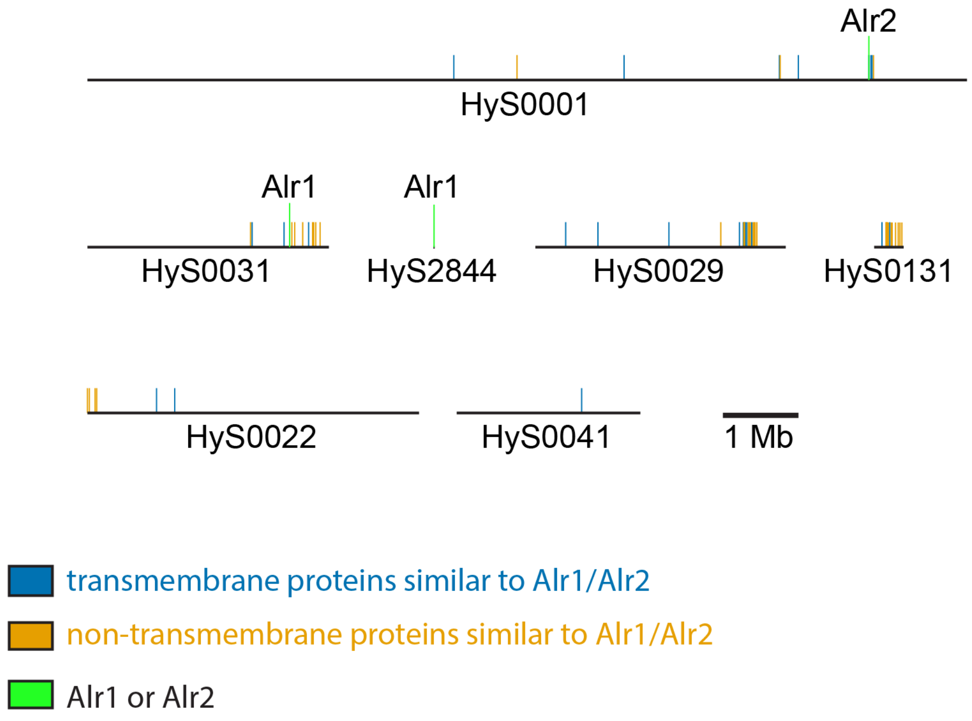

Figure S29: The allorecognition region of the genome represented on multiple *H. symbiolongicarpus* scaffolds. Lines depict transmembrane proteins similar to Alr1/Alr2 (blue), non-transmembrane proteins similar to Alr1/Alr2 (orange), and Alr1 and Alr2 (green) are labeled.

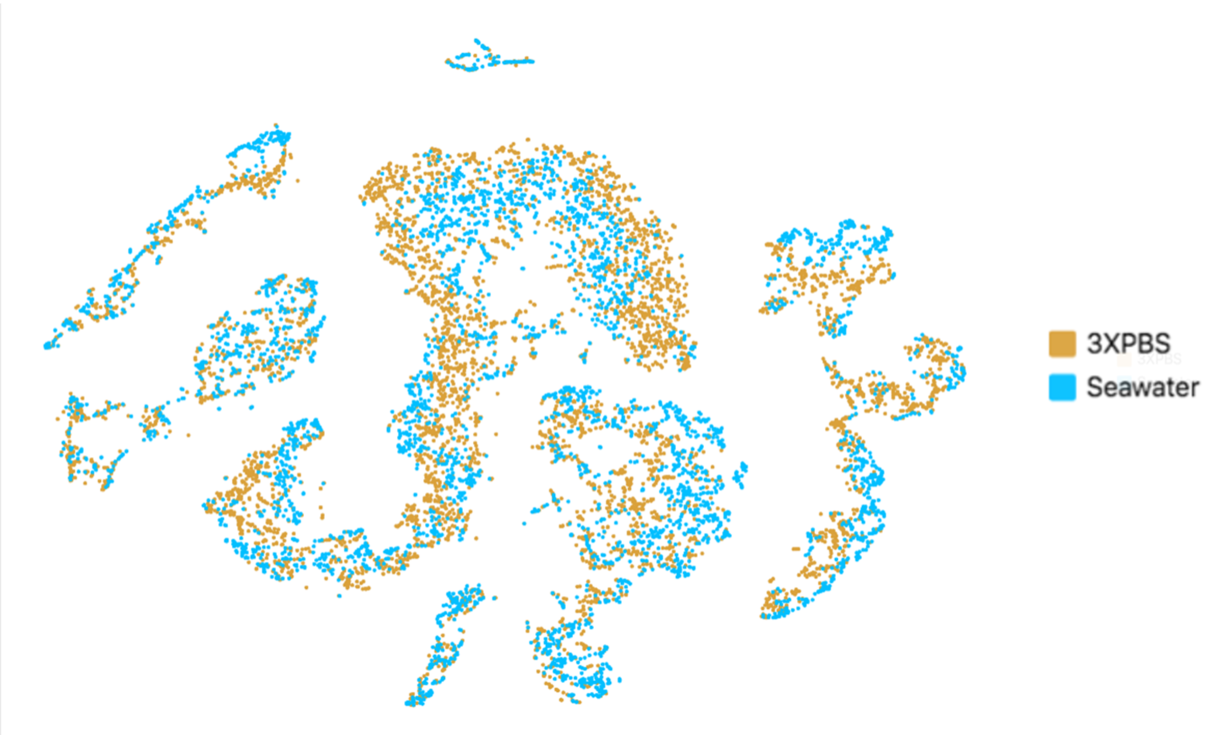

Figure S30: t-SNE representation showing how two different 10X single-cell libraries (library 1: final resuspension in 3XPBS; library 2: final resuspension in CMFASW) contribute to clustering. All clusters have cells contributed from each library. The two libraries were combined for all further analyses.

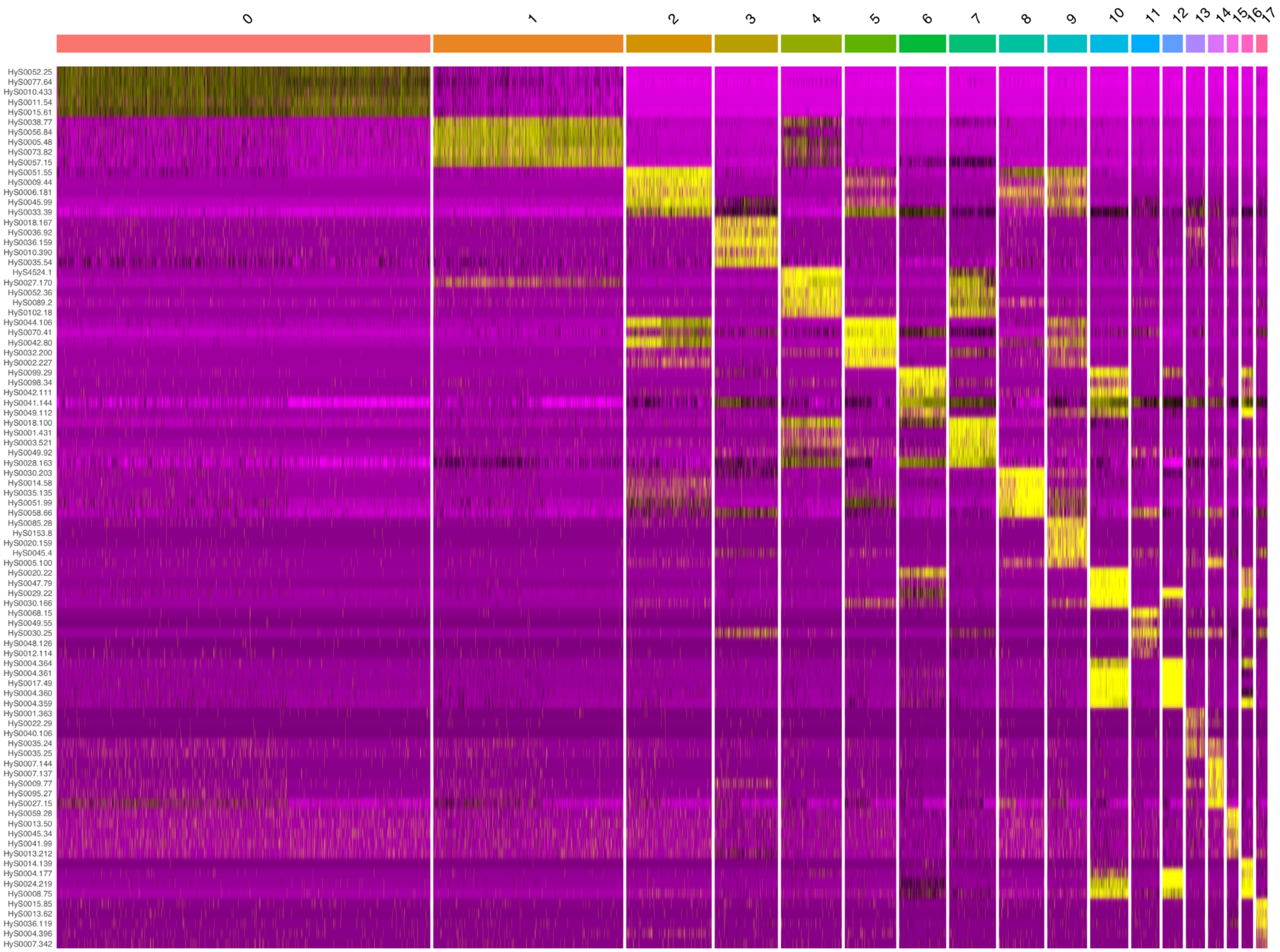

Figure S31: Heatmap of the top five marker genes per cluster from the *H. symbiolongicarpus* single cell atlas. Marker gene IDs are shown on the left and cluster numbers are at the top.

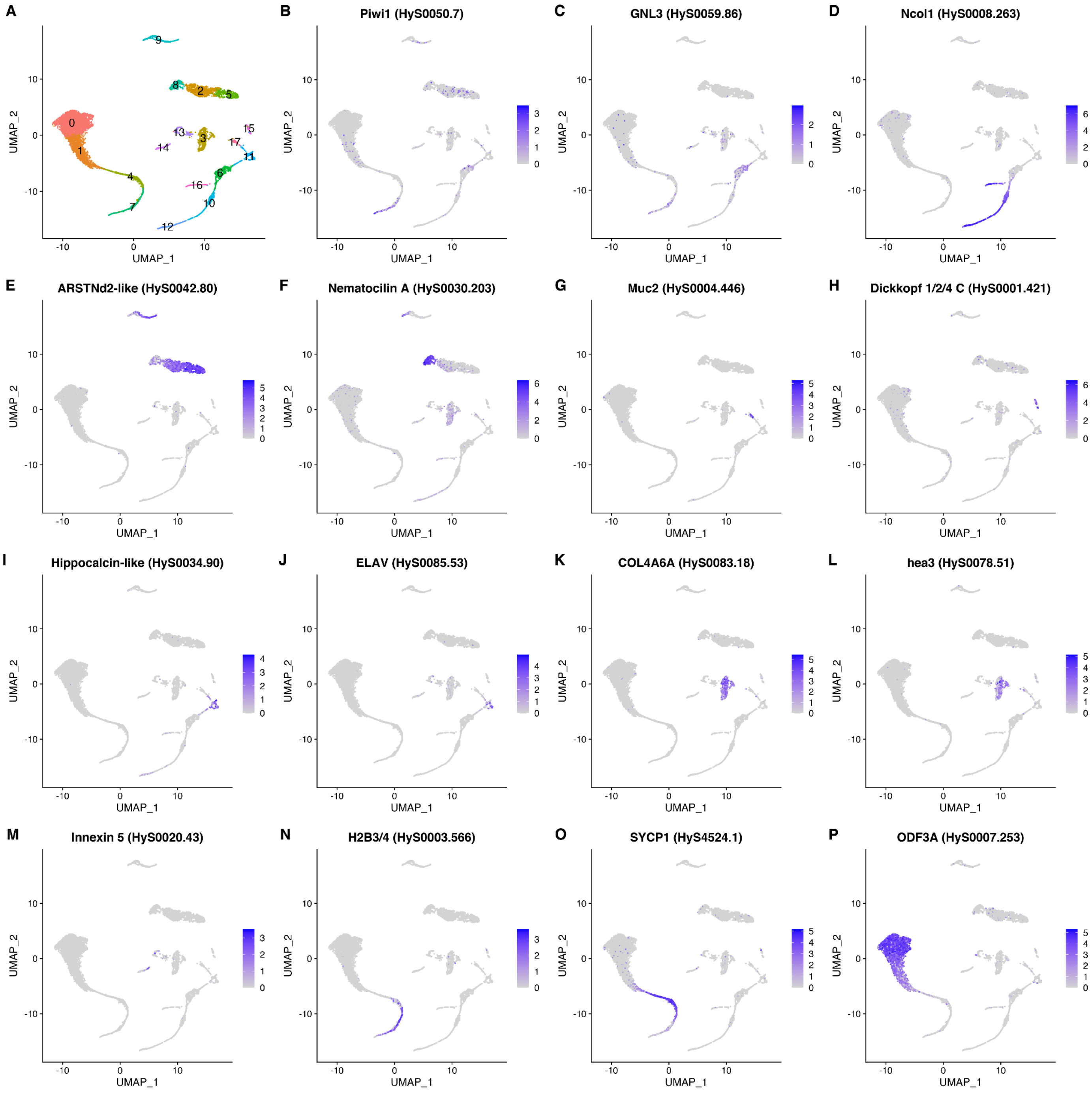

Figure S32: *Hydractinia* single-cell atlas annotated with cell type markers. (A-P) UMAP expression patterns of highly expressed markers characterizing 7 major cell types found in *H. symbiolongicarpus*. (A) *Hydractinia* single-cell atlas represented as a UMAP plot with 18 clusters. (B) Described *Hydractinia* i-cell marker *Piwi1*

(Bradshaw et al., 2015). (C) Described *Hydractinia* i-cell and germ cell marker *GNL3* (Quiroga-Artigas et al. 2022) (D) Described *Hydra* and *Hydractinia* nematoblast marker *Ncol1* ((Bradshaw et al., 2015; David et al., 2008). (E) Uncharacterized *ARSTNd2-like* marker that showed specific nematocyte cluster expression in *Hydra* and *Hydractinia* single-cell atlases (Siebert et al., 2019). (F) Described *Hydra* mature nematocyte marker *Nematocilin A* (Hwang et al., 2007). (G-H) Described *Hydra* mucous and zymogen gland cell markers, *Muc2* and *Dickkopf 1/2/4 C* (Augustin et al., 2006; Siebert et al., 2019). (I-J) Known neuronal markers, *Hippocalcin-like* and *ELAV* (Jacobs et al., 2007; Nakanishi et al., 2012). (K-L) Putative and described epithelial cell type markers, *COL4A6A* from planarian epitheliomuscular cells and *hea3* from endodermal epithelial cells in *Hydractinia* (Fincher et al., 2018; Möhrlen et al., 2006). (M) Described *Hydra* ectodermal epithelial cell marker *Innexin 5* (Buzgariu et al., 2015). (N-P) Described *Hydractinia* sperm progenitor marker (*H2B3/4*) (Török et al. 2023) and *Hydra* spermatogenesis markers *SYCP1* and *ODF3A* (Fraune et al., 2012; Siebert et al., 2019).

Species	Number of samples	1C Mbp	+/- SE Mbp
Hydractinia symbiolongicarpus 291-10	7	514.35	11.19
Hydractinia echinata F4	3	774.70	5.29
Podocoryna carnea PcLH01	2	517.15	9.36
Hydra vulgaris 105	6	1086.47	16.38

Table S1: Genome size estimates for four Hydrozoan species. Propidium-iodide staining of nuclei followed by flow cytometry-based genome size estimates for *H. symbiolongicarpus* strain 291-10, *H. echinata* strain F4, *Podocoryna carnea* strain PcLH01, and *H. vulgaris* strain 105.

Species	Library Name	Number of SMRT cells sequenced	Average insert size (kb)
H. echinata	bath_6	11	9.2
H. echinata	AID0047_1	27	10.6
H. echinata	AID0049_1	16	10.2
H. echinata	AID0051_1	18	8.7
H. echinata	AID0051_2	11	8.9
H. symbiolongicarpus	ARE0002_1	43	8.3
H. symbiolongicarpus	ARE0003_1	18	7.0
H. symbiolongicarpus	ARE0004_1	19	6.9

Table S2: Number of SMRT cells sequenced and average insert sizes for PacBio libraries from *H. symbiolongicarpus* and *H. echinata*.

	H. echinata wild type F4 (PacBio+Dovetail)	*H. symbiolongicarpus* wild type 291-10 (PacBio+Dovetail)
Number of SMRT cells sequenced	83	80
Genome size estimate	774 Mb	514 Mb
Ploidy	Diploid	Diploid
Primary assembly size	565.066 Mb	406.693 Mb
Secondary assembly size	376.122 Mb	335.412 Mb
Assembly method	Canu contigs; Dovetail HiRise scaffolds; PBJelly gap filling; arrow and pilon polishing	Canu contigs; Dovetail HiRise scaffolds; PBJelly gap filling; arrow and pilon polishing
Coverage	84x	94x
Scaffolds – primary assembly	7,767	4,840
Scaffold N50 length	904.2 kb	2,236 kb
% gap	0.005%	0.007%
AT content	65%	65%
Repetitive elements	50.78%	56.30%
BUSCOv5 primary assembly preliminary	C:88.2%[S:81.7%,D:6.5%], F:6.0%,M:5.8%	C:88.3%[S:82.8%,D:5.5%], F:4.7%,M:7.0%
BUSCOv5 primary assembly final	C:89.1%[S:75.8%,D:13.3%], F:5.2%,M:5.7%	C:89.6%[S:83.8%,D:5.8%], F:4.6%,M:5.8%
BUSCOv5 gene models (proteins)	C:90.7%[S:78.4%,D:12.3%], F:2.7%,M:6.6%	C:92.5%[S:82.3%,D:10.2%], F:1.3%,M:6.2%

Table S3: Details of Canu primary and secondary assemblies for *H. echinata* and *H. symbiolongicarpus.* The BUSCOv5 statistics using the Metazoa dataset on the preliminary assemblies were run after Dovetail scaffolding but before any polishing steps (*H. symbiolongicarpus* preliminary assembly had 4,611 scaffolds; *H. echinata* preliminary assembly had 7,095 scaffolds). The BUSCO statistics on the final assemblies were run after all polishing steps. Abbreviations: C=Complete, D=Duplicate, F=Fragmented, M=Missing, S=Single-Copy

	H. echinata wild type F4 (PacBio+Dovetail)	*H. symbiolongicarpus* wild type 291-10 (PacBio+Dovetail)
Number of SMRT cells	83	80
Genome size estimate	774 Mb	514 Mb
Ploidy	Diploid	Diploid
Primary assembly size	456 Mb	447 Mb
Secondary assembly size	290 Mb	269 Mb
Assembly method	Falcon unzip module; Dovetail HiRise scaffolding	Falcon unzip module; Dovetail HiRise scaffolding
Coverage	84x	94x
Scaffolds – primary assembly	2,361	2,081
Scaffold N50 length	971 kb	1,527 kb
AT content	65%	65%
BUSCOv5 primary assembly	C:86.7%[S:80.2%,D:6.5%], F:6.7%,M:6.6%	C:87.8%[S:77.8%,D:10.0%], F:5.0%,M:7.2%

Table S4: Details of Falcon_unzip primary and secondary assemblies for *H. echinata* and *H. symbiolongicarpus.* The BUSCOv5 statistics using the Metazoa dataset on the primary assemblies were run after Dovetail scaffolding but before any polishing steps. Abbreviations: C=Complete, D=Duplicate, F=Fragmented, M=Missing, S=Single-Copy.

	H. symbiolongicarpus			*H. echinata*		
	**Total Length (bp)**	**Total Number**	**Average Length (bp)**	**Total Length (bp)**	**Total Number**	**Average Length (bp)**
Introns	130,132,768	142,541	912.95	165,427,111	161,489	1,024.39
Exons	36,542,751	164,576	222.04	45,165,408	190,327	237.30
Transcripts (includes UTR)	166,675,519	22,035	7,564.13	210,592,519	28,838	7,302.60
Proteins	11,219,524	22,035	509.17	13,382,194	28,838	464.05
Intergenic regions	139,279,695	20,851	6,679.76	197,688,250	25,999	7,603.69
Scaffolds	406,663,980	4,840	84,021.48	565,065,865	7,767	72,752.14
	**Total length (bp)**	**Total length genome (bp)**	**Percentage (%)**	**Total length (bp)**	**Total length genome (bp)**	**Percentage (%)**
Total coding	33,724,677	406,663,980	8.29	40,233,096	565,065,865	7.12
Total Non-coding	372,939,303	406,663,980	91.71	524,832,769	565,065,865	92.88
	**Total number of introns/exons**	**Total number of genes**	**Average number of introns/exons per gene**	**Total number of introns/exons**	**Total number of genes**	**Average number of introns/exons per gene**
Introns	142,541	22,035	6.47	161,489	28,838	5.60
Exons	164,576	22,035	7.47	190,327	28,838	6.60

Table S5: Summary statistics for final gene models generated with a pipeline involving Augustus and PASA.

	genes	trans-cripts	N50	BUSCO v5 statistics
Trinity_denovo_allpaths_hc12*	52,470	69,084	1768	C:94.8%[S:70.6%,D:24.2%],F:2.1%,M:3.1%
Trinity_denovo_allpaths	61,726	83,937	1718	C:95.2%[S:61.9%,D:33.3%],F:1.7%,M:3.1%
Trinity_denovo_hc12	79,585	101,233	1530	C:95.2%[S:68.8%,D:26.4%],F:2.1%,M:2.7%
Trinity_genome_guided_ unmasked_with_dta	70,740	79,161	1551	C:93.7%[S:70.5%,D:23.2%],F:2.7%,M:3.6%
Trinity_genome_guided_ unmasked_without_dta	70,534	79,650	1588	C:94.3%[S:70.3%,D:24.0%],F:2.1%,M:3.6%
Trinity_genome_guided_ masked_with_dta	72,281	80,835	1490	C:93.0%[S:70.4%,D:22.6%],F:3.4%,M:3.6%
Trinity_genome_guided_ masked_without_dta	71,956	81,215	1527	C:93.8%[S:69.8%,D:24.0%],F:2.5%,M:3.7%
TopHat/stringtie_genome_ guided_unmasked_allpaths	58,556	58,556	2566	C:93.0%[S:65.7%,D:27.3%],F:2.3%,M:4.7%
HISAT2/stringtie_unmasked_ge nome_with_dta	60,605	60,605	2524	C:93.4%[S:62.3%,D:31.1%],F:2.1%,M:4.5%
HISAT2/stringtie_unmasked_ge nome_without_dta	59,163	59,163	2666	C:94.1%[S:61.2%,D:32.9%],F:1.7%,M:4.2%
HISAT2/stringtie_masked _with_dta	57,614	57,614	2400	C:91.5%[S:62.7%,D:28.8%],F:2.8%,M:5.7%
HISAT2/stringtie_masked _without_dta	56,518	56,518	2542	C:91.9%[S:74.3%,D:17.6%],F:2.4%,M:5.7%

Table S6: *H. symbiolongicarpus* adult strand-specific transcriptomes statistics including numbers of 'genes' and 'transcripts' as defined by Trinity, the N50 length and BUSCOv5 statistics using the Metazoa dataset and 'transcriptome' mode. The best transcriptome according to N50 and BUSCO statistics is marked with an asterisk.

	genes	transcripts	N50	BUSCO v5 statistics
Trinity_denovo_hc12	275,502	383,439	849	C:95.8%[S:54.3%,D:41.5%],F:1.7%,M:2.5%
Trinity_denovo_allpaths_hc12	116,790	152,694	1392	C:95.6%[S:64.7%,D:30.9%],F:1.8%,M:2.6%
Trinity_denovo_allpaths_hc12 _lib1_13032602	83,371	113,148	1458	C:95.4%[S:64.7%,D:30.7%],F:1.9%,M:2.7%
Trinity_denovo_allpaths_hc12 _lib2_13032603*	86,753	115,184	1480	C:95.6%[S:67.1%,D:28.5%],F:1.9%,M:2.5%
Trinity_denovo_allpaths_hc12 _lib3_13032604	84,314	112,022	1465	C:95.8%[S:65.7%,D:30.1%],F:1.5%,M:2.7%

Table S7: *H. echinata* adult strand-specific transcriptomes statistics including numbers of ‘genes’ and ‘transcripts’ as defined by Trinity, the N50 length, and BUSCOv5 statistics using the Metazoa dataset and ‘transcriptome’ mode. The best transcriptome according to N50 and BUSCO statistics is marked with an asterisk.

		developmental stages		adult		combined	
		all_genes	unassigned	all_genes	unassigned	all_genes	unassigned
H. symbiolong-icarpus	mean overlap per gene	71.84	43.55	69.82	35.71	81.84	57.09
	perccent genes with >99% overlap	68.54	40.37	53.12	23.92	75.72	49.2
	perccent genes with >90% overlap	69.55	40.93	61.82	27.93	78.11	51.28
	perccent genes with >50% overlap	71.96	43.42	70.87	36.04	82.13	57.14
	perccent genes with <10% overlap	26.07	53.93	23.59	56.98	14.99	37.56
		regeneration data		adult		combined	
		all_genes	unassigned	all_genes	unassigned	all_genes	unassigned
H. echinata	mean overlap per gene	56.71	24.04	66.19	38.48	70.75	43.16
	perccent genes with >99% overlap	43.84	17.54	49.19	25.87	57.83	31.04
	perccent genes with >90% overlap	50.07	19.77	57.71	30.35	63.45	35.35
	perccent genes with >50% overlap	57.27	23.81	67.13	38.85	71.42	43.41
	perccent genes with <10% overlap	37.3	71.22	26.74	53.72	23	49.26

Table S8: Percentage of gene models with transcript support for each species of *Hydractinia*. Results are shown for different RNAseq datasets (developmental stages or adult for *H. symbiolongicarpus* and polyp head regeneration or adult for *H. echinata*). Combined results reflect the level of transcript support when transcripts from different datasets are combined. Unassigned refers to gene models that were not found in orthogroups in our OrthoFinder analysis.

Gene Name	EMBL-EBI accession
Cox2	CUS58563.1
Atp8	CUS58564.1
Atp6	CUS58565.1
Cox3	CUS58566.1
Nad2	CUS58567.1
Nad5	CUS58568.1
Nad6	CUS58569.1
Nad3	CUS58570.1
Nad4L	CUS58571.1
Nad1	CUS58572.1
Nad4	CUS58573.1
Cob	CUS58574.1
Cox1	CUS58575.1

Table S10: EMBL-EBI accession numbers for the *H. symbiolongicarpus* mitochondrial genes as determined by (Kayal et al. 2015) that were used to annotate the mitochondrial genomes of *H. symbiolongicarpus* and *H. echinata.*

	H. symbiolongicarpus	*H. echinata*
**RepeatMasker de novo**		
Genome total bases	406,663,979 bp	565,065,865 bp
Repeats total bases	201,402,193 bp	309,993,746 bp
% genome repetitive regions	49.52%	54.86%
**RepeatMasker de novo and known combined**		
Genome total bases	406,663,979 bp	565,065,865 bp
Repeats total bases	206,502,560 bp	318,157,919 bp
% genome repetitive regions	50.78%	56.30%
**RepeatMasker de novo and known combined minus overlap with transcripts mapped to the assemblies (used to create masked assembly files)**		
Genome total bases	406,663,979 bp	565,065,865 bp
Repeats total bases	163,768,240 bp	240,920,515 bp
% genome repetitive regions	40.27%	42.64%

Table S13: Number of bases and % of the genome found in repeat region for the de novo RepeatMasker analysis, the de novo plus known RepeatMasker analysis combined, and the combined analysis minus overlap with transcripts that mapped to the assemblies performed for each *Hydractinia* species.

Total length:	565065865 bp		
Bases masked:	310005690 bp (54.86 %)		
	Number of elements	Length occupied	Percentage of sequence
SINEs:	29842	7945374 bp	1.41 %
ALUs	0	0 bp	0.00 %
MIRs	1691	287275 bp	0.05 %
LINEs:	45523	23505904 bp	4.16 %
LINE1	0	0 bp	0.00 %
LINE2	13080	7615272 bp	1.35 %
L3/CR1	2620	995658 bp	0.18 %
LTR elements:	11165	8169382 bp	1.45 %
ERVL	0	0 bp	0.00%
ERVL-MaLRs	0	0 bp	0.00%
ERV_classI	541	77584 bp	0.01%
ERV_classII	151	27099 bp	0.00%
DNA elements:	129188	62310653 bp	11.03 %
hAT-Charlie	0	0 bp	0.00 %
TcMar-Tigger	0	0 bp	0.00%
Unclassified:	359462	200289812 bp	35.45 %
Total interspersed repeats:		302221125 bp	53.48 %
Small RNA:	9813	4061162 bp	0.72 %
Satellites:	745	87281 bp	0.02 %
Simple repeats:	76138	6234152 bp	1.10 %
Low complexity:	14294	732878 bp	0.13 %

Table S14: Summary output of RepeatMasker "de novo" run for *H. echinata*. RepeatMasker Combined Database: Dfam_Consensus-20170127, RepBase-20170127. Run with cross_match version 1.090518.

Total length:	565065865 bp		
Bases masked:	37006431 bp (6.55 %)		
	Number of elements	Length occupied	Percentage of sequence
Retroelements	50576	15354418 bp	2.72 %
SINEs:	14285	1627293 bp	0.29 %
Penelope	2678	815396 bp	0.14 %
LINEs:	23316	8670822 bp	1.53 %
CRE/SLACS	992	481207 bp	0.09 %
L2/CR1/Rex	11668	3897109 bp	0.69 %
R1/LOA/Jockey	1014	84071 bp	0.01 %
R2/R4/NeSL	705	144907 bp	0.03 %
RTE/Bov-B	3384	2962780 bp	0.52 %
L1/CIN4	740	54720 bp	0.01 %
LTR elements:	12975	5056303 bp	0.89 %
BEL/Pao	2521	1044854 bp	0.18 %
Ty1/Copia	1218	1084829 bp	0.19 %
Gypsy/DIRS1	7227	2667182 bp	0.47 %
Retroviral	1184	76920 bp	0.01 %
DNA transposons:	46797	9567067 bp	1.69 %
hobo-Activator	5178	711173 bp	0.13 %
Tc1-IS630-Pogo	8184	840050 bp	0.15 %
En-Spm	0	0 bp	0.00 %
MuDR-IS905	0	0 bp	0.00 %
PiggyBac	1352	219874 bp	0.04 %
Tourist/Harbinger	1836	509651 bp	0.09 %
Other (Mirage, P-element, Transib)	1725	617843 bp	0.11 %
Rolling-circles	0	0 bp	0.00%
Unclassified:	6136	1695133 bp	0.30 %
Total interspersed repeats:		26616618 bp	4.71 %
Small RNA:	25177	2776046 bp	0.49 %
Satellites:	5890	794969 bp	0.14 %
Simple repeats:	107790	5850258 bp	1.04 %
Low complexity:	21966	1104514 bp	0.20 %

Table S15: Summary output of RepeatMasker "known" run for *H. echinata*. The query species was assumed to be metazoan. RepeatMasker Combined Database: Dfam_Consensus-20170127, RepBase-20170127. Run with cross_match version 1.090518.

Total length:	406693435 bp		
Bases masked:	201416248 bp (49.53 %)		
	Number of elements	Length occupied	Percentage of sequence
SINEs:	20120	4546984 bp	1.12 %
ALUs	0	0 bp	0.00 %
MIRs	0	0 bp	0.00 %
LINEs:	32350	15784905 bp	3.88 %
LINE1	0	0 bp	0.00 %
LINE2	8654	5466273 bp	1.34 %
L3/CR1	536	320276 bp	0.08 %
LTR elements:	13092	12489546 bp	3.07 %
ERVL	95	14525 bp	0.00 %
ERVL-MaLRs	0	0 bp	0.00 %
ERV_classI	847	60238 bp	0.01 %
ERV_classII	0	0 bp	0.00 %
DNA elements:	73929	45913880 bp	11.29 %
hAT-Charlie	119	36758 bp	0.01 %
TcMar-Tigger	0	0 bp	0.00 %
Unclassified:	255696	124569550 bp	30.63 %
Total interspersed repeats:		203304865 bp	49.99 %
Small RNA:	4431	915189 bp	0.23 %
Satellites:	1022	335516 bp	0.08 %
Simple repeats:	56850	3787338 bp	0.93 %
Low complexity:	12323	601250 bp	0.15 %

Table S16: Summary output of RepeatMasker "de novo" run for *H. symbiolongicarpus*. RepeatMasker Combined Database: Dfam_Consensus-20170127, RepBase-20170127. Run with cross_match version 1.090518.

Total length:	406693435 bp		
Bases masked:	24634196 bp (6.06 %)		
	Number of elements	Length occupied	Percentage of sequence
Retroelements	38392	10442084 bp	2.57 %
SINEs:	10555	1159024 bp	0.28 %
Penelope	2325	671796 bp	0.17 %
LINEs:	17709	5718216 bp	1.41 %
CRE/SLACS	815	382208 bp	0.09 %
L2/CR1/Rex	8001	2484462 bp	0.61 %
R1/LOA/Jockey	1492	171761 bp	0.04 %
R2/R4/NeSL	510	88865 bp	0.02 %
RTE/Bov-B	2387	1705230 bp	0.42 %
L1/CIN4	575	37899 bp	0.01 %
LTR elements:	10128	3564844 bp	0.88 %
BEL/Pao	1827	862999 bp	0.21 %
Ty1/Copia	1103	963819 bp	0.24 %
Gypsy/DIRS1	5299	1544615 bp	0.38 %
Retroviral	1116	70736 bp	0.02 %
DNA transposons:	28843	5968002 bp	1.47 %
hobo-Activator	3914	698796 bp	0.17 %
Tc1-IS630-Pogo	4576	517942 bp	0.13 %
En-Spm	0	0 bp	0.00 %
MuDR-IS905	0	0 bp	0.00 %
PiggyBac	1098	147833 bp	0.04 %
Tourist/Harbinger	1275	340380 bp	0.08 %
Other (Mirage, P-element, Transib)	1263	471392 bp	0.12 %
Rolling-circles	0	0 bp	0.00 %
Unclassified:	4153	757912 bp	0.19 %
Total interspersed repeats:		17167998 bp	4.22 %
Small RNA:	20438	1573742 bp	0.39 %
Satellites:	6966	895598 bp	0.22 %
Simple repeats:	76794	4271472 bp	1.05 %
Low complexity:	16883	819654 bp	0.20 %

Table S17: Summary output of RepeatMasker "known" run for *H. symbiolongicarpus*. The query species was assumed to be metazoan. RepeatMasker Combined Database: Dfam_Consensus-20170127, RepBase-20170127. Run with cross_match version 1.090518.

RNA Family	Rfam Accession	*H. ech.* Count	*H. sym.* Count	*H. ech.* Fraction In TA	*H. sym.* Fraction In TA	*H. ech.* Largest TA *X*(*N*)	*H. sym.* Largest TA *X*(*N*)
tRNA	N/A	28055	24077	0.556	0.630	254(210)	176(159)
5S rRNA	RF00001	1287	1891	0.887	0.883	73(72)	79(76)
Metazoa SRP	RF00017	513	35	0.922	0.371	78(73)	13(12)
U5	RF00020	343	167	0.883	0.880	70(68)	56(52)
Histone3	RF00032	255	195	0.071	0.051	18(13)	10(8)
LSU_rRNA eukarya	RF02543	251	50	0.088	-	11(10)	-
U4	RF00015	172	124	0.959	0.823	68(67)	70(69)
U1	RF00003	45	219	0.444	0.731	20(15)	40(38)
SSU rRNA eukarya	RF01960	226	36	0.102	0.306	13(11)	11(8)
5 8S rRNA	RF00002	199	25	0.161	-	11(10)	-
U6	RF00026	132	53	0.970	1.000	111(108)	53(52)
U2	RF00004	50	65	0.420	0.154	21(16)	10(8)
U3	RF00012	36	45	0.917	0.533	19(18)	13(12)
K chan RES	RF00485	14	14	-	-	-	-
U12	RF00007	13	10	-	-	-	-
RNaseP nuc	RF00009	13	4	-	-	-	-
SNORD36	RF00049	9	6	-	-	-	-
U8	RF00096	5	6	-	-	-	-
SNORD57	RF00274	3	6	-	-	-	-
U4atac	RF00618	5	3	-	-	-	-
Vault	RF00006	3	4	-	-	-	-
snosnR60 Z15	RF00309	3	3	-	-	-	-
SNORA73	RF00045	3	3	-	-	-	-
U6atac	RF00619	3	2	-	-	-	-
SNORD18	RF00093	2	3	-	-	-	-
SNORD12	RF00581	2	2	-	-	-	-
SNORD24	RF00069	2	2	-	-	-	-
U11	RF00548	2	2	-	-	-	-
SCARNA8	RF00286	2	1	-	-	-	-
SNORA79	RF00600	1	1	-	-	-	-
SNORD103	RF00188	1	1	-	-	-	-
RNase MRP	RF00030	1	1	-	-	-	-
snR191	RF01263	-	1	-	-	-	-
**Total**		**31651**	**27057**	**0.568**	**0.642**	**-**	**-**

Table S19: **Number and attributes of ncRNA annotations in the *H. echinata* and *H. symbiolongicarpus* v1.0 genome assemblies.** Zero values are represented by a dash. Tandem Array is abbreviated as 'TA'. The first number in the two rightmost Largest TA columns is the total number of predictions in the largest tandem array for the family (X parameter; see definition in Supplemental Material and Methods) and number in parentheses is the number of spaces between predictions that satisfy the spacing constraints (N parameter; see definition in Supplemental Materials and Methods); $N/(X-1)$ must be ≥ 0.75. The tRNA row includes a sum

across all isotype-specific tRNAscan-SE predictions, but tandem arrays were determined for each isotype-specific set independently. The largest *H. echinata* tRNA tandem array is for isotype *Pro*. The largest *H. symbiolongicarpus* tRNA tandem array is for isotype *Leu*.

Rfam family	Rfam accession	total count	high-sc:yes fragment:no	high-sc:yes fragment:yes	high-sc:no fragment:no	high-sc:no fragment:yes
5S_rRNA	RF00001	1287	918	-	366	3
Metazoa_SRP	RF00017	513	137	-	368	8
U5	RF00020	343	330	-	13	-
Histone3	RF00032	255	130	-	125	-
LSU_rRNA_eukarya	RF02543	251	144	-	29	78
SSU_rRNA_eukarya	RF01960	226	158	-	21	47
5_8S_rRNA	RF00002	199	175	-	18	6
U4	RF00015	172	152	-	16	4
U6	RF00026	132	123	-	6	3
U2	RF00004	50	37	-	10	3
U1	RF00003	45	40	-	4	1
U3	RF00012	36	28	-	7	1
K_chan_RES	RF00485	14	4	-	10	-
RNaseP_nuc	RF00009	13	4	-	9	-
U12	RF00007	13	7	-	6	-
SNORD36	RF00049	9	9	-	-	-
U8	RF00096	5	4	-	1	-
U4atac	RF00618	5	4	-	1	-
U6atac	RF00619	3	3	-	-	-
snosnR60_Z15	RF00309	3	3	-	-	-
SNORD57	RF00274	3	2	-	1	-
SNORA73	RF00045	3	3	-	-	-
Vault	RF00006	3	2	-	1	-
SCARNA8	RF00286	2	2	-	-	-
U11	RF00548	2	2	-	-	-
SNORD18	RF00093	2	2	-	-	-
SNORD24	RF00069	2	2	-	-	-
SNORD12	RF00581	2	2	-	-	-
SNORA79	RF00600	1	1	-	-	-
RNase_MRP	RF00030	1	1	-	-	-
SNORD103	RF00188	1	1	-	-	-
total	-	3596	2430	-	1012	154

Table S20: Per-Rfam-family counts of RNA annotations in the *H. echinata* v1.0 genome assembly. Zero values are represented as "-". "High-scoring" is abbreviated as "high-sc". RNAs were defined as ”high-scoring” if the Infernal cmsearch bit score was within 10% of the top-scoring prediction for that family in the genome, and as ”fragments” if their length was less than 90% the length of that top-scoring prediction.

Rfam family	Rfam accession	total count	high-sc:yes fragment:no	high-sc:yes fragment:yes	high-sc:no fragment:no	high-sc:no fragment:yes
5S_rRNA	RF00001	1891	1501	-	382	8
U1	RF00003	219	49	-	9	161
Histone3	RF00032	195	175	-	20	-
U5	RF00020	167	131	-	35	1
U4	RF00015	124	115	-	8	1
U2	RF00004	65	52	-	12	1
U6	RF00026	53	53	-	-	-
LSU_rRNA_eukarya	RF02543	50	10	-	7	33
U3	RF00012	45	42	-	2	1
SSU_rRNA_eukarya	RF01960	36	15	-	4	17
Metazoa_SRP	RF00017	35	30	-	3	2
5_8S_rRNA	RF00002	25	20	-	4	1
K_chan_RES	RF00485	14	3	-	11	-
U12	RF00007	10	6	-	4	-
SNORD57	RF00274	6	4	-	2	-
U8	RF00096	6	3	-	3	-
SNORD36	RF00049	6	6	-	-	-
RNaseP_nuc	RF00009	4	2	-	1	1
Vault	RF00006	4	2	-	2	-
snosnR60_Z15	RF00309	3	3	-	-	-
U4atac	RF00618	3	3	-	-	-
SNORA73	RF00045	3	3	-	-	-
SNORD18	RF00093	3	3	-	-	-
U6atac	RF00619	2	2	-	-	-
SNORD12	RF00581	2	2	-	-	-
U11	RF00548	2	2	-	-	-
SNORD24	RF00069	2	2	-	-	-
SNORD103	RF00188	1	1	-	-	-
snR191	RF01263	1	1	-	-	-
SNORA79	RF00600	1	1	-	-	-
SCARNA8	RF00286	1	1	-	-	-
RNase_MRP	RF00030	1	1	-	-	-
total	-	2980	2244	-	509	227

Table S21: Per-Rfam-family counts of RNA annotations in the *H. symbiolongicarpus* v1.0 genome assembly. Zero values are represented as “-”. “High-scoring” is abbreviated as “high-sc”. RNAs were defined as ”high-scoring” if the Infernal cmsearch bit score was within 10% of the top-scoring prediction for that family in the genome, and as ”fragments” if their length was less than 90% the length of that top-scoring prediction.

tRNA isotype	total count	fraction in tandem array (TA)	fraction flagged as pseudo	fraction overlap with HydSINE1 repeat	fraction pseudo and in TA	fraction HydSINE1 and in TA	fraction HydSINE1 and not pseudo
Leu	4840	0.517	0.053	0.002	0.024	-	-
Arg	3847	0.632	0.346	0.011	0.108	-	0.0013
Gly	2171	0.198	0.064	0.004	0.017	-	-
Pro	1908	0.823	0.045	-	0.031	-	-
Asn	1712	0.960	0.048	-	0.044	-	-
Undet	1648	-	0.140	0.004	-	-	-
Trp	1628	0.403	0.498	0.464	0.022	-	0.0129
Glu	1363	0.795	0.031	0.003	0.014	-	-
Ser	1266	0.573	0.081	0.002	0.023	-	-
Thr	1136	0.670	0.252	-	0.014	-	-
His	1105	0.900	0.016	-	0.012	-	-
Lys	1084	0.284	0.509	-	0.009	-	-
Val	874	0.713	0.025	-	0.001	-	-
Gln	856	0.709	0.049	0.001	0.008	-	-
Ile	561	0.825	0.050	-	-	-	-
Met	521	0.691	0.192	0.006	0.023	-	-
Ala	410	0.507	0.190	-	0.007	-	-
Sup	409	-	0.328	0.174	-	-	0.0024
Cys	227	0.423	0.093	0.079	0.004	-	0.0044
Phe	210	0.452	0.057	0.005	0.010	-	-
Tyr	109	0.110	0.055	-	-	-	-
Asp	84	0.143	0.119	-	-	-	-
iMet	62	0.194	-	-	-	-	-
SeC	24	-	-	-	-	-	-
total	28055	0.556	0.156	0.033	0.030	-	0.0010

Table S22: Counts and attributes of per-tRNA-isotype tRNAscan-SE predictions in the *H. echinata* v1.0 genome assembly. The second column includes absolute counts of predictions and all columns right of that include fractions of those total counts. Zero values are represented as "-". "Tandem array" is abbreviated as "TA".

tRNA isotype	total count	fraction in tandem array (TA)	fraction flagged as pseudo	fraction overlap with HydSINE1 repeat	fraction pseudo and in TA	fraction HydSINE1 and in TA	fraction HydSINE1 and not pseudo
Leu	3247	0.602	0.083	0.003	0.059	-	0.0003
Arg	2839	0.579	0.363	0.004	0.092	-	-
Gln	2758	0.773	0.045	-	0.019	-	-
Ser	2035	0.724	0.051	0.001	0.019	-	-
Lys	1652	0.627	0.240	0.002	0.097	-	-
Val	1358	0.780	0.033	-	0.008	-	-
Asn	1319	0.818	0.187	-	0.153	-	-
Pro	1107	0.643	0.060	0.001	0.036	0.00090	0.0009
Thr	1095	0.797	0.117	0.001	0.024	-	-
Undet	974	-	0.120	-	-	-	-
Gly	940	0.564	0.135	-	0.060	-	-
His	844	0.823	0.059	-	0.034	-	-
Ala	764	0.627	0.110	-	0.048	-	-
Trp	724	0.519	0.246	0.225	0.008	-	0.0041
Glu	653	0.504	0.096	0.002	0.026	-	-
Met	489	0.738	0.059	-	0.002	-	-
Sup	388	-	0.588	0.492	-	-	0.0077
Ile	268	0.743	0.067	-	-	-	-
Phe	139	0.360	0.029	-	-	-	-
Cys	126	0.302	0.175	0.135	-	-	0.0079
Tyr	110	0.291	0.036	0.018	-	-	-
Asp	100	0.290	0.010	-	-	-	-
iMet	87	0.506	-	-	-	-	-
SeC	61	0.787	-	-	-	-	-
total	24077	0.630	0.138	0.017	0.047	0.00004	0.0004

Table S23: Counts and attributes of per-tRNA-isotype tRNAscan-SE predictions in the *H. symbiolongicarpus* v1.0 genome assembly. The second column includes absolute counts of predictions and all columns right of that include fractions of those total counts. Zero values are represented as “-”. “Tandem array” is abbreviated as “TA”.

tRNA isotype or Rfam family	total count	fraction in TA	eligible for TA count	fraction of eligible in TA	largest tandem array per family (maximum X) sequence name/ strand	count in TA (X)	fraction within Dspan (N/(X-1))	spacer range and span (Dspan)
tRNA-Leu	4840	0.517	4608	0.543	HyE0907/+	105	0.750	295-391(97)
tRNA-Arg	3847	0.632	2942	0.827	HyE0172/+	123	0.902	385-447(63)
tRNA-Gly	2171	0.198	2080	0.207	HyE0151/+	53	0.981	1071-1091(21)
tRNA-Pro	1908	0.823	1813	0.866	HyE0174/-	254	0.830	192-272(81)
tRNA-Asn	1712	0.960	1676	0.981	HyE0051/-	236	0.885	189-287(99)
tRNA-Undet	1648	-	113	-	-	-	-	-
tRNA-Trp	1628	0.403	799	0.821	HyE1917/+	44	0.977	544-578(35)
tRNA-Glu	1363	0.795	1294	0.838	HyE0545/-	115	0.956	398-460(63)
tRNA-Ser	1266	0.573	1076	0.675	HyE0148/+	59	0.810	546-645(100)
tRNA-Thr	1136	0.670	821	0.927	HyE0003/-	94	0.925	350-449(100)
tRNA-His	1105	0.900	1058	0.940	HyE0986/+	102	0.921	185-232(48)
tRNA-Lys	1084	0.284	381	0.808	HyE1052/+	41	0.975	549-562(14)
tRNA-Val	874	0.713	745	0.836	HyE1196/+	68	0.985	468-530(63)
tRNA-Gln	856	0.709	716	0.848	HyE2398/-	49	0.979	471-530(60)
tRNA-Ile	561	0.825	469	0.987	HyE0971/-	53	1.000	601-607(7)
tRNA-Met	521	0.691	399	0.902	HyE1196/-	54	1.000	468-531(64)
tRNA-Ala	410	0.507	255	0.816	HyE0039/-	102	0.782	399-455(57)
tRNA-Sup	409	-	143	-	-	-	-	-
tRNA-Cys	227	0.423	132	0.727	HyE0151/-	52	0.961	1065-1092(28)
tRNA-Phe	210	0.452	119	0.798	HyE0151/-	52	0.980	1068-1092(25)
tRNA-Tyr	109	0.110	38	0.316	HyE0081/+	12	1.000	1225-1226(2)
tRNA-Asp	84	0.143	25	0.480	HyE0081/-	12	1.000	1247-1248(2)
tRNA-iMet	62	0.194	37	0.324	HyE0081/+	12	1.000	1247-1248(2)
tRNA-SeC	24	-	23	-	-	-	-	-
5S_rRNA	1287	0.887	1215	0.939	HyE0698/-	73	1.000	446-463(18)
Metazoa_SRP	513	0.922	509	0.929	HyE1473/-	78	0.948	151-159(9)
U5	343	0.883	327	0.927	HyE0881/+	70	0.986	550-581(32)
Histone3	255	0.071	62	0.290	HyE0368/+	18	0.765	5951-5971(21)
LSU_rRNA_eukarya	251	0.088	23	0.957	HyE0249/-	11	1.000	3428-3463(36)
SSU_rRNA_eukarya	226	0.102	23	1.000	HyE0522/-	13	0.917	5227-5248(22)
5_8S_rRNA	199	0.161	33	0.970	HyE0522/-	11	1.000	6853-6885(33)
U4	172	0.959	171	0.965	HyE0175/+	68	1.000	390-401(12)
U6	132	0.970	128	1.000	HyE0823/-	111	0.982	274-282(9)
U2	50	0.420	21	1.000	HyE0368/+	21	0.800	5787-5838(52)
U1	45	0.444	20	1.000	HyE0368/+	20	0.789	5812-5864(53)
U3	36	0.917	33	1.000	HyE0233/+	19	1.000	1315-1339(25)
K_chan_RES	14	-	-	-	-	-	-	-
RNaseP_nuc	13	-	12	-	-	-	-	-
U12	13	-	-	-	-	-	-	-
total	31651	0.568	24339	0.739	-	-	-	-

Table S24: Tandem array statistics for tRNAscan-SE and Rfam predictions in the *Hydractinia echinata* genome. "total" column includes counts from 16 Rfam families with fewer than 10 predictions that are not shown in the table. Zero values are represented as "-". "Tandem array" is abbreviated as "TA". See definition of TA in text. "eligible for TA count": number of predictions that occur on a sequence/strand with >= 10 predictions for the same family and so are eligible to be in a TA The four rightmost columns include information on the longest TA

for each family, if any. “count in TA”: total number of predictions in the largest tandem array for the family (X). “fraction within Dspan”: fraction of spaces between predictions in the TA that satisfy the spacing constraints in Dspan column (equal to N/(X − 1)). “spacer range and span (Dspan)”: minimum (min) and maximum (max) spacer lengths and range of the N spacers between adjacent predictions in the TA in format min − max(max − min + 1).

tRNA isotype or Rfam family	total count	fraction in TA	eligible for TA count	fraction of eligible in TA	sequence name/ strand	largest tandem array per family (maximum X) count in TA (X)	fraction within Dspan (N/(X-1))	spacer range and span (Dspan)
tRNA-Leu	3247	0.602	3084	0.634	HyS0119/+	176	0.909	244-300(57)
tRNA-Arg	2839	0.579	2087	0.788	HyS0172/+	95	0.787	402-459(58)
tRNA-Gln	2758	0.773	2583	0.825	HyS0582/+	89	0.773	307-334(28)
tRNA-Ser	2035	0.724	1872	0.787	HyS0340/+	75	0.838	510-553(44)
tRNA-Lys	1652	0.627	1390	0.745	HyS0340/-	74	0.918	535-564(30)
tRNA-Val	1358	0.780	1280	0.827	HyS0042/+	78	0.909	481-549(69)
tRNA-Asn	1319	0.818	1264	0.854	HyS0679/-	111	0.936	188-196(9)
tRNA-Pro	1107	0.643	975	0.730	HyS0303/+	49	0.750	588-608(21)
tRNA-Thr	1095	0.797	905	0.965	HyS0695/+	81	0.988	300-367(68)
tRNA-Undet	974	-	52	-	-	-	-	-
tRNA-Gly	940	0.564	836	0.634	HyS1685/-	54	0.906	201-281(81)
tRNA-His	844	0.823	787	0.883	HyS1728/-	65	0.953	216-234(19)
tRNA-Ala	764	0.627	667	0.718	HyS0144/+	58	0.772	427-513(87)
tRNA-Trp	724	0.519	475	0.792	HyS0259/-	29	0.750	684-727(44)
tRNA-Glu	653	0.504	541	0.608	HyS0933/+	58	0.930	448-465(18)
tRNA-Met	489	0.738	433	0.834	HyS0042/+	75	0.865	520-549(30)
tRNA-Sup	388	-	47	-	-	-	-	-
tRNA-Ile	268	0.743	211	0.943	HyS0846/+	40	1.000	619-623(5)
tRNA-Phe	139	0.360	70	0.714	HyS0138/-	17	1.000	1090-1091(2)
tRNA-Cys	126	0.302	58	0.655	HyS0138/-	18	1.000	1091-1092(2)
tRNA-Tyr	110	0.291	54	0.593	HyS1538/-	18	1.000	1207-1209(3)
tRNA-Asp	100	0.290	51	0.569	HyS1538/+	18	1.000	1229-1231(3)
tRNA-iMet	87	0.506	44	1.000	HyS1538/-	18	1.000	1229-1231(3)
tRNA-SeC	61	0.787	52	0.923	HyS0093/+	24	0.870	879-886(8)
5S_rRNA	1891	0.883	1813	0.921	HyS0219/+	79	0.974	431-488(58)
U1	219	0.731	171	0.936	HyS1307/+	40	0.974	488-494(7)
Histone3	195	0.051	30	0.333	HyS0385/+	10	0.889	5745-5830(86)
U5	167	0.880	161	0.913	HyS0475/-	56	0.945	544-560(17)
U4	124	0.823	122	0.836	HyS0767/-	70	1.000	372-396(25)
U2	65	0.154	10	1.000	HyS0385/+	10	0.889	5657-5684(28)
U6	53	1.000	53	1.000	HyS0009/-	53	1.000	292-304(13)
LSU_rRNA_eukarya	50	-	10	-	-	-	-	-
U3	45	0.533	26	0.923	HyS0031/-	13	1.000	1052-1053(2)
SSU_rRNA_eukarya	36	0.306	11	1.000	HyS0316/-	11	0.800	5236-5280(45)
Metazoa_SRP	35	0.371	22	0.591	HyS0834/+	13	1.000	1110-1155(46)
5_8S_rRNA	25	-	10	-	-	-	-	-
K_chan_RES	14	-	-	-	-	-	-	-
U12	10	-	-	-	-	-	-	-
total	27057	0.642	22257	0.781	-	-	-	-

Table S25: Tandem array statistics for tRNAscan-SE and Rfam predictions in the *Hydractinia symbiolongicarpus* genome. "total" column includes counts from 18 Rfam families with fewer than 10 predictions that are not shown in the table. Zero values are represented as "-". "Tandem array" is abbreviated as "TA". See definition of TA in text. "eligible for TA count": number of predictions that occur on a sequence/strand with >= 10 predictions for the same family and so are eligible to be in a TA The four rightmost columns include information on the longest TA for each family, if any. "count in TA": total number of predictions in the largest

tandem array for the family (X). “fraction within Dspan”: fraction of spaces between predictions in the TA that satisfy the spacing constraints in Dspan column (equal to $N/(X - 1)$). “spacer range and span (Dspan)”: minimum (min) and maximum (max) spacer lengths and range of the N spacers between adjacent predictions in the TA in format $\min - \max(\max - \min + 1)$.

	H. echinata	*H. symbiolongicarpus*
ANTP	26	28
CERS	3	1
LIM	6	5
POU	5	4
PRD	21	16
SINE	6*	5
TALE	4	5
Unclassified	11	7
**Total**	**82****	**71**

Table S26: Classification of homeobox genes identified in the *H. echinata* and *H. symbiolongicarpus* genomes.

*One protein in each dataset (HyE0051.123 and HyS0062.116), appears in a different clade to SINE in the phylogenetic trees but has a SIX domain. We have classified these as SINE proteins based on the presence of the SIX domain and the OrthoFinder results (Supplemental Table S27). These two proteins are homologs based on the pairwise alignment (98.5% similarity of homeodomain) (Supplemental Table S27). ** *H. echinata* has more homeobox proteins than *H. symbiolongicarpus* but some of these may be duplicates from different alleles of the same gene that were not removed from the primary assembly.

	CMFASW	3XPBS
Estimated number of cells captured	4,526	5,711
Mean reads per cell	78,818	126,224
Median genes per cell	811	772
Fraction of reads in cells	85.4%	81.1%
Total genes detected	17,138	17,967
Median UMI Counts per cell	1,872	2,344
Number of Reads	356,728,464	720,867,666
Reads mapped to genome	92.3%	84.4%

Table S28: Overall statistics for two 10X single-cell RNAseq libraries from *H. symbiolongicarpus* and final statistics from when the two libraries were combined. Statistics were generated by the 10X Cell Ranger pipeline version 7.0.1.

Table S9. DIAMOND BLASTp top hits and PANNZER2 annotations for all predicted gene models for *H. symbiolongicarpus* and *H. echinata*.

Table S11. OrthoFinder and CAFÉ results for the orthology analyses of 49 species.

Table S12. Combined PANNZER2 annotation for proteomes from 49 species included in orthology analyses including orthology cluster information for each protein.

Table S18. List of 38 unique high quality mature miRNA sequences from *H. echinata*. Duplicates highlighted in yellow.

Table S27. Classification and annotation for all *H. echinata* and *H. symbiolongicarpus* homeobox proteins including final class assignment, orthogroup ID, PANNZER2 annotations, and nr best BLAST hits. A pairwise alignment matrix of all *H. echinata* and *H. symbiolongicarpus* homeodomains is included in a separate tab.

Table S29. Marker genes that were used to annotate the *H. symbiolongicarpus* single cell RNAseq atlas clusters and associated publications.

Table S30. Cluster marker genes from the Seurat single-cell RNAseq analysis of *H. symbiolongicarpus*. Positive markers for all clusters were identified using the Seurat function FindAllMarkers using min.pct=0.25 and default parameters otherwise. The list was further filtered, keeping markers that had a pct1 > 0.25, a pct2 < 0.05, and an adjusted p-value cutoff of 10^{-200}. All duplicated markers (markers that showed up in multiple clusters) were also removed. Markers are annotated with PANNZER annotations and BLASTp top hits. Taxon-specificity annotation and Orthogroup ID and size from OrthoFinder2 analysis is also included for each marker. Abbreviations for cluster_id column: sprm (sperm), nem (nematocyte), enEP/ecEP (endodermal epithelial/ectodermal epithelial),

ISC (interstitial stem cell), prog (somatic progenitor), nb (nematoblast), Zgc (zymogen gland cell), Mgc (mucous gland cell).

Table S31. Primer sequences used for cloning genes for creating riboprobes for *in situ* hybridization in *H. symbiolongicarpus*.

Supplemental Data S1. Text file detailing how different transcriptomes were generated for *Hydractinia echinata* and *Hydractinia symbiolognicarpus*. Details include how assemblies were generated using Trinity, TopHat, and HISAT2/StringTie and includes parameters used to generate each assembly.

Supplemental Data S2. Text file detailing the pipeline for gene model prediction for both *Hydractinia* species. The file covers the steps used and the parameters used at each step. This pipeline involves PASA and Augustus.

Supplemental Data S3. Orthogroups_Dec_20.tsv is a tab separated text file that is an OrthoFinder results file. Each row contains the genes belonging to a single orthogroup. The genes from each orthogroup are organized into columns, one per species.

Supplemental Data S4. Orthogroups_SpeciesOverlaps.tsv is a tab separated text file that is an OrthoFinder results file that contains the number of orthogroups shared between each species-pair as a square matrix.

Supplemental Data S5. Orthogroups_UnassignedGenes_Dec20.tsv is a tab separated text file that is an OrthoFinder results file that is identical in format to Orthogroups.tsv but contains all of the genes that were not assigned to any orthogroup.

Supplemental Data S6. Orthogroups.GeneCount_dec_20.tsv is a tab separated text file that is an OrthoFinder results file that is identical in format to Orthogroups.csv but contains counts of the number of genes for each species in each orthogroup.

Supplemental Data S7. Orthogroups.txt is an OrthoFinder results file containing the orthogroups described in the Orthogroups_Dec_20.tsv file (Supplemental Data S3) but using the OrthoMCL output format.

Supplemental Data S8. Statistics_Overall.tsv is a tab separated text file that is an OrthoFinder results file that contains general statistics about orthogroup sizes and proportion of genes assigned to orthogroups.

Supplemental Data S9. Statistics_PerSpecies.tsv is a tab separated text file that is an OrthoFinder results file that contains the same information as the Statistics_Overall.tsv file (Supplemental Data S8) but for each individual species.

Supplemental Data S10. concat_trim.fa is a text file in fasta format that represents the final input matrix provided to IQ-TREE2. It is comprised of a subset of single copy ortholog (SCO) sequences from our orthogroup data set. These SCOs were chosen for their presence in at least 12 of 15 cnidarian species; four bilaterian and three non-bilaterian outgroup species that also contained these SCOs were included in the analysis. The final concatenated, aligned, and trimmed data set included sequences from 216 orthogroups, resulting in an alignment of 50,457 nucleotides.

Supplemental Data S11. concat_trim.fa.iqtree is a text file that is the main IQ-TREE2 output file. It includes a text representation of the final maximum likelihood tree.

Supplemental Data S12. r8s_simple_aque_root_with_chronogram.out is a text file that is the output from r8s.

Supplemental Data S13. orthogroup_queries.html is an html-formatted R markdown file that provides details of R code and information related to calculating the numbers of overlaps of orthogroups between major groups of cnidarians and bilaterians.

Supplemental Data S14. phylum_specific_and_unassigned.html is an html-formatted R markdown file that provides details of R code and information related to calculating the numbers of phylum specific and unassigned orthogroups.

Supplemental Data S15. unassigned_annotation.html is an html-formatted R markdown file that provides details of R code and information related to annotating unassigned genes from the OrthoFinder results.

Supplemental Data S16. NoTadh_r8s_gene_counts_sampled_FULL_input.txt is a text file that represents the full matrix of gene family sizes per species estimated by OrthoFinder for gene families present in the selected species for the CAFÉ analysis.

Supplemental Data S17. NoTadh_r8s_gene_counts_sampled_small_input.txt is a text file that represents the reduced matrix of gene family sizes per species estimated by OrthoFinder for gene families present in the selected species with fewer than 100 sequences per species. Before running CAFE to estimate ancestral gene family sizes and gene family gains/losses over the selected subtree, one first needs to estimate a value for lambda (λ), the symmetrical gene birth-death rate for the entire tree expressed in gains or losses per gene per million years. To estimate λ, it is recommended that only orthogroups with low variance in gene family size amongst taxa be used; this can be achieved by selecting those with fewer than 100 sequences per species.

Supplemental Data S18. NoTadh.constrained.FULL.cafe_test.sh: Script submitted to run CAFE to do actual estimates of evolutionary dynamics, using as input the full set of possible orthogroups inferred to be in the common ancestor of the included species (Supplemental Data S28) and the value for λ calculated using Supplemental Data S19.

Supplemental Data S19. NoTadh.constrained.LAMBDA.cafe_test.sh: Script submitted to run CAFE in order to infer the λ parameter (symmetrical gene birth-death rate) for our data, including an input tree and directing CAFE to analyze only those groups those orthogroups that meet requirements (see Supplemental Data S29). To estimate λ, it is recommended that only orthogroups with low variance in gene family size amongst taxa be used; this can be achieved by selecting those with less than 100 sequences per species.

Supplemental Data S20. NoTadh.constrained.CAFE.report.cafe: Report generated from raw CAFE output for the run using Supplemental Data S18, using accessory scripts included with the CAFE installation. Explanation of the data found in this file can be found in CAFE documentation: https://hahnlab.github.io/CAFE/src_docs/html/Report.html

Supplemental Data S21. NoTadh.constrained.FULL.log: Progress log produced during running of CAFE as specified in Supplemental Data S18.

Supplemental Data S22. NoTadh.constrained.LAMBDA.log: Progress log produced during running of CAFE as specified in Suppelmental Data S19 to estimate λ parameter.

Supplemental Data S23. NoTadh.constrained.LAMBDA.report.cafe: Report generated from raw CAFE output for the run using Supplemental Data S19, using accessory scripts included with the CAFE installation. Explanation of the data found in this file can be found in CAFE documentation: https://hahnlab.github.io/CAFE/src_docs/html/Report.html

Supplemental Data S24. NoTadh.constrained.summary_anc.txt: Output produced by running accessory scripts on output from script S18. Each row contains counts of members of each gene family present inferred to be present at the common ancestor (i.e. at each node). Node numbers are as designated in the tree on top of Supplemental Data S25.

Supplemental Data S25. NoTadh.constrained.summary_fams.txt: Output produced by running accessory scripts on output from Supplemental Data S18. This shows how many rapidly evolving families, and their identities, were found overall on the tree, and for each species (terminal branch) and on each internal branch on the input tree. Nodes and species are named in accordance with the version of the input tree rendered at the top of this file.

Supplemental Data S26. NoTadh.constrained.summary_node.txt: Output produced by running accessory scripts on output from Supplemental Data S18. This gives a per-node (or per-terminal-taxon) counts of expansions, contractions and significantly rapidly evolving families. Node numbers are as designated in the tree on top of Supplemental Data S25.

Supplemental Data S27. NoTadh.constrained.summary_pub.txt: "Publication friendly" per-species summary of the results in Supplemental Data S20 across all branches of the tree. Numbers in parentheses indicate counts of significantly rapidly evolving families in relevant categories.

Supplemental Data S28. README-gff.md is a file that provides detailed explanation of all *Hydractinia* RNA annotation GFF files (Supplemental Data S29-S36).

Supplemental Data S29. hech.rfam.detailed.gff is a gff file for Rfam predictions (all RNAs except tRNA) of *H. echinata*, with all metadata included.

Supplemental Data S30. hech.rfam.minimal.gff is a gff file Rfam predictions (all RNAs except tRNA) of *H. echinata*, without metadata.

Supplemental Data S31. hech.trna.detailed.gff is a gff file for tRNA predictions of *H. echinata*, with all metadata included.

Supplemental Data S32. hech.trna.minimal.gff is a gff file for for tRNA predictions of *H. echinata*, without metadata.

Supplemental Data S33. hsym.rfam.detailed.gff is a gff file for Rfam predictions (all RNAs except tRNA) of *H. symbiolongicarpus*, with all metadata included.

Supplemental Data S34. hsym.rfam.minimal.gff is a gff file for Rfam predictions (all RNAs except tRNA) of *H. symbiolongicarpus*, without metadata.

Supplemental Data S35. hsym.trna.detailed.gff is a gff file for tRNA predictions of *H. symbiolongicarpus*, with all metadata included.

Supplemental Data S36. hsym.trna.minimal.gff is a gff file for tRNA predictions of *H. symbiolongicarpus,* without metadata.

Supplemental Data S37. Hech_SuperTree.phy is a phylip-formatted text file containing the final 60 amino acid homeodomain alignment for *H. echinata* that was used for subsequent phylogenetic analyses.

Supplemental Data S38. Hysm_SuperTree.phy is a phylip-formatted text file containing the final 60 amino acid homeodomain alignment for *H. symbiolongicarpus* that was used for subsequent phylogenetic analyses.

Supplemental Data S39. RAxML_bipartitions.hech_Hbox is a RAXML generated maximum likelihood tree file for the homeobox genes from *H. echinata*. Bootstrap values are included.

Supplemental Data S40. RAxML_bipartitions.hsym_Hbox is a RAXML generated maximum likelihood tree file for the homeobox genes from *H. symbiolongicarpus*. Bootstrap values are included.

Supplemental Data S41. Hech_SuperTree_renamed.nex.con.tre is the final consensus Bayseian tree file for the homeobox genes from *H. echinata* generated by MRBAYES.

Supplemental Data S42. Hsym_SuperTree_renamed.nex.con.tre is the final consensus Bayseian tree file for the homeobox genes from *H. symbiolongicarpus* generated by MRBAYES.

Supplemental Data S43. ANTP_aln_Hech.phy is a phylip-formatted text file containing the final homeodomain alignment for the ANTP class of homeobox proteins for *H. echinata* that was used for subsequent phylogenetic analyses.

Supplemental Data S44. ANTP_aln_Hsym.phy is a phylip-formatted text file containing the final homeodomain alignment for the ANTP class of homeobox proteins for *H. symbiolongicarpus* that was used for subsequent phylogenetic analyses.

Supplemental Data S45. RAxML_bipartitions.hsym_ANTP is a RAXML generated maximum likelihood tree file for the ANTP class of homeobox proteins from *H. symbiolongicarpus*. Bootstrap values are included.

Supplemental Data S46. RAxML_bipartitions.hech_ANTP is a RAXML generated maximum likelihood tree file for the ANTP class of homeobox proteins from *H. echinata*. Bootstrap values are included.

Supplemental Data S47. ANTP_aln_hech.nex.con.tre is the final consensus Bayseian tree file for the ANTP class of homeobox proteins from *H. echinata* generated by MRBAYES.

Supplemental Data S48. ANTP_aln_Hsym.next.con.tre is the final consensus Bayseian tree file for the ANTP class of homeobox proteins from *H. symbiolongicarpus* generated by MRBAYES.

Supplemental Data S49. Supplemental_analysis_FINAL.docx is a word document that includes step-by-step descriptions of the computational analyses used to create the final filtered *H. symbiolongicarpus* single cell atlas.

Supplemental Code S1.tgz - Repository of code used to generate the results presented in this manuscript.

Supplemental Code S1 - generate_stats.pl script is a perl script that was used to generate summary statistics for the predicted gene models.

Supplemental Code S2 - overlapTranscripts.pl is a perl script that calculates intersection length for each gene model that had overlapping transcripts when RNAseq data was aligned to the gene models.

Supplemental Code S3 - calculate_overlap.pl is a perl script that calculates the percent transcript overlap for each gene in terms of length of the gene for each dataset when determining how many gene models had transcript support.

Supplemental Code S4 - calculate_multiple_overlap.pl is a perl script that processes multiple overlap files from the different transcript datasets for determining how many gene models had transcript support.

Supplemental Code S5 - select_longest_isoform.html is an html-formatted python script that uses the lists of proteins that correspond to specific genes in each proteome, along with the input proteomes themselves, to select the longest isoform per gene.

Supplemental Code S6 - filter_filln_og.py is a python script that filters out non-single-copy orthogroupss.

Supplemental Code S7 - orthoFinderToOrthogroup.pl is a perl script used together with prepMsynt.pl (Supplemental Code S8) to calculate the number of gene copies of each orthogroup for each species for the synteny analysis.

Supplemental Code S8 - prepMsynt.pl is a perl script is a perl script used together with OrthoFinderToOrthogroup.pl (Supplemental Code S7) to calculate the number of gene copies of each orthogroup for each species for the synteny analysis.

Supplemental Code S9 - plot_msynt.R is an R script that performs several major functions for the synteny analysis. First, it calculates the number of shared orthogroups for all pairwise scaffolds and clusters the resulting count matrix by hierarchical clustering (using the function 'hclust' with ward.D2 algorithm).

Supplemental Code S10 - find_common_og.R is and R script that is used to further examine and extract highly conserved clusters in the synteny analysis.

Supplemental Code S11 - age_plot_out.R is an R script that produced repeat landscape plots for the repeats analysis together with age_plot_divsum.R (Supplemental Code S12).

Supplemental Code S12 - age_plot_divsum.R is an R script that produced repeat landscape plots for the repeats analysis together with age_plot_out.R (Supplemental Code S11).

REFERENCES

Almagro Armenteros JJ, Tsirigos KD, Sønderby CK, Petersen TN, Winther O, Brunak S, von Heijne G, Nielsen H. 2019. SignalP 5.0 improves signal peptide predictions using deep neural networks. *Nat Biotechnol* **37**: 420–423.

Altekar G, Dwarkadas S, Huelsenbeck JP, Ronquist F. 2004. Parallel Metropolis coupled Markov chain Monte Carlo for Bayesian phylogenetic inference. *Bioinformatics* **20**: 407–415.

Altschul SF, Gish W, Miller W, Myers EW, Lipman DJ. 1990. Basic local alignment search tool. *Journal of Molecular Biology* **215**: 403–410.

Anders S, Pyl PT, Huber W. 2015. HTSeq—a Python framework to work with high-throughput sequencing data. *Bioinformatics* **31**: 166–169.

Benson G. 1999. Tandem repeats finder: a program to analyze DNA sequences. *Nucleic Acids Research* **27**: 573–580.

Bolger AM, Lohse M, Usadel B. 2014. Trimmomatic: a flexible trimmer for Illumina sequence data. *Bioinformatics* **30**: 2114–2120.

Buchfink B, Xie C, Huson DH. 2015. Fast and sensitive protein alignment using DIAMOND. *Nature Methods* **12**: 59–60.

Camacho C, Coulouris G, Avagyan V, Ma N, Papadopoulos J, Bealer K, Madden TL. 2009. BLAST+: architecture and applications. *BMC Bioinformatics* **10**: 421.

Capella-Gutiérrez S, Silla-Martínez JM, Gabaldón T. 2009. trimAl: a tool for automated alignment trimming in large-scale phylogenetic analyses. *Bioinformatics* **25**: 1972–1973.

Cartwright P, Collins A. 2007. Fossils and phylogenies: integrating multiple lines of evidence to investigate the origin of early major metazoan lineages. *Integrative and Comparative Biology* **47**: 744–751.

Chan PP, Lin BY, Mak AJ, Lowe TM. 2021. tRNAscan-SE 2.0: improved detection and functional

classification of transfer RNA genes. *Nucleic Acids Research* **49**: 9077–9096.

Chang ES, Neuhof M, Rubinstein ND, Diamant A, Philippe H, Huchon D, Cartwright P. 2015. Genomic insights into the evolutionary origin of Myxozoa within Cnidaria. *Proceedings of the National Academy of Sciences* **112**: 14912–14917.

Chapman JA, Kirkness EF, Simakov O, Hampson SE, Mitros T, Weinmaier T, Rattei T, Balasubramanian PG, Borman J, Busam D, et al. 2010. The dynamic genome of Hydra. *Nature* **464**: 592–596.

Chin C-S, Alexander DH, Marks P, Klammer AA, Drake J, Heiner C, Clum A, Copeland A, Huddleston J, Eichler EE, et al. 2013. Nonhybrid, finished microbial genome assemblies from long-read SMRT sequencing data. *Nature Methods* **10**: 563–569.

Chin C-S, Peluso P, Sedlazeck FJ, Nattestad M, Concepcion GT, Clum A, Dunn C, O'Malley R, Figueroa-Balderas R, Morales-Cruz A, et al. 2016. Phased diploid genome assembly with single-molecule real-time sequencing. *Nat Methods* **13**: 1050–1054.

Darriba D, Taboada GL, Doallo R, Posada D. 2011. ProtTest 3: fast selection of best-fit models of protein evolution. *Bioinformatics* **27**: 1164–1165.

De Bie T, Cristianini N, Demuth JP, Hahn MW. 2006. CAFE: a computational tool for the study of gene family evolution. *Bioinformatics* **22**: 1269–1271.

DuBuc TQ, Ryan JF, Shinzato C, Satoh N, Martindale MQ. 2012. Coral Comparative Genomics Reveal Expanded Hox Cluster in the Cnidarian–Bilaterian Ancestor. *Integrative and Comparative Biology* **52**: 835–841.

Edgar RC. 2004. MUSCLE: multiple sequence alignment with high accuracy and high throughput. *Nucleic Acids Research* **32**: 1792–1797.

Emms DM, Kelly S. 2019. OrthoFinder: phylogenetic orthology inference for comparative genomics.

Genome Biol **20**: 238.

Falda M, Toppo S, Pescarolo A, Lavezzo E, Di Camillo B, Facchinetti A, Cilia E, Velasco R, Fontana P. 2012. Argot2: a large scale function prediction tool relying on semantic similarity of weighted Gene Ontology terms. *BMC Bioinformatics* **13**: S14.

Frank U, Nicotra ML, Schnitzler CE. 2020. The colonial cnidarian Hydractinia. *EvoDevo* **11**: 7.

Friedländer MR, Mackowiak SD, Li N, Chen W, Rajewsky N. 2012. miRDeep2 accurately identifies known and hundreds of novel microRNA genes in seven animal clades. *Nucleic Acids Research* **40**: 37–52.

Gasteiger E, Gattiker A, Hoogland C, Ivanyi I, Appel RD, Bairoch A. 2003. ExPASy: the proteomics server for in-depth protein knowledge and analysis. *Nucleic Acids Research* **31**: 3784–3788.

Gehrke AR, Neverett E, Luo Y-J, Brandt A, Ricci L, Hulett RE, Gompers A, Ruby JG, Rokhsar DS, Reddien PW, et al. 2019. Acoel genome reveals the regulatory landscape of whole-body regeneration. *Science* **363**: eaau6173.

Gnerre S, MacCallum I, Przybylski D, Ribeiro FJ, Burton JN, Walker BJ, Sharpe T, Hall G, Shea TP, Sykes S, et al. 2011. High-quality draft assemblies of mammalian genomes from massively parallel sequence data. *Proceedings of the National Academy of Sciences* **108**: 1513–1518.

Gold DA, Katsuki T, Li Y, Yan X, Regulski M, Ibberson D, Holstein T, Steele RE, Jacobs DK, Greenspan RJ. 2019. The genome of the jellyfish Aurelia and the evolution of animal complexity. *Nature Ecology & Evolution* **3**: 96–104.

Grabherr MG, Haas BJ, Yassour M, Levin JZ, Thompson DA, Amit I, Adiconis X, Fan L, Raychowdhury R, Zeng Q, et al. 2011. Trinity: reconstructing a full-length transcriptome without a genome from RNA-Seq data. *Nat Biotechnol* **29**: 644–652.

Grohme MA, Schloissnig S, Rozanski A, Pippel M, Young GR, Winkler S, Brandl H, Henry I, Dahl A,

Powell S, et al. 2018. The genome of Schmidtea mediterranea and the evolution of core cellular mechanisms. *Nature* **554**: 56–61.

Haas BJ, Delcher AL, Mount SM, Wortman JR, Smith Jr RK, Hannick LI, Maiti R, Ronning CM, Rusch DB, Town CD, et al. 2003. Improving the Arabidopsis genome annotation using maximal transcript alignment assemblies. *Nucleic Acids Research* **31**: 5654–5666.

Haas BJ, Papanicolaou A, Yassour M, Grabherr M, Blood PD, Bowden J, Couger MB, Eccles D, Li B, Lieber M, et al. 2013. De novo transcript sequence reconstruction from RNA-seq using the Trinity platform for reference generation and analysis. *Nat Protoc* **8**: 1494–1512.

Hamosh A, Scott AF, Amberger JS, Bocchini CA, McKusick VA. 2005. Online Mendelian Inheritance in Man (OMIM), a knowledgebase of human genes and genetic disorders. *Nucleic Acids Research* **33**: D514–D517.

Han MV, Thomas GWC, Lugo-Martinez J, Hahn MW. 2013. Estimating Gene Gain and Loss Rates in the Presence of Error in Genome Assembly and Annotation Using CAFE 3. *Molecular Biology and Evolution* **30**: 1987–1997.

Hare EE, Johnston JS. 2011. Genome Size Determination Using Flow Cytometry of Propidium Iodide-Stained Nuclei. In *Molecular Methods for Evolutionary Genetics* (eds. V. Orgogozo and M.V. Rockman), *Methods in Molecular Biology*, pp. 3–12, Humana Press, Totowa, NJ https://doi.org/10.1007/978-1-61779-228-1_1 (Accessed May 4, 2021).

Huelsenbeck JP, Ronquist F. 2001. MRBAYES: Bayesian inference of phylogenetic trees. *Bioinformatics* **17**: 754–755.

Jones P, Binns D, Chang H-Y, Fraser M, Li W, McAnulla C, McWilliam H, Maslen J, Mitchell A, Nuka G, et al. 2014. InterProScan 5: genome-scale protein function classification. *Bioinformatics* **30**: 1236–1240.

Kalvari I, Argasinska J, Quinones-Olvera N, Nawrocki EP, Rivas E, Eddy SR, Bateman A, Finn RD, Petrov AI. 2018a. Rfam 13.0: shifting to a genome-centric resource for non-coding RNA families. *Nucleic Acids Research* **46**: D335–D342.

Kalvari I, Nawrocki EP, Argasinska J, Quinones-Olvera N, Finn RD, Bateman A, Petrov AI. 2018b. Non-Coding RNA Analysis Using the Rfam Database. *Current Protocols in Bioinformatics* **62**: e51.

Kalyaanamoorthy S, Minh BQ, Wong TKF, von Haeseler A, Jermiin LS. 2017. ModelFinder: fast model selection for accurate phylogenetic estimates. *Nat Methods* **14**: 587–589.

Kapitonov VV, Jurka J. 2003. A Novel Class of SINE Elements Derived from 5S rRNA. *Molecular Biology and Evolution* **20**: 694–702.

Katoh K, Misawa K, Kuma K, Miyata T. 2002. MAFFT: a novel method for rapid multiple sequence alignment based on fast Fourier transform. *Nucleic Acids Research* **30**: 3059–3066.

Kayal E, Bentlage B, Cartwright P, Yanagihara AA, Lindsay DJ, Hopcroft RR, Collins AG. 2015. Phylogenetic analysis of higher-level relationships within Hydroidolina (Cnidaria: Hydrozoa) using mitochondrial genome data and insight into their mitochondrial transcription. *PeerJ* **3**: e1403.

Kayal E, Bentlage B, Sabrina Pankey M, Ohdera AH, Medina M, Plachetzki DC, Collins AG, Ryan JF. 2018. Phylogenomics provides a robust topology of the major cnidarian lineages and insights on the origins of key organismal traits. *BMC Evol Biol* **18**: 68.

Kearse M, Moir R, Wilson A, Stones-Havas S, Cheung M, Sturrock S, Buxton S, Cooper A, Markowitz S, Duran C, et al. 2012. Geneious Basic: An integrated and extendable desktop software platform for the organization and analysis of sequence data. *Bioinformatics* **28**: 1647–1649.

Kent WJ. 2002. BLAT—The BLAST-Like Alignment Tool. *Genome Res* **12**: 656–664.

Khalturin K, Shinzato C, Khalturina M, Hamada M, Fujie M, Koyanagi R, Kanda M, Goto H, Anton-Erxleben F, Toyokawa M, et al. 2019. Medusozoan genomes inform the evolution of the jellyfish body plan. *Nature Ecology & Evolution* **3**: 811–822.

Kim D, Langmead B, Salzberg SL. 2015. HISAT: a fast spliced aligner with low memory requirements. *Nat Methods* **12**: 357–360.

Koren S, Walenz BP, Berlin K, Miller JR, Bergman NH, Phillippy AM. 2017. Canu: scalable and accurate long-read assembly via adaptive k-mer weighting and repeat separation. *Genome Res* **27**: 722–736.

Koskinen P, Törönen P, Nokso-Koivisto J, Holm L. 2015. PANNZER: high-throughput functional annotation of uncharacterized proteins in an error-prone environment. *Bioinformatics* **31**: 1544–1552.

Kramerov DA, Vassetzky NS. 2005. Short Retroposons in Eukaryotic Genomes. In *International Review of Cytology*, Vol. 247 of *A Survey of Cell Biology*, pp. 165–221, Academic Press https://www.sciencedirect.com/science/article/pii/S0074769605470047 (Accessed May 11, 2022).

Krogh A, Larsson B, von Heijne G, Sonnhammer ELL. 2001. Predicting transmembrane protein topology with a hidden markov model: application to complete genomes11Edited by F. Cohen. *Journal of Molecular Biology* **305**: 567–580.

Kurtz S, Phillippy A, Delcher AL, Smoot M, Shumway M, Antonescu C, Salzberg SL. 2004. Versatile and open software for comparing large genomes. *Genome Biol* **5**: R12.

Laslett D, Canbäck B. 2008. ARWEN: a program to detect tRNA genes in metazoan mitochondrial nucleotide sequences. *Bioinformatics* **24**: 172–175.

Laumer CE, Fernández R, Lemer S, Combosch D, Kocot KM, Riesgo A, Andrade SCS, Sterrer W,

Sørensen MV, Giribet G. 2019. Revisiting metazoan phylogeny with genomic sampling of all phyla. *Proceedings of the Royal Society B: Biological Sciences* **286**: 20190831.

Lavezzo E, Falda M, Fontana P, Bianco L, Toppo S. 2016. Enhancing protein function prediction with taxonomic constraints – The Argot2.5 web server. *Methods* **93**: 15–23.

Leclère L, Horin C, Chevalier S, Lapébie P, Dru P, Peron S, Jager M, Condamine T, Pottin K, Romano S, et al. 2019. The genome of the jellyfish Clytia hemisphaerica and the evolution of the cnidarian life-cycle. *Nature Ecology & Evolution* **3**: 801–810.

Leng N, Kendziorski C. 2022. EBSeqHMM: Bayesian analysis for identifying gene or isoform expression changes in ordered RNA-seq experiments.

Li H, Durbin R. 2009. Fast and accurate short read alignment with Burrows–Wheeler transform. *Bioinformatics* **25**: 1754–1760.

Lowe TM, Eddy SR. 1997. tRNAscan-SE: A Program for Improved Detection of Transfer RNA Genes in Genomic Sequence. *Nucleic Acids Research* **25**: 955–964.

Marçais G, Kingsford C. 2011. A fast, lock-free approach for efficient parallel counting of occurrences of k-mers. *Bioinformatics* **27**: 764–770.

Markham NR, Zuker M. 2008. UNAFold. In *Bioinformatics: Structure, Function and Applications* (ed. J.M. Keith), *Methods in Molecular Biology™*, pp. 3–31, Humana Press, Totowa, NJ https://doi.org/10.1007/978-1-60327-429-6_1 (Accessed May 11, 2022).

Marlétaz F, Peijnenburg KTCA, Goto T, Satoh N, Rokhsar DS. 2019. A New Spiralian Phylogeny Places the Enigmatic Arrow Worms among Gnathiferans. *Current Biology* **29**: 312-318.e3.

Martin M. 2011. Cutadapt removes adapter sequences from high-throughput sequencing reads. *EMBnet.journal* **17**: 10–12.

Maxwell EK, Schnitzler CE, Havlak P, Putnam NH, Nguyen A-D, Moreland RT, Baxevanis AD. 2014.

Evolutionary profiling reveals the heterogeneous origins of classes of human disease genes: implications for modeling disease genetics in animals. *BMC Evol Biol* **14**: 212.

Minh BQ, Schmidt HA, Chernomor O, Schrempf D, Woodhams MD, von Haeseler A, Lanfear R. 2020. IQ-TREE 2: New Models and Efficient Methods for Phylogenetic Inference in the Genomic Era. *Molecular Biology and Evolution* **37**: 1530–1534.

Mistry J, Finn RD, Eddy SR, Bateman A, Punta M. 2013. Challenges in homology search: HMMER3 and convergent evolution of coiled-coil regions. *Nucleic Acids Research* **41**: e121.

Nawrocki EP, Eddy SR. 2013. Infernal 1.1: 100-fold faster RNA homology searches. *Bioinformatics* **29**: 2933–2935.

Nishihara H, Plazzi F, Passamonti M, Okada N. 2016. MetaSINEs: Broad Distribution of a Novel SINE Superfamily in Animals. *Genome Biology and Evolution* **8**: 528–539.

Pastrana CC, DeBiasse MB, Ryan JF. 2019. Sponges Lack ParaHox Genes. *Genome Biology and Evolution* **11**: 1250–1257.

Pertea M, Pertea GM, Antonescu CM, Chang T-C, Mendell JT, Salzberg SL. 2015. StringTie enables improved reconstruction of a transcriptome from RNA-seq reads. *Nat Biotechnol* **33**: 290–295.

Putnam NH, Srivastava M, Hellsten U, Dirks B, Chapman J, Salamov A, Terry A, Shapiro H, Lindquist E, Kapitonov VV, et al. 2007. Sea Anemone Genome Reveals Ancestral Eumetazoan Gene Repertoire and Genomic Organization. *Science* **317**: 86–94.

Ranallo-Benavidez TR, Jaron KS, Schatz MC. 2020. GenomeScope 2.0 and Smudgeplot for reference-free profiling of polyploid genomes. *Nat Commun* **11**: 1432.

Ronquist F, Huelsenbeck JP. 2003. MrBayes 3: Bayesian phylogenetic inference under mixed models. *Bioinformatics* **19**: 1572–1574.

Ryan JF, Pang K, Mullikin JC, Martindale MQ, Baxevanis AD, NISC Comparative Sequencing

Program. 2010. The homeodomain complement of the ctenophore Mnemiopsis leidyi suggests that Ctenophora and Porifera diverged prior to the ParaHoxozoa. *EvoDevo* **1**: 9.

Sanderson MJ. 2003. r8s: inferring absolute rates of molecular evolution and divergence times in the absence of a molecular clock. *Bioinformatics* **19**: 301–302.

Seppey M, Manni M, Zdobnov EM. 2019. BUSCO: Assessing Genome Assembly and Annotation Completeness. In *Gene Prediction: Methods and Protocols* (ed. M. Kollmar), *Methods in Molecular Biology*, pp. 227–245, Springer, New York, NY https://doi.org/10.1007/978-1-4939-9173-0_14 (Accessed May 11, 2022).

Siebert S, Farrell JA, Cazet JF, Abeykoon Y, Primack AS, Schnitzler CE, Juliano CE. 2019. Stem cell differentiation trajectories in Hydra resolved at single-cell resolution. *Science* **365**: eaav9314.

Stamatakis A. 2014. RAxML version 8: a tool for phylogenetic analysis and post-analysis of large phylogenies. *Bioinformatics* **30**: 1312–1313.

Stanke M, Waack S. 2003. Gene prediction with a hidden Markov model and a new intron submodel. *Bioinformatics* **19**: ii215–ii225.

Stuart T, Butler A, Hoffman P, Hafemeister C, Papalexi E, Mauck WM, Hao Y, Stoeckius M, Smibert P, Satija R. 2019. Comprehensive Integration of Single-Cell Data. *Cell* **177**: 1888-1902.e21.

Sun F-J, Fleurdépine S, Bousquet-Antonelli C, Caetano-Anollés G, Deragon J-M. 2007. Common evolutionary trends for SINE RNA structures. *Trends in Genetics* **23**: 26–33.

Thomas JM, Horspool D, Brown G, Tcherepanov V, Upton C. 2007. GraphDNA: a Java program for graphical display of DNA composition analyses. *BMC Bioinformatics* **8**: 21.

Török A, Schiffer PH, Schnitzler CE, Ford K, Mullikin JC, Baxevanis AD, Bacic A, Frank U, Gornik SG. 2016. The cnidarian Hydractinia echinata employs canonical and highly adapted histones to pack its DNA. *Epigenetics & Chromatin* **9**: 36.

Törönen P, Medlar A, Holm L. 2018. PANNZER2: a rapid functional annotation web server. *Nucleic Acids Research* **46**: W84–W88.

Walker BJ, Abeel T, Shea T, Priest M, Abouelliel A, Sakthikumar S, Cuomo CA, Zeng Q, Wortman J, Young SK, et al. 2014. Pilon: An Integrated Tool for Comprehensive Microbial Variant Detection and Genome Assembly Improvement. *PLOS ONE* **9**: e112963.

Wickham H, François R, Henry L, Müller K. 2022. dplyr: A Grammar of Data Manipulation. https://dplyr.tidyverse.org, https://github.com/tidyverse/dplyr.

Wong WY, Simakov O, Bridge DM, Cartwright P, Bellantuono AJ, Kuhn A, Holstein TW, David CN, Steele RE, Martínez DE. 2019. Expansion of a single transposable element family is associated with genome-size increase and radiation in the genus Hydra. *Proceedings of the National Academy of Sciences* **116**: 22915–22917.

Wu TD, Reeder J, Lawrence M, Becker G, Brauer MJ. 2016. GMAP and GSNAP for Genomic Sequence Alignment: Enhancements to Speed, Accuracy, and Functionality. In *Statistical Genomics: Methods and Protocols* (eds. E. Mathé and S. Davis), *Methods in Molecular Biology*, pp. 283–334, Springer, New York, NY https://doi.org/10.1007/978-1-4939-3578-9_15 (Accessed May 11, 2022).

Yu G, Smith DK, Zhu H, Guan Y, Lam TT-Y. 2017. ggtree: an r package for visualization and annotation of phylogenetic trees with their covariates and other associated data. *Methods in Ecology and Evolution* **8**: 28–36.

Zacharias H, Anokhin B, Khalturin K, Bosch TCG. 2004. Genome sizes and chromosomes in the basal metazoan Hydra. *Zoology* **107**: 219–227.

Zheng GXY, Terry JM, Belgrader P, Ryvkin P, Bent ZW, Wilson R, Ziraldo SB, Wheeler TD, McDermott GP, Zhu J, et al. 2017. Massively parallel digital transcriptional profiling of single

cells. *Nat Commun* **8**: 14049.

Zimmermann B, Montenegro JD, Robb SMC, Fropf WJ, Weilguny L, He S, Chen S, Lovegrove-Walsh J, Hill EM, Chen C-Y, et al. 2023. Topological structures and syntenic conservation in sea anemone genomes. *Nat Commun* **14**: 8270.
